# Global, Regional, and National Cancer Incidence, Mortality, Years of Life Lost, Years Lived With Disability, and Disability-Adjusted Life-Years for 29 Cancer Groups, 1990 to 2017: A Systematic Analysis for the Global Burden of Disease Study

**DOI:** 10.1001/jamaoncol.2019.2996

**Published:** 2019-09-27

**Authors:** Global Burden of Disease Cancer Collaboration, Christina Fitzmaurice, Degu Abate, Naghmeh Abbasi, Hedayat Abbastabar, Foad Abd-Allah, Omar Abdel-Rahman, Ahmed Abdelalim, Amir Abdoli, Ibrahim Abdollahpour, Abdishakur S. M. Abdulle, Nebiyu Dereje Abebe, Haftom Niguse Abraha, Laith Jamal Abu-Raddad, Ahmed Abualhasan, Isaac Akinkunmi Adedeji, Shailesh M. Advani, Mohsen Afarideh, Mahdi Afshari, Mohammad Aghaali, Dominic Agius, Sutapa Agrawal, Ayat Ahmadi, Elham Ahmadian, Ehsan Ahmadpour, Muktar Beshir Ahmed, Mohammad Esmaeil Akbari, Tomi Akinyemiju, Ziyad Al-Aly, Assim M. AlAbdulKader, Fares Alahdab, Tahiya Alam, Genet Melak Alamene, Birhan Tamene T. Alemnew, Kefyalew Addis Alene, Cyrus Alinia, Vahid Alipour, Syed Mohamed Aljunid, Fatemeh Allah Bakeshei, Majid Abdulrahman Hamad Almadi, Amir Almasi-Hashiani, Ubai Alsharif, Shirina Alsowaidi, Nelson Alvis-Guzman, Erfan Amini, Saeed Amini, Yaw Ampem Amoako, Zohreh Anbari, Nahla Hamed Anber, Catalina Liliana Andrei, Mina Anjomshoa, Fereshteh Ansari, Ansariadi Ansariadi, Seth Christopher Yaw Appiah, Morteza Arab-Zozani, Jalal Arabloo, Zohreh Arefi, Olatunde Aremu, Habtamu Abera Areri, Al Artaman, Hamid Asayesh, Ephrem Tsegay Asfaw, Alebachew Fasil Ashagre, Reza Assadi, Bahar Ataeinia, Hagos Tasew Atalay, Zerihun Ataro, Suleman Atique, Marcel Ausloos, Leticia Avila-Burgos, Euripide F. G. A. Avokpaho, Ashish Awasthi, Nefsu Awoke, Beatriz Paulina Ayala Quintanilla, Martin Amogre Ayanore, Henok Tadesse Ayele, Ebrahim Babaee, Umar Bacha, Alaa Badawi, Mojtaba Bagherzadeh, Eleni Bagli, Senthilkuimar Balakrishnan, Abbas Balouchi, Till Winfried Bärnighausen, Robert J. Battista, Masoud Behzadifar, Meysam Behzadifar, Bayu Begashaw Bekele, Yared Belete Belay, Yaschilal Muche Belayneh, Kathleen Kim Sachiko Berfield, Adugnaw Berhane, Eduardo Bernabe, Mircea Beuran, Nickhill Bhakta, Krittika Bhattacharyya, Belete Biadgo, Ali Bijani, Muhammad Shahdaat Bin Sayeed, Charles Birungi, Catherine Bisignano, Helen Bitew, Tone Bjørge, Archie Bleyer, Kassawmar Angaw Bogale, Hunduma Amensisa Bojia, Antonio M. Borzì, Cristina Bosetti, Ibrahim R. Bou-Orm, Hermann Brenner, Jerry D. Brewer, Andrey Nikolaevich Briko, Nikolay Ivanovich Briko, Maria Teresa Bustamante-Teixeira, Zahid A. Butt, Giulia Carreras, Juan J. Carrero, Félix Carvalho, Clara Castro, Franz Castro, Ferrán Catalá-López, Ester Cerin, Yazan Chaiah, Wagaye Fentahun Chanie, Vijay Kumar Chattu, Pankaj Chaturvedi, Neelima Singh Chauhan, Mohammad Chehrazi, Peggy Pei-Chia Chiang, Tesfaye Yitna Chichiabellu, Onyema Greg Chido-Amajuoyi, Odgerel Chimed-Ochir, Jee-Young J. Choi, Devasahayam J. Christopher, Dinh-Toi Chu, Maria-Magdalena Constantin, Vera M. Costa, Emanuele Crocetti, Christopher Stephen Crowe, Maria Paula Curado, Saad M. A. Dahlawi, Giovanni Damiani, Amira Hamed Darwish, Ahmad Daryani, José das Neves, Feleke Mekonnen Demeke, Asmamaw Bizuneh Demis, Birhanu Wondimeneh Demissie, Gebre Teklemariam Demoz, Edgar Denova-Gutiérrez, Afshin Derakhshani, Kalkidan Solomon Deribe, Rupak Desai, Beruk Berhanu Desalegn, Melaku Desta, Subhojit Dey, Samath Dhamminda Dharmaratne, Meghnath Dhimal, Daniel Diaz, Mesfin Tadese Tadese Dinberu, Shirin Djalalinia, David Teye Doku, Thomas M. Drake, Manisha Dubey, Eleonora Dubljanin, Eyasu Ejeta Duken, Hedyeh Ebrahimi, Andem Effiong, Aziz Eftekhari, Iman El Sayed, Maysaa El Sayed Zaki, Shaimaa I. El-Jaafary, Ziad El-Khatib, Demelash Abewa Elemineh, Hajer Elkout, Richard G. Ellenbogen, Aisha Elsharkawy, Mohammad Hassan Emamian, Daniel Adane Endalew, Aman Yesuf Endries, Babak Eshrati, Ibtihal Fadhil, Vahid Fallah, Mahbobeh Faramarzi, Mahdieh Abbasalizad Farhangi, Andrea Farioli, Farshad Farzadfar, Netsanet Fentahun, Eduarda Fernandes, Garumma Tolu Feyissa, Irina Filip, Florian Fischer, James L. Fisher, Lisa M. Force, Masoud Foroutan, Marisa Freitas, Takeshi Fukumoto, Neal D. Futran, Silvano Gallus, Fortune Gbetoho Gankpe, Reta Tsegaye Gayesa, Tsegaye Tewelde Gebrehiwot, Gebreamlak Gebremedhn Gebremeskel, Getnet Azeze Gedefaw, Belayneh K. Gelaw, Birhanu Geta, Sefonias Getachew, Kebede Embaye Gezae, Mansour Ghafourifard, Alireza Ghajar, Ahmad Ghashghaee, Asadollah Gholamian, Paramjit Singh Gill, Themba T. G. Ginindza, Alem Girmay, Muluken Gizaw, Ricardo Santiago Gomez, Sameer Vali Gopalani, Giuseppe Gorini, Bárbara Niegia Garcia Goulart, Ayman Grada, Maximiliano Ribeiro Guerra, Andre Luiz Sena Guimaraes, Prakash C. Gupta, Rahul Gupta, Kishor Hadkhale, Arvin Haj-Mirzaian, Arya Haj-Mirzaian, Randah R. Hamadeh, Samer Hamidi, Lolemo Kelbiso Hanfore, Josep Maria Haro, Milad Hasankhani, Amir Hasanzadeh, Hamid Yimam Hassen, Roderick J. Hay, Simon I. Hay, Andualem Henok, Nathaniel J. Henry, Claudiu Herteliu, Hagos D. Hidru, Chi Linh Hoang, Michael K. Hole, Praveen Hoogar, Nobuyuki Horita, H. Dean Hosgood, Mostafa Hosseini, Mehdi Hosseinzadeh, Mihaela Hostiuc, Sorin Hostiuc, Mowafa Househ, Mohammedaman Mama Hussen, Bogdan Ileanu, Milena D. Ilic, Kaire Innos, Seyed Sina Naghibi Irvani, Kufre Robert Iseh, Sheikh Mohammed Shariful Islam, Farhad Islami, Nader Jafari Balalami, Morteza Jafarinia, Leila Jahangiry, Mohammad Ali Jahani, Nader Jahanmehr, Mihajlo Jakovljevic, Spencer L. James, Mehdi Javanbakht, Sudha Jayaraman, Sun Ha Jee, Ensiyeh Jenabi, Ravi Prakash Jha, Jost B. Jonas, Jitendra Jonnagaddala, Tamas Joo, Suresh Banayya Jungari, Mikk Jürisson, Ali Kabir, Farin Kamangar, André Karch, Narges Karimi, Ansar Karimian, Amir Kasaeian, Gebremicheal Gebreslassie Kasahun, Belete Kassa, Tesfaye Dessale Kassa, Mesfin Wudu Kassaw, Anil Kaul, Peter Njenga Keiyoro, Abraham Getachew Kelbore, Amene Abebe Kerbo, Yousef Saleh Khader, Maryam Khalilarjmandi, Ejaz Ahmad Khan, Gulfaraz Khan, Young-Ho Khang, Khaled Khatab, Amir Khater, Maryam Khayamzadeh, Maryam Khazaee-Pool, Salman Khazaei, Abdullah T. Khoja, Mohammad Hossein Khosravi, Jagdish Khubchandani, Neda Kianipour, Daniel Kim, Yun Jin Kim, Adnan Kisa, Sezer Kisa, Katarzyna Kissimova-Skarbek, Hamidreza Komaki, Ai Koyanagi, Kristopher J. Krohn, Burcu Kucuk Bicer, Nuworza Kugbey, Vivek Kumar, Desmond Kuupiel, Carlo La Vecchia, Deepesh P. Lad, Eyasu Alem Lake, Ayenew Molla Lakew, Dharmesh Kumar Lal, Faris Hasan Lami, Qing Lan, Savita Lasrado, Paolo Lauriola, Jeffrey V. Lazarus, James Leigh, Cheru Tesema Leshargie, Yu Liao, Miteku Andualem Limenih, Stefan Listl, Alan D. Lopez, Platon D. Lopukhov, Raimundas Lunevicius, Mohammed Madadin, Sameh Magdeldin, Hassan Magdy Abd El Razek, Azeem Majeed, Afshin Maleki, Reza Malekzadeh, Ali Manafi, Navid Manafi, Wondimu Ayele Manamo, Morteza Mansourian, Mohammad Ali Mansournia, Lorenzo Giovanni Mantovani, Saman Maroufizadeh, Santi Martini S. Martini, Tivani Phosa Mashamba-Thompson, Benjamin Ballard Massenburg, Motswadi Titus Maswabi, Manu Raj Mathur, Colm McAlinden, Martin McKee, Hailemariam Abiy Alemu Meheretu, Ravi Mehrotra, Varshil Mehta, Toni Meier, Yohannes A. Melaku, Gebrekiros Gebremichael Meles, Hagazi Gebre Meles, Addisu Melese, Mulugeta Melku, Peter T. N. Memiah, Walter Mendoza, Ritesh G. Menezes, Shahin Merat, Tuomo J. Meretoja, Tomislav Mestrovic, Bartosz Miazgowski, Tomasz Miazgowski, Kebadnew Mulatu M. Mihretie, Ted R. Miller, Edward J. Mills, Seyed Mostafa Mir, Hamed Mirzaei, Hamid Reza Mirzaei, Rashmi Mishra, Babak Moazen, Dara K. Mohammad, Karzan Abdulmuhsin Mohammad, Yousef Mohammad, Aso Mohammad Darwesh, Abolfazl Mohammadbeigi, Hiwa Mohammadi, Moslem Mohammadi, Mahdi Mohammadian, Abdollah Mohammadian-Hafshejani, Milad Mohammadoo-Khorasani, Reza Mohammadpourhodki, Ammas Siraj Mohammed, Jemal Abdu Mohammed, Shafiu Mohammed, Farnam Mohebi, Ali H. Mokdad, Lorenzo Monasta, Yoshan Moodley, Mahmood Moosazadeh, Maryam Moossavi, Ghobad Moradi, Mohammad Moradi-Joo, Maziar Moradi-Lakeh, Farhad Moradpour, Lidia Morawska, Joana Morgado-da-Costa, Naho Morisaki, Shane Douglas Morrison, Abbas Mosapour, Seyyed Meysam Mousavi, Achenef Asmamaw Muche, Oumer Sada S. Muhammed, Jonah Musa, Ashraf R. Nabhan, Mehdi Naderi, Ahamarshan Jayaraman Nagarajan, Gabriele Nagel, Azin Nahvijou, Gurudatta Naik, Farid Najafi, Luigi Naldi, Hae Sung Nam, Naser Nasiri, Javad Nazari, Ionut Negoi, Subas Neupane, Polly A. Newcomb, Haruna Asura Nggada, Josephine W. Ngunjiri, Cuong Tat Nguyen, Leila Nikniaz, Dina Nur Anggraini Ningrum, Yirga Legesse Nirayo, Molly R. Nixon, Chukwudi A. Nnaji, Marzieh Nojomi, Shirin Nosratnejad, Malihe Nourollahpour Shiadeh, Mohammed Suleiman Obsa, Richard Ofori-Asenso, Felix Akpojene Ogbo, In-Hwan Oh, Andrew T. Olagunju, Tinuke O. Olagunju, Mojisola Morenike Oluwasanu, Abidemi E. Omonisi, Obinna E. Onwujekwe, Anu Mary Oommen, Eyal Oren, Doris D. V. Ortega-Altamirano, Erika Ota, Stanislav S. Otstavnov, Mayowa Ojo Owolabi, Mahesh P A, Jagadish Rao Padubidri, Smita Pakhale, Amir H. Pakpour, Adrian Pana, Eun-Kee Park, Hadi Parsian, Tahereh Pashaei, Shanti Patel, Snehal T. Patil, Alyssa Pennini, David M. Pereira, Cristiano Piccinelli, Julian David Pillay, Majid Pirestani, Farhad Pishgar, Maarten J. Postma, Hadi Pourjafar, Farshad Pourmalek, Akram Pourshams, Swayam Prakash, Narayan Prasad, Mostafa Qorbani, Mohammad Rabiee, Navid Rabiee, Amir Radfar, Alireza Rafiei, Fakher Rahim, Mahdi Rahimi, Muhammad Aziz Rahman, Fatemeh Rajati, Saleem M. Rana, Samira Raoofi, Goura Kishor Rath, David Laith Rawaf, Salman Rawaf, Robert C. Reiner, Andre M. N. Renzaho, Nima Rezaei, Aziz Rezapour, Ana Isabel Ribeiro, Daniela Ribeiro, Luca Ronfani, Elias Merdassa Roro, Gholamreza Roshandel, Ali Rostami, Ragy Safwat Saad, Parisa Sabbagh, Siamak Sabour, Basema Saddik, Saeid Safiri, Amirhossein Sahebkar, Mohammad Reza Salahshoor, Farkhonde Salehi, Hosni Salem, Marwa Rashad Salem, Hamideh Salimzadeh, Joshua A. Salomon, Abdallah M. Samy, Juan Sanabria, Milena M. Santric Milicevic, Benn Sartorius, Arash Sarveazad, Brijesh Sathian, Maheswar Satpathy, Miloje Savic, Monika Sawhney, Mehdi Sayyah, Ione J. C. Schneider, Ben Schöttker, Mario Sekerija, Sadaf G. Sepanlou, Masood Sepehrimanesh, Seyedmojtaba Seyedmousavi, Faramarz Shaahmadi, Hosein Shabaninejad, Mohammad Shahbaz, Masood Ali Shaikh, Amir Shamshirian, Morteza Shamsizadeh, Heidar Sharafi, Zeinab Sharafi, Mehdi Sharif, Ali Sharifi, Hamid Sharifi, Rajesh Sharma, Aziz Sheikh, Reza Shirkoohi, Sharvari Rahul Shukla, Si Si, Soraya Siabani, Diego Augusto Santos Silva, Dayane Gabriele Alves Silveira, Ambrish Singh, Jasvinder A. Singh, Solomon Sisay, Freddy Sitas, Eugène Sobngwi, Moslem Soofi, Joan B. Soriano, Vasiliki Stathopoulou, Mu’awiyyah Babale Sufiyan, Rafael Tabarés-Seisdedos, Takahiro Tabuchi, Ken Takahashi, Omid Reza Tamtaji, Mohammed Rasoul Tarawneh, Segen Gebremeskel Tassew, Parvaneh Taymoori, Arash Tehrani-Banihashemi, Mohamad-Hani Temsah, Omar Temsah, Berhe Etsay Tesfay, Fisaha Haile Tesfay, Manaye Yihune Teshale, Gizachew Assefa Tessema, Subash Thapa, Kenean Getaneh Tlaye, Roman Topor-Madry, Marcos Roberto Tovani-Palone, Eugenio Traini, Bach Xuan Tran, Khanh Bao Tran, Afewerki Gebremeskel Tsadik, Irfan Ullah, Olalekan A. Uthman, Marco Vacante, Maryam Vaezi, Patricia Varona Pérez, Yousef Veisani, Simone Vidale, Francesco S. Violante, Vasily Vlassov, Stein Emil Vollset, Theo Vos, Kia Vosoughi, Giang Thu Vu, Isidora S. Vujcic, Henry Wabinga, Tesfahun Mulatu Wachamo, Fasil Shiferaw Wagnew, Yasir Waheed, Fitsum Weldegebreal, Girmay Teklay Weldesamuel, Tissa Wijeratne, Dawit Zewdu Wondafrash, Tewodros Eshete Wonde, Adam Belay Wondmieneh, Hailemariam Mekonnen Workie, Rajaram Yadav, Abbas Yadegar, Ali Yadollahpour, Mehdi Yaseri, Vahid Yazdi-Feyzabadi, Alex Yeshaneh, Mohammed Ahmed Yimam, Ebrahim M. Yimer, Engida Yisma, Naohiro Yonemoto, Mustafa Z. Younis, Bahman Yousefi, Mahmoud Yousefifard, Chuanhua Yu, Erfan Zabeh, Vesna Zadnik, Telma Zahirian Moghadam, Zoubida Zaidi, Mohammad Zamani, Hamed Zandian, Alireza Zangeneh, Leila Zaki, Kazem Zendehdel, Zerihun Menlkalew Zenebe, Taye Abuhay Zewale, Arash Ziapour, Sanjay Zodpey, Christopher J. L. Murray

**Affiliations:** 1 Institute for Health Metrics and Evaluation, University of Washington, Seattle,; 2 Division of Hematology, University of Washington, Seattle; 3 Haramaya University, Harar, Ethiopia; 4 Department of Clinical Biochemistry, Babol University of Medical Sciences, Babol, Iran; 5 Iranian Center of Neurological Research, Tehran University of Medical Sciences, Tehran, Iran; 6 Department of Neurology, Cairo University, Cairo, Egypt; 7 Department of Oncology, University of Calgary, Calgary, Alberta, Canada; 8 Department of Oncology, Ain Shams University, Cairo, Egypt; 9 Department of Parasitology and Mycology, Jahrom University of Medical Sciences, Jahrom, Iran; 10 Research Center for Non-communicable Diseases, Jahrom University of Medical Sciences, Jahrom, Iran; 11 Department of Epidemiology, Arak University of Medical Sciences, Arak, Iran; 12 Multiple Sclerosis Research Center, Tehran, Iran; 13 Public Health Research Center, New York University Abu Dhabi, Abu Dhabi, United Arab Emirates; 14 School of Public Health, Addis Ababa University, Addis Ababa, Ethiopia; 15 Department of Public Health, Wachemo University, Hossana, Ethiopia; 16 Clinical Pharmacy Unit, Mekelle University, Mekelle, Ethiopia; 17 Department of Healthcare Policy and Research, Weill Cornell Medical College in Qatar, Doha, Qatar; 18 Department of Sociology, Olabisi Onabanjo University, Ago Iwoye, Nigeria; 19 Social Behavioral Research Branch, National Institutes of Health, Bethesda, Maryland; 20 Cancer Prevention and Control Program, Georgetown University, Washington, DC; 21 Endocrinology and Metabolism Research Institute, Tehran University of Medical Sciences, Tehran, Iran; 22 Zabol University of Medical Sciences, Zabol, Iran; 23 Department of Epidemiology and Biostatistics, Qom University of Medical Sciences, Qom, Iran; 24 Department of Health, Directorate for Health Information and Research, Pieta, Malta; 25 Public Health Foundation of India, Gurugram, India; 26 Vital Strategies, Gurugram, India; 27 Knowledge Utilization Research Center, Tehran University of Medical Sciences, Tehran, Iran; 28 Department of Pharmacology and Toxicology, Tabriz University of Medical Sciences, Tabriz, Iran; 29 Department of Parasitology and Mycology, Tabriz University of Medical Sciences, Tabriz, Iran; 30 Department of Epidemiology, Jimma University, Jimma, Ethiopia; 31 Cancer Research Center, Shahid Beheshti University of Medical Sciences, Tehran, Iran; 32 Department of Population Health Sciences, Duke University, Durham, North Carolina; 33 Duke Global Health Institute, Duke University, Durham, North Carolina; 34 John T. Milliken Department of Internal Medicine, Washington University in St. Louis, St Louis, Missouri; 35 Clinical Epidemiology Center, VA Saint Louis Health Care System, Department of Veterans Affairs, St Louis, Missouri; 36 Department of Family and Community Medicine, Imam Abdulrahman Bin Faisal University, Dammam, Saudi Arabia; 37 Department of Family Medicine and Community Health, Case Western Reserve University, Cleveland, Ohio; 38 Evidence-Based Practice Research Center, Mayo Clinic Foundation for Medical Education and Research, Rochester, Minnesota; 39 School of Health Sciences, Madda Walabu University, Bale Goba, Ethiopia; 40 Department of Health Sciences, Woldia University, Woldia, Ethiopia; 41 Department of Microbiology, Immunology, and Parasitology, Addis Ababa University, Addis Ababa, Ethiopia; 42 Institute of Public Health, University of Gondar, Gondar, Ethiopia; 43 Research School of Population Health, Australian National University, Canberra, Australian Capitol Territory, Australia; 44 Department of Health Care Management and Economics, Urmia University of Medical Science, Urmia, Iran; 45 Health Management and Economics Research Center, Iran University of Medical Sciences, Tehran, Iran; 46 Department of Health Economics, Iran University of Medical Sciences, Tehran, Iran; 47 Department of Health Policy and Management, Kuwait University, Safat, Kuwait; 48 International Centre for Casemix and Clinical Coding, National University of Malaysia, Bandar Tun Razak, Malaysia; 49 Department of Social Medicine, Behbahan Faculty of Medical Sciences, Behbahan, Iran; 50 Department of Medicine, King Saud University, Riyadh, Saudi Arabia; 51 Department of Gastroenterology and Hepatology, McGill University, Montreal, Québec, Canada; 52 Department of Oral and Maxillofacial Surgery, University Hospital Knappschaftskrankenhaus Bochum, Bochum, Germany; 53 College of Medicine and Health Sciences, United Arab Emirates University, Al-Ain, United Arab Emirates; 54 Research Group in Health Economics, Universidad de Cartagena, Cartagena, Colombia; 55 Research Group in Hospital Management and Health Policies, Universidad de la Costa, Barranquilla, Colombia; 56 Department of Urology, Tehran University of Medical Sciences, Tehran, Iran; 57 Department of Health Services Management, Arak University of Medical Sciences, Arak, Iran; 58 Department of Internal Medicine, Komfo Anokye Teaching Hospital, Kumasi, Ghana; 59 Mansoura University, Mansoura, Egypt; 60 Carol Davila University of Medicine and Pharmacy, Bucharest, Romania; 61 Social Determinants of Health Research Center, Rafsanjan University of Medical Sciences, Rafsanjan, Iran; 62 Research Center for Evidence Based Medicine, Health Management and Safety Promotion Research Institute, Tabriz University of Medical Sciences, Tabriz, Iran; 63 School of Public Health, Hasanuddin University, Makassar, Indonesia; 64 Department of Sociology and Social Work, Kwame Nkrumah University of Science and Technology, Kumasi, Ghana; 65 Center for International Health, Ludwig Maximilians University, Munich, Germany; 66 Department of Healthcare Management, Tabriz University of Medical Sciences, Tabriz, Iran; 67 Department of Health Education and Health Promotion, Tehran University of Medical Sciences, Tehran, Iran; 68 School of Health Sciences, Birmingham City University, Birmingham, England, United Kingdom; 69 School of Nursing and Midwifery, Addis Ababa University, Addis Ababa, Ethiopia; 70 Department of Community Health Sciences, University of Manitoba, Winnipeg, Manitoba, Canada; 71 Qom University of Medical Sciences, Qom, Iran; 72 Institute of Biomedical Science, Mekelle University, Mekelle, Ethiopia; 73 Department of Clinical Chemistry, University of Gondar, Gondar, Ethiopia; 74 Education Development Center, Mashhad University of Medical Sciences, Mashhad, Iran; 75 Non-communicable Diseases Research Center, Tehran University of Medical Sciences, Tehran, Iran; 76 College of Nursing, Aksum University, Aksum, Ethiopia; 77 Department of Medical Laboratory Science, Haramaya University, Harar, Ethiopia; 78 University Institute of Public Health, The University of Lahore, Lahore, Pakistan; 79 College ofPublic Health, University of Hail, Hail, Saudi Arabia; 80 School of Business, University of Leicester, Leicester, England, United Kingdom; 81 Center for Health Systems Research, National Institute of Public Health, Cuernavaca, Mexico; 82 Bénin Clinical Research Institute, Abomey-Calavi, Benin; 83 Contrôle des Maladies Infectieuses, Laboratory of Studies and Research-Action in Health, Porto Novo, Benin; 84 Indian Institute of Public Health, Gandhinagar, India; 85 Department of Nursing, Wolaita Sodo University, Sodo, Ethiopia; 86 The Judith Lumley Centre, La Trobe University, Melbourne, Victoria, Australia; 87 General Office for Research and Technological Transfer, Peruvian National Institute of Health, Lima, Peru; 88 Department of Family and Community Health, School of Public Health, University of Health and Allied Sciences, Ho, Ghana; 89 Department of Epidemiology, Biostatistics, and Occupational Health, McGill University, Montreal, Québec, Canada; 90 Public Health Department, Dilla University, Dilla, Ethiopia; 91 Preventive Medicine and Public Health Research Center, Iran University of Medical Sciences, Tehran, Iran; 92 School of Health Sciences, University of Management and Technology, Lahore, Pakistan; 93 Public Health Risk Sciences Division, Public Health Agency of Canada, Toronto, Ontario, Canada; 94 Department of Nutritional Sciences, University of Toronto, Toronto, Ontario, Canada; 95 Department of Chemistry, Sharif University of Technology, Tehran, Iran; 96 Department of Ophthalmology, University Hospital of Ioannina, Ioannina, Greece; 97 Institute of Molecular Biology & Biotechnology, Foundation for Research & Technology, Ioannina, Greece; 98 Department of Medical Microbiology, Haramaya University, Harar, Ethiopia; 99 School of Nursing and Allied Medicine, Iran University of Medical Sciences, Tehran, Iran; 100 Heidelberg Institute of Global Health, Faculty of Medicine and University Hospital, Heidelberg University, Heidelberg, Germany; 101 Harvard T. H. Chan School of Public Health, Harvard University, Boston, Massachusetts; 102 Doctor Evidence, Santa Monica, California; 103 Social Determinants of Health Research Center, Lorestan University of Medical Sciences, Khorramabad, Iran; 104 Lorestan University of Medical Sciences, Khorramabad, Iran; 105 Public Health Department, Mizan-Tepi University, Teppi, Ethiopia; 106 Department of Pharmacoepidemiology and Social Pharmacy, Mekelle University, Mekelle, Ethiopia; 107 AC Environments Foundation, Cuernavaca, Mexico; 108 Department of Pharmacy, Wollo University, Dessie, Ethiopia; 109 Division of Cardiothoracic Surgery, University of Washington, Seattle,; 110 Dental Institute, King’s College London, London, England, United Kingdom; 111 Emergency Hospital of Bucharest, Carol Davila University of Medicine and Pharmacy, Bucharest, Romania; 112 Department of Global Pediatric Medicine, St. Jude Children’s Research Hospital, Memphis, Tennessee; 113 Department of Biostatistics and Bioinformatics, National Institute of Biomedical Genomics, Kalyani, India; 114 Social Determinants of Health Research Center, Babol University of Medical Sciences, Babol, Iran; 115 National Centre for Epidemiology & Population Health, Australian National University, Canberra, Australian Capital Territory, Australia; 116 Department of Clinical Pharmacy and Pharmacology, University of Dhaka, Ramna, Bangladesh; 117 The UCL Centre for Global Health Economics, University College London, London, England, United Kingdom; 118 Fast-Track Implementation Department, United Nations Programme on HIV/AIDS, Gaborone, Botswana; 119 School of Pharmacy, Mekelle University, Mekelle, Ethiopia; 120 Department of Global Public Health and Primary Care, University of Bergen, Bergen, Norway; 121 Cancer Registry of Norway, Oslo, Norway; 122 Department of Radiation Medicine, Oregon Health and Science University, Portland; 123 Department of Pediatrics, The University of Texas, Houston; 124 Department of Public Health and Epidemiology, Bahir Dar University, Bahir Dar, Ethiopia; 125 School of Pharmacy, College of Medicine and Health Science, Haramaya University, Harar, Ethiopia; 126 Department of Clinical and Molecular Biomedicine, University of Catania, Catania, Italy; 127 Department of Oncology, Mario Negri Institute for Pharmacological Research, Milan, Italy; 128 Ministry of Public Health, Beirut, Lebanon; 129 Division of Clinical Epidemiology and Aging Research, German Cancer Research Center, Heidelberg, Germany; 130 Department of Dermatology, Mayo Clinic, Rochester, Minnesota; 131 Biomedical Technologies, Bauman Moscow State Technical University, Moscow, Russia; 132 Department of Epidemiology and Evidence-Based Medicine, I. M. Sechenov First Moscow State Medical University, Moscow, Russia; 133 Public Health Department, Federal University of Juiz de Fora, Juiz de Fora, Brazil; 134 School of Population and Public Health, University of British Columbia, Vancouver, British Columbia, Canada; 135 Al Shifa School of Public Health, Al Shifa Trust Eye Hospital, Rawalpindi, Pakistan; 136 Institute for Cancer Research, Prevention and Clinical Network, Florence, Italy; 137 Department of Medical Epidemiology and Biostatistics, Karolinska Institutet, Stockholm, Sweden; 138 Applied Molecular Biosciences Unit, University of Porto, Porto, Portugal; 139 Institute of Public Health, University of Porto, Porto, Portugal; 140 Department of Epidemiology, Portuguese Oncology Institute of Porto, Porto, Portugal; 141 EpiUnit, Instituto de Saúde Pública, University of Cartagena, Cartagena, Colombia; 142 Department of Research and Health Technology Assessment, Gorgas Memorial Institute for Health Studies, Panama City, Panama; 143 National School of Public Health, Carlos III Health Institute, Madrid, Spain; 144 Clinical Epidemiology Program, Ottawa Hospital Research Institute, Ottawa, Ontario, Canada; 145 Mary MacKillop Institute for Health Research, Australian Catholic University, Melbourne, Victoria, Australia; 146 School of Public Health, The University of Hong Kong, Hong Kong, China; 147 College of Medicine, Alfaisal University, Riyadh, Saudi Arabia; 148 Department of Obstetrics and Gynecology, University of Gondar, Gondar, Ethiopia; 149 Department of Psychiatry, University of Toronto, Toronto, Ontario, Canada; 150 China Institute, University of Alberta, Edmonton, Alberta, Canada; 151 Department of Surgical Oncology, Tata Memorial Hospital, Mumbai, India; 152 Department of Obstetrics and Gynecology, People’s College of Medical Sciences and Research Centre, Bhopal, India; 153 Department of Biostatistics and Epidemiology, Babol University of Medical Sciences, Babol, Iran; 154 Epidemiology Research Center, Royan Institute, Tehran, Iran; 155 Clinical Governance, Gold Coast Health, Gold Coast, Queensland, Australia; 156 Department of Epidemiology, Human Genetics, and Environmental Sciences, The University of Texas, Houston; 157 Institute of Industrial Ecological Science, University of Occupational and Environmental Health, Kitakyushu, Japan; 158 Departments ofBiochemistry and Biomedical Science, Seoul National University Hospital, Seoul, South Korea; 159 Department of Pulmonary Medicine, Christian Medical College and Hospital, Vellore, India; 160 Faculty of Biology, Hanoi National University of Education, Hanoi, Vietnam; 161 Department of Cancer Immunology, Oslo University Hospital, Oslo, Norway; 162 Department of Dermatology, 2nd Clinic of Dermatology, Carol Davila University of Medicine and Pharmacy, Bucharest, Romania; 163 2nd Department of Dermatology, Colentina Clinical Hospital, Bucharest, Romania; 164 UCIBIO/REQUIMTE, Laboratory of Toxicology, Faculty of Pharmacy, University of Porto, Porto, Portugal; 165 Romagnolo Scientific Institute for the Study and Treatment of Tumors, Meldola, Italy; 166 Division of Plastic Surgery, University of Washington, Seattle; 167 Department of Epidemiology, A. C. Camargo Cancer Center, Sao Paulo, Brazil; 168 Department of Environmental Health, College of Public Health, Imam Abdulrahman Bin Faisal University, Dammam, Saudi Arabia; 169 Department of Dermatology, Case Western Reserve University, Cleveland, Ohio; 170 Pediatric Department, Faculty of Medicine, Tanta University, Tanta, Egypt; 171 Toxoplasmosis Research Center, Mazandaran University of Medical Sciences, Sari, Iran; 172 Institute for Research and Innovation in Health (i3S), University of Porto, Porto, Portugal; 173 Institute of Biomedical Engineering (INEB), University of Porto, Porto, Portugal; 174 Department of Medical Laboratory Sciences, College of Medicine and Health Sciences, Bahir Dar University, Bahir Dar, Ethiopia; 175 Nursing Department, Woldia University, Woldia, Ethiopia; 176 Department of Nursing, Jimma University, Jimma, Ethiopia; 177 School of Pharmacy, Aksum University, Aksum, Ethiopia; 178 Addis Ababa University, Addis Ababa, Ethiopia; 179 Center for Nutrition and Health Research, National Institute of Public Health, Cuernavaca, Mexico; 180 Department of Immunology, Birjand University of Medical Sciences, Birjand, Iran; 181 Department of Preventive Medicine, Addis Ababa University, Addis Ababa, Ethiopia; 182 Division of Cardiology, Atlanta Veterans Affairs Medical Center, Decatur, Georgia; 183 School of Nutrition, Food Science and Technology, Hawassa University, Hawassa, Ethiopia; 184 Department of Midwifery, Debre Berhan University, Debre Berhan, Ethiopia; 185 Faculty of Veterinary Medicine and Zootechnics, Autonomous University of Sinaloa, Culiacán Rosales, Mexico; 186 Disha Foundation, Gurgaon, India; 187 Department of Community Medicine, University of Peradeniya, Peradeniya, Sri Lanka; 188 Health Research Section, Nepal Health Research Council, Kathmandu, Nepal; 189 Center of Complexity Sciences, National Autonomous University of Mexico, Mexico City, Mexico; 190 Research and Technology, Ministry of Health and Medical Education, Tehran, Iran; 191 Department of Population and Health, University of Cape Coast, Cape Coast, Ghana; 192 Faculty of Social Sciences, Health Sciences, University of Tampere, Tampere, Finland; 193 Department of Clinical Surgery, University of Edinburgh, Edinburgh, Scotland, United Kingdom; 194 United Nations World Food Programme, New Delhi, India; 195 Faculty of Medicine, University of Belgrade, Belgrade, Serbia; 196 College of Health Sciences, Wollega University, Nekemte, Ethiopia; 197 Mycobacteriology Research Center, Jimma University, Jimma, Ethiopia; 198 Liver and Pancreaticobiliary Disease Research Center, Tehran University of Medical Sciences, Tehran, Iran; 199 Department of Clinical Epidemiology and Biostatistics, University of Newcastle, Newcastle, New South Wales, Australia; 200 Department of Basic Sciences, Maragheh University of Medical Sciences, Maragheh, Iran; 201 Medical Research Institute, Alexandria University, Alexandria, Egypt; 202 Department of Clinical Pathology, Mansoura University, Mansoura, Egypt; 203 Department of Public Health Sciences, Karolinska Institutet, Stockholm, Sweden; 204 Department of Statistics, Debre Markos University, Debre Markos, Ethiopia; 205 Department of Community Medicine, Tripoli University, Tripoli, Libya; 206 Department of Health Information, World Health Organization, Tripoli, Libya; 207 Department of Neurology, University of Washington, Seattle; 208 Department of Surgery, Seattle Children’s Hospital, Seattle, Washington; 209 Endemic Medicine and Hepatogastroenterology Department, Cairo University, Cairo, Egypt; 210 Ophthalmic Epidemiology Research Center, Shahroud University of Medical Sciences, Shahroud, Iran; 211 Department of Midwifery, Wolkite University, Wolkite, Ethiopia; 212 Public Health Department, St Paul’s Hospital Millennium Medical College, Addis Ababa, Ethiopia; 213 Center of Communicable Disease Control, Ministry of Health and Medical Education, Tehran, Iran; 214 School of Public Health, Arak University of Medical Sciences, Arak, Iran; 215 Department of Non-communicable Diseases, Ministry of Public Health, Dubai, United Arab Emirates; 216 Shahid Beheshti University of Medical Sciences, Tehran, Iran; 217 Babol University of Medical Sciences, Babol, Iran; 218 Department of Community Nutrition, Tabriz University of Medical Sciences, Tabriz, Iran; 219 Department of Medical and Surgical Sciences, University of Bologna, Bologna, Italy; 220 Department of Public Health Nutrition, Bahir Dar University, Bahir Dar, Ethiopia; 221 Requimte/LAQV, University of Porto, Porto, Portugal; 222 Department of Health Education and Behavioral Sciences, Jimma University, Jimma, Ethiopia; 223 Jimma University, Jimma, Ethiopia; 224 Department of Psychiatry, Kaiser Permanente, Fontana, California; 225 School of Health Sciences, A.T. Still University, Mesa, Arizona; 226 School of Public Health Medicine, Bielefeld University, Bielefeld, Germany; 227 James Cancer Hospital, Ohio State University, Columbus, Ohio; 228 Department of Oncology, St. Jude Children’s Research Hospital, Memphis, Tennessee; 229 Abadan School of Medical Sciences, Abadan, Iran; 230 Department of Chemical Sciences, Faculty of Pharmacy, University of Porto, Porto, Portugal; 231 Gene Expression and Regulation Program, Cancer Institute, Philadelphia, Pennsylvania; 232 Department of Dermatology, Kobe University, Kobe, Japan; 233 Department of Otolaryngology–Head and Neck Surgery, University of Washington, Seattle; 234 Department of Environmental Health Science, Mario Negri Institute for Pharmacological Research, Milan, Italy; 235 Faculty of Medicine and Pharmacy of Fez, University Sidi Mohammed Ben Abdellah, Fez, Morocco; 236 Non-communicable Disease Department, Laboratory of Studies and Research-Action in Health, Porto Novo, Benin; 237 Department of Nursing, Wollega University, Nekemte, Ethiopia; 238 Department of Nursing, Mekelle University, Mekelle, Ethiopia; 239 Bahir Dar University, Bahir Dar, Ethiopia; 240 Haramaya University, Dire Dawa, Ethiopia; 241 School of Pharmacy, Ambo University, Ambo, Ethiopia; 242 Institute of Epidemiology, Biostatistics and Informatics, Martin Luther University Halle-Wittenberg, Halle, Germany; 243 Department of Biostatistics, Mekelle University, Mekelle, Ethiopia; 244 Medical Surgical Department, Tabriz University of Medical Sciences, Tabriz, Iran; 245 Department of Medicine, Massachusetts General Hospital, Boston; 246 Department of Health Services Management, School of Health Management and Information Sciences, Iran University of Medical Sciences, Tehran, Iran; 247 Physiology Department, Iran University of Medical Sciences, Tehran, Iran; 248 Medical Department, Islamic Azad University, Rasht, Iran; 249 Unit of Academic Primary Care, University of Warwick, Coventry, England, United Kingdom; 250 Department of Public Health Medicine, University of KwaZulu-Natal, Durban, South Africa; 251 University of KwaZulu-Natal, Durban, South Africa; 252 Department of Nursing, A.C.S. Medical College and Hospital, Aksum, Ethiopia; 253 Department of Surgery, Federal University of Minas Gerais, Belo Horizonte, Brazil; 254 Department of Biostatistics and Epidemiology, University of Oklahoma, Oklahoma City; 255 Department of Health and Social Affairs, Government of the Federated States of Micronesia, Palikir, Federated States of Micronesia; 256 Occupational and Environmental Epidemiology Section, Cancer Prevention and Research Institute, Florence, Italy; 257 Postgraduate Program in Epidemiology, Federal University of Rio Grande do Sul, Porto Alegre, Brazil; 258 School of Medicine, Boston University, Boston, Massachusetts; 259 Department of Public Health, Federal University of Juiz de Fora, Juiz de Fora, Brazil; 260 School of Dentistry, State University of Montes Claros, Montes Claros, Brazil; 261 Department of Epidemiology, Healis Sekhsaria Institute for Public Health, Mumbai, India; 262 West Virginia Bureau for Public Health, Charleston; 263 Department of Health Policy, Management & Leadership, West Virginia University, Morgantown; 264 University of Tampere, UKK Institute, Tampere, Finland; 265 Department of Pharmacology, Tehran University of Medical Sciences, Tehran, Iran; 266 Obesity Research Center, Shahid Beheshti University of Medical Sciences, Tehran, Iran; 267 Department of Radiology, Johns Hopkins University, Baltimore, Maryland; 268 Department of Family and Community Medicine, Arabian Gulf University, Manama, Bahrain; 269 School of Health and Environmental Studies, Hamdan Bin Mohammed Smart University, Dubai, United Arab Emirates; 270 Biomedical Research Networking Center for Mental Health Network, Madrid, Spain; 271 Research and Development Unit, San Juan de Dios Sanitary Park, Sant Boi de Llobregat, Spain; 272 School of Nutrition and Food Sciences, Tabriz University of Medical Sciences, Tabriz, Iran; 273 Department of Microbiology, Maragheh University of Medical Sciences, Maragheh, Iran; 274 Department of Microbiology, Tehran University of Medical Sciences, Tehran, Iran; 275 Unit of Epidemiology and Social Medicine, University Hospital Antwerp, Wilrijk, Belgium; 276 International Foundation for Dermatology, London, England, United Kingdom; 277 St John’s Institute of Dermatology, King’s College London, London, England, United Kingdom; 278 Department of Health Metrics Sciences, School of Medicine, University of Washington, Seattle; 279 Mizan-Tepi University, Teppi, Ethiopia; 280 Department of Statistics and Econometrics, Bucharest University of Economic Studies, Bucharest, Romania; 281 Department of Epidemiology, Adigrat University, Adigrat, Ethiopia; 282 Center of Excellence in Behavioral Medicine, Nguyen Tat Thanh University, Ho Chi Minh City, Vietnam; 283 The University of Texas at Austin, Austin; 284 Transdisciplinary Centre for Qualitative Methods, Manipal University, Manipal, India; 285 Department of Pulmonology, Yokohama City University, Kanazawa-ku, Yokohama, Japan; 286 National Human Genome Research Institute, National Institutes of Health, Bethesda, Maryland; 287 Department of Epidemiology and Population Health, Albert Einstein College of Medicine, Bronx, NY; 288 Department of Epidemiology and Biostatistics, Tehran University of Medical Sciences, Tehran, Iran; 289 Department of Computer Engineering, Science and Research Branch, Islamic Azad University, Tehran, Iran; 290 Department of Computer Science, University of Human Development, Sulaimaniyah, Iraq; 291 Department of General Surgery, Carol Davila University of Medicine and Pharmacy, Bucharest, Romania; 292 Department of Internal Medicine, Bucharest Emergency Hospital, Bucharest, Romania; 293 Faculty of Dentistry, Department of Legal Medicine and Bioethics, Carol Davila University of Medicine and Pharmacy, Bucharest, Romania; 294 Department of Clinical Legal Medicine, National Institute of Legal Medicine Mina Minovici, Bucharest, Romania; 295 Division of Information and Computing Technology, College of Science and Engineering, Hamad Bin Khalifa University, Doha, Qatar; 296 Qatar Foundation, Doha, Qatar; 297 Department of Medical Laboratory Science, Arba Minch University, Arba Minch, Ethiopia; 298 Center for Health Outcomes & Evaluation, Bucharest, Romania; 299 Department of Epidemiology, Faculty of Medical Sciences, University of Kragujevac, Kragujevac, Serbia; 300 Department of Epidemiology and Biostatistics, National Institute for Health Development, Tallinn, Estonia; 301 Research Institute for Endocrine Sciences, Shahid Beheshti University of Medical Sciences, Tehran, Iran; 302 Department of Surgery, Usmanu Danfodiyo University Teaching Hospital, Sokoto, Nigeria; 303 Institute for Physical Activity and Nutrition, Deakin University, Burwood, Victoria, Australia; 304 Sydney Medical School, University of Sydney, Sydney, New South Wales, Australia; 305 Surveillance and Health Services Research, American Cancer Society, Atlanta, Georgia; 306 Department of Psychosis, Babol Noshirvani University of Technology, Babol, Iran; 307 A.C.S. Medical College and Hospital, Isfahan, Iran; 308 Health Education and Health Promotion Department, Tabriz University of Medical Sciences, Tabriz, Iran; 309 Faculty of Medicine, Babol University of Medical Sciences, Babol, Iran; 310 School of Public Health, Shahid Beheshti University of Medical Sciences, Tehran, Iran; 311 Safety Promotion and Injury Prevention Research Center, Shahid Beheshti University of Medical Sciences, Tehran, Iran; 312 Medical Sciences Department, University of Kragujevac, Kragujevac, Serbia; 313 Newcastle University, Tyne, United Kingdom; 314 Department of Surgery, Virginia Commonwealth University, Richmond; 315 Department of Public Health, Yonsei University, Seoul, South Korea; 316 Harvard Medical School, Harvard University, Boston, Massachusetts; 317 Faculty of Nursing & Midwifery, Hamadan University of Medical Sciences, Hamadan, Iran; 318 Department of Community Medicine, Banaras Hindu University, Varanasi, India; 319 Department of Ophthalmology, Heidelberg University, Mannheim, Germany; 320 Beijing Institute of Ophthalmology, Beijing Tongren Hospital, Beijing, China; 321 School of Public Health and Community Medicine, University of New South Wales, Sydney, New South Wales, Australia; 322 NSW Health, Sydney, New South Wales, Australia; 323 Health Services Management Training Centre, Semmelweis University, Budapest, Hungary; 324 School of Health Sciences, Savitribai Phule Pune University, Pune, India; 325 Institute of Family Medicine and Public Health, University of Tartu, Tartu, Estonia; 326 Minimally Invasive Surgery Research Center, Iran University of Medical Sciences, Tehran, Iran; 327 Department of Biology, Morgan State University, Baltimore, Maryland; 328 Institute for Epidemiology and Social Medicine, University of Münster, Münster, Germany; 329 Immunogenetics Research Center, Mazandaran University of Medical Sciences, Sari, Iran; 330 Department of Neurology, Mazandaran University of Medical Sciences, Sari, Iran; 331 Cellular and Molecular Biology Research Center, Babol University of Medical Sciences, Babol, Iran; 332 Drug Applied Research Center, Tabriz University of Medical Sciences, Tabriz, Iran; 333 Hematology-Oncology and Stem Cell Transplantation Research Center, Tehran University of Medical Sciences, Tehran, Iran; 334 Hematologic Malignancies Research Center, Tehran University of Medical Sciences, Tehran, Iran; 335 Department of Pharmacology and Clinical Pharmacy, Addis Ababa University, Dessie, Ethiopia; 336 Department of Public Health, Amhara Public Health Institute, Bahir Dar, Ethiopia; 337 School of Health Care Administration, Oklahoma State University, Tulsa; 338 Health Care Delivery Sciences, University of Tulsa, Tulsa, Oklahoma; 339 ODeL Campus, University of Nairobi, Nairobi, Kenya; 340 Department of Dermatology, Wolaita Sodo University, Wolaita Sodo, Ethiopia; 341 Department of Public Health, Madda Walabu University, Goba, Ethiopia; 342 School of Public Health, Wolaita Sodo University, Wolaita Sodo, Ethiopia; 343 Department of Public Health and Community Medicine, Jordan University of Science and Technology, Ramtha, Jordan; 344 Epidemiology and Biostatistics Department, Health Services Academy, Islamabad, Pakistan; 345 Department of Medical Microbiology & Immunology, United Arab Emirates University, Al Ain, United Arab Emirates; 346 Division of Health Policy and Management, Seoul National University, Seoul, South Korea; 347 Faculty of Health and Wellbeing, Sheffield Hallam University, Sheffield, United Kingdom; 348 College of Arts and Sciences, Ohio University, Zanesville; 349 Internal Medicine and Gastroenterology Department, National Hepatology and Tropical Research Institute, Cairo, Egypt; 350 Academy of Medical Sciences, Tehran, Iran; 351 Department of Public Health, Mazandaran University of Medical Sciences, Sari, Iran; 352 Health Sciences Research Center, Mazandaran University of Medical Sciences, Sari, Iran; 353 Department of Epidemiology, Hamadan University of Medical Sciences, Hamadan, Iran; 354 Department of Public Health, Imam Muhammad Ibn Saud Islamic University, Riyadh, Saudi Arabia; 355 Department of Health Policy and Management, Johns Hopkins University, Baltimore, Maryland; 356 Student Research Committee, Baqiyatallah University of Medical Sciences, Tehran, Iran; 357 International Otorhinolaryngology Research Association, Tehran, Iran; 358 Department of Nutrition and Health Science, Ball State University, Muncie, Indiana; 359 Department of Public Health, Kermanshah University of Medical Sciences, Kermanshah, Iran; 360 Department of Health Sciences, Northeastern University, Boston, Massachusetts; 361 School of Medicine, Xiamen University Malaysia, Sepang, Malaysia; 362 Department of Health Management and Health Economics, Kristiania University College, Oslo, Norway; 363 Department of Health Services Policy & Management, University of South Carolina, Columbia; 364 Department of Nursing and Health Promotion, Oslo Metropolitan University, Oslo, Norway; 365 Department of Health Economics and Social Security, Jagiellonian University Medical College, Krakow, Poland; 366 Neurophysiology Research Center, Hamadan University of Medical Sciences, Hamadan, Iran; 367 Brain Engineering Research Center, Institute for Research in Fundamental Sciences, Tehran, Iran; 368 Networking Center for Mental Health Network, San Juan de Dios Sanitary Park, Sant Boi de Llobregat, Spain; 369 Catalan Institution for Research and Advanced Studies, Barcelona, Spain; 370 Department of Public Health, Yüksek Ihtisas University, Ankara, Turkey; 371 Department of Public Health, Hacettepe University, Ankara, Turkey; 372 Department of Psychology and Health Promotion, University of KwaZulu-Natal, Durban, South Africa; 373 Department of Medicine Brigham and Women’s Hospital, Harvard University, Boston, Massachusetts; 374 Department of Nursing, St. John of God Hospital, Duayaw Nkwanta, Ghana; 375 Clinical Medicine and Community Health, A.C.S. Medical College and Hospital, Milan, Italy; 376 Department of Internal Medicine, Postgraduate Institute of Medical Education and Research, Chandigarh, India; 377 Department of Epidemiology and Biostatistics, University of Gondar, Gondar, Ethiopia; 378 Department of Community and Family Medicine, Academy of Medical Science, Baghdad, Iraq; 379 Division of Cancer Epidemiology and Genetics, National Cancer Institute, Rockville, Maryland; 380 Department of Otorhinolaryngology–Head and Neck Surgery, Father Muller Medical College, Mangalore, India; 381 Institute of Clinical Physiology, Italian National Research Council, Pisa, Italy; 382 Barcelona Institute for Global Health, Barcelona, Spain; 383 Asbestos Diseases Research Institute, University of Sydney, Sydney, New South Wales, Australia; 384 Department of Public Health, Debre Markos University, Debre Markos, Ethiopia; 385 Department of Medical Statistics and Epidemiology, Sun Yat-sen University, Guangzhou, China; 386 Alliance for Improving Health Outcomes, Inc, Quezon City, Philippines; 387 Department of Dentistry, Radboud University, Nijmegen, Netherlands; 388 Section for Translational Health Economics, Heidelberg University Hospital, Heidelberg, Germany; 389 University of Melbourne, Melbourne, Queensland, Australia; 390 Department of General Surgery, Aintree University Hospital National Health Service Foundation Trust, Liverpool, England, United Kingdom; 391 Department of Surgery, University of Liverpool, Liverpool, England, United Kingdom; 392 Department of Pathology, College of Medicine, Imam Abdulrahman Bin Faisal University, Dammam, Saudi Arabia; 393 Proteomics and Metabolomics Unit, Suez Canal University, Cairo, Egypt; 394 Department of Physiology, Suez Canal University, Ismailia, Egypt; 395 Department of Cardiology, Damietta University, Damietta, Egypt; 396 Department of Primary Care and Public Health, Imperial College London, London, England, United Kingdom; 397 Department of Environmental Health, Tehran University of Medical Sciences, Tehran, Iran; 398 Environmental Health Research Center, Research Institute for Health Development, Kurdistan University of Medical Sciences, Sanandaj, Iran; 399 Digestive Diseases Research Institute, Tehran University of Medical Sciences, Tehran, Iran; 400 Non-communicable Diseases Research Center, Shiraz University of Medical Sciences, Shiraz, Iran; 401 Department of Plastic Surgery, Iran University of Medical Sciences, Tehran, Iran; 402 Department of Ophthalmology, Iran University of Medical Sciences, Tehran, Iran; 403 Department of Ophthalmology, University of Manitoba, Winnipeg, Manitoba, Canada; 404 Department of Health Education and Promotion, Iran University of Medical Sciences, Tehran, Iran; 405 School of Medicine and Surgery, University of Milan-Bicocca, Monza, Italy; 406 School of Nursing and Midwifery, Guilan University of Medical Sciences, Rasht, Iran; 407 Department of Epidemiology, Airlangga University, Surabaya, Indonesia; 408 Indonesian Public Health Association, Surabaya, Indonesia; 409 School of Public Health, University of Botswana, Gaborone, Botswana; 410 Department of Epidemiology and Public Health, University College London, London, England, United Kingdom; 411 Department of Ophthalmology, Hywel Dda University Health Board, Carmarthen, Wales, United Kingdom; 412 Department of Health Services Research and Policy, London School of Hygiene & Tropical Medicine, London, England, United Kingdom; 413 Department of Nursing, Debre Markos University, Debre Markos, Ethiopia; 414 School of Public Health, Bahir Dar University, Bahir Dar, Ethiopia; 415 Department of Preventive Oncology, National Institute of Cancer Prevention and Research, Noida, India; 416 Department of Internal Medicine, SevenHills Hospital, Mumbai, India; 417 Institute for Agricultural and Nutritional Sciences, Martin Luther University Halle-Wittenberg, Halle, Germany; 418 Innovation Office, Competence Cluster for Nutrition and Cardiovascular Health, Halle, Germany; 419 Adelaide Medical School, University of Adelaide, Adelaide, South Australia, Australia; 420 Department of Public Health and Epidemiology, Arba Minch University, Arba Minch, Ethiopia; 421 Mekelle University, Mekelle, Ethiopia; 422 Department of Medical Laboratory Science, Bahir Dar University, Bahir Dar, Ethiopia; 423 Department of Public Health, University of West Florida, Pensacola, Florida; 424 Peru Country Office, United Nations Population Fund, Lima, Peru; 425 Forensic Medicine Division, Department of Pathology, College of Medicine, Imam Abdulrahman Bin Faisal University, Dammam, Saudi Arabia; 426 Breast Surgery Unit, Helsinki University Hospital, Helsinki, Finland; 427 University of Helsinki, Helsinki, Finland; 428 Clinical Microbiology and Parasitology Unit, Dr. Zora Profozic Polyclinic, Zagreb, Croatia; 429 University Centre Varazdin, University North, Varazdin, Croatia; 430 Center for Innovation in Medical Education, Pomeranian Medical University, Szczecin, Poland; 431 Pomeranian Medical University, Szczecin, Poland; 432 Department of Hypertension, Pomeranian Medical University, Szczecin, Poland; 433 Department of Epidemiology, Bahir Dar University, Bahir Dar, Ethiopia; 434 Pacific Institute for Research and Evaluation, Calverton, Maryland; 435 School of Public Health, Curtin University, Perth, Western Australia, Australia; 436 Department of Health Research Methods, Evidence, and Impact, McMaster University, Hamilton, Ontario, Canada; 437 Golestan University of Medical Sciences, Golestan, Iran; 438 Research Center for Biochemistry and Nutrition in Metabolic Diseases, Kashan University of Medical Sciences, Kashan, Iran; 439 Department of Medical Immunology, Tehran University of Medical Sciences, Tehran, Iran; 440 Department of Oral Medicine, University of Washington, Seattle,; 441 Institute of Public Health, Heidelberg University, Heidelberg, Germany; 442 Institute of Addiction Research, Frankfurt University of Applied Sciences, Frankfurt, Germany; 443 Department of Biology, Salahaddin University, Erbil, Iraq; 444 Department of Medicine, Huddinge, Karolinska Institutet, Stockholm, Sweden; 445 ISHIK University, Erbil, Iraq; 446 Department of Internal Medicine, King Saud University, Riyadh, Saudi Arabia; 447 Department of Information Technology, University of Human Development, Sulaymaniyah, Iraq; 448 Department of Neurology, Kermanshah University of Medical Sciences, Kermanshah, Iran; 449 Department of Physiology and Pharmacology, Mazandaran University of Medical Sciences, Sari, Iran; 450 Department of Epidemiology and Biostatistics, Bushehr University of Medical Sciences, Bushehr, Iran; 451 Department of Epidemiology and Biostatistics, Shahrekord University of Medical Sciences, Shahrekord, Iran; 452 Department of Clinical Biochemistry, Tarbiat Modares University, Tehran, Iran; 453 Department of Nursing, Shahroud University of Medical Sciences, Shahroud, Iran; 454 Department of Public Health, Samara University, Samera, Ethiopia; 455 Health Systems and Policy Research Unit, Ahmadu Bello University, Zaria, Nigeria; 456 Iran National Institute of Health Research, Tehran University of Medical Sciences, Tehran, Iran; 457 Clinical Epidemiology and Public Health Research Unit, Burlo Garofolo Institute for Maternal and Child Health, Trieste, Italy; 458 Department of Molecular Medicine, Birjand University of Medical Sciences, Birjand, Iran; 459 Social Determinants of Health Research Center, Kurdistan University of Medical Sciences, Sanandaj, Iran; 460 Department of Epidemiology and Biostatistics, Kurdistan University of Medical Sciences, Sanandaj, Iran; 461 Department of Economics and Management Sciences for Health, Tehran University of Medical Sciences, Tehran, Iran; 462 International Laboratory for Air Quality and Health, Queensland University of Technology, Brisbane, Queensland, Australia; 463 Hospital de Santo António, Hospital Center of Porto, Porto, Portugal; 464 Department of Social Medicine, National Center for Child Health and Development, Setagaya, Japan; 465 Department of Surgery, University of Washington, Seattle,; 466 Department of Health Management and Economics, Tehran University of Medical Sciences, Tehran, Iran; 467 Department of Pharmacology and Clinical Pharmacy, Addis Ababa University, Addis Ababa, Ethiopia; 468 Department of Obstetrics and Gynecology, University of Jos, Jos, Nigeria; 469 Center for Global Health, Northwestern University, Chicago, Illinois; 470 Department of Obstetrics and Gynecology, Ain Shams University, Cairo, Egypt; 471 Knowledge Translation and Utilization, Egyptian Center for Evidence Based Medicine, Cairo, Egypt; 472 School of Paramedical Sciences, Kermanshah University of Medical Sciences, Kermanshah, Iran; 473 Department of Research and Analytics, Initiative for Financing Health and Human Development, Chennai, India; 474 Department of Research and Analytics, Bioinsilico Technologies, Chennai, India; 475 Institute of Epidemiology and Medical Biometry, Ulm University, Ulm, Germany; 476 Cancer Institute, Tehran University of Medical Sciences, Tehran, Iran; 477 O’NealComprehensive Cancer Center, University of Alabama at Birmingham; 478 Department of Epidemiology and Biostatistics, Kermanshah University of Medical Sciences, Kermanshah, Iran; 479 Department of Dermatology, San Bortolo Hospital, Vicenza, Italy; 480 GISED Study Center, Bergamo, Italy; 481 Department of Preventive Medicine and Public Health, Chungnam National University School of Medicine, Daejeon, South Korea; 482 Daejeon Regional Cancer Center, Chungnam National University Hospital, Daejeon, South Korea; 483 Department of Public Health, School of Public Health, Jiroft University of Medical Sciences, Jiroft, Iran; 484 Department of Pediatrics, Arak University of Medical Sciences, Arak, Iran; 485 Ministry of Health and Medical Education, Tehran, Iran; 486 Faculty of Health Sciences, University of Tampere, Tampere, Finland; 487 Public Health Sciences Division, Fred Hutchinson Cancer Research Center, Seattle, Washington; 488 Department of Epidemiology, University of Washington, Seattle,; 489 Department of Histopathology, University of Maiduguri Teaching Hospital, Maiduguri, Nigeria; 490 Department of Human Pathology, University of Maiduguri, Maiduguri, Nigeria; 491 Department of Biological Sciences, University of Embu, Embu, Kenya; 492 Institute for Global Health Innovations, Duy Tan University, Hanoi, Vietnam; 493 Tabriz Health Services Management Research Center, Tabriz University of Medical Sciences, Tabriz, Iran; 494 Department of Public Health Sciences, State University of Semarang, Semarang, Indonesia; 495 Graduate Institute of Biomedical Informatics, Taipei Medical University, Taipei City, Taiwan; 496 Cochrane South Africa, South African Medical Research Council, Cape Town, South Africa; 497 School of Public Health and Family Medicine, University of Cape Town, Cape Town, South Africa; 498 Department of Community and Family Medicine, Iran University of Medical Sciences, Tehran, Iran; 499 Department of Health Economics, Tabriz University of Medical Sciences, Tabriz, Iran; 500 Mazandaran University of Medical Sciences, Sari, Iran; 501 Department of Anesthesia, Wolaita Sodo University, Sodo, Ethiopia; 502 Centre of Cardiovascular Research and Education in Therapeutics, Monash University, Melbourne, Victoria, Australia; 503 Independent consultant, Accra, Ghana; 504 Translational Health Research Institute, Western Sydney University, Penrith, New South Wales, Australia; 505 Department of Preventive Medicine, Kyung Hee University, Dongdaemun-gu, South Korea; 506 Department of Psychiatry and Behavioural Neurosciences, McMaster University, Hamilton, Ontario, Canada; 507 Department of Psychiatry, University of Lagos, Lagos, Nigeria; 508 Department of Pathology and Molecular Medicine, McMaster University, Hamilton, Ontario, Canada; 509 Department of Health Promotion and Education, Faculty of Public Health, College of Medicine, University of Ibadan, Ibadan, Nigeria; 510 Department of Anatomic Pathology, Ekiti State University, Ado- Ekiti, Nigeria; 511 Department of Anatomic Pathology, Ekiti State University Teaching Hospital, Ado-Ekiti, Nigeria; 512 Department of Pharmacology and Therapeutics, University of Nigeria, Nsukka, Enugu, Nigeria; 513 Department of Community Health, Christian Medical College, Vellore, India; 514 Graduate School of Public Health, San Diego State University, San Diego, California; 515 Department of Global Health Nursing, St. Luke’s International University, Chuo-ku, Japan; 516 Analytical Center, Moscow Institute of Physics and Technology, Dolgoprudny, Russia; 517 Institute for Advanced Medical Research and Training, University of Ibadan, Ibadan, Nigeria; 518 Department of TB & Respiratory Medicine, Jagadguru Sri Shivarathreeswara University, Mysore, India; 519 Department of Forensic Medicine and Toxicology, Manipal University, Mangaluru, India; 520 Department of Medicine, Ottawa Hospital Research Institute, Ottawa, Ontario, Canada; 521 Department of Public Health, Qazvin University of Medical Sciences, Qazvin, Iran; 522 Department of Nursing, Jönköping University, Jönköping, Sweden; 523 Department of Medical Humanities and Social Medicine, Kosin University, Busan, South Korea; 524 Environmental Health Research Center, Research Institute for Health Development, Kurdistan University of Medical Sciences, Sanandaj, Iran; 525 Department of Medicine, Maimonides Medical Center, Brooklyn, New York; 526 Krishna Institute of Medical Sciences, Deemed University, Karad, India; 527 University of Cartagena, Cartagena, Colombia; 528 ReferenceCenter for Epidemiology and Cancer Prevention, CPO Piedmont, Torino, Italy; 529 Basic Medical Sciences Department, Durban University of Technology, Durban, South Africa; 530 Departments of Parasitology and Entomology, Faculty of Medical Sciences, Tarbiat Modares University, Tehran, Iran; 531 Uro-Oncology Research Center, Tehran University of Medical Sciences, Tehran, Iran; 532 University Medical Center Groningen, University of Groningen, Groningen, Netherlands; 533 Faculty of Economics and Business, University of Groningen, Groningen, Netherlands; 534 Department of Public Health, Maragheh University of Medical Sciences, Maragheh, Iran; 535 Department of Nutrition and Food Sciences, Maragheh University of Medical Sciences, Maragheh, Iran; 536 Department of Nephrology, Sanjay Gandhi Postgraduate Institute of Medical Sciences, Lucknow, India; 537 Non-communicable Diseases Research Center, Alborz University of Medical Sciences, Karaj, Iran; 538 Department of Biomedical Engineering, Amirkabir University of Technology, Tehran, Iran; 539 Divisions of Chemistry and Diseases, Advanced Technologies Research Group, Tehran, Iran; 540 A.T. Still University, Mesa, Arizona; 541 Department of Immunology, Mazandaran University of Medical Sciences, Sari, Iran; 542 Molecular and Cell Biology Research Center, Mazandaran University of Medical Sciences, Sari, Iran; 543 Thalassemia and Hemoglobinopathy Research Center, Ahvaz Jundishapur University of Medical Sciences, Ahvaz, Iran; 544 Austin Health Clinical School of Nursing, La Trobe University, Heidelberg, Victoria, Australia; 545 National Centre for Farmer Health, Deakin University, Waurn Ponds, Victoria, Australia; 546 Department of Health Education and Promotion, Kermanshah University of Medical Sciences, Kermanshah, Iran; 547 Department of Public Health, Contech School of Public Health, Lahore, Pakistan; 548 Public Health Department, University of Health Sciences, Lahore, Pakistan; 549 Department of Radiation Oncology, All India Institute of Medical Sciences, New Delhi, India; 550 WHO Collaborating Centre for Public Health Education and Training, Imperial College London, London, England, United Kingdom; 551 University College London Hospitals, London, England, United Kingdom; 552 Academic Public Health, Public Health England, London, England, United Kingdom; 553 School of Social Sciences and Psychology, Western Sydney University, Penrith, New South Wales, Australia; 554 Research Center for Immunodeficiencies, Children’s Medical Center, Tehran University of Medical Sciences, Tehran, Iran; 555 Network of Immunity in Infection, Malignancy, and Autoimmunity, Universal Scientific Education and Research Network, Tehran, Iran; 556 Health Management and Economics Research Center, Iran University of Medical Sciences, Tehran, Iran; 557 Epidemiology Research Unit, Institute of Public Health, University of Porto, Porto, Portugal; 558 Department of Applied Chemistry, Faculty of Pharmacy, University of Porto, Porto, Portugal; 559 Department of Public Health, Wollega University, Nekemte, Ethiopia; 560 Golestan Research Center of Gastroenterology and Hepatology, Golestan University of Medical Sciences, Gorgan, Iran; 561 Infectious Diseases and Tropical Medicine Research Center, Babol University of Medical Sciences, Babol, Iran; 562 Clinical Operations, Doctor Evidence, Santa Monica, California; 563 Department of Epidemiology, Shahid Beheshti University of Medical Sciences, Tehran, Iran; 564 Medical Department, University of Sharjah, Sharjah, United Arab Emirates; 565 Managerial Epidemiology Research Center, Maragheh University of Medical Sciences, Maragheh, Iran; 566 Biotechnology Research Center, Mashhad University of Medical Sciences, Mashhad, Iran; 567 Neurogenic Inflammation Research Center, Mashhad University of Medical Sciences, Mashhad, Iran; 568 A.C.S. Medical College and Hospital, Tehran, Iran; 569 Taleghani Hospital, Kermanshah University of Medical Sciences, Kermanshah, Iran; 570 Department of Urology, Cairo University, Cairo, Egypt; 571 Public Health and Community Medicine Department, Cairo University, Giza, Egypt; 572 Center for Health Policy and Center for Primary Care and Outcomes Research, Stanford University, Stanford, California; 573 Department of Entomology, Ain Shams University, Cairo, Egypt; 574 Department of Surgery, Marshall University, Huntington, West Virginia; 575 Departments of Nutrition and Preventive Medicine, Case Western Reserve University, Cleveland, Ohio; 576 School of Public Health and Health Management, University of Belgrade, Belgrade, Serbia; 577 Faculty of Infectious and Tropical Diseases, London School of Hygiene & Tropical Medicine, London, England, United Kingdom; 578 Colorectal Research Center, Iran University of Medical Sciences, Tehran, Iran; 579 Surgery Department, Hamad Medical Corporation, Doha, Qatar; 580 Faculty of Health & Social Sciences, Bournemouth University, Bournemouth, England, United Kingdom; 581 UGC Centre of Advanced Study in Psychology, Utkal University, Bhubaneswar, India; 582 Udyam-Global Association for Sustainable Development, Bhubaneswar, India; 583 GSK Biologicals, Wavre, Belgium; 584 Department of Public Health Sciences, University of North Carolina at Charlotte; 585 Education Development Center, Ahvaz Jundishapur University of Medical Sciences, Ahvaz, Iran; 586 School of Health Sciences, Federal University of Santa Catarina, Ararangua, Brazil; 587 Department of Medical Statistics, Epidemiology and Medical Informatics, University of Zagreb, Zagreb, Croatia; 588 Division of Epidemiology and Prevention of Chronic Noncommunicable Diseases, Croatian Institute of Public Health, Zagreb, Croatia; 589 Gastrointestinal and Liver Diseases Research Center, Guilan University of Medical Sciences, Rasht, Iran; 590 Center of Expertise in Microbiology, Tehran University of Medical Sciences, Tehran, Iran; 591 Invasive Fungi Research Center, Mazandaran University of Medical Sciences, Sari, Iran; 592 Department of Health Promotion and Education, Alborz University of Medical Sciences, Karaj, Iran; 593 Department of Health Policy, Iran University of Medical Sciences, Tehran, Iran; 594 Department of Epidemiology, Shahid Beheshti University of Medical Sciences, Tehran, Iran; 595 Independent consultant, Karachi, Pakistan; 596 Department of Medical Laboratory Sciences, Mazandaran University of Medical Sciences, Sari, Iran; 597 Chronic Diseases (Home Care) Research Center, Hamadan University of Medical Sciences, Hamadan, Iran; 598 Department of Molecular Hepatology, Middle East Liver Disease Center, Tehran, Iran; 599 Razi Herbal Medicines Research Center, Lorestan University of Medical Sciences, Khorramabad, Iran; 600 Department of Basic Sciences, Islamic Azad University, Sari, Iran; 601 Department of Laboratory Sciences, Islamic Azad University, Sari, Iran; 602 Department of Ophthalmology, Kerman University of Medical Sciences, Kerman, Iran; 603 HIV/STI Surveillance Research Center, Institute for Futures Studies in Health, Kerman University of Medical Sciences, Kerman, Iran; 604 University School of Management and Entrepreneurship, Delhi Technological University, New Delhi, India; 605 Usher Institute of Population Health Sciences and Informatics, University of Edinburgh, Edinburgh, Scotland, United Kingdom; 606 Division of General Internal Medicine and Primary Care, Harvard University, Boston, Massachusetts; 607 Cancer Biology Research Center, Tehran University of Medical Sciences, Tehran, Iran; 608 Symbiosis Institute of Health Sciences, Symbiosis International University, Pune, Maharashtra, India; 609 School of Public Health and Preventive Medicine, Monash University, Melbourne, Victoria, Australia; 610 Imam Ali Cardiovascular Research Center, Kermanshah University of Medical Sciences, Kermanshah, Iran; 611 Faculty of Health, University of Technology Sydney, Sydney, New South Wales, Australia; 612 School of Health Sciences, Federal University of Santa Catarina, Florianópolis, Brazil; 613 University of Brasília, Brasília, Brazil; 614 Department of the Health Industrial Complex and Innovation in Health, Ministry of Health, Brasília, Brazil; 615 Menzies Institute for Medical Research, University of Tasmania, Hobart, Tasmania, Australia; 616 Global Patient Outcome and Real World Evidence, Eli Lilly and Company, Indianapolis, Indiana; 617 Department of Medicine, University of Alabama at Birmingham; 618 Department of Epidemiology, University of Alabama at Birmingham; 619 Medical Department, German Leprosy and TB Relief Association, Addis Ababa, Ethiopia; 620 Sydney School of Public Health, University of Sydney, Sydney, New South Wales, Australia; 621 Department of Internal Medicine and Specialties, University of Yaoundé I, Yaoundé, Cameroon; 622 Department of Endocrinology and Diabetes, Central Hospital of Yaoundé, Yaoundé, Cameroon; 623 Social Development and Health Promotion Research Center, Kermanshah University of Medical Sciences, Kermanshah, Iran; 624 Hospital Universitario de la Princesa, Autonomous University of Madrid, Madrid, Spain; 625 Centro de Investigación en Red de Enfermedades Respiratorias, Institute of Health Carlos III, Madrid, Spain; 626 Department of Occupational Therapy, Athens University of Applied Sciences, Athens, Greece; 627 Department of Community Medicine, Ahmadu Bello University, Zaria, Nigeria; 628 Department of Medicine, University of Valencia, Valencia, Spain; 629 Biomedical Research Networking Center for Mental Health Network, Carlos III Health Institute, Madrid, Spain; 630 Cancer Control Center, Osaka International Cancer Institute, Osaka, Japan; 631 University of Sydney, Sydney, New South Wales, Australia; 632 Jordan University of Science and Technology, Ramtha, Jordan; 633 Department of Public Health, Kurdistan University of Medical Sciences, Sanandaj, Iran; 634 Department of Community Medicine, Iran University of Medical Sciences, Tehran, Iran; 635 Department of Pediatrics, King Saud University, Riyadh, Saudi Arabia; 636 Department of Public Health, Adigrat University, Adigrat, Ethiopia; 637 Southgate Institute for Health, Society and Equity, Flinders University, Adelaide, South Australia, Australia; 638 Department of Public Health, Arba Minch University, Arba Minch, Ethiopia; 639 School of Public Health, University of Adelaide, Adelaide, South Australia, Australia; 640 Department of Public Health, University of Southern Denmark, Odense, Denmark; 641 Faculty of Health Sciences, Jagiellonian University Medical College, Krakow, Poland; 642 The Agency for Health Technology Assessment and Tariff System, Warsaw, Poland; 643 Department of Pathology and Legal Medicine, University of São Paulo, Ribeirão Preto, Brazil; 644 Department of Health Economics, Hanoi Medical University, Hanoi, Vietnam; 645 Department of Molecular Medicine and Pathology, The University of Auckland, Auckland, New Zealand; 646 Department of Clinical Hematology and Toxicology, Military Medical University, Hanoi, Vietnam; 647 Gomal Center of Biochemistry and Biotechnology, Gomal University, Dera Ismail Khan, Pakistan; 648 Division of Health Sciences, University of Warwick, Coventry, England, United Kingdom; 649 Department of General Surgery and Medical-Surgical Specialties, University of Catania, Catania, Italy; 650 Women’s Reproductive Health Research Center, A.C.S. Medical College and Hospital, Tabriz, Iran; 651 Alzahra Teaching Hospital, Tabriz, Iran; 652 Department for International Development, Health Network of Cuba, Havana, Cuba; 653 Centre of Research in Environmental Epidemiology, Barcelona Institute for Global Health, Barcelona, Spain; 654 Psychosocial Injuries Research Center, Ilam University of Medical Sciences, Ilam, Iran; 655 Department of Neurology & Stroke Unit, Sant’Anna Hospital, Como, Italy; 656 Occupational Health Unit, Sant’Orsola Malpighi Hospital, Bologna, Italy; 657 Department of Health Care Administration and Economy, National Research University Higher School of Economics, Moscow, Russia; 658 Department of Gastroenterology and Hepatology, Johns Hopkins University, Baltimore, Maryland; 659 Department of Pathology, Makerere University, Kampala, Uganda; 660 Department of Public Health, Woldia University, Woldia, Ethiopia; 661 Foundation University Medical College, Foundation University, Rawalpindi, Pakistan; 662 Department of Psychology and Counselling, University of Melbourne, Melbourne, Victoria, Australia; 663 Department of Medicine, University of Melbourne, St Albans, Victoria, Australia; 664 Department of Pharmacology and Toxicology, Mekelle University, Mekelle, Ethiopia; 665 Department of Nursing, Wollo University, Dessie, Ethiopia; 666 Department of Nursing, Addis Ababa University, Addis Ababa, Ethiopia; 667 Department of Neonatal and Pediatric Health Nursing, Bahir Dar University, Bahirdar, Ethiopia; 668 Department of Population Studies, International Institute for Population Sciences, Mumbai, India; 669 Foodborne and Waterborne Diseases Research Center, Shahid Beheshti University of Medical Sciences, Tehran, Iran; 670 Department of Medical Physics, Ahvaz Jundishapur University of Medical Sciences, Ahvaz, Iran; 671 Ophthalmic Research Center, Shahid Beheshti University of Medical Sciences, Tehran, Iran; 672 Department of Health Management, Policy, and Economics, Kerman University of Medical Sciences, Kerman, Iran; 673 Health Services Management Research Center, Institute for Futures Studies in Health, Kerman University of Medical Sciences, Kerman, Iran; 674 Wolkite University, Wolkite, Ethiopia; 675 School of Allied Health Sciences, Addis Ababa University, Addis Ababa, Ethiopia; 676 Department of Neuropsychopharmacology, National Center of Neurology and Psychiatry, Tokyo, Japan; 677 Health Economics & Finance, Global Health, Jackson State University, Jackson, Mississippi; 678 Department of Public Health, Tsinghua University, Beijing, China; 679 Department of Clinical Biochemistry, Tabriz University of Medical Sciences, Tabriz, Iran; 680 Physiology Research Center, Iran University of Medical Sciences, Tehran, Iran; 681 Department of Epidemiology and Biostatistics, Wuhan University, Wuhan, China; 682 Global Health Institute, Wuhan University, Wuhan, China; 683 Department of Electrical Engineering, Institute for Research in Fundamental Sciences, Tehran, Iran; 684 Department of Electrical Engineering, Bioelectric Group, Sharif University of Technology, Tehran, Iran; 685 Epidemiology and Cancer Registry Sector, Institute of Oncology, Ljubljana, Slovenia; 686 Social Determinants of Health Research Center, Ardabil University of Medical Science, Ardabil, Iran; 687 Department of Epidemiology, University Hospital of Setif, Setif, Algeria; 688 Student Research Committee, Babol University of Medical Sciences, Babol, Iran; 689 Department of Parasitology, Tarbiat Modares University, Tehran, Iran; 690 Department of Midwifery, Mekelle University, Mekelle, Ethiopia; 691 Department of Epidemiology and Biostatistics, Bahir Dar University, Bahir Dar, Ethiopia; 692 Health Promotion Research Center, Iran University of Medical Sciences, Tehran, Iran

## Abstract

**Importance:**

Cancer and other noncommunicable diseases (NCDs) are now widely recognized as a threat to global development. The latest United Nations high-level meeting on NCDs reaffirmed this observation and also highlighted the slow progress in meeting the 2011 Political Declaration on the Prevention and Control of Noncommunicable Diseases and the third Sustainable Development Goal. Lack of situational analyses, priority setting, and budgeting have been identified as major obstacles in achieving these goals. All of these have in common that they require information on the local cancer epidemiology. The Global Burden of Disease (GBD) study is uniquely poised to provide these crucial data.

**Objective:**

To describe cancer burden for 29 cancer groups in 195 countries from 1990 through 2017 to provide data needed for cancer control planning.

**Evidence Review:**

We used the GBD study estimation methods to describe cancer incidence, mortality, years lived with disability, years of life lost, and disability-adjusted life-years (DALYs). Results are presented at the national level as well as by Socio-demographic Index (SDI), a composite indicator of income, educational attainment, and total fertility rate. We also analyzed the influence of the epidemiological vs the demographic transition on cancer incidence.

**Findings:**

In 2017, there were 24.5 million incident cancer cases worldwide (16.8 million without nonmelanoma skin cancer [NMSC]) and 9.6 million cancer deaths. The majority of cancer DALYs came from years of life lost (97%), and only 3% came from years lived with disability. The odds of developing cancer were the lowest in the low SDI quintile (1 in 7) and the highest in the high SDI quintile (1 in 2) for both sexes. In 2017, the most common incident cancers in men were NMSC (4.3 million incident cases); tracheal, bronchus, and lung (TBL) cancer (1.5 million incident cases); and prostate cancer (1.3 million incident cases). The most common causes of cancer deaths and DALYs for men were TBL cancer (1.3 million deaths and 28.4 million DALYs), liver cancer (572 000 deaths and 15.2 million DALYs), and stomach cancer (542 000 deaths and 12.2 million DALYs). For women in 2017, the most common incident cancers were NMSC (3.3 million incident cases), breast cancer (1.9 million incident cases), and colorectal cancer (819 000 incident cases). The leading causes of cancer deaths and DALYs for women were breast cancer (601 000 deaths and 17.4 million DALYs), TBL cancer (596 000 deaths and 12.6 million DALYs), and colorectal cancer (414 000 deaths and 8.3 million DALYs).

**Conclusions and Relevance:**

The national epidemiological profiles of cancer burden in the GBD study show large heterogeneities, which are a reflection of different exposures to risk factors, economic settings, lifestyles, and access to care and screening. The GBD study can be used by policy makers and other stakeholders to develop and improve national and local cancer control in order to achieve the global targets and improve equity in cancer care.

## Key Points

**Question:**

What is the cancer burden over time at the global and national levels, measured in incidence, mortality, years lived with disability, years of life lost, and disability-adjusted life-years (DALYs), and how does it compare with other diseases?

**Findings:**

Results of this systematic analysis show that in 2017 there were 24.5 million incident cases (16.8 million without nonmelanoma skin cancer), 9.6 million deaths, and 233.5 million DALYs due to cancer; between 2007 and 2017, incident cases increased by 33%, with the lowest increase in the most developed countries, and between 1990 and 2017 neoplasms increased among the top causes of DALYs from the sixth to the second place. Fifty-one percent of cancer cases occurred in countries of high Socio-demographic Index, but only 30% of cancer deaths and 24% of cancer DALYs.

**Meaning:**

To ensure sustainable global development, increased efforts are needed in cancer prevention and in ensuring universal access to cancer care.

## Teaser

This systematic analysis describes cancer burden for 29 cancer groups across 195 countries from 1990 through 2017 to provide data needed for cancer control planning.

## Introduction

Cancer is now widely recognized as a global problem that unfortunately lacks a global solution. The latest United Nations high-level meeting on noncommunicable diseases (NCDs) exemplified this conundrum.^1^ Despite global commitment to reducing the risk of and disability from NCDs, including cancer, implementation of known solutions is inadequate to reach the 2011 Political Declaration on the Prevention and Control of Noncommunicable Diseases^2,3^ (25% reduction in premature mortality from NCDs by 2025) and the third Sustainable Development Goal (by 2030 reduce by one-third premature mortality from NCDs through prevention and treatment, and promote mental health and well-being).^4^ To reduce cancer burden, identifying the scope of the problem and mapping out implementation of solutions is best done in National Cancer Control Plans (NCCPs). However, a recent review showed that only 29% of low-income countries had a NCCP, and even if NCCPs existed, cost, financing, monitoring, and expansion of information systems was often inadequate. Many highly effective prevention and treatment strategies exist for cancer. However, they are often very specific (eg, vaccination for human papillomavirus and hepatitis B virus for prevention of cervical and liver cancer, or tyrosine kinase inhibitors for cancers with targetable mutations). Effective NCCPs therefore require detailed knowledge about the local burden of cancer and associated risk factors. We herein present results from the Global Burden of Disease (GBD) 2017 study describing cancer incidence, mortality, years of life lost (YLLs), years lived with disability (YLDs), and disability-adjusted life-years (DALYs) for 195 countries from 1990 through 2017, which can inform cancer control through policy, resource allocation, and health system planning.

## Methods

Methods have remained similar to the GBD 2016 study.^5^ Detailed descriptions of the methods can be found in the GBD 2017 publications^6,7,8,9^ as well as in the eAppendix, eFigures, and eTables in the Supplement. For each GBD study, the entire time series is re-estimated. This study therefore supersedes prior GBD iterations. The GBD study is compliant with the Guidelines for Accurate and Transparent Health Estimates Reporting statement (eTable 1 in the Supplement). Compared with the prior GBD study (GBD 2016), the neoplasms category for GBD 2017 also includes benign and in situ neoplasms (*International Statistical Classification of Diseases and Related Health Problems, Tenth Revision *[*ICD-10*] codes D00-D49). Because disability associated with benign neoplasms is most often very small, we only estimated disability for the new cause: myelodysplastic, myeloproliferative, and other hematopoietic neoplasms. The terms *malignant neoplasms* or *cancer* in this article only include *ICD-10* codes C00 through C96. Other changes since GBD 2016 are the addition of new data sources (eTable 3 in the Supplement) for GBD 2017 and improvements in the way we estimated cancer survival by using the mortality-to-incidence ratio (MIR). In this study, estimates are presented for 29 cancer categories and 195 countries and territories. Estimates for benign neoplasms as well as selected subnational estimates are available online (https://vizhub.healthdata.org/gbd-compare/ and http://ghdx.healthdata.org/gbd-results-tool). All rates are reported per 100 000 person-years. The GBD world population standard was used for the calculation of age-standardized rates.^9^ We report 95% uncertainty intervals for all estimates.

### Estimation Framework

The GBD cancer estimation process starts with mortality. Mortality estimates are made based on vital registration system (83% of data), cancer registry (16% of data) (eTable 3 in the Supplement), and verbal autopsy data (1% of data) using an ensemble model approach.^9,10^ Predictive covariates used in the model can be found in the eAppendix (eTable 8 in the Supplement). Single-cause mortality estimates are scaled into the separately estimated all-cause estimate.^9^ To estimate cancer incidence, mortality estimates are divided by a separately estimated MIR for each cancer type, sex, 5-year age group, location, and year; additional information regarding incidence and MIR estimation can be found in the eAppendix and eFigure 2 in the Supplement. Data sources used for estimating MIRs are described in eTable 2 in the Supplement. MIRs allow for a uniform method to estimate incidence. Other cancer estimation frameworks^11,12^ have set a precedent for using MIRs for decades and have detailed its benefits, including greater representativeness, especially in settings that lack quality or complete population-based cancer registry systems. By determining incidence using mortality, we are able to account for uncaptured incident cases and, if mortality and incidence are determined correctly, estimating incidence based on MIRs should result in the similar results if using incidence directly. The correlation between survival data and the MIR is used to estimate 10-year cancer prevalence. Total prevalence is partitioned into 4 sequelae: (1) diagnosis/treatment, (2) remission, (3) metastatic/disseminated, and (4) terminal phase. Each sequela prevalence is multiplied by a disability weight to estimate YLDs. Lifetime prevalence of procedure-related disability is estimated for larynx, breast, colorectal, bladder, and prostate cancers. A standard life expectancy is used to estimate years of life lost (YLLs).^9^ DALYs are the sum of YLDs and YLLs. To determine the contribution of population aging, population growth, and change in age-specific rates on the change in incident cases between 2007 and 2017, we use hypothetical demographic scenarios holding 2 of these 3 components constant. Results are stratified by quintiles of Socio-demographic Index (SDI), which is a composite indicator including fertility, education, and income.^7^

## Results

### Global Incidence, Mortality, and DALYs

In 2017, there were 24.5 million (95% UI, 22.0-27.4 million) incident cancer cases worldwide and 9.6 million (95% UI, 9.4-9.7 million) cancer deaths (Table). Cancer caused 233.5 million (95% UI, 228.8-238.0 million) DALYs in 2017, of which 97% came from YLLs and 3% came from YLDs (eTable 15 and eFigure 4 in the Supplement). Globally, the odds of developing cancer during a lifetime (ages 0-79 years) were 1 in 3 for men and 1 in 4 for women (eTable 16 in the Supplement). These odds differ substantially among SDI quintiles, ranging from 1 in 7 at the lowest SDI quintile to 1 in 2 at the highest SDI quintile for both sexes. In 2017, skin; tracheal, bronchus, and lung (TBL); and prostate cancers were the most common incident cancers in men, accounting for 54% of all cancer cases. The most common causes of cancer deaths and DALYs for men were TBL, liver, and stomach cancers (Table). For women in 2017, the most common incident cancers were nonmelanoma skin cancer (NMSC), breast cancer, and colorectal cancer, accounting for 54% of all incident cases. The leading causes of cancer deaths and DALYs for women were breast, TBL, and colorectal cancers.

**Table.** 2017 Global Incidence and Deaths for All Cancers and 29 Cancer Groups^a^

Cancer Type^b^	Incident Cases, Thousands^c^			ASIR (per 100 000)		Deaths, Thousands			ASDR (per 100 000)	
	Total	Male	Female	Male	Female	Total	Male	Female	Male	Female
All malignant neoplasms	24 491 (22 041-27 441)	13 294 (11 932-15 035)	11 197 (10 129-12 450)	365 (327-415)	265 (240-295)	9556 (9396-9692)	5442 (5325-5554)	4114 (4016-4201)	151.5 (148.2-154.6)	96.9 (94.5-98.9)
Lip and oral cavity	390 (374-404)	239 (226-249)	151 (144-159)	6.2 (5.9-6.5)	3.6 (3.4-3.8)	194 (185-202)	125 (117-131)	69 (65-72)	3.3 (3.1-3.5)	1.6 (1.5-1.7)
Nasopharynx	110 (104-116)	81 (76-87)	29 (27-30)	2.0 (1.9-2.2)	0.7 (0.7-0.7)	70 (67-72)	51 (48-54)	19 (18-19)	1.3 (1.3-1.4)	0.4 (0.4-0.5)
Other pharynx	179 (160-189)	131 (114-141)	48 (45-51)	3.3 (2.9-3.6)	1.1 (1.1-1.2)	117 (102-124)	84 (70-91)	33 (31-36)	2.2 (1.8-2.4)	0.8 (0.7-0.8)
Esophageal	473 (459-485)	331 (319-342)	142 (135-148)	8.9 (8.6-9.2)	3.3 (3.2-3.5)	436 (425-448)	311 (300-321)	125 (120-130)	8.4 (8.1-8.7)	2.9 (2.8-3.1)
Stomach	1221 (1189-1255)	799 (771-830)	421 (408-434)	21.7 (21.0-22.6)	9.9 (9.6-10.2)	865 (848-885)	546 (531-564)	319 (310-328)	15.2 (14.8-15.7)	7.5 (7.3-7.7)
Colon and rectum	1833 (1792-1873)	1015 (977-1047)	819 (795-839)	28.0 (27.0-28.9)	19.2 (18.6-19.6)	896 (876-916)	482 (465-498)	414 (401-423)	13.8 (13.3-14.2)	9.6 (9.4-9.9)
Liver	953 (917-997)	690 (654-734)	264 (254-275)	17.9 (17.0-19.1)	6.2 (6.0-6.5)	819 (790-856)	572 (543-610)	247 (239-257)	15.1 (14.4-16.1)	5.8 (5.6-6.0)
Gallbladder and biliary tract	211 (186-225)	90 (77-100)	120 (104-131)	2.6 (2.2-2.9)	2.8 (2.4-3.1)	174 (154-185)	72 (60-79)	102 (89-110)	2.1 (1.8-2.3)	2.4 (2.1-2.6)
Pancreatic	448 (439-456)	232 (225-239)	215 (210-221)	6.4 (6.2-6.6)	5.0 (4.9-5.2)	441 (433-449)	226 (219-233)	215 (211-220)	6.3 (6.1-6.5)	5.0 (4.9-5.1)
Larynx	211 (206-216)	178 (174-183)	33 (32-34)	4.6 (4.5-4.7)	0.8 (0.7-0.8)	126 (123-130)	106 (103-109)	21 (20-22)	2.8 (2.7-2.9)	0.5 (0.5-0.5)
Tracheal, bronchus, and lung	2163 (2117-2213)	1468 (1424-1514)	695 (674-715)	39.9 (38.7-41.1)	16.3 (15.8-16.7)	1883 (1844-1923)	1287 (1250-1322)	596 (579-614)	35.4 (34.4-36.3)	13.9 (13.5-14.4)
Malignant skin melanoma	309 (238-366)	157 (91-194)	152 (113-207)	4.2 (2.4-5.1)	3.6 (2.7-5.0)	62 (48-70)	33 (20-39)	29 (22-36)	0.9 (0.6-1.1)	0.7 (0.5-0.9)
Nonmelanoma skin cancer	7664 (5251-10 570)	4350 (2974-6035)	3314 (2276-4558)	122.1 (83.9-170.3)	77.9 (53.6-107.0)	65 (63-66)	43 (41-45)	22 (21-22)	1.3 (1.2-1.3)	0.5 (0.5-0.5)
Breast	1961 (1891-2023)	23 (22-24)	1938 (1868-2000)	0.6 (0.6-0.6)	45.9 (44.2-47.4)	612 (589-641)	11 (10-11)	601 (579-630)	0.3 (0.3-0.3)	14.1 (13.6-14.8)
Cervical	601 (554-625)	NA	601 (554-625)	NA	14.5 (13.4-15.1)	260 (241-269)	NA	260 (241-269)	NA	6.1 (5.7-6.4)
Uterine	407 (397-418)	NA	407 (397-418)	NA	9.6 (9.3-9.8)	85 (83-87)	NA	85 (83-87)	NA	2.0 (1.9-2.0)
Ovarian	286 (278-295)	NA	286 (278-295)	NA	6.8 (6.6-7.1)	176 (171-181)	NA	176 (171-181)	NA	4.1 (4.0-4.3)
Prostate	1334 (1171-1698)	1334 (1171-1698)	NA	37.9 (33.0-48.0)	NA	416 (357-490)	416 (357-490)	NA	13.1 (11.2-15.3)	NA
Testicular	71 (69-74)	71 (69-74)	NA	1.8 (1.7-1.9)	NA	8 (7-8)	8 (7-8)	NA	0.2 (0.2-0.2)	NA
Kidney	393 (371-405)	241 (226-249)	152 (141-158)	6.4 (6.0-6.6)	3.7 (3.4-3.8)	139 (129-143)	90 (85-93)	49 (43-51)	2.5 (2.4-2.6)	1.2 (1.0-1.2)
Bladder	474 (462-492)	362 (350-380)	111 (108-115)	10.3 (10.0-10.8)	2.6 (2.5-2.7)	197 (192-206)	145 (140-154)	52 (50-53)	4.4 (4.2-4.7)	1.2 (1.2-1.2)
Brain and nervous system	405 (351-443)	221 (189-251)	184 (132-213)	5.8 (4.9-6.5)	4.6 (3.3-5.3)	247 (213-265)	140 (118-158)	107 (76-119)	3.7 (3.1-4.1)	2.6 (1.9-2.9)
Thyroid	255 (246-272)	76 (73-79)	179 (170-196)	1.9 (1.9-2.0)	4.3 (4.1-4.7)	41 (40-44)	17 (16-18)	24 (23-27)	0.5 (0.5-0.5)	0.6 (0.5-0.6)
Mesothelioma	35 (34-36)	25 (24-26)	10 (10-11)	0.7 (0.7-0.7)	0.2 (0.2-0.3)	30 (29-31)	22 (21-22)	8 (8-9)	0.6 (0.6-0.6)	0.2 (0.2-0.2)
Hodgkin lymphoma	101 (88-119)	61 (50-75)	40 (34-48)	1.6 (1.3-1.9)	1.0 (0.9-1.2)	33 (28-38)	21 (17-26)	12 (10-14)	0.5 (0.4-0.7)	0.3 (0.2-0.3)
Non-Hodgkin lymphoma	488 (479-497)	279 (271-286)	209 (203-214)	7.5 (7.3-7.7)	5.0 (4.9-5.1)	249 (243-253)	144 (140-148)	104 (102-107)	4.0 (3.9-4.1)	2.5 (2.4-2.6)
Multiple myeloma	153 (141-173)	82 (70-98)	70 (67-82)	2.3 (1.9-2.7)	1.6 (1.6-1.9)	107 (99-119)	55 (46-64)	52 (49-58)	1.6 (1.3-1.8)	1.2 (1.1-1.4)
Other	716 (656-740)	383 (340-401)	333 (303-353)	10.3 (9.1-10.8)	8.2 (7.5-8.7)	360 (331-371)	187 (167-194)	173 (156-182)	5.1 (4.6-5.3)	4.2 (3.8-4.4)
Leukemia										
Acute lymphoid	108 (91-117)	64 (54-71)	43 (34-49)	1.7 (1.5-1.9)	0.7 (0.6-0.8)	52 (46-57)	31 (27-34)	22 (18-24)	0.8 (0.7-0.9)	0.6 (0.5-0.6)
Chronic lymphoid	114 (108-121)	66 (62-72)	48 (44-52)	1.8 (1.7-2.0)	1.1 (1.0-1.2)	35 (34-37)	21 (20-22)	14 (13-15)	0.6 (0.6-0.7)	0.3 (0.3-0.4)
Acute myeloid	140 (127-147)	79 (69-84)	61 (54-67)	2.1 (1.9-2.3)	1.5 (1.3-1.7)	100 (91-105)	57 (51-61)	42 (38-46)	1.6 (1.4-1.7)	1.0 (0.9-1.1)
Chronic myeloid	40 (37-43)	23 (20-24)	17 (15-20)	0.6 (0.6-0.7)	0.4 (0.4-0.5)	24 (22-26)	13 (12-14)	11 (10-13)	0.4 (0.3-0.4)	0.3 (0.2-0.3)
Other	246 (212-267)	142 (121-157)	104 (85-113)	3.9 (3.3-4.3)	2.6 (2.1-2.9)	136 (121-147)	76 (65-84)	61 (51-65)	2.1 (1.8-2.3)	1.5 (1.2-1.6)

Abbreviations: ASDR, age-standardized death rate; ASIR, age-standardized incidence rate; NA, not applicable.

^a^ All data reported as number or rate (95% uncertainty interval).

^b^ Cancer groups are defined based on *International Classification of Diseases, Ninth Revision (ICD-9)* and *International Statistical Classification of Diseases and Related Health Problems, Tenth Revision (ICD-10)* codes and include all codes pertaining to malignant neoplasms (*ICD-9* 140-208 and *ICD-10* C00-C96) except for Kaposi sarcoma (C46). eTables 4 and 5 in the Supplement detail how the original *ICD* codes were mapped to the standardized Global Burden of Disease cause list.

^c^ Detailed results for incidence, mortality, and disability-adjusted life-years for the global level, by Socio-demographic Index quintile, region, and country can be accessed in eTables 14 and 18 in the Supplement, as well as at https://vizhub.healthdata.org/gbd-compare/.

**Figure 1.**
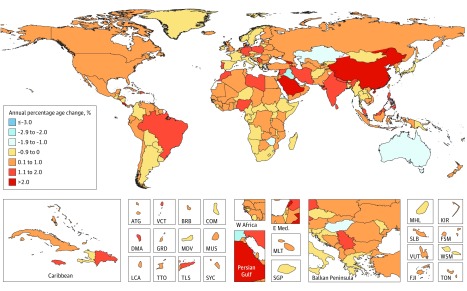
Average Annual Percentage Change in Age-Standardized Incidence Rate in Both Sexes for All Cancers From 2007 to 2017 ATG indicates Antigua and Barbuda; BRB, Barbados; COM, Comoros; DMA, Dominica; E Med., Eastern Mediterranean; FJI, Fiji; FSM, Federated States of Micronesia; GRD, Grenada; KIR, Kiribati; LCA, Saint Lucia; MDV, Maldives; MLT, Malta; MUS, Mauritius; MHL, Marshall Islands; SGP, Singapore; SLB, Solomon Islands; SYC, Seychelles; TLS, Timor-Leste; TON, Tonga; TTO, Trinidad and Tobago; VCT, Saint Vincent and the Grenadines; VUT, Vanuatu; W Africa, West Africa; WSM, Samoa.

**Figure 2.**
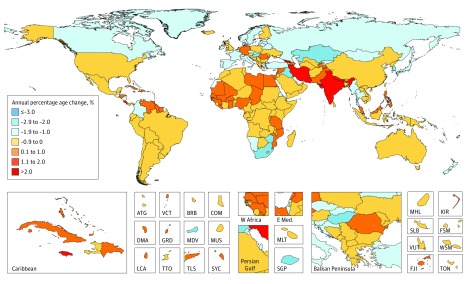
Average Annual Percentage Change in Age-Standardized Mortality Rate in Both Sexes for All Cancers From 2007 to 2017 ATG indicates Antigua and Barbuda; BRB, Barbados; COM, Comoros; DMA, Dominica; E Med., Eastern Mediterranean; FJI, Fiji; FSM, Federated States of Micronesia; GRD, Grenada; KIR, Kiribati; LCA, Saint Lucia; MDV, Maldives; MLT, Malta; MUS, Mauritius; MHL, Marshall Islands; SGP, Singapore; SLB, Solomon Islands; SYC, Seychelles; TLS, Timor-Leste; TON, Tonga; TTO, Trinidad and Tobago; VCT, Saint Vincent and the Grenadines; VUT, Vanuatu; W Africa, West Africa; WSM, Samoa.

Between 2007 and 2017, the average annual age-standardized incidence rates (ASIRs) for all cancers combined increased in 123 of 195 countries (Figure 1 and eFigure 5 in the Supplement). In contrast, the average annual age-standardized death rates for all cancers combined decreased within that timeframe in 145 of 195 countries (Figure 2 and eFigure 6 in the Supplement). Incident cases for both sexes combined increased in all SDI quintiles between 2007 and 2017 for nearly all cancers (eTable 14 in the Supplement). The largest increase in cancer incident cases between 2007 and 2017 occurred in middle SDI countries, with a 52% increase, of which changing age structure contributed 24%, population growth 10%, and changing age-specific incidence rates 18%. The drivers behind increasing cancer incidence differ substantially by SDI. Whereas in the lowest SDI quintile, population growth is the major contributor to the increase in total cancer incidence, in low-middle SDI countries aging and changes in incidence rates contribute equally (each 12%), and in high-middle and high SDI countries, increased incidence is mainly driven by population aging (eTable 14 in the Supplement).

### Global Top 10 Cancers in 2017

The global top 10 cancers were ranked by the highest number of incident cases, excluding “other malignant neoplasms.”

#### 1. Nonmelanoma Skin Cancer

**Figure 3.**
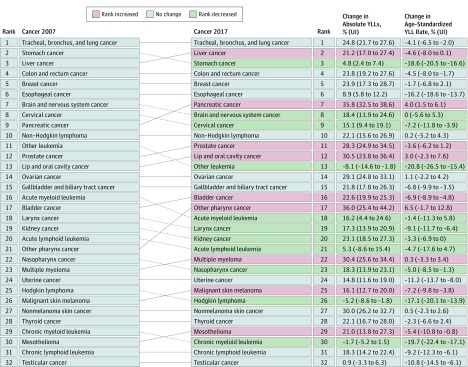
Cancers Ranked by Absolute Years of Life Lost (YLLs) Among Both Sexes Between 2007 and 2017^a^ UI indicates uncertainty interval. ^a^Excluding other cancer.

In 2017, there were 7.7 million (95% UI, 5.3-10.6 million) incident cases of NMSC, of which 5.9 million (95% UI, 3.7-8.7 million) were due to basal cell carcinoma and 1.8 million (95% UI, 1.1-2.6 million) due to squamous cell carcinoma. There were 65 000 (95% UI, 63 000-66 000) deaths due to NMSC (Table) and 1.3 million (95% UI, 1.3-1.4 million) DALYs, of which 97% came from YLLs (Figure 3) and 3% from YLDs (eTable 15 and eFigure 4 in the Supplement). Over a lifetime, the odds of developing NMSC were 1 in 7 for men and 1 in 10 for women globally. For men, the odds ranged from 1 in 71 in low SDI countries to 1 in 2 in high SDI countries, and for women from 1 in 104 in low SDI countries to 1 in 4 in high SDI countries (eTable 16 in the Supplement). An aging and growing population has led to a 33% (95% UI, 29%-36%) increase in NMSC cancer cases, from 5.8 million (95% UI, 4.1-7.8 million) in 2007 to 7.7 million (95% UI, 5.3-10.6 million) in 2017. The majority of this increase (20%) can be attributed to a change in the population age structure, and 13% can be attributed to population growth (eTable 14 and eFigure 11 in the Supplement).

#### 2. Tracheal, Bronchus, and Lung Cancer

In 2017, there were 2.2 million (95% UI, 2.1-2.2 million) incident cases of TBL cancer and 1.9 million (95% UI, 1.8-1.9 million) deaths. Tracheal, bronchus, and lung cancer caused 40.9 million (95% UI, 40.0-41.9 million) DALYs in 2017, of which 99% came from YLLs and 1% from YLDs (eTable 15 and eFigure 4 in the Supplement). Men were more likely to develop TBL cancer over a lifetime than women (1 in 17 men vs 1 in 43 women) (eTable 16 in the Supplement). The odds were the highest in high-middle SDI countries for men (1 in 13) and in high SDI countries for women (1 in 28). In low SDI countries, the odds were the lowest (1 in 45 for men and 1 in 142 for women). Tracheal, bronchus, and lung cancer was the leading cause of cancer in high-middle SDI countries (eFigure 5 in the Supplement). It was the most common cause of cancer deaths by absolute cases globally, as well as in all SDI quintiles (eFigure 6 in the Supplement). For men, TBL cancer was the most common incident cancer in 48 countries and the most common cause for cancer deaths in 110 countries (eFigures 7 and 9 in the Supplement). For women, TBL cancer was the most common incident cancer in Greenland and the most common cause of cancer deaths in 22 countries (eFigures 8 and 10 in the Supplement). Between 2007 and 2017, TBL cancer cases increased by 37% (95% UI, 33%-40%). Changing age structure contributed 19%, population growth 13%, and changes in age-specific incidence rates 5% (eTable 14 and eFigure 11 in the Supplement). The ASIRs between 1990 and 2017 show diverging results between men and women globally and in high SDI countries, with ASIRs decreasing in men but increasing in women (eFigure 12 in the Supplement). In high-middle SDI countries, ASIRs remained stable for men but increased for women, whereas rates increased for both sexes in middle SDI countries (eFigures 13 and 14 in the Supplement).

#### 3. Breast Cancer

Breast cancer was the third most common incident cancer overall with an estimated 2.0 million (95% UI, 1.9-2.0 million) incident cases in 2017. The majority occurred in women (1.9 million [95% UI, 1.9 -2.0 million]) (Table). Breast cancer was among the top 3 leading causes of cancer in all SDI quintiles except for the high and high-middle SDI quintiles, where it was the fourth most common cancer (eFigure 5 in the Supplement). It caused 601 000 (95% UI, 579 000-630 000) deaths in women and 11 000 (95% UI, 10 000-11 000) deaths in men, making it the fifth leading cause of cancer deaths for both sexes combined in 2017 globally (eFigure 6 in the Supplement). For women, breast cancer was the leading cause of cancer death in 2017 (Table). Breast cancer caused 17.7 million (95% UI, 16.9-18.7 million) DALYs for both sexes, of which 93% came from YLLs and 7% from YLDs (eTable 15 and eFigure 4 in the Supplement). Globally, 1 in 18 women developed breast cancer over a lifetime (eTable 16 in the Supplement). For women, the odds of developing breast cancer were the highest in high SDI countries (1 in 11), and the lowest in low SDI countries (1 in 38). For women, breast cancer was the most common cancer in 143 countries and the most common cause of cancer deaths in 112 countries (eFigures 8 and 10 in the Supplement). Overall, incident cases increased by 35% (95% UI, 30%-39%) because of a change in the population age structure (contributing 15%), population growth (contributing 13%), and an increase in age-specific incidence rates (contributing 7%) (eFigure 11 in the Supplement). Between 2007 and 2017, ASIRs for women decreased in high SDI countries but increased in the other SDI quintiles (eFigures 12-16 in the Supplement).

#### 4. Colon and Rectum Cancer

In 2017, there were 1.8 million (95% UI, 1.8-1.9 million) incident cases of colon and rectum cancer, and 896 000 (95% UI, 876 000-916 000) deaths (Table). Colon and rectum cancer caused 19.0 million (95% UI, 18.5-19.5 million) DALYs in 2017, of which 95% came from YLLs and 5% from YLDs (eTable 15 and eFigure 4 in the Supplement). The odds of developing colon and rectum cancer globally were higher for men than for women (1 in 26 for men vs 1 in 40 for women) (eTable 16 in the Supplement). The highest odds were in the high SDI quintile (1 in 15 for men and 1 in 25 for women) and the lowest in the low SDI quintile (1 in 81 for men and 1 in 98 for women). Between 2007 and 2017, incidence increased by 38% (95% UI, 34%-41%), from 1.3 million (95% UI, 1.3-1.3 million) to 1.8 million (95% UI, 1.8-1.9 million) cases (eTable 14 in the Supplement). Most of this increase can be explained by an aging and growing population (20% and 13%, respectively); however, even with the same population size and age structure, colorectal cancer cases would have increased by 5% between 2007 and 2017 owing to changing age-specific incidence rates. The ASIRs between 1990 and 2017 are similar for men and women at the global level and for all SDI quintiles (eFigures 12-16 in the Supplement).

#### 5. Prostate Cancer

In 2017, there were 1.3 million (95% UI, 1.2-1.7 million) incident cases of prostate cancer and 416 000 (95% UI, 357 000-490 000) deaths. Prostate cancer caused 7.1 million (95% UI, 6.1 million-8.4 million) DALYs globally in 2017, with 88% coming from YLLs and 12% from YLDs (eTable 15 and eFigure 4 in the Supplement). Globally, the odds of developing prostate cancer were 1 in 18, ranging from 1 in 52 for low SDI countries to 1 in 9 in high SDI countries (eTable 16 in the Supplement). In 2017, prostate cancer was the cancer with the highest incidence for men in 114 countries and the leading cause of cancer-related deaths for men in 56 countries (eFigures 7 and 9 in the Supplement). The increasing incidence rates, together with an aging and growing population, have led to a 42% (95% UI, 37%-52%) increase in prostate cancer cases since 2007 (940 000 [95% UI, 774 000-1.2 million] in 2007 and 1.3 million [95% UI, 1.2-1.7 million] in 2017). Twenty-one percent of this increase can be attributed to a change in the population age structure, 13% to a change in the population size, and 8% to a change in the age-specific incidence rates (eTable 14 and eFigure 11 in the Supplement).

#### 6. Stomach Cancer

In 2017, there were 1.2 million (95% UI, 1.2-1.3 million) incident cases of stomach cancer and 865 000 (95% UI, 848 000-885 000) deaths worldwide. Stomach cancer caused 19.1 million (95% UI, 18.7-19.6 million) DALYs in 2017, with 98% coming from YLLs and 2% coming from YLDs (eTable 15 and eFigure 4 in the Supplement). One in 33 men and 1 in 78 women developed stomach cancer over a lifetime. The highest odds for men and women were in high-middle SDI countries (1 in 21 and 1 in 57, respectively), and the lowest odds were for men in low SDI countries (1 in 78) and for women in low-middle SDI countries (1 in 104) (eTable 16 in the Supplement). Between 2007 and 2017, stomach cancer moved from the second leading cause of crude cancer YLLs to the third place with a 5% (95% UI, 2%-7%) increase in absolute YLLs (Figure 3). Overall, incidence between 2007 and 2017 increased by 25% (95% UI, 22%-29%), of which a change in the population age structure contributed 19%, population growth 13%, and falling age-specific rates −6% (eTable 14 and eFigure 11 in the Supplement). The ASIRs have dropped substantially since 1990 globally and for all SDI quintiles (eFigures 12-16 in the Supplement).

#### 7. Liver Cancer

In 2017, there were 953 000 (95% UI, 917 000-997 000) incident cases of liver cancer globally and 819 000 (95% UI, 790 000-856 000) deaths. Liver cancer caused 20.8 million (95% UI, 19.9-21.8 million) DALYs in 2017, with 99% coming from YLLs and 1% coming from YLDs (eTable 15 and eFigure 4 in the Supplement). Globally, liver cancer was more common in men, with 1 in 42 men developing liver cancer compared with 1 in 118 women. The highest odds of developing liver cancer were in high-middle SDI countries for men (1 in 31) and in middle SDI countries for women (1 in 94), whereas the lowest were seen in low SDI countries (1 in 98 men and 1 in 177 women) (eTable 16 in the Supplement). Population aging and population growth were the drivers of the increase from 705 000 (95% UI, 690 000-734 000) cases in 2007 to 953 000 (95% UI, 917 000-997 000) cases in 2017 (eTable 14 and eFigure 11 in the Supplement). Of the 35% increase in cases between 2007 and 2017, 17% was due to population aging, 13% due to population growth, and 6% due to an increase in age-specific incidence rates.

#### 8. Cervical Cancer

In 2017, 601 000 (95% UI, 554 000-625 000) women developed cervical cancer worldwide, and it caused 260 000 (95% UI, 241 000-269 000) deaths (Table). Cervical cancer caused 8.1 million (95% UI, 7.5-8.4 million) DALYs, with 96% coming from YLLs and 4% from YLDs (eTable 15 and eFigure 4 in the Supplement). Globally, 1 in 65 women developed cervical cancer during a lifetime (eTable 16 in the Supplement). The odds were the highest in low SDI countries (1 in 40) and the lowest in high SDI countries (1 in 106). In 2017, cervical cancer was the most common incident cancer for women in 50 countries (eFigure 8 in the Supplement) and the most common cause of cancer deaths in 39 countries (eFigure 10 in the Supplement). Between 2007 and 2017, incident cases increased by 19% (95% UI, 13%-23%) globally. Population growth contributed 13% and population aging 9%, while falling age-specific incidence rates offset this increase by −3% (eFigure 11 and eTable 14 in the Supplement). Deaths increased by 19% (95% UI, 13%-23%) between 2007 and 2017, and DALYs by 15% (95% UI, 10%-19%). The ASIRs decreased globally and for all SDI quintiles (eFigures 12-16 in the Supplement).

#### 9. Non-Hodgkin Lymphoma

In 2017, there were 488 000 (95% UI, 479 000-497 000) incident cases of non-Hodgkin lymphoma and 249 000 (95% UI, 243 000-253 000) deaths. Non-Hodgkin lymphoma caused 7.0 million (95% UI, 6.8-7.2 million) DALYs in 2017, with 97% coming from YLLs and 3% from YLDs (eTable 15 and eFigure 4 in the Supplement). Globally, 1 in 108 men and 1 in 162 women developed non-Hodgkin lymphoma over a lifetime. The highest odds were in high SDI countries (1 in 54 for men and 1 in 80 for women) and the lowest in low SDI countries (1 in 221 for men and 1 in 322 for women) (eTable 16 in the Supplement). Globally, incident cases between 2007 and 2017 increased by 39% (95% UI, 35%-42%), of which 15% was due to changing population age structure, 13% due to population growth, and 11% due to change in incidence rates (eTable 14 and eFigure 11 in the Supplement).

#### 10. Bladder Cancer

In 2017, there were 474 000 (95% UI, 462 000-492 000) incident cases of bladder cancer and 197 000 (95% UI, 192 000-206 000) deaths. Bladder cancer caused 3.6 million (95% UI, 3.5-3.8 million) DALYs in 2017, with 93% coming from YLLs and 7% from YLDs (eTable 15 and eFigure 4 in the Supplement). Globally, 1 in 74 men and 1 in 301 women developed bladder cancer over a lifetime. The highest odds were in high SDI countries (1 in 42 for men and 1 in 185 for women) and the lowest in low SDI countries (1 in 198 for men and 1 in 489 for women) (eTable 16 in the Supplement). Globally, incident cases between 2007 and 2017 increased by 32% (95% UI, 30%-35%), of which 20% was due to changing population age structure and 13% to population growth (eTable 14 and eFigure 11 in the Supplement).

#### Cancer in Comparison to Other Diseases

**Figure 4.**
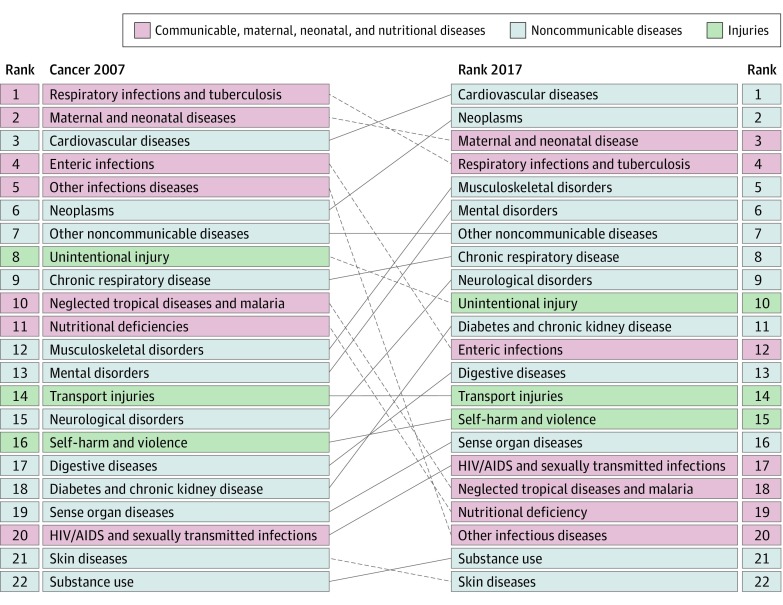
Change in the Absolute Number of Disability-Adjusted Life-Years (DALYs) Between 1990 and 2017 for Both Sexes at the Global Level for Global Burden of Disease Level 2 Causes^a^ The cause neoplasms includes all cancers as defined under *International Statistical Classification of Diseases and Related Health Problems, Tenth Revision (ICD-10) *causes C00 through C96, as well as myelodysplastic, myeloproliferative, and other hematopoietic neoplasms (*ICD-10* codes D45-D47.9). ^a^All diseases are grouped into 22 mutually exclusive and collectively exhaustive causes.

Within the 22 mutually exclusive and collectively exhaustive GBD level 2 disease categories (eTable 17 in the Supplement), neoplasms ranked last for incidence in 1990 and 2017 (eTable 18 in the Supplement). For prevalence, neoplasms ranked last in 1990 but surpassed enteric infections in 2017. The YLDs ranking for neoplasms also increased between 1990 and 2017 from the 21st to the 19th position. Mortality due to neoplasms remained at the second place between 1990 and 2017. The largest increase was seen for neoplasm YLLs and DALYs, which increased from the sixth place in 1990 to the second place in 2017 after cardiovascular diseases (Figure 4). The 4 causes with higher DALYs in 1990 that had been surpassed by neoplasms in 2017 are respiratory infections and tuberculosis, maternal and neonatal disorders, enteric infections, and other infections.

## Discussion

The GBD study results are updated on an annual basis. In this article we focus on changes over the past decade and present the most recent results from the GBD 2017 study using cancer registry, vital registration, and verbal autopsy data to estimate the burden of cancer for 195 countries and territories from 1990 through 2017.^13,14^ All results presented can also be found online at https://vizhub.healthdata.org/gbd-compare/ and http://ghdx.healthdata.org/gbd-results-tool. For this article, we also compare cancer burden with other diseases.

The GBD 2017 results show that there are 24.5 million incident cancer cases worldwide (16.8 million without NMSC) and 9.6 million deaths, which is similar to the latest GLOBOCAN estimates for 2018 that estimate 17.0 million cases (without NMSC) and 9.4 million deaths.^15^

The largest change in our estimates compared with the last iteration of the GBD study (GBD 2016) are the incidence estimates for NMSC, which have substantially increased. Despite being the most common incident cancer in many populations, cancer registry data to inform incidence estimates are often unreliable or nonexistent. For GBD 2017 we have therefore used Marketscan data for the United States, which has led to substantially higher estimates for NMSC.^16^

A key strength of the GBD study is the comparative health assessment. Our analysis shows how cancer has increased in importance as a global health problem. Although it ranked sixth in 1990 among the top causes for DALYs worldwide, it has risen to the second place in 2017 behind cardiovascular diseases. Cancer now occupies the second place in the ranking of global deaths, YLLs, and DALYs, and is among the top 2 leading causes of deaths, YLLs, and DALYs in the highest 3 SDI quintiles. This shift in disease burden owing to the demographic and epidemiological transitions has important implications on health policy: ensuring access to universal health coverage and protection against catastrophic health expenditure directly related to the cancer treatment, but also against the long-term costs associated with a cancer diagnosis for a household, has to be prioritized.^17^ Fifty percent of cancer cases occur in high SDI countries, but only 30% of cancer deaths, 25% of cancer DALYs, and 23% of cancer YLLs. To ensure sustainable global development, increased efforts are needed to reduce these health inequalities. Recognizing the strong interdependencies between socioeconomic status and health and the large contribution of cancer to the overall disease burden is a first step in making investments in cancer prevention and treatment a priority.^18^ Cervical cancer is likely the best example of inequalities in cancer with vast differences in burden by SDI. As a completely preventable cancer where cost-effective vaccination^3^ and screening approaches are available, cervical cancer has recently gained global attention through the World Health Organization’s call for elimination.^19^ Falling incidence rates in all SDI quintiles are encouraging, but countries with the least resources are still facing the largest burden because of lack of screening programs. Immunization against human papillomavirus, screening, and treatment of cervical cancer is therefore of utmost importance in all socioeconomic settings.

Deaths due to cancer contribute the majority of total health loss measured in DALYs, with disability contributing less than 12% for all cancers. As access to cancer care increases and treatments improve, cancer mortality decreases, but prevalence and disability in the survivor population increase, which is already the case in some high-income countries.^20^ The World Health Organization Global Action Plan for the Prevention and Control of NCDs and the United Nations Sustainable Development Goals focus on the reduction of premature mortality as the first goal. At the same time, infrastructure should be planned that can address the growing survivor population’s need.

### Limitations

The most important limitation for the GBD, as for other disease burden estimation, is the lack of data for many locations. A key GBD principle is to take advantage of all relevant data sources. This means for cancer estimation that incidence data from cancer registries, as well as mortality data from vital registration systems or verbal autopsies, is used to produce disease burden estimates. Despite these broad inclusion criteria for different types of data sources, certain locations have neither of these data sources available, and estimates rely either on predictive covariates or trends from neighboring locations. Also, diagnostic accuracy for cause of death data and ascertainment bias in cancer registries remains a limitation, which requires corrections for underregistration and redistribution algorithms for insufficiently specific or implausible diagnostic codes. Because of a lag in data availability, estimates for the most recent years are based on past time trends and covariates rather than data, which is reflected in larger uncertainty. Scarcity of reliable survival data worldwide requires the estimation of survival based on the mortality-to-incidence ratio, which is a surrogate for survival. Because in the majority of deaths due to Kaposi sarcoma the underlying cause of deaths is AIDS, deaths and incidence of Kaposi sarcoma are not estimated in the GBD. Also, common pediatric cancers are not estimated separately in the GBD and are estimated under the aggregated cause “other malignant neoplasms.”

## Conclusions

The national epidemiological profiles of cancer burden in the GBD study show large heterogeneities, which are a reflection of different exposures to risk factors, economic settings, lifestyles, and access to care. The GBD study can be used by policy makers and other stakeholders to develop and improve local cancer control in order to achieve the global targets and improve equity in cancer care.

## Notes

Supplement. eAppendix. eTables 1 through 18. eFigures 1 through 16.
