# Supplementary material for: Global, Regional, and National Cancer Incidence, Mortality, Years of Life Lost, Years Lived With Disability, and Disability-Adjusted Life-Years for 29 Cancer Groups, 1990 to 2017: A Systematic Analysis for the Global Burden of Disease Study
Source: JAMA Oncol. 2019 Sep 27;5(12):1749–68. doi: 10.1001/jamaoncol.2019.2996 (PMC6777271; doi:10.1001/jamaoncol.2019.2996)
Supplement: Supplement. — eAppendix. eTables 1 through 18. eFigures 1 through 16. [file jamaoncol-5-1749-s001.pdf]

© 2019 Global Burden of Disease Cancer Collaboration. JAMA Oncol.

Supplementary Online Content

Global Burden of Disease Cancer Collaboration. Global, Regional, and National Cancer Incidence, Mortality, Years of Life Lost, Years Lived With Disability, and Disability-Adjusted Life-Years for 29 Cancer Groups, 1990 to 2017: A Systematic Analysis for the Global Burden of Disease Study. *JAMA Oncol*. Published online September 27, 2019. doi:10.1001/jamaoncol.2019.2996

eAppendix.
eTables 1 through 18.
eFigures 1 through 16.

This supplementary material has been provided by the authors to give readers additional information about their work.

Supplementary Online Content

Global Burden of Disease Cancer Collaboration. Global, regional, and national cancer incidence, mortality, years of life lost, years lived with disability, and disability-adjusted life years for 29 cancer groups, 1990 to 2017: a systematic analysis for the Global Burden of Disease Study 2017.

Of note, updates to this appendix were only made if relevant, other parts remain the same as in the appendix to the GBD 2016 manuscript "Global Burden of Disease Cancer Collaboration, Fitzmaurice C, Akinyemiju TF, et al. Global, Regional, and National Cancer Incidence, Mortality, Years of Life Lost, Years Lived With Disability, and Disability-Adjusted Life-Years for 29 Cancer Groups, 1990 to 2016: A Systematic Analysis for the Global Burden of Disease Study. JAMA Oncol. June 2018. doi:10.1001/jamaoncol.2018.2706." Certain parts of this appendix have also been published in other GBD 2017 publications.[1–5]

eAppendix

TABLES 3

FIGURES 3

Additional method summaries for all GBD neoplasms except for NMSC, benign and in situ neoplasms, and myelodysplastic, myeloproliferative, and other hematological neoplasms 5

- Definition of indicator 5
- Data sources 5
  - Cancer incidence data sources 5
  - Mortality/incidence ratio data sources 5
  - Cancer mortality data sources 5
  - Bias of categories of input data 6
- Data analysis 6
  - Cancer registry data formatting 6
- Cause of death database formatting 9
- CODEm models 9
- Liver cancer etiology split models 9
- CoDCorrect 11
- Incidence estimation 11
- Prevalence and YLD estimation 11
- Probability of cancer 13

Additional method summaries for NMSC, benign and in situ neoplasms, and myelodysplastic, myeloproliferative, and other hematological neoplasms 14
Non-melanoma skin cancer (squamous and basal cell carcinoma) 14
Case definition 14
Non-melanoma skin cancer (NMSC) is defined as basal cell carcinoma and squamous cell carcinoma. NMSC does not include other types of skin cancer (e.g., melanoma, Merkel cell carcinoma). 14
Input data 14
We estimated squamous cell and basal cell skin cancer incidence by using cancer registry as well as primary literature and MarketScan data for incidence. Only cancer registries that were listed in CI5 VIII as registering squamous cell carcinoma or basal cell carcinoma, respectively, were included in the analysis. 14
Modeling strategy 14
Myelodysplastic, myeloproliferative, and other hematological neoplasms 14
Case definition 14
Input data 15
Modeling strategy 15
Benign and in situ intestinal neoplasms; benign and in situ cervical and uterine neoplasms; other benign and in situ neoplasms 15
Case definition 15
Input data 15
Modeling strategy 15
References 17
Supplementary Tables and Figures 21

TABLES

eTable 1: GATHER guidelines checklist ... 21
eTable 2: Sources for cancer incidence and mortality-to-incidence ratio data by country, year, and registry ... 23
eTable 3: Number of site-years for cancer mortality data by type ... 45
eTable 4: List of International Classification of Diseases (ICD) codes mapped to the Global Burden of Disease cause list for cancer incidence data ... 53
eTable 5: List of International Classification of Diseases (ICD) codes mapped to the Global Burden of Disease cause list for cancer mortality data ... 55
eTable 6: Undefined cancer code categories (ICD-10) and respective target codes for cancer registry incidence data ... 57
eTable 7: Socio-demographic Index groupings by geography, based on 2017 values ... 57
eTable 8: Covariates selected for CODEm for each GBD cancer group and expected direction of covariate ... 62
eTable 9: Comparison of GBD 2016 and GBD 2017 covariates used and level of covariates ... 93
eTable 10: Results for CODEm model testing ... 121
eTable 11: Percent change before and after CoDCorrect by cancer for all ages, both sexes combined, 2017 ... 129
eTable 12: Duration of four prevalence phases by cancer ... 131
eTable 13: Disability weights ... 134
eTable 14: Decomposition of trends in incidence globally, and by SDI quintile, both sexes, 2007 to 2017 ... 135
eTable 15: Contribution of YLDs and YLLs to DALYs by cancer, global, both sexes, 2017 ... 144
eTable 16: Probability of developing cancer within selected age intervals, global, and by SDI quintile, by sex, 2007-2017 in % (odds) ... 145
eTable 17: List of 22 level 2 causes in the GBD cause hierarchy ... 164
eTable 18: Global number of incidence, prevalence, YLDs, deaths, YLLs, DALYs for both sexes, 1990 and 2017 for all level 2 GBD causes ... 165

FIGURES

eFigure 1: Flowchart GBD cancer mortality, YLL estimation ... 178
eFigure 2: Flowchart GBD cancer incidence, prevalence, YLD estimation ... 179
eFigure 3: Socio-demographic Index quintiles ... 180
eFigure 4: Contribution of YLDs and YLLs to DALYs by cancer, global, both sexes, 2017 ... 181
eFigure 5: Cancer ranking by total incidence based on global level for developing and developed regions and all countries, both sexes, 2017 ... 182
eFigure 6: Cancer ranking by total mortality based on global level for developing and developed regions and all countries, both sexes, 2017 ... 200
eFigure 7: Top-ranked cancers by absolute incident cases for all ages in males, 2017 ... 201
eFigure 8: Top-ranked cancers by absolute incident cases for all ages in females, 2017 ... 202
eFigure 9: Top-ranked cancers by absolute deaths for all ages in males, 2017 ... 203
eFigure 10: Top-ranked cancers by absolute deaths for all ages in females, 2017 ... 204
eFigure 11: Global Decomposition of Changes in Cancer Incident Cases due to Population Growth, Population Aging, and Changes in Age-specific Incidence Rates, Both Sexes, 2007 to 2017. ... 205

eFigure 12: High SDI quintile decomposition of changes in cancer incident cases due to population growth, population ageing, and changes in age-specific incidence rates, both sexes, 2007 to 2017.... 206
eFigure 13: High-middle SDI quintile decomposition of changes in cancer incident cases due to population growth, population ageing, and changes in age-specific incidence rates, both sexes, 2007 to 2017........ 207
eFigure 14: Middle SDI quintile decomposition of changes in cancer incident cases due to population growth, population ageing, and changes in age-specific incidence rates, both sexes, 2007 to 2017.... 208
eFigure 15: Low-middle SDI quintile decomposition of changes in cancer incident cases due to population growth, population ageing, and changes in age-specific incidence rates, both sexes, 2007 to 2017.... 209

eFigure 16: Low SDI quintile decomposition of changes in cancer incident cases due to population growth, population ageing, and changes in age-specific incidence rates, both sexes, 2007 to 2017.... 210

© 2019 Global Burden of Disease Cancer Collaboration. JAMA Oncol.

Additional method summaries for all GBD neoplasms except for NMSC, benign and in situ neoplasms, and myelodysplastic, myeloproliferative, and other hematological neoplasms

Definition of indicator

The GBD cause list is organized in a hierarchy. Levels 1 and 2 represent general groupings. The broad group "neoplasms." which includes all malignant and benign neoplasms, is at Level 2 under the Level 1 group "Non-communicable diseases." Level 3 includes 29 cancer groups, and Level 4 includes 37 groups since in Level 4, leukemia, liver cancer, and non-melanoma skin cancer are further subdivided. In this publication, estimates for the GBD cancer groups, for both sexes, for the time from 1980 to 2017, and for the 5-year GBD age groups (0-5; 5-9; etc. until 95+) are presented for 195 countries or territories. All ICD9 codes pertaining to cancer (140-209) and ICD10 codes (C00-C96) except for Kaposi sarcoma (ICD10: C46) are included in the estimates for "malignant neoplasms," all ICD9 and ICD10 codes pertaining to neoplasms (ICD9 140-239, ICD10 C00-D49) are included in the estimates for "neoplasms." Of note, in the GBD Compare visualization (https://vizhub.healthdata.org/gbd-compare/), the Level 3 cause "other neoplasms" (ICD9 codes 210-239, ICD10 codes D00-D49), which includes the Level 4 causes "myelodysplastic, myeloproliferative, and other hematopoietic neoplasms," "benign and in situ cervical and uterine neoplasms," "benign and in situ intestinal neoplasms," and "other benign and in situ neoplasms") are not counted in the total incidence for the Level 2 "neoplasms" cause. However, they are counted for prevalence, mortality, years lived with disability (YLDs), years of life lost (YLLs), and disability-adjusted life years (DALYs). eTable 4 and eTable 5 list all ICD codes and their respective GBD cause. Countries and territories reported can be found in eTable 7.

Data sources

Cancer incidence data sources

Cancer incidence was sought from individual cancer registries or aggregated databases of cancer registry data like "Cancer Incidence In Five Continents" (CI5),"[6–15] EUREG,[16] or NORDCAN.[17] Data were excluded if they were not representative of the coverage population (e.g., hospital-based registries), if they did not cover all malignant neoplasms as defined in ICD9 (140-208) or ICD10 (C00-C96) (e.g., specialty cancer registry), if they did not include data for both sexes and all age groups, if the data were limited to years prior to 1980, or if the source did not provide details on the population covered. Preference was given to registries with national coverage over those with only local coverage, except those from countries where the GBD study provides subnational estimates. A list of the cancer registries included in our analysis and the years covered can be found in eTable 2. Additional metadata for each source are available in the online GBD citation tool, http://ghdx.healthdata.org/gbd-2017.

Mortality/incidence ratio data sources

Most cancer registries only report cancer incidence. However, if a cancer registry also reported cancer mortality, mortality data were also extracted from the source to be used in the mortality to incidence estimation. eTable 2 lists the registries used for the estimation of mortality-to-incidence ratios.

Cancer mortality data sources

A detailed description of the data sources and processing steps for the cause of death database can be found in the appendix to the GBD 2017 paper "Global, regional, and national age-sex-specific mortality

for 282 causes of death in 195 countries and territories, 1980–2017: a systematic analysis for the Global Burden of Disease Study 2017."[13]

Bias of categories of input data

Bias of the input data included for the COD database is described elsewhere.[13] Cancer registry data can be biased in multiple ways. A high proportion of ill-defined cancer cases in the registry data requires redistribution of these cases to other cancers, which introduces a potential for bias. Changes between coding systems can lead to artificial differences in disease estimates; however, we adjust for this bias by mapping the different coding systems to the GBD causes. Underreporting of cancers that require advanced diagnostic techniques (e.g., leukemia, brain, pancreatic, and liver cancer) can be an issue in cancer registries from low-income countries. On the other hand, misclassification of metastatic sites as primary cancer can lead to overestimation of cancer sites that are common sites for metastases like brain or liver. Since many cancer registries are located in urban areas, the representativeness of the registry for the general population can also be problematic. The accuracy of mortality data reported in cancer registries usually depends on the quality of the vital registration system. If the vital registration system is incomplete or of poor quality, the mortality-to-incidence ratio can be biased to lower ratios.

Data analysis

Flowcharts describing the conceptual overview of the data processing are available in eFigure 1 and eFigure 2.

Cancer registry data formatting

Cancer registry data went through multiple processing steps before integration with the COD database. First, the original data were transformed into standardized files, which included standardization of format, categorization, and registry names (#1 in eFigure 1).
Second, some cancer registries report individual codes as well as aggregated totals (e.g., C18, C19, and C20 are reported individually, but the aggregated group of C18–C20 (colorectal cancer) is also reported in the registry data). The data processing step, "subtotal recalculation" (#2 in flowchart), verifies these totals and subtracts the values of any individual codes from the aggregates.
In the third step (#3 in the flowchart), cancer registry incidence data and cancer registry mortality data are mapped to GBD causes. A different map is used for incidence and for mortality data because of the assumption that there are no deaths for certain cancers. One example is basal cell carcinoma of the skin. In the cancer registry incidence data, basal cell carcinoma is mapped to non-melanoma skin cancer (basal cell carcinoma). However, if basal cell skin cancer is recorded in the cancer registry mortality data, the deaths are instead mapped to non-melanoma skin cancer (squamous cell carcinoma) under the assumption that they were indeed misclassified squamous cell skin cancers. Other examples are benign or in situ neoplasms. Benign or in situ neoplasms found in the cancer registry incidence dataset were simply dropped from that dataset since cancer registries do not collect non-malignant neoplasms in a standardized way. The same neoplasms reported in a cancer registry mortality dataset were mapped to the respective invasive cancer (e.g., melanoma in situ in the cancer registry incidence dataset was dropped from the dataset; melanoma in situ in the cancer registry mortality dataset was mapped to melanoma). Mapping for incidence and mortality data can be found in eTable 4 and eTable 5.
In the fourth data processing step (#4 in the flowchart), cancer registry data were standardized to the GBD age groups. Age-specific incidence rates were generated age weights from administrative claims data as specified in appendix section 2.1.5 (James SL, Abate D, Abate KH, et al. Global, regional, and national incidence, prevalence, and years lived with disability for 354 diseases and injuries for 195

countries and territories, 1990–2017: a systematic analysis for the Global Burden of Disease Study 2017. The Lancet. 2018;392(10159):1789-1858. doi:10.1016/S0140-6736(18)32279-7),[14] while age-specific mortality rates were generated from the CoD data.[13] Age-specific weights were then generated by applying the age-specific rates to a given registry population that required age-splitting to produce the expected number of cases/deaths for that registry by age. The expected number of cases/deaths for each sex, age, and cancer were then normalized to 1, creating final, age-specific proportions. These proportions were then applied to the total number of cases/deaths by sex and cancer to get the age-specific number of cases/deaths.

In the rare case that the cancer registry only contained data for both sexes combined, the age-specific cases/deaths were split and reassigned to separate sexes using the same weights that are used for the age-splitting process. Starting from the expected number of deaths, proportions were generated by sex for each age (e.g., if for ages 15-19 years old there are 6 expected deaths for males and 4 expected deaths for females, then 60% of the combined-sex deaths for ages 15-19 years would be assigned to males and the remaining 40% would be assigned to females).

In the fifth step (#5 in the flowchart), data for cause entries that are aggregates of GBD causes were redistributed. Examples of these aggregated causes include some registries reporting ICD10 codes C00-C14 together as, "lip, oral cavity, and pharyngeal cancer." These groups were broken down into subcauses that could be mapped to single GBD causes. In this example, those include lip and oral cavity cancer (C00-C08), nasopharyngeal cancer (C11), cancer of other parts of the pharynx (C09-C10, C12-C13), and "Malignant neoplasm of other and ill-defined sites in the lip, oral cavity, and pharynx" (C14). To redistribute the data, weights were created using the same method employed in age-sex splitting (see step four above). For the undefined code (C14 in the example) an "average all cancer" weight was used, which was generated by adding all cases from SEER/NORDCAN/CI5 and dividing those by the combined population. Then, proportions were generated by subcause for each aggregate cause as in the sex-splitting example above (see step four). The total number of cases from the aggregated group (C00-C14) was recalculated for each subgroup and the undefined code (C14). C14 was then redistributed as a insufficiently specific code in step six. Distinct proportions were used for C46 (Kaposi sarcoma). C46 entries were redistributed as "other cancer" and HIV.

In the sixth step (#6 in the flowchart), unspecified codes ("garbage code") were redistributed. Redistribution of cancer registry incidence and mortality data mirrored the process of the redistribution used in the cause of death database and has not changed compared to GBD 2013.[19]

In the seventh step (#7 in the flowchart), duplicate or redundant sources were removed from the processed cancer registry dataset. Duplicate sources were present if, for example, the cancer registry was part of the CI5 database but we also had data from the registry directly. Redundancies occurred and were removed as described in "Inclusion and Exclusion Criteria," where more detailed data were available, or when national registry data could replace regionally representative data. From here, two parallel selection processes were run to generate input data for the MI models and to generate incidence for final mortality estimation. Higher priority was given to registry data from the most standardized source when creating the final incidence input, whereas for the MI model input, only sources that reported incidence and mortality were used.

In the eighth step (#8 in the flowchart), the processed incidence and mortality data from cancer registries were matched by cancer, age, sex, year, and location to generate MI ratios. These MI ratios were used as input for a three-step modeling approach using the general GBD ST-GPR[17] approach with the HAQ Index as a covariate in the linear step mixed effects model using a logit link function.[20]

$$\text{logit}\left(MI\ ratio_{c,a,s,t}\right) = \alpha + \beta_1 HAQI_{c,t} + \sum_{a}^{A} \beta_2 I_a + \beta_3 I_s + \epsilon_{c,a,s,t}$$

c: country, a: age group, t: time (years); s: sex
HAQI: Healthcare Access and Quality index
I: indicator variable
$\epsilon_{c,a,s,t}$: error term

This is different compared to GBD 2016, where we used the Socio-demographic Index (SDI) as a predictor. Predictions were made without the random effects. The ST-GPR model has three main hyper-parameters that control for smoothing across time, age, and geography. The time adjustment parameter (λ) was set to 0.07, which aims to borrow strength from neighboring time points (i.e., the exposure in this year is highly correlated with exposure in the previous year but less so further back in time). The age adjustment parameter ω was set to 1, which borrows strength from data in neighboring age groups. The space adjustment parameter ξ was set to 0.02. Zeta aims to borrow strength across the hierarchy of geographical locations.[18] For the amplitude parameter in the Gaussian process regression we used 1 and for the scale we used a value of 15.
The data cleaning has remained the same as in GBD 2016 where we excluded data based on the SDI quintile categorization. For each cancer, MI ratios from locations in SDI quintiles 1-4 (low to high-middle SDI) were dropped if they were below the median of MI ratios from locations in SDI quintile 5 (high SDI). We also dropped MI ratios from locations in SDI quintiles 1-4 if the MI ratios were above the third quartile + 1.5 * IQR (inter-quartile range). We dropped all MIR that were based on less than 25 cases to avoid noise due to small numbers except for mesothelioma and acute lymphoid leukemia, where we dropped MIR that were based on less than 10 cases because of lower data availability for these two cancers. We also aggregated incidence and mortality to the youngest five-year age bin where we had at least 50 data points to avoid MIR predictions in young age groups that were based on few data points. The MIR in the age-bin that was used to aggregate MIR was used to backfill the MIR for younger age groups.
Since MI ratios can be above 1, especially in older age groups and cancers with low cure rates, we used the 95th percentile of the cleaned dataset that only included MIR that were based on 50 or more cases to cap the MIR input data. This “upper cap” was used to allow MIR over 1 but to constrain the MIR to a maximum level. To run the logit model, the input data were divided by the upper caps and model predictions after ST-GPR was rescaled by multiplying them by the upper caps.
Upper caps used for GBD 2017 were the following:

**Age group**	**Maximum MIR**
0-4	0.56
5-9	0.71
10-14	0.84
15-19	0.86
20-24	0.65
25-29	0.59
30-34	0.63
35-39	0.73

 © 2019 Global Burden of Disease Cancer Collaboration. JAMA Oncol.

40-44	0.83
45-49	0.86
50-54	0.89
55-59	0.91
60-64	0.96
65-69	1.01
70-74	1.09
75-79	1.22
80-84	1.36
85-89	1.39
90-94	1.45
95+	1.87

To constrain the model at the lower end, we used the 5th percentile of the cancer-specific cleaned MIR input data to replace all model predictions with this lower cap.
Final MI ratios were matched with the cancer registry incidence dataset in the ninth step (#9 in the flowchart) to generate mortality estimates (Incidence * Mortality/Incidence = Mortality) (#10 in the flowchart). The final mortality estimates were then uploaded into the COD database (#11 in the flowchart). Cancer-specific mortality modeling then followed the general CODEm process.

Cause of death database formatting

Formatting of data sources for the cause of death database has been described in detail elsewhere (#11 in the flowchart).[13]

CODEm models

Mortality estimates for each cancer were generated using CODEm (#12 in the flowchart). Methods describing the CODEm approach have been described elsewhere.[2,21] In brief, the CODEm modeling approach is based on the principles that all types of available data should be used even if data quality varies; that individual models but also ensemble models should be tested for their predictive validity; and that the best model or sets of models should be chosen based on the out of sample predictive validity. Models were run separately for countries with extensive and complete vital registration data and countries with less VR data to prevent an inflation in the uncertainty around the estimates in "data-rich" countries. Covariates were selected based on a possible predictive relationship between the covariate and the specific cancer mortality. Level 1 covariates have a proven strong relationship with the outcome such as etiological or biological roles. Level 2 covariates have a strong relationship but not a direct biological link. Covariates that are more distal in the causal chain or are mediated through Level 1 or 2 covariates are categorized as Level 3.[21] Differences in covariate selection between GBD 2016 and GBD 2017 by cause and direction of the covariate can be found in eTable 9.

Liver cancer etiology split models

For GBD 2017, the etiologies for liver cancer were expanded to include a separate etiology of liver cancer due to non-alcoholic steatohepatitis (NASH). To find the proportion of liver cancer cases due to the five etiology groups included in GBD (1. Liver cancer due to hepatitis B, 2. Liver cancer due to hepatitis C, 3. Liver cancer due to alcohol, 4. Liver cancer due to NASH, 5. Liver cancer due to other

causes), a systematic literature search was performed in PubMed on 10/24/2016 using the following search string: “("liver neoplasms"[All Fields] OR "HCC"[All Fields] OR "liver cancer"[All Fields] OR "Carcinoma, Hepatocellular"[Mesh]) AND (("hepatitis B"[All Fields] OR "Hepatitis B"[Mesh] OR "Hepatitis B virus"[Mesh] OR "Hepatitis B Antibodies"[Mesh] OR "Hepatitis B Antigens"[Mesh]) OR ("hepatitis C"[All Fields] OR "Hepatitis C"[Mesh] OR "hepatitis C antibodies"[MESH] OR "Hepatitis C Antigens"[Mesh] OR "Hepacivirus"[Mesh]) OR ("alcohol"[All Fields] OR "Alcohol Drinking"[Mesh] OR "Alcohol-Related Disorders"[Mesh] OR "Alcoholism"[Mesh] OR "Alcohol-Induced Disorders"[Mesh])) NOT (animals[MeSH] NOT humans[MeSH])”. Also, studies not found through this search but included in the meta-analysis by de Martel et al, were included.[22] We also included the study by Hong et al, after the authors provided us with additional data on the overlap in risk factors.[23]

Studies were included if the study population was representative of liver cancer population for the respective location. For each study, the proportions of liver cancer due to the five specific risk factors were calculated. Cases were considered to be due to NASH when the manuscript explicitly listed the etiology to be NASH or non-alcoholic fatty liver disease (NAFLD). Cases where the etiology was listed as “cryptogenic,” “idiopathic,” or “unknown” were included within the “other causes” category. In manuscripts where the etiology for a case was not known but major categories could not be ruled out (for example, the study tested for hepatitis B and C, but did not assess alcohol use), these cases were excluded from the numerator of the study (in other words, did not contribute a proportion to any etiology). Remaining risk factors were included under a combined “other” group (for example, hemochromatosis, autoimmune hepatitis, Wilson’s disease, etc.). If multiple risk factors were reported for an individual patient, these were apportioned proportionally to the individual risk factors. The proportion data found through the systematic literature review were used as input for five separate DisMod-MR 2.1 models to determine the proportion of liver cancers due to the five subgroups for all locations, both sexes, and all age groups (step #16 in the flowchart). A study covariate was used for publications that only assessed liver cancer in a cirrhotic population. The reference or “gold standard” that was used for crosswalking was the compilation of all studies that assessed the etiology of liver cancer in a general population. For liver cancer due to hepatitis C and hepatitis B, a prior value of 0 was set between age 0 and 0.01. For liver cancer due to alcohol, a prior value of 0 was set for ages 0 to 5 years. For liver cancer due to hepatitis C, hepatitis C (IgG) seroprevalence was used as a covariate as well as a covariate for alcohol (liters per capita), hepatitis B prevalence (HBsAg seroprevalence), and NASH/NAFLD prevalence, forcing a negative relationship between the alcohol, hepatitis B, hepatitis C, and NASH/NAFLD covariates and the outcome of liver cancer due to alcohol proportion. For liver cancer due to hepatitis B, seroprevalence of HBsAg was used as a covariate as well as a covariate for alcohol, hepatitis C IgG seroprevalence, NASH/NAFLD prevalence, and the population coverage of three-dose Hepatitis B vaccination, forcing a negative relationship between these covariates and the outcome of liver cancer due to hepatitis B proportion. For liver cancer due to alcohol, alcohol (liters per capita) was used as a covariate as well as a covariate for proportion of alcohol abstainers, hepatitis B and hepatitis C seroprevalence, and NASH/NAFLD prevalence, forcing a negative relationship between the proportion of alcohol abstainers, NASH/NAFLD, and hepatitis B and hepatitis C covariates and the outcome of liver cancer due to alcohol proportion. For liver cancer due to NASH, NASH/NAFLD prevalence was used as a covariate as well as a covariate for obesity prevalence and mean body mass index (BMI), forcing a positive relationship between these covariates and the outcome of liver cancer due to NASH proportion. All covariates used were modeled independently. To ensure consistency between cirrhosis and liver cancer estimates and to take advantage of the data for the respective other related cause (e.g., liver

cancer due to hepatitis C and the related cause cirrhosis due to hepatitis C), we generated covariates from the liver cancer proportion models that we used in the cirrhosis etiology proportion models. We then created covariates from the cirrhosis etiology proportion models and used those in the liver cancer etiology models.

Since the proportion models are run independently of each other, the final proportion models were scaled to sum to 100% within each age, sex, year, and location, by dividing each proportion by the sum of the five (step # 17). For the liver cancer subtype mortality estimates, we multiplied the parent cause "liver cancer" by the corresponding scaled proportions (step # 18). Single cause estimates were adjusted to fit into the separately modeled all-cause mortality in the process CoDCorrect.

CoDCorrect

CODEm models estimate the individual cause-level mortality without taking into account the all-cause mortality (#13 in the flowchart). To ensure that all single causes add up to the all-cause mortality and that all child-causes add up to the parent cause, an algorithm called "CoDCorrect" is used (#14 and #15 in the flowchart). Details regarding the algorithm can be found elsewhere.[13]

Incidence estimation

GBD cancer incidence estimates were generated by dividing final mortality estimates (after CoDCorrect adjustment) by the MI ratio for the specific cancer (#1 eFigure 2). To propagate uncertainty from the MI ratios and the mortality estimates to incidence, this process was done at the 1,000-draw level. It was assumed that uncertainty in the MI ratio is independent of uncertainty in the estimated age-specific death rates.

Prevalence and YLD estimation

Prevalence is estimated as 10-year prevalence for all cancers. After transforming the final GBD cancer mortality estimates to incidence estimates (step 1 in the flowchart), incidence was combined with the relative yearly survival estimates up to 10 years (step 7 in the flowchart). For GBD 2017 we updated our methods to more directly utilize MIRs to generate these yearly cancer relative survival estimates. Previous reports suggest that the value of (1 – MIR) may serve as a proxy for 5-year relative survival, with the exact correlation varying slightly by cancer type.[24] We used SEER*Stat to obtain national mortality, incidence, and relative survival statistics from the nine SEER registries reporting from 1980 to 2014 (step 2), by cancer type, sex, 5-year blocks (i.e., 1980–1984, 1985–1989, etc.), and 5-year age groups (except combining 80+). For each cancer, we modeled 5-year relative survival with the SEER MIRs using Poisson regression, weighted by the number of incident cases (step 3). To reduce variability due to small samples, we only included MIRs based on at least 25 incident cases (except for the rarer cancers mesothelioma, nasopharyngeal cancer, and acute myeloid leukemia, where MIRs based on at least 10 cases were included). These models were then applied to the GBD MIR estimates to predict an estimated 5-year survival for each age/sex/year/location (winsorized to between 0 and 100% survival; step 4). To obtain yearly survival estimates up to 10 years, we compared these estimates to the SEER sex-specific all-ages relative survival statistics from 2004 (the latest year with 10-year survival available). The proportion of the predicted GBD survival estimate to the SEER survival statistic was used to scale the SEER 10-year relative survival curve for each country (step 5).

To transform relative to absolute survival (adjusting for background mortality), GBD 2017 lifetables were used (step 6 and 7 in the flowchart) to calculate lambda values: lambda= (ln(nLxn/nLxn+1))/5, where

nLx=person years lived between ages x and x+n (from GBD lifetable). Absolute survival was then calculated using an exponential survival function (absolute survival = relative survival * $e^{lambda*t}$).

Survivors beyond 10 years were considered cured. The survivor population prevalence was divided into two sequelae (1. diagnosis and primary therapy; 2. controlled phase). The yearly prevalence of the population that did not survive beyond 10 years was divided into the four sequelae by assigning the fixed durations for each of the diagnosis and primary therapy phase, metastatic phase, and terminal phase, and assigning the remaining prevalence to the controlled phase (step 9 in the flowchart). Duration of these four sequelae remained the same as for GBD 2016. eTable 12 lists the duration of each, along with the sources used to determine their length. YLDs were calculated by multiplying each phase with the respective disability weight (eTable 13). To generate the total YLDs for each cancer (with the exception of cancers where additional disability is added due to procedures – see next paragraph) the YLDs for each cancer sequela were added (step 13 in eFigure 2).

Additional disability was estimated for breast cancer (disability due to mastectomy), larynx cancer (disability due to laryngectomy), colon and rectum cancer (disability due to stoma), bladder cancer (disability due to incontinence), and prostatectomy (disability due to incontinence and impotence) (#10 in eFigure 2). Hospital data were used to estimate the number of cancer patients undergoing mastectomy, laryngectomy, stoma, prostatectomy, and cystectomy. These proportions remained the same as in GBD 2013, GBD 2015, and GBD 2016 and were used as input for proportion models that were run in DisMod-MR 2.1 (#9 in eFigure 2).[24] The procedure proportion (proportion of cancer population that undergoes procedures) from hospital data was used as input for a proportion model in DisMod-MR 2.1 in order to estimate the proportions for all locations, by age, and by sex.

Since colostomy or ileostomy procedures are done for reasons other than cancer, a literature review was done to determine the proportion of ostomies due to colorectal cancer. The “all cause” colostomy proportions were multiplied by 0.58 based on the results of the literature review showing that on average 58% of ostomies are done for colorectal cancer. [27–29]

The final procedure proportions were applied to the incidence cases of the respective cancers and multiplied with the proportion of the incidence population surviving for 10 years to determine the incident cases of the cancer population that underwent procedures and that survived beyond 10 years. These incident cases were used again as an input for DisMod-MR 2.1, with a remission specification of zero and an excess mortality rate prior of 0 to 0.1, as well as with increasing the age of the population and the year by 10 years to reflect prevalence after that population has survived 10 years. This approach was updated compared to GBD 2016, where we did not include an age or time shift. The results from this model are incidence and lifetime prevalent cases of persons with these cancer-related sequelae who have survived beyond 10 years.

Since disability associated with prostatectomy comes from impotence and incontinence, and not from the prostatectomy itself, 18% of the prostatectomy prevalence was assumed to have incontinence and 55% was assumed to have impotence, based on a literature review done for GBD 2013.[30–37] Cases were assigned disability for either impotence or incontinence, but no cases were assigned disability from both.

We assumed that for the population surviving up to 10 years, only the prevalence population being in remission experiences additional disability due to procedures (e.g., a woman suffering from metastatic

breast cancer does not experience additional disability due to a mastectomy during this phase). To estimate the prevalence of the cancer population in remission during the first 10 years after diagnosis with and without procedure-related disability, we multiplied the prevalence of the population in the remission phase with the proportion of the population undergoing a procedure. This step allowed us to estimate disability during the remission phase for both the population experiencing disability due to the remission phase alone, as well as the population experiencing disability from the remission phase and the additional procedure-related disability.

Lastly, the procedure sequelae prevalence and general sequelae prevalence were multiplied with their respective disability weights (eTable 13) to obtain the number of YLDs (steps 11, 12, 13 in the flowchart). The sum of these YLDs is the final YLD estimate associated with each cancer.

Probability of cancer

The cumulative probability of developing cancer for certain age groups and an approximated lifetime risk for all cancer groups (age 0 to 79) as well as the odds of developing cancer for 2017 were calculated. The method use does not take into account competing risks of death. The cancer risk is approximated using the following formula[38]:

$$Cumulative\ risk = 1 - e^{-cumulative\ rate}$$

 © 2019 Global Burden of Disease Cancer Collaboration. JAMA Oncol.

Additional method summaries for NMSC, benign and in situ neoplasms, and myelodysplastic, myeloproliferative, and other hematological neoplasms

Non-melanoma skin cancer (squamous and basal cell carcinoma)

Case definition

Non-melanoma skin cancer (NMSC) is defined as basal cell carcinoma and squamous cell carcinoma. NMSC does not include other types of skin cancer (e.g., melanoma, Merkel cell carcinoma).

Input data

We estimated squamous cell and basal cell skin cancer incidence by using cancer registry as well as primary literature and MarketScan data for incidence. Only cancer registries that were listed in CI5 VIII as registering squamous cell carcinoma or basal cell carcinoma, respectively, were included in the analysis.

Modeling strategy

For cancer registry data reported at the three-digit level (i.e., C44: Other and unspecified malignant neoplasm of skin), proportions from Karagas et al were used to split C44 into squamous cell carcinoma and basal cell carcinoma.[39] The only new data we added compared to GBD 2015 were MarketScan data. DisMod-MR 2.1 was used to model incidence and prevalence. Prevalence was calculated as function of two extreme scenarios (duration 1 versus 5 years). Country-, age-, sex-, and year-specific duration was estimated using a country-age-sex-year-specific relative access-to-care-score.

The access to care score was based on the melanoma mortality to incidence ratio:

$$Access\ to\ care = 1 - \frac{Age\ standardized\ MIR_{cys} - Age\ standardized\ MIR_{min}}{Age\ standardized\ MIR_{max} - Age\ standardized\ MIR_{min}}$$

c=country; y=year; s=sex; Age-standardized MI ratio_{min}=lowest MIR for all countries and years; Age standardized MIR_{max}=highest MIR for all countries and years

Remission was calculated as the inverse of the duration estimates and used as additional input for DisMod-MR 2.1.

To reflect differing degrees of disability due to squamous cell carcinoma, we used three levels of severity that were derived from MEPS (Medical Expenditure Panel Survey). Prevalence was multiplied by distinct disability weights (eTable 13) to generate YLDs.

Myelodysplastic, myeloproliferative, and other hematological neoplasms

Case definition

For GBD 2017 we newly estimated the myelodysplastic, myeloproliferative, and other hematological neoplasms (MDS/MPN). While these neoplasms comprise a wide variety of diseases and outcomes, we have modeled them together as a single group for 2017.

Input data

We estimated MDS/MPN deaths using vital registration data (as outlined above). We did not use cancer registry data for these neoplasms, as it has only been reported within cancer registries since 2001 and is recognized to be underreported.[40] We estimated MDS/MPN prevalence using MarketScan claims data from the United States in the years 2000, 2010, and 2012, as well as hospital and outpatient data from other health systems worldwide.

Modeling strategy

We modeled deaths for all locations and years, by age and by sex, using CODEm. As MDS/MPN can be a precursor to leukemia, our MDS/MPN CODEm model used the same covariates as the CODEm model for acute myeloid leukemia.

We modeled the prevalence of these diseases for all locations, by age, year, and by sex using a prevalence model in DisMod-MR 2.1. Each of the MarketScan 2000, 2010, and hospital data sources were crosswalked to the 2012 MarketScan data. For DisMod model specifications, cause-specific mortality rates came from the CODEm model, remission was specified to be zero, and the excess mortality rate was set to be inversely related to the Healthcare Access and Quality index covariate.

While this broad category of hematological neoplasms is heterogeneous in its components' severity or propensity for transformation to leukemia, modeling these components separately was not feasible for 2017. This is an admitted limitation, and an area of desired future improvement as data availability improves. For GBD 2017, the generic medication disability weight was assigned for all MDS/MPN cases.

Benign and in situ intestinal neoplasms; benign and in situ cervical and uterine neoplasms; other benign and in situ neoplasms

Case definition

For GBD 2017 we newly estimated three categories of benign and in-situ neoplasms: intestinal neoplasms; cervical and uterine neoplasms; and other neoplasms. Benign and in situ intestinal neoplasms were defined as any diagnosed non-invasive intestinal growth. Benign and in situ cervical and uterine neoplasms were defined as any non-invasive cervical and uterine growth, except for uterine fibroids. Other benign and in situ neoplasms were defined as any non-invasive neoplasms not covered by other causes.

Input data

To estimate the prevalence of each of these categories for all locations, by age, year, and sex, the prevalence of these neoplasms from hospital data was used as input for a prevalence model in DisMod-MR 2.1. These inputs included MarketScan claims data from the United States in the years 2000, 2010, and 2012, as well as hospital and outpatient data from other health systems worldwide. Each of these data sources were crosswalked to the 2012 MarketScan data.

Modeling strategy

In the DisMod model, excess mortality rate was specified to be zero, and remission was allowed to vary from 0 to 1. For benign and in situ cervical and uterine neoplasms, in the DisMod model, excess mortality rate was specified to be zero, and remission was allowed to vary from 0 to 0.75. For other

benign and in situ neoplasms, in the DisMod model, excess mortality rate was specified to be zero, and remission was allowed to vary from 0 to 1.

All three of these benign and in-situ neoplasms are by definition benign, localized, and not malignant. As such, no deaths or disability were attributed to their occurrence in GBD 2017.

References

1. Stanaway JD, Afshin A, Gakidou E, et al. Global, regional, and national comparative risk assessment of 84 behavioural, environmental and occupational, and metabolic risks or clusters of risks for 195 countries and territories, 1990–2017: a systematic analysis for the Global Burden of Disease Study 2017. *The Lancet*. 2018;392(10159):1923-1994. doi:10.1016/S0140-6736(18)32225-6

2. Roth GA, Abate D, Abate KH, et al. Global, regional, and national age-sex-specific mortality for 282 causes of death in 195 countries and territories, 1980–2017: a systematic analysis for the Global Burden of Disease Study 2017. *The Lancet*. 2018;392(10159):1736-1788. doi:10.1016/S0140-6736(18)32203-7

3. Kyu HH, Abate D, Abate KH, et al. Global, regional, and national disability-adjusted life-years (DALYs) for 359 diseases and injuries and healthy life expectancy (HALE) for 195 countries and territories, 1990–2017: a systematic analysis for the Global Burden of Disease Study 2017. *The Lancet*. 2018;392(10159):1859-1922. doi:10.1016/S0140-6736(18)32335-3

4. James SL, Abate D, Abate KH, et al. Global, regional, and national incidence, prevalence, and years lived with disability for 354 diseases and injuries for 195 countries and territories, 1990–2017: a systematic analysis for the Global Burden of Disease Study 2017. *The Lancet*. 2018;392(10159):1789-1858. doi:10.1016/S0140-6736(18)32279-7

5. Dicker D, Nguyen G, Abate D, et al. Global, regional, and national age-sex-specific mortality and life expectancy, 1950–2017: a systematic analysis for the Global Burden of Disease Study 2017. *The Lancet*. 2018;392(10159):1684-1735. doi:10.1016/S0140-6736(18)31891-9

6. Doll R, Payne P, Waterhouse J. *Cancer Incidence in Five Continents I*. Geneva: Union Internationale Contre le Cancer; 1966.

7. Doll R, Muir C, Waterhouse J. *Cancer Incidence in Five Continents II*. Geneva: Union Internationale Contre le Cancer, Geneva; 1970.

8. Waterhouse J, Muir C, Correa P, Powell J. *Cancer Incidence in Five Continents III*. Lyon: IARC; 1976.

9. Waterhouse J, Muir C, Shanmugaratnam K, Powell J. *Cancer Incidence in Five Continents IV*. Lyon: IARC; 1982.

10. Muir C, Mack T, Powell J, Whelan S. *Cancer Incidence in Five Continents V*. Lyon: IARC; 1987.

11. Parkin D, Muir C, Whelan S, Gao Y, Ferlay J, Powell J. *Cancer Incidence in Five Continents VI*. Lyon: IARC; 1992.

12. Parkin D, Whelan S, Ferlay J, Raymond L, Young J. *Cancer Incidence in Five Continents VII*. Lyon: IARC; 1997.

13. Parkin D, Whelan S, Ferlay J, Teppo L, Thomas D. *Cancer Incidence in Five Continents VIII*. Lyon: IARC; 2002.

14. Curado M, Edwards B, Shin H, et al. *Cancer Incidence in Five Continents IX*. Lyon: IARC; 2007. http://www.iarc.fr/en/publications/pdfs-online/epi/sp160/CI5vol9-A.pdf.

15. Forman D, Bray F, Brewster D, et al. Cancer Incidence in Five Continents X. http://ci5.iarc.fr. Published 2013.

16. Steliarova-Foucher E, O'Callaghan M, Ferlay J, Masuyer E, Forman D, Comber H, Bray F. European Cancer Observatory: Cancer Incidence, Mortality, Prevalence and Survival in Europe. International Agency for Research on Cancer. http://eco.iarc.fr. Accessed August 10, 2016.

17. Engholm G, Ferlay J, Christensen N, et al. NORDCAN--a Nordic tool for cancer information, planning, quality control and research. *Acta Oncol*. 2010;49(5):725-736. doi:10.3109/02841861003782017

18. Abajobir AA, Abbafati C, Abbas KM, et al. Global, regional, and national age-sex specific mortality for 264 causes of death, 1980–2016: a systematic analysis for the Global Burden of Disease Study 2016. *The Lancet*. 2017;390(10100):1151-1210. doi:10.1016/S0140-6736(17)32152-9

19. GBD 2013 Mortality and Causes of Death Collaborators. Global, regional, and national age–sex specific all-cause and cause-specific mortality for 240 causes of death, 1990–2013: a systematic analysis for the Global Burden of Disease Study 2013. *The Lancet*. December 2014. doi:10.1016/S0140-6736(14)61682-2

20. Barber RM, Fullman N, Sorensen RJD, et al. Healthcare Access and Quality Index based on mortality from causes amenable to personal health care in 195 countries and territories, 1990–2015: a novel analysis from the Global Burden of Disease Study 2015. *The Lancet*. 2017;390(10091):231-266. doi:10.1016/S0140-6736(17)30818-8

21. Foreman KJ, Lozano R, Lopez AD, Murray CJ. Modeling causes of death: an integrated approach using CODEm. *Popul Health Metr*. 2012;10(1):1. doi:10.1186/1478-7954-10-1

22. de Martel C, Maucort-Boulch D, Plummer M, Franceschi S. World-wide relative contribution of hepatitis B and C viruses in hepatocellular carcinoma. *Hepatol Baltim Md*. 2015;62(4):1190-1200. doi:10.1002/hep.27969

23. Hong TP, Gow P, Fink M, et al. Novel population-based study finding higher than reported hepatocellular carcinoma incidence suggests an updated approach is needed. *Hepatol Baltim Md*. 2016;63(4):1205-1212. doi:10.1002/hep.28267

24. Asadzadeh Vostakolaei F, Karim-Kos HE, Janssen-Heijnen MLG, Visser O, Verbeek ALM, Kiemeney LALM. The validity of the mortality to incidence ratio as a proxy for site-specific cancer survival. *Eur J Public Health*. 2011;21(5):573-577. doi:10.1093/eurpub/ckq120

25. Flaxman AD, Vos T, Murray C. *An Integrative Metaregression Framework for Descriptive Epidemiology*. University of Washington Press; 2015.

26. Fitzmaurice C, Dicker D, Pain A, et al. The Global Burden of Cancer 2013. *JAMA Oncol*. May 2015. doi:10.1001/jamaoncol.2015.0735

27. Canova C, Giorato E, Roveron G, Turrini P, Zanotti R. Validation of a stoma-specific quality of life questionnaire in a sample of patients with colostomy or ileostomy. *Colorectal Dis Off J Assoc Coloproctology G B Irel*. 2013;15(11):e692-698. doi:10.1111/codi.12324

28. Caricato M, Ausania F, Ripetti V, Bartolozzi F, Campoli G, Coppola R. Retrospective analysis of long-term defunctioning stoma complications after colorectal surgery. *Colorectal Dis Off J Assoc Coloproctology G B Irel*. 2007;9(6):559-561. doi:10.1111/j.1463-1318.2006.01187.x

29. Erwin-Toth P, Thompson SJ, Davis JS. Factors impacting the quality of life of people with an ostomy in North America: results from the Dialogue Study. *J Wound Ostomy Cont Nurs Off Publ Wound Ostomy Cont Nurses Soc WOCN*. 2012;39(4):417-422; quiz 423-424. doi:10.1097/WON.0b013e318259c441

30. Catalona WJ, Carvalhal GF, Mager DE, Smith DS. Potency, continence and complication rates in 1,870 consecutive radical retropubic prostatectomies. *J Urol*. 1999;162(2):433-438.

31. Donnellan SM, Duncan HJ, MacGregor RJ, Russell JM. Prospective assessment of incontinence after radical retropubic prostatectomy: objective and subjective analysis. *Urology*. 1997;49(2):225-230. doi:10.1016/S0090-4295(96)00451-7

32. Eastham JA, Kattan MW, Rogers E, et al. Risk factors for urinary incontinence after radical prostatectomy. *J Urol*. 1996;156(5):1707-1713.

33. Kundu SD, Roehl KA, Eggener SE, Antenor JAV, Han M, Catalona WJ. Potency, continence and complications in 3,477 consecutive radical retropubic prostatectomies. *J Urol*. 2004;172(6 Pt 1):2227-2231.

34. Potosky AL, Davis WW, Hoffman RM, et al. Five-Year Outcomes After Prostatectomy or Radiotherapy for Prostate Cancer: The Prostate Cancer Outcomes Study. *JNCI J Natl Cancer Inst*. 2004;96(18):1358-1367. doi:10.1093/jnci/djh259

35. Sacco E, Prayer-Galetti T, Pinto F, et al. Urinary incontinence after radical prostatectomy: incidence by definition, risk factors and temporal trend in a large series with a long-term follow-up. *BJU Int*. 2006;97(6):1234-1241. doi:10.1111/j.1464-410X.2006.06185.x

36. Stanford JL, Feng Z, Hamilton AS, et al. Urinary and sexual function after radical prostatectomy for clinically localized prostate cancer: the Prostate Cancer Outcomes Study. *JAMA*. 2000;283(3):354-360.

37. Walsh PC, Marschke P, Ricker D, Burnett AL. Patient-reported urinary continence and sexual function after anatomic radical prostatectomy. *Urology*. 2000;55(1):58-61.

38. Esteve J, Benhamou E, Raymond L. *Descriptive Epidemiology*. Vol VI. IARC Scientific Publications No.128. Lyon, France: IARC Publications; 1994.

39. Karagas MR, Greenberg ER, Spencer SK, Stukel TA, Mott LA. Increase in incidence rates of basal cell and squamous cell skin cancer in New Hampshire, USA. New Hampshire Skin Cancer Study Group. *Int J Cancer J Int Cancer*. 1999;81(4):555-559.

40. Cogle CR, Craig BM, Rollison DE, List AF. Incidence of the myelodysplastic syndromes using a novel claims-based algorithm: high number of uncaptured cases by cancer registries. *Blood*. 2011;117(26):7121-7125. doi:10.1182/blood-2011-02-337964

41. Neal RD, Din NU, Hamilton W, et al. Comparison of cancer diagnostic intervals before and after implementation of NICE guidelines: analysis of data from the UK General Practice Research Database. *Br J Cancer*. 2014;110(3):584-592. doi:10.1038/bjc.2013.791

42. *Surveillance, Epidemiology, and End Results (SEER) Program (Www.Seer.Cancer.Gov) SEER*Stat Database: Incidence - SEER 18 Regs Research Data + Hurricane Katrina Impacted Louisiana Cases, Nov 2012 Sub (1973-2010 Varying) - Linked To County Attributes - Total U.S., 1969-2011 Counties, National Cancer Institute, DCCPS, Surveillance Research Program, Surveillance Systems Branch, Released April 2013, Based on the November 2012 Submission.*

43. Allgar VL, Neal RD. Delays in the diagnosis of six cancers: analysis of data from the National Survey of NHS Patients: Cancer. *Br J Cancer*. 2005;92(11):1959-1970. doi:10.1038/sj.bjc.6602587

44. Neal RD, Cannings-John R, Hood K, et al. Excision of malignant melanomas in North Wales: effect of location and surgeon on time to diagnosis and quality of excision. *Fam Pract*. 2008;25(4):221-227. doi:10.1093/fampra/cmn036

45. Nolan RC, Chan MT-L, Heenan PJ. A clinicopathologic review of lethal nonmelanoma skin cancers in Western Australia. *J Am Acad Dermatol*. 2005;52(1):101-108. doi:10.1016/j.jaad.2004.08.016

46. Kewalramani T, Nimer SD, Zelenetz AD, et al. Progressive disease following autologous transplantation in patients with chemosensitive relapsed or primary refractory Hodgkin's disease or aggressive non-Hodgkin's lymphoma. *Bone Marrow Transplant*. 2003;32(7):673-679. doi:10.1038/sj.bmt.1704214

47. Esteban D, Tovar N, Jiménez R, et al. Patients with relapsed/refractory chronic lymphocytic leukaemia may benefit from inclusion in clinical trials irrespective of the therapy received: a case-control retrospective analsysis. *Blood Cancer J*. 2015;5:e356. doi:10.1038/bcj.2015.78

 © 2019 Global Burden of Disease Cancer Collaboration. JAMA Oncol.

Supplementary Tables and Figures

eTable 1: GATHER guidelines checklist

**Objectives and Funding**	**Reported in the Manuscript and Appendix**
1. Define the indicator(s), populations (including age, sex, and geographic entities), and time period(s) for which estimates were made.	Appendix: "Definition of indicator"
2. List the funding sources for the work.	See main manuscript
**Data Inputs**	
**For all data inputs from multiple sources that are synthesized as part of the study:**	
3. Describe how the data were identified and how the data were accessed.	Appendix: "Data sources"
4. Specify the inclusion and exclusion criteria. Identify all ad-hoc exclusions.	Appendix: "Data sources"
5. Provide information about all included data sources and their main characteristics. For each data source used, report reference information or contact name/institution, population represented, data collection method, year(s) of data collection, sex and age range, diagnostic criteria or measurement method, and sample size, as relevant.	http://ghdx.healthdata.org/gbd-2017
6. Identify and describe any categories of input data that have potentially important biases (e.g., based on characteristics listed in item 5).	Appendix: "Bias of categories of input data"
**For data inputs that contribute to the analysis but were not synthesized as part of the study:**	
7. Describe and give sources for any other data inputs.	http://ghdx.healthdata.org/gbd-2017
**For all data inputs:**	
8. Provide all data inputs in a file format from which data can be efficiently extracted (e.g., a spreadsheet rather than a PDF), including all relevant meta-data listed in item 5. For any data inputs that cannot be shared because of ethical or legal reasons, such as third-party ownership, provide a contact name or the name of the institution that retains the right to the data.	http://ghdx.healthdata.org/gbd-2017
**DATA ANALYSIS**	
9. Provide a conceptual overview of the data analysis method. A diagram may be helpful.	• Appendix Figure 1: Flowchart GBD cancer mortality, YLL estimation

	• Appendix Figure 2: Flowchart GBD cancer incidence, prevalence, YLD estimation
10. Provide a detailed description of all steps of the analysis, including mathematical formulae. This description should cover, as relevant, data cleaning, data pre-processing, data adjustments and weighting of data sources, and mathematical or statistical model(s).	Appendix: "Data Analysis"
11. Describe how candidate models were evaluated and how the final model(s) were selected.	CODEm models[3]; see Appendix Table 3: GBD 2017 covariates and level of covariates used in cause of death modeling for cancer types estimated
12. Provide the results of an evaluation of model performance, if done, as well as the results of any relevant sensitivity analysis.	See SR Figure 5 on p 20 of Supplement 2 to "Global, regional, and national age-sex-specific mortality for 282 causes of death in 195 countries and territories, 1980-2017: a systematic analysis for the Global Burden of Disease Study 2017"[4]
13. Describe methods of calculating uncertainty of the estimates. State which sources of uncertainty were, and were not, accounted for in the uncertainty analysis.	Appendix: "Data Analysis"
14. State how analytic or statistical source code used to generate estimates can be accessed.	http://ghdx.healthdata.org/gbd-2017/code
**RESULTS AND DISCUSSION**	
15. Provide published estimates in a file format from which data can be efficiently extracted.	GBD 2017 estimates are available online (http://vizhub.healthdata.org/gbd-compare and http://ghdx.healthdata.org/gbd-results-tool)
16. Report a quantitative measure of the uncertainty of the estimates (e.g., uncertainty intervals).	See main manuscript "Results"
17. Interpret results in light of existing evidence. If updating a previous set of estimates, describe the reasons for changes in estimates.	See main manuscript "Discussion"
18. Discuss limitations of the estimates. Include a discussion of any modelling assumptions or data limitations that affect interpretation of the estimates.	See main manuscript "Discussion"

eTable 2: Sources for cancer incidence and mortality-to-incidence ratio data by country, year, and registry

Location	Registry	Years available from registry	Years used for incidence	Years available for MI ratio	Years used for MI ratio
Algeria	Algiers	1993-1997	5	1993-1997	0
Algeria	Batna	2000-2012	13	2000-2012	0
Algeria	Oran	2005-2006	2	2005-2006	0
Algeria	Setif	1986-2011	26	1986-2011	0
Antilles except Aruba	Antilles except Aruba	1973-1982	10	1973-1982	0
Argentina	Bahia Blanca	1993-2007	15	1993-2007	0
Argentina	Concordia	1990-1997	8	1990-1997	0
Argentina	Cordoba	2003-2012	9	2004-2012	0
Argentina	Mendoza	2003-2012	10	2003-2012	0
Argentina	Tierra del Fuego	2003-2012	10	2003-2012	0
Australia	Capital Territory	1978-2007	25	1983-2007	0
Australia	National Registry	1982-2014	33	1968-2014	26
Australia	New South Wales	1973-2012	30	1983-2012	0
Australia	Northern Territory	1993-2012	15	1998-2012	0
Australia	Queensland	1982-2012	20	1993-2012	0
Australia	South Australia	1977-2012	36	1977-2012	0
Australia	Tasmania	1978-2012	35	1978-2012	0
Australia	Victoria	1982-2012	30	1983-2012	0
Australia	Western Australia	1982-2012	30	1983-2012	0
Austria	National Registry	1983-2012	30	1983-2012	7
Austria	Salzburg	NA		1999-2006	0
Austria	Tyrol	1988-2012	25	1988-2012	0
Austria	Vorarlberg	1993-2012	20	1993-2012	0
Bahrain	National Registry	1998-2007	10	1998-2007	0
Belarus	National Registry	1983-2012	30	1983-2012	0
Belgium	Antwerp	1998-2002	5	1998-2002	0
Belgium	Flanders	1998-2001	4	1998-2001	0
Belgium	Flanders except Limburg	1997-1998	2	1997-1998	0
Belgium	Limburg	1997-1998	2	1997-1998	0
Belgium	National Registry	2003-2012	10	2003-2012	0
Bermuda	Bermuda	1983-1987	5	1983-1987	0
Brazil	Aracaju	1996-2013	18	1996-2013	0
Brazil	Barretos	2008-2013	6	2008-2013	0
Brazil	Belem	1989-2012	24	1989-2012	0
Brazil	Belo Horizonte	2000-2011	12	2000-2011	0
Brazil	Brasilia	1998-2001	4	1998-2001	0

© 2019 Global Burden of Disease Cancer Collaboration. JAMA Oncol.

Location	Registry	Years available from registry	Years used for incidence	Years available for MI ratio	Years used for MI ratio
Brazil	Campinas	1991-2005	15	1991-2005	0
Brazil	Campo Grande	2000-2010	11	2000-2010	0
Brazil	Cuiaba	2000-2009	10	2000-2009	0
Brazil	Curitiba	1998-2012	15	1998-2012	0
Brazil	Distrito Federal	1999-2002	4	1999-2002	0
Brazil	Espirito Santo	1997-2012	16	1997-2012	0
Brazil	Florianopolis	2008-2012	5	2008-2012	0
Brazil	Fortaleza	1978-2009	32	1978-2009	0
Brazil	Goiania	1988-2012	25	1988-2012	0
Brazil	Jahu	1996-2015	20	1996-2015	0
Brazil	Joao Pessoa	1999-2012	14	1999-2012	0
Brazil	Manaus	1999-2009	11	1999-2009	0
Brazil	Mato Grosso Interior	2001-2005	5	2001-2005	0
Brazil	Natal	1999-2005	7	1999-2005	0
Brazil	Palmas	2000-2012	13	2000-2012	0
Brazil	Pocos de Caldas	2007-2011	5	2007-2011	0
Brazil	Porto Alegre	1979-2006	28	1979-2006	0
Brazil	Recife	1968-2012	45	1968-2012	0
Brazil	Roraima	2003-2010	8	2003-2010	0
Brazil	Salvador	1996-2005	10	1996-2005	0
Brazil	Santos	2008-2009	2	2008-2009	0
Brazil	Sao Paulo	1969-2013	45	1969-2013	0
Brazil	Teresina	2000-2006	7	2000-2006	0
Bulgaria	National Registry	1993-2012	20	1993-2012	12
Canada	Alberta	1960-2012	53	1960-2012	0
Canada	British Columbia	1969-2012	44	1969-2012	0
Canada	Manitoba	1958-2012	55	1958-2012	0
Canada	Maritime	1969-1987	19	1969-1987	0
Canada	National Registry	1978-2007	30	1978-2007	0
Canada	New Brunswick	1962-2012	51	1962-2012	0
Canada	Newfoundland	1969-2002	34	1969-2002	0
Canada	Newfoundland and Labrador	1960-2012	53	1960-2012	0
Canada	Northwest Territories	1983-2012	30	1983-2012	0
Canada	Northwest Territories and Yukon	1973-1987	15	1973-1987	0
Canada	Nova Scotia	1978-2012	35	1978-2012	0
Canada	Ontario	1969-2012	44	1969-2012	0
Canada	Prince Edward Island	1978-2012	35	1978-2012	0
Canada	Quebec	1963-2007	45	1963-2007	0

Location	Registry	Years available from registry	Years used for incidence	Years available for MI ratio	Years used for MI ratio
Canada	Saskatchewan	1960-2012	53	1960-2012	0
Canada	Yukon	1983-2012	30	1983-2012	0
Chile	Antofagasta	2003-2010	8	2003-2010	0
Chile	Bio Bio	2003-2012	10	2003-2012	0
Chile	Los Rios	2003-2007	5	2003-2007	0
Chile	National Registry	1959-1961	3	1959-1961	0
Chile	Valdivia	1998-2012	15	1998-2012	0
China	Anshan	1998-2012	15	1998-2012	13
China	Baoding	2009-2011	3	2009-2011	2
China	Beijing	1990-2012	23	1990-2012	21
China	Beijing Rural Areas	2011	1	2011	1
China	Beiliu	2011	1	2011	1
China	Bengbu	2011	1	2011	1
China	Benxi	2003-2011	9	2003-2011	8
China	Bijiang District, Tongren	2011	1	2011	1
China	Bincheng District, Binzhou	2011	1	2011	1
China	Binghai	2011	1	2011	1
China	Boli	2011	1	2011	1
China	Cangwu	2011	1	2011	1
China	Cangzhou	2011	1	2011	1
China	Changfeng	2011	1	2011	1
China	Changle	1990-2011	22	1990-2011	21
China	Changle	2006-2007	4	2004-2007	0
China	Changning	2011	1	2011	1
China	Changzhou	2011	1	2011	1
China	Chifeng	2009-2011	3	2009-2011	2
China	Chuzhou District, Huai'an	2004-2007	4	2004-2007	0
China	Ci County	1990-2012	23	1990-2012	12
China	Cili	2011	1	2011	1
China	Cixi	2011	1	2011	1
China	Cixian	2011	1	2011	1
China	Daan	2011	1	2011	1
China	Dafeng	2003-2011	9	2003-2011	8
China	Dalian City	1998-2011	14	1998-2011	13
China	Dancheng	2011	1	2011	1
China	Dandong	2008-2011	4	2008-2011	3
China	Daoli District, Harbin City	2005-2011	7	2005-2011	6
China	Dawukou	2011	1	2011	1
China	Dazhu	2011	1	2011	1

Location	Registry	Years available from registry	Years used for incidence	Years available for MI ratio	Years used for MI ratio
China	Decheng District, Dezhou	2011	1	2011	1
China	Dehui	2009-2011	3	2009-2011	2
China	Dingan	2011	1	2011	1
China	Donggang	2009-2011	3	2009-2011	2
China	Donghai County	2004-2011	8	2004-2011	2
China	Dunhuang	2011	1	2011	1
China	Faku	2011	1	2011	1
China	Feicheng	1998-2011	14	1998-2011	13
China	Feidong	2011	1	2011	1
China	Feixi County	2009-2011	3	2009-2011	2
China	Fusui County	1990-2011	22	1990-2011	8
China	Fuyuan	2011	1	2011	1
China	Ganyu	2004-2011	8	2004-2011	1
China	Ganzhou District, Zhangye	2011	1	2011	1
China	Gaomi	2011	1	2011	1
China	Gaotang	2011	1	2011	1
China	Gejiu	2004-2011	8	2004-2011	2
China	Gongan	2011	1	2011	1
China	Guangrao	2011	1	2011	1
China	Guangzhou City	2000-2012	13	2000-2012	11
China	Guannan	2011	1	2011	1
China	Guanyun County	2004-2012	9	2004-2012	3
China	Guilin	2011	1	2011	1
China	Guyuan	2011	1	2011	1
China	Hai'an County	2009-2011	3	2009-2011	2
China	Haimen	2003-2012	10	2003-2012	8
China	Hainan	2011	1	2011	1
China	Haining	1998-2011	14	1998-2011	13
China	Hangzhou City	2000-2012	13	2000-2012	11
China	Hanjiang District, Putian	2011	1	2011	1
China	Hefei	2010-2012	3	2010-2012	1
China	Hengdong County	2009-2012	4	2009-2012	2
China	Hepu	2011	1	2011	1
China	Hetian	2011	1	2011	1
China	Hong Kong Special Administrative Region of China	1974-2013	40	1974-2013	8
China	Hongta District, Yuxi	2011	1	2011	1
China	Hongtong	2011	1	2011	1

Location	Registry	Years available from registry	Years used for incidence	Years available for MI ratio	Years used for MI ratio
China	Hongze	2011	1	2011	1
China	Huai'an District, Huai'an	1998-2009	12	1998-2009	12
China	Huaiyin District, Huai'an	2009-2012	4	2009-2012	2
China	Huangdao District, Qingdao	2011	1	2011	1
China	Huian	2011	1	2011	1
China	Huichuan District, Zunyi	2011	1	2011	1
China	Huinong	2011	1	2011	1
China	Huixian	2011	1	2011	1
China	Huzhu	2011	1	2011	1
China	Jiangmen	2010-2012	3	2010-2012	1
China	Jianhu County	2003-2012	10	2003-2012	8
China	Jianou	2011	1	2011	1
China	Jianping	2011	1	2011	1
China	Jiashan County	1990-2012	23	1990-2012	21
China	Jiaxing	2000-2012	13	2000-2012	11
China	Jilin	2011	1	2011	1
China	Jinan	2011	1	2011	1
China	Jingan	2011	1	2011	1
China	Jingtai County	2009-2011	3	2009-2011	2
China	Jingxian	2011	1	2011	1
China	Jingyang	2011	1	2011	1
China	Jinhu County	2007-2011	5	2007-2011	4
China	Jintan District	2003-2011	9	2003-2011	7
China	Jinzhai	2011	1	2011	1
China	Jiulongpo District, Chongqing	2004-2011	8	2004-2011	4
China	Jiyuan	2011	1	2011	1
China	Junan	2011	1	2011	1
China	Kaihua	2011	1	2011	1
China	Kailu	2011	1	2011	1
China	Kaiyang	2011	1	2011	1
China	Kangping	2011	1	2011	1
China	Kunes County	2009	1	2009	1
China	Lanping	2011	1	2011	1
China	Lanzhou	2011	1	2011	1
China	Leishan	2011	1	2011	1
China	Leshan	2011	1	2011	1
China	Lhasa	2011	1	2011	1
China	Liangzhou District	2008-2011	4	2008-2011	3

Location	Registry	Years available from registry	Years used for incidence	Years available for MI ratio	Years used for MI ratio
China	Lianhu District, Xi’an	2011	1	2011	1
China	Lianshui	2011	1	2011	1
China	Lianyungang	2004-2012	9	2004-2012	4
China	Lingbi	2011	1	2011	1
China	Linhe District, Bayannaoer	2011	1	2011	1
China	Linqu County	1998-2011	14	1998-2011	13
China	Lintan	2011	1	2011	1
China	Linxian	2011	1	2011	1
China	Linzhou	1990-2012	23	1990-2012	21
China	Liuzhou	2009-2012	4	2009-2012	2
China	Liyang	2011	1	2011	1
China	Longnan	2011	1	2011	1
China	Longquanyi District, Chengdu	2011	1	2011	1
China	Lujiang	2011	1	2011	1
China	Luoshan	2011	1	2011	1
China	Luoyang	2011	1	2011	1
China	Lushan	2011	1	2011	1
China	Ma’anshan	2003-2012	10	2003-2012	8
China	Macao Special Administrative Region of China	2003-2007	5	2003-2007	0
China	Macheng	2011	1	2011	1
China	Maiji District, Tianshui	2011	1	2011	1
China	Mayang	2011	1	2011	1
China	Meixian	2011	1	2011	1
China	Minhe	2011	1	2011	1
China	Naidong	2011	1	2011	1
China	Nangang District, Harbin City	1992-2012	21	1992-2012	14
China	Nantong	2011	1	2011	1
China	Neixiang	2011	1	2011	1
China	Ningyang	2011	1	2011	1
China	Nongqishi	2011	1	2011	1
China	Pengzhou	2011	1	2011	1
China	Pingluo	2011	1	2011	1
China	Qianxi County	2009-2011	3	2009-2011	2
China	Qidong County	1990-2011	22	1990-2011	21
China	Qidong County	1983-2012	30	1983-2012	0

Location	Registry	Years available from registry	Years used for incidence	Years available for MI ratio	Years used for MI ratio
China	Qingdao	2011	1	2011	1
China	Qinghe	2011	1	2011	1
China	Qingpu	2011	1	2011	1
China	Qingyang District, Chengdu	2009-2011	3	2009-2011	2
China	Qinhuangdao	2011	1	2011	1
China	Qionghai	2011	1	2011	1
China	Renhe District, Panzhihua	2011	1	2011	1
China	Rushan	2011	1	2011	1
China	Sanmenxia	2011	1	2011	1
China	Sanya	2011	1	2011	1
China	Shanggao	2011	1	2011	1
China	Shanghai	1975-2012	38	1975-2012	21
China	Shangyu	2009-2011	3	2009-2011	2
China	Shangzhi	2009-2011	3	2009-2011	2
China	Shangzhou District, Shangluo	2011	1	2011	1
China	Shapingba District, Chongqing	2011	1	2011	1
China	Shenqiu	2011	1	2011	1
China	Shenyang City	2003-2012	10	2003-2012	8
China	Shenzen City	2004-2011	8	2004-2011	1
China	Shexian County	2003-2012	10	2003-2012	8
China	Sheyang County	2008-2012	5	2008-2012	3
China	Shifeng District, Zhuzhou	2011	1	2011	1
China	Shihezi	2011	1	2011	1
China	Shouxian	2011	1	2011	1
China	Shouyang	2011	1	2011	1
China	Sihui	1998-2011	14	1998-2011	13
China	Suzhou	2006-2011	6	2006-2011	5
China	Taixing	2004-2011	8	2004-2011	6
China	Tengchong	2011	1	2011	1
China	Tengzhou	2011	1	2011	1
China	Tianchang	2011	1	2011	1
China	Tianjin	1981-2011	31	1981-2011	16
China	Tianjin Rural Areas	2011	1	2011	1
China	Tianshan District, Urumqi	2011	1	2011	1
China	Tong’an District, Xiamen	2011	1	2011	1
China	Tongguan	2011	1	2011	1
China	Tonghua	2011	1	2011	1

Location	Registry	Years available from registry	Years used for incidence	Years available for MI ratio	Years used for MI ratio
China	Tongling	2008-2012	5	2008-2012	3
China	Wanzhouqu District, Chongqing	2011	1	2011	1
China	Wenshang County	2009-2011	3	2009-2011	2
China	Wuan	2011	1	2011	1
China	Wufeng	2011	1	2011	1
China	Wuhan City	1990-2012	23	1990-2012	18
China	Wuhu	2011	1	2011	1
China	Wuning	2011	1	2011	1
China	Wuwei	2004	1	2004	0
China	Wuxi	2006-2012	7	2006-2012	2
China	Xiamen City	2009-2011	3	2009-2011	2
China	Xiang'an District, Xiamen	2011	1	2011	1
China	Xiangfang District, Harbin	2011	1	2011	1
China	Xianju County	2009-2012	4	2009-2012	2
China	Xilinhaote	2011	1	2011	1
China	Xinghualing District, Taiyuan	2011	1	2011	1
China	Xining	2009-2011	3	2009-2011	2
China	Xinyuan	2011	1	2011	1
China	Xinzhou District, Shangrao	2011	1	2011	1
China	Xiping County	2009-2012	4	2009-2012	2
China	Xuanwei	2011	1	2011	1
China	Xuyi County	2009-2011	3	2009-2011	2
China	Xuzhou	2011	1	2011	1
China	Yakeshi	2011	1	2011	1
China	Yancheng	2011	1	2011	1
China	Yancheng District, Luohe	2011	1	2011	1
China	Yangcheng County	2003-2011	9	2003-2011	8
China	Yangquan	2009-2011	3	2009-2011	2
China	Yangshan	2011	1	2011	1
China	Yangzhong	1998-2011	14	1998-2011	13
China	Yanji	2009-2011	3	2009-2011	2
China	Yanshi	2009-2012	4	2009-2012	2
China	Yantai	2011	1	2011	1
China	Yanting County	1998-2012	15	1998-2012	13
China	Yinchuan	2011	1	2011	1
China	Yingdong District, Fuyang	2011	1	2011	1
China	Yingshan	2011	1	2011	1
China	Yiyuan	2011	1	2011	1

© 2019 Global Burden of Disease Cancer Collaboration. JAMA Oncol.

Location	Registry	Years available from registry	Years used for incidence	Years available for MI ratio	Years used for MI ratio
China	Yongding	2011	1	2011	1
China	Yongqiao District, Suzhou	2011	1	2011	1
China	Yuanhui District, Luohe	2011	1	2011	1
China	Yuanqu	2011	1	2011	1
China	Yucheng	2011	1	2011	1
China	Yuci District, Jinzhong	2011	1	2011	1
China	Yueyanglou	2009-2012	4	2009-2012	1
China	Yunmeng County	2009-2011	3	2009-2011	2
China	Yuzhong District, Chongqing	2011	1	2011	1
China	Yuzhou	2011	1	2011	1
China	Zanhuang	2011	1	2011	1
China	Zhanggong District	2009	1	2009	1
China	Zhanggong District, Ganzhou	2011	1	2011	1
China	Zhangqiu	2011	1	2011	1
China	Zhaoling District, Luohe	2011	1	2011	1
China	Zhaoyuan	2011	1	2011	1
China	Zhongshan	1998-2012	15	1998-2012	13
China	Zhongshan County	2004-2007	4	2004-2007	0
China	Zhongwei	2011	1	2011	1
China	Zhongxiang	2011	1	2011	1
China	Zhuanghe	2009-2011	3	2009-2011	2
China	Zhuhai	2010-2012	3	2010-2012	1
China	Ziliujing District	2009	1	2009	1
China	Ziliujing District, Zigong	2011	1	2011	1
China	Zixing	2011	1	2011	1
China	Zoucheng	2011	1	2011	1
Colombia	Bucaramanga	2003-2012	10	2003-2012	0
Colombia	Cali	1962-2012	51	1962-2012	0
Colombia	Manizales	2003-2012	10	2003-2012	0
Colombia	National Registry	2003-2010	8	2003-2010	0
Colombia	Pasto	2003-2012	10	2003-2012	0
Costa Rica	National Registry	1980-2011	32	1980-2013	0
Croatia	National Registry	1988-2012	25	1988-2012	8
Cuba	National Registry	1968-1987	19	1968-1986	0
Cuba	Villa Clara	1995-2007	13	1995-2007	0
Cyprus	National Registry	1998-2012	15	1998-2012	4
Czech Republic	National Registry	1983-2012	30	1983-2012	5
Denmark	National Registry	1953-2014	62	1953-2014	41

© 2019 Global Burden of Disease Cancer Collaboration. JAMA Oncol.

Location	Registry	Years available from registry	Years used for incidence	Years available for MI ratio	Years used for MI ratio
Ecuador	Cuenca	2003-2007	5	2003-2007	0
Ecuador	Quito	1985-2012	28	1985-2012	0
Egypt	Aswan	2008	1	2008	0
Egypt	Damietta	2009	1	2009	0
Egypt	Gharbiah	1999-2007	9	1999-2007	0
Egypt	Minia	2009	1	2009	0
Estonia	National Registry	1968-2012	45	1968-2012	5
Faroe Islands	Faroe Islands	1960-2005	46	1960-2006	0
Fiji	National Registry	1998-2010	13	1998-2010	11
Finland	National Registry	1953-2014	62	1953-2014	41
France	Bas Rhin	1975-2011	37	1975-2011	0
France	Calvados	1978-2012	35	1978-2012	0
France	Calvados Digestive	1978-2009	32	1978-2009	0
France	Cote d'Or	1980-2009	30	1980-2009	0
France	Doubs	1977-2012	36	1977-2012	0
France	Finistere Digestive	1984-2009	26	1984-2009	0
France	Haut Rhin	1988-2012	25	1988-2012	0
France	Herault	1987-2012	26	1987-2012	0
France	Isere	1979-2012	34	1979-2012	0
France	Loire Atlantique	1991-2012	22	1991-2012	0
France	Manche	1994-2011	18	1994-2011	0
France	Nord	2005-2009	5	2005-2009	0
France	Normandy	2002-2009	8	2002-2009	0
France	Somme	1982-2012	31	1982-2012	0
France	Tarn	1982-2012	31	1982-2012	0
France	Vendee	1998-2012	15	1998-2012	0
French Polynesia	French Polynesia	1988-2002	5	1998-2002	0
Germany	Bavaria	2002-2012	11	2002-2012	0
Germany	Berlin	1998-2007	10	1998-2007	0
Germany	Brandenburg	1998-2007	10	1998-2007	0
Germany	Bremen	2000-2012	13	2000-2012	0
Germany	Eastern States (former GDR)	1964-1989	26	1964-1989	0
Germany	Free State of Saxony	1998-2007	10	1998-2007	0
Germany	Hamburg	1969-2012	44	1969-2012	2
Germany	Lower Saxony	2003-2012	10	2003-2012	0
Germany	Mecklenburg	1998-2007	10	1998-2007	0

Location	Registry	Years available from registry	Years used for incidence	Years available for MI ratio	Years used for MI ratio
Germany	Mecklenburg-West Pomerania	1998-2007	10	1998-2007	0
Germany	Munich	1998-2012	15	1998-2012	0
Germany	National Registry	2000-2010	11	2000-2010	11
Germany	North Rhine Westphalia	1998-2007	10	1994-2007	2
Germany	Rhineland Palatinate	2000-2012	13	2000-2012	0
Germany	Saarland	1968-2012	45	1968-2012	30
Germany	Saxony-Anhalt	1998-2007	10	1998-2007	0
Germany	Schleswig Holstein	1998-2012	15	1998-2012	2
Germany	Thuringen	1998-2007	10	1998-2007	0
Germany	Westphalia	1998-2012	15	1998-2012	0
Greece	National Registry	1990-1991	2	1990-1991	0
Greenland	Greenland	1980-2014	35	1980-2014	0
Grenada	St. George's Central Hospital	1996-2000	5	1996-2000	0
Guinea	Conakry	1992-1995	4	1992-1995	0
Hungary	County Szabolcs-Szatmar	1962-1987	26	1962-1987	0
Hungary	County Vas	1962-1987	26	1962-1987	0
Hungary	Miskolc	1962-1966	5	1962-1966	0
Hungary	National Registry	2001-2011	11	2001-2011	0
Iceland	National Registry	1955-2014	60	1955-2014	41
Iran	Ardabil	1985-2008	24	1985-2008	0
Iran	Golestan	1996-2011	16	1996-2011	0
Iran	National Registry	2003-2007	5	2003-2007	0
Iraq	National Registry	2007-2011	5	2007-2011	0
Ireland	National Registry	1994-2012	19	1994-2012	0
Ireland	Southern Ireland	1980-1992	13	1980-1992	0
Israel	National Registry	1960-2012	53	1960-2012	0
Italy	Alto Adige	2003-2006	4	2003-2006	0
Italy	Biella	1995-2012	18	1995-2012	0
Italy	Brescia	1999-2007	8	1999-2006	0
Italy	Catania and Messina	2003-2005	3	2003-2005	0
Italy	Catanzaro	2003-2007	5	2003-2007	0
Italy	Como	2003-2011	9	2003-2011	0
Italy	Ferrara	1991-2011	21	1991-2011	0
Italy	Florence	1985-2002	18	1985-2002	0
Italy	Florence and Prato	1985-2010	26	1985-2010	0
Italy	Friuli Venezia Giulia	2003-2010	8	2003-2010	0
Italy	Genoa	1986-2007	21	1986-2006	0
Italy	Latina	1983-2012	30	1983-2012	0

Location	Registry	Years available from registry	Years used for incidence	Years available for MI ratio	Years used for MI ratio
Italy	Lecco	2003-2010	8	2003-2010	0
Italy	Macerata	1991-2000	10	1991-2000	0
Italy	Mantua	2003-2010	8	2003-2010	0
Italy	Milan	1999-2012	14	1999-2012	0
Italy	Modena	1988-2012	25	1988-2012	0
Italy	Naples	1998-2012	15	1998-2012	0
Italy	National Registry	2006-2009	4	2006-2009	4
Italy	North East Italy	1995-2002	8	1995-2002	0
Italy	Nuoro	2003-2012	10	2003-2012	0
Italy	Palermo	2003-2012	10	2003-2012	0
Italy	Parma	1978-2012	35	1978-2012	0
Italy	Ragusa	1978-2007	27	1981-2007	0
Italy	Reggio Emilia	1998-2012	15	1998-2012	0
Italy	Romanga	1985-2012	27	1986-2012	0
Italy	Salerno	1998-2007	10	1998-2007	0
Italy	Sassari	1993-2011	19	1993-2011	0
Italy	Sondrio	1998-2012	15	1998-2012	0
Italy	South Lombard	2003-2005	3	2003-2005	0
Italy	South Tyrol	2003-2010	3	2008-2010	0
Italy	Syracuse	1999-2012	14	1999-2012	0
Italy	Torino	1984-2007	23	1985-2007	0
Italy	Trapani	2003-2006	4	2003-2006	0
Italy	Trento	2003-2010	8	2003-2010	0
Italy	Trieste	1983-1992	9	1984-1992	0
Italy	Umbria	1994-2011	18	1994-2011	1
Italy	Varese	1976-2012	37	1976-2012	0
Italy	Veneto	1988-2010	23	1988-2010	0
Jamaica	National Registry	1958-2011	54	1958-2011	0
Japan	Aichi	1998-2012	15	1998-2012	1
Japan	Fukui	1998-2012	15	1998-2012	0
Japan	Fukuoka	1974-1975	2	1974-1975	0
Japan	Hiroshima	1978-2012	35	1978-2012	0
Japan	Miyagi	1959-2010	52	1959-2010	0
Japan	Nagasaki	1973-2012	40	1973-2012	0
Japan	National Registry	1975-2010	36	1958-2013	36
Japan	Niigata	2003-2012	10	2003-2012	0
Japan	Okayama	1966-1969	4	1966-1969	0
Japan	Osaka	1963-2012	50	1963-2012	0
Japan	Saga	1984-2007	24	1984-2007	0

Location	Registry	Years available from registry	Years used for incidence	Years available for MI ratio	Years used for MI ratio
Japan	Yamagata	1983-2012	30	1983-2012	0
Jordan	National Registry	2001-2008	8	2001-2008	0
Kenya	Nairobi	2000-2012	13	2000-2012	0
Kuwait	National Registry	1979-2012	34	1979-2012	0
Kyrgyzstan	National Registry	1986-1987	2	1986-1987	0
La Martinique	La Martinique	1981-2012	32	1981-2012	0
La Reunion	La Reunion	1988-1994	7	1988-1994	0
Latvia	National Registry	1983-2012	30	1983-2012	5
Lebanon	National Registry	1998-2007	10	1998-2007	0
Libya	Benghazi	2003-2005	3	2003-2005	0
Lithuania	National Registry	1978-2012	35	1978-2012	3
Malawi	Blantyre	1994-2007	14	1994-2007	0
Malaysia	National Registry	2003	1	2003	0
Malaysia	Penang	1998-2010	13	1998-2010	0
Malaysia	Sarawak	1998-2002	5	1998-2002	0
Mali	Bamako	1987-1996	10	1987-1996	0
Malta	National Registry	1969-2012	44	1969-2012	15
Mongolia	National Registry	2003-2007	5	2003-2007	0
Morocco	Greater Casablanca	2004	1	2004	0
Mozambique	Lourenco Marques	1956-1960	5	1956-1960	0
Namibia	National Registry	2000-2014	15	2000-2014	0
Netherlands	Eindhoven	1973-2007	35	1973-2007	0
Netherlands	Maastricht	1986-2002	17	1986-2002	0
Netherlands	National Registry	1989-2012	24	1989-2012	0
Netherlands	Three Provinces	1960-1962	3	1960-1962	0
New Zealand	National Registry	1968-2015	33	1983-2015	6
Nigeria	Calabar	2009-2013	5	2009-2013	0
Nigeria	Ibadan	1960-1969	10	1960-1969	0
Nigeria	Midwestern Nigeria	NA		2008-2009	0
Norway	National Registry	1953-2014	62	1953-2014	40
Oman	National Registry	1993-2013	21	1993-2013	0

Location	Registry	Years available from registry	Years used for incidence	Years available for MI ratio	Years used for MI ratio
Pakistan	South Karachi	1995-2002	8	1995-2002	0
Palestine	West Bank	2010-2011	2	2010-2011	0
Panama	National Registry	1988-2011	24	1988-2011	12
Paraguay	Asuncion Region	1988-1989	2	1988-1989	0
Peru	Lima	1990-2012	23	1990-2012	0
Peru	Trujillo	1984-2002	19	1984-2002	1
Philippines	Manila	1983-2012	30	1983-2012	0
Philippines	Rizal	1978-2012	35	1978-2012	0
Poland	Cieszyn	1968-1977	5	1973-1977	0
Poland	Cieszyn and Nowy Sacz	1968-1972	5	1968-1972	0
Poland	Cracow	1968-2006	34	1973-2006	0
Poland	Cracow City and District	1965-1972	8	1965-1972	0
Poland	Four Rural Areas	1965-1966	2	1965-1966	0
Poland	Katowice	1965-1977	10	1965-1974	0
Poland	Kielce	1988-2012	25	1988-2012	0
Poland	Lower Silesia	1984-2012	29	1984-2012	0
Poland	National Registry	1999-2011	13	1999-2011	13
Poland	Nowy Sacz	1973-1986	14	1973-1986	0
Poland	Opole	1985-1987	3	1985-1987	0
Poland	Rzeszow	2003-2007	5	2003-2007	0
Poland	Warsaw	1988-2002	15	1988-2002	0
Poland	Warsaw Rural	1968-1987	20	1968-1987	0
Poland	Warsaw Urban	1965-2002	38	1965-2002	0
Portugal	Azores	1997-2011	15	1981-2012	15
Portugal	Centre	2003-2007	5	2003-2007	5
Portugal	North Portugal	2000-2006	7	2000-2006	7
Portugal	Porto	1998-2002	5	1998-2002	0
Portugal	South Portugal	1998-2007	10	1998-2007	0
Portugal	Vila Nova de Gaia	1983-1997	15	1983-1997	0
Qatar	National Registry	2003-2007	5	2003-2007	0
Romania	Banat Region	1967	1	1967	0
Romania	Cluj	2007	1	2007	1
Romania	County Cluj	1974-1987	14	1974-1987	0
Romania	County Timis	1970-1972	3	1970-1972	0
Romania	Timisoara	2008	1	2008	0

Location	Registry	Years available from registry	Years used for incidence	Years available for MI ratio	Years used for MI ratio
Russian Federation	St Petersburg	1983-2007	25	1983-2007	0
Samoa	National Registry	1980-1988	9	1980-1988	0
Saudi Arabia	National Registry	1994-2012	19	1994-2012	8
Senegal	Dakar	1969-1974	6	1969-1974	0
Serbia	Central Serbia	2003-2007	5	2003-2007	5
Serbia	National Registry	1999-2002	4	1999-2002	0
Serbia	Vojvodina	1988-1997	10	1988-1997	0
Seychelles	National Registry	2009-2012	4	2009-2012	0
Singapore	National Registry	1950-2015	66	1950-2015	0
Slovakia	National Registry	1968-2010	43	1968-2010	30
Slovenia	National Registry	1956-2014	59	1956-2014	12
South Africa	Johannesburg, Bantu	1953-1955	3	1953-1955	0
South Africa	National Registry	2003-2011	9	2003-2011	0
South Africa	PROMEC	1998-2007	10	1998-2007	0
South Korea	Busan	1996-2012	17	1996-2012	0
South Korea	Daegu	1997-2012	16	1997-2012	0
South Korea	Daejeon	1998-2012	15	1998-2012	0
South Korea	Gwangju	1998-2012	15	1998-2012	0
South Korea	Incheon	1998-2012	15	1998-2012	0
South Korea	Jejudo	2000-2012	13	2000-2012	0
South Korea	Kangwha County	1986-1997	12	1986-1997	0
South Korea	National Registry	1999-2014	16	1999-2014	0
South Korea	Seoul	1993-2012	20	1993-2012	0
South Korea	Ulsan	1999-2012	14	1999-2012	0
Spain	Albacete	1991-2010	20	1991-2010	6
Spain	Asturias	1988-2010	23	1988-2010	5

© 2019 Global Burden of Disease Cancer Collaboration. JAMA Oncol.

Location	Registry	Years available from registry	Years used for incidence	Years available for MI ratio	Years used for MI ratio
Spain	Balears	1988-2005	18	1988-2005	0
Spain	Basque Country	1986-2012	27	1986-2012	4
Spain	Canary Islands	1993-2011	19	1993-2011	0
Spain	Ciudad Real	2004-2011	8	2004-2011	0
Spain	Cuenca	1993-2012	20	1993-2012	5
Spain	Girona	1980-2012	33	1980-2012	23
Spain	Granada	1985-2012	28	1985-2012	23
Spain	La Rioja	1993-2012	20	1993-2012	13
Spain	Mallorca	1988-2011	24	1988-2011	0
Spain	Murcia	1983-2010	28	1983-2010	25
Spain	Navarra	1973-2010	38	1973-2010	31
Spain	Tarragona	1980-2012	33	1980-2012	24
Spain	Zaragoza	1968-2000	33	1968-2000	0
Sri Lanka	National Registry	2001-2005	5	2001-2005	0
Sweden	National Registry	1958-2014	57	1958-2014	40
Sweden	Stockholm	1990-2016	27	1990-2016	0
Sweden	Sweden except Stockholm	1990-2016	27	1990-2016	0
Switzerland	Basel	1981-2007	27	1981-2007	0
Switzerland	Geneva	1970-2012	43	1970-2012	29
Switzerland	Graubunden	1989-1997	9	1989-1997	0
Switzerland	Graubunden and Glarus	1989-2012	24	1980-2012	20
Switzerland	National Registry	1989-2013	25	1989-2013	0
Switzerland	Neuchatel	1974-2012	39	1974-2012	5
Switzerland	St Gallen - Appenzell	1980-2012	33	1980-2012	29
Switzerland	Ticino	1996-2012	17	1996-2012	0
Switzerland	Valais	1989-2012	24	1989-2012	0
Switzerland	Vaud	1975-2012	38	1975-2012	5
Switzerland	Zurich	1980-2012	33	1980-2012	0
Taiwan	National Registry	1980-2007	28	1980-2007	28
Thailand	Bangkok	1995-2010	16	1995-2010	0
Thailand	Chiang Mai	1983-2012	30	1983-2012	0
Thailand	Chonburi	2001-2011	11	2001-2011	0
Thailand	Khon Kaen	1988-2012	25	1988-2012	0
Thailand	Lampang	1993-2012	20	1993-2012	0
Thailand	Lop Buri	2001-2003	3	2001-2003	0
Thailand	Nakhon Phanom	2001-2003	3	2001-2003	0
Thailand	Prachuap Khiri	2001-2003	3	2001-2003	0
Thailand	Rayong	2001-2003	3	2001-2003	0
Thailand	Songkhla	1993-2012	20	1993-2012	0

 © 2019 Global Burden of Disease Cancer Collaboration. JAMA Oncol.

Location	Registry	Years available from registry	Years used for incidence	Years available for MI ratio	Years used for MI ratio
Thailand	Surat Thani	2001-2003	3	2001-2003	0
Thailand	Ubon Ratchathani	2001-2003	3	2001-2003	0
Thailand	Udon Thani	2001-2003	3	2001-2003	0
The Gambia	National Registry	1987-1998	12	1987-1998	0
Trinidad and Tobago	National Registry	1995-2006	12	1995-2006	0
Tunisia	Centre Sousse	1998-2002	5	1998-2002	0
Tunisia	North Tunisia	2003-2005	3	2003-2005	0
Turkey	Ankara	2002-2005	4	2002-2005	0
Turkey	Antalya	1998-2012	15	1998-2012	0
Turkey	Edirne	2002-2012	11	2002-2012	0
Turkey	Eight Provinces	2006-2007	2	2006-2007	0
Turkey	Erzurum	2002-2012	11	2002-2012	0
Turkey	Eskisehir	2002-2012	11	2002-2012	0
Turkey	Izmir	1998-2012	15	1998-2012	0
Turkey	Nine Provinces	2008-2014	7	2008-2014	0
Turkey	Samsun	2002-2012	11	2002-2012	0
Turkey	Trabzon	2002-2012	11	2002-2012	0
Uganda	Kampala	1954-2013	60	1954-2013	0
Ukraine	National Registry	2003-2012	10	2003-2012	2
United Kingdom	Aryshire	1970-1972	3	1970-1972	0
United Kingdom	East Anglia	1988-1997	10	1988-1997	0
United Kingdom	East Midlands	1990-2014	25	1981-2014	16
United Kingdom	East Scotland	1973-1987	15	1973-1987	0
United Kingdom	East of England	1990-2014	25	1981-2014	16
United Kingdom	England	1993-2016	24	1993-2016	0
United Kingdom	England and Wales	1979-1990	12	1979-1990	0
United Kingdom	Greater London	1990-2014	25	1981-2014	16
United Kingdom	Merseyside and Cheshire	1959-2002	44	1959-2002	0
United Kingdom	National Registry	2008-2012	5	2008-2012	0

© 2019 Global Burden of Disease Cancer Collaboration. JAMA Oncol.

Location	Registry	Years available from registry	Years used for incidence	Years available for MI ratio	Years used for MI ratio
United Kingdom	North East England	1990-2014	25	1981-2014	16
United Kingdom	North East Scotland	1973-1987	15	1973-1987	0
United Kingdom	North Scotland	1973-1987	15	1973-1987	0
United Kingdom	North West England	1973-2014	42	1973-2014	24
United Kingdom	Northern England and Yorkshire	1998-2012	15	1998-2012	0
United Kingdom	Oxford	1963-2007	45	1963-2007	0
United Kingdom	Scotland	1963-2015	53	1963-2015	81
United Kingdom	South East England	1990-2014	25	1981-2014	16
United Kingdom	South East Scotland	1973-1987	15	1973-1987	0
United Kingdom	South Thames	1960-2007	38	1960-1997	0
United Kingdom	South West England	1960-2014	55	1960-2014	15
United Kingdom	Thames	1998-2007	5	1991-2007	0
United Kingdom	Trent	1963-2007	45	1963-2007	0
United Kingdom	Wales	2003-2012	10	1981-2012	2
United Kingdom	West Midlands	1960-2014	55	1960-2014	27
United Kingdom	West Scotland	1975-1992	18	1975-1992	0
United Kingdom	Yorkshire	1983-2002	20	1983-2002	0
United Kingdom	Yorkshire and the Humber	1990-2014	25	1981-2014	17
United States	Alabama	1998-2012	15	1998-2012	0
United States	Alameda County	1969-1987	5	1983-1987	0
United States	Alaska	1992-2013	22	1992-2013	0
United States	Arizona	1998-2012	15	1998-2012	0

Location	Registry	Years available from registry	Years used for incidence	Years available for MI ratio	Years used for MI ratio
United States	Arkansas	2003-2012	10	2003-2012	0
United States	Atlanta	1973-2013	41	1973-2013	46
United States	California	1998-2012	15	1998-2012	0
United States	California except SF, SJ-M, & LA	2000-2013	14	2000-2013	9
United States	Central California	1988-1992	5	1988-1992	0
United States	Colorado	1998-2012	15	1998-2012	0
United States	Connecticut	1960-2013	54	1960-2013	48
United States	Delaware	2003-2012	10	2003-2012	0
United States	Detroit	1969-2013	41	1973-2013	47
United States	District of Columbia	1998-2002	5	1998-2002	0
United States	El Paso	1960-1970	11	1960-1970	0
United States	Florida	1998-2012	15	1998-2012	0
United States	Georgia	1998-2012	15	1998-2012	0
United States	Greater Georgia	1973-2013	41	1973-2013	13
United States	Hawaii	1960-2013	54	1960-2013	48
United States	Idaho	1998-2012	15	1998-2012	0
United States	Illinois	1998-2012	15	1998-2012	0
United States	Indiana	1998-2012	15	1998-2012	0
United States	Iowa	1969-2013	41	1973-2013	48
United States	Kentucky	1973-2013	41	1973-2013	21
United States	Los Angeles	1973-2013	41	1973-2013	29
United States	Louisiana	1973-2013	41	1973-2013	20

Location	Registry	Years available from registry	Years used for incidence	Years available for MI ratio	Years used for MI ratio
United States	Maine	1998-2012	15	1998-2012	0
United States	Maryland	2008-2012	5	2008-2012	0
United States	Massachusetts	1998-2012	15	1998-2012	0
United States	Michigan	1998-2012	15	1998-2012	0
United States	Minnesota	2008-2012	5	2008-2012	0
United States	Mississippi	2003-2012	10	2003-2012	0
United States	Missouri	1998-2012	15	1998-2012	0
United States	Montana	1998-2012	15	1998-2012	0
United States	National Registry	1962-2012	51	1962-2012	0
United States	Nebraska	2003-2012	10	2003-2012	0
United States	Nevada	1959-2012	54	1959-2012	0
United States	New Hampshire	2003-2012	10	2003-2012	0
United States	New Jersey	1973-2013	41	1973-2013	21
United States	New Mexico	1969-2013	45	1969-2013	48
United States	New Orleans	1974-2012	30	1983-2012	0
United States	New York	1993-2012	20	1993-2012	0
United States	New York except New York City	1959-1987	3	1959-1961	0
United States	North Carolina	2003-2012	10	2003-2012	0
United States	North Dakota	2003-2012	10	2003-2012	0
United States	Ohio	1998-2012	15	1998-2012	0
United States	Oklahoma	1998-2012	15	1998-2012	0
United States	Oregon	1998-2012	15	1998-2012	0

Location	Registry	Years available from registry	Years used for incidence	Years available for MI ratio	Years used for MI ratio
United States	Pennsylvania	1998-2012	15	1998-2012	0
United States	Rhode Island	1998-2012	15	1998-2012	0
United States	Rural Georgia	1973-2013	41	1973-2013	24
United States	San Francisco	1969-2012	40	1973-2012	0
United States	San Francisco, Oakland, San Mateo, and Surrounding Area	1973-2013	41	1973-2013	36
United States	San Jose Monterey	1973-2013	41	1973-2013	28
United States	Seattle	1973-2013	41	1973-2013	47
United States	South Carolina	1998-2012	15	1998-2012	0
United States	South Dakota	2003-2012	10	2003-2012	0
United States	Tennessee	2003-2012	10	2003-2012	0
United States	Texas	1998-2012	15	1998-2012	0
United States	Utah	1966-2013	41	1973-2013	47
United States	Vermont	1998-2012	15	1998-2012	0
United States	Virginia	2003-2012	10	2003-2012	0
United States	Washington	1998-2012	15	1998-2012	0
United States	West Virginia	1998-2012	15	1998-2012	0
United States	Wisconsin	1998-2012	15	1998-2012	0
United States	Wyoming	2003-2012	10	2003-2012	0
Uruguay	Montevideo	1990-1995	6	1990-1995	0
Uruguay	National Registry	2002-2012	11	2002-2012	0
Vietnam	Hanoi	1991-1997	7	1991-1997	0
Vietnam	Ho Chi Minh	1995-2012	18	1995-2012	0
Zimbabwe	Bulawayo	1963-1972	10	1963-1972	0
Zimbabwe	Harare	1990-2006	17	1990-2006	0

Location	Registry	Years available from registry	Years used for incidence	Years available for MI ratio	Years used for MI ratio
Zimbabwe	National Registry	2005-2006	2	2005-2006	0

 © 2019 Global Burden of Disease Cancer Collaboration. JAMA Oncol.

eTable 3: Number of site-years for cancer mortality data by type

Cause	VR GBD 2016	VR GBD 2017	VR change GBD 2016 to GBD 2017	VA GBD 2016	VA GBD 2017	VA change GBD 2016 to GBD 2017	CR GBD 2016	CR GBD 2017	CR change GBD 2016 to GBD 2017	Total GBD 2016	Total GBD 2017	Total change GBD 2016 to GBD 2017
All malignant neoplasms	540770	656430	21%	6541	6900	5%	82457	129641	57%	629768	792971	26%
Lip and oral cavity cancer	15962	19322	21%	585	638	9%	2758	4315	56%	19305	24275	26%
Nasopharynx cancer	15988	19320	21%				2806	4411	57%	18794	23731	26%
Other pharynx cancer	15967	19320	21%	374	374	0%	2782	4404	58%	19123	24098	26%
Esophageal cancer	16298	19614	20%	590	648	10%	2849	4453	56%	19737	24715	25%
Stomach cancer	16304	19618	20%	374	374	0%	2873	4474	56%	19551	24466	25%

 © 2019 Global Burden of Disease Cancer Collaboration. JAMA Oncol.

**Cause**	**VR GBD 2016**	**VR GBD 2017**	**VR change GBD 2016 to GBD 2017**	**VA GBD 2016**	**VA GBD 2017**	**VA change GBD 2016 to GBD 2017**	**CR GBD 2016**	**CR GBD 2017**	**CR change GBD 2016 to GBD 2017**	**Total GBD 2016**	**Total GBD 2017**	**Total change GBD 2016 to GBD 2017**
All malignant neoplasms	540770	656430	21%	6541	6900	5%	82457	129641	57%	629768	792971	26%
Colon and rectum cancer	16303	19618	20%	602	660	10%	2873	4474	56%	19778	24752	25%
Liver cancer	16028	19334	21%	374	374	0%	2859	4464	56%	19261	24172	25%
Gallbladder and biliary tract cancer	14972	18771	25%				2779	4400	58%	17751	23171	31%
Pancreatic cancer	15701	19321	23%				2862	4472	56%	18563	23793	28%
Larynx cancer	16280	19614	20%	374	374	0%	2846	4456	57%	19500	24444	25%

© 2019 Global Burden of Disease Cancer Collaboration. JAMA Oncol.

**Cause**	**VR GBD 2016**	**VR GBD 2017**	**VR change GBD 2016 to GBD 2017**	**VA GBD 2016**	**VA GBD 2017**	**VA change GBD 2016 to GBD 2017**	**CR GBD 2016**	**CR GBD 2017**	**CR change GBD 2016 to GBD 2017**	**Total GBD 2016**	**Total GBD 2017**	**Total change GBD 2016 to GBD 2017**
All malignant neoplasms	540770	656430	21%	6541	6900	5%	82457	129641	57%	629768	792971	26%
Tracheal, bronchus, and lung cancer	16303	19618	20%	602	655	9%	2853	4459	56%	19758	24732	25%
Malignant skin melanoma	16018	19322	21%				2704	4351	61%	18722	23673	26%
Non-melanoma skin cancer	15581	19329	24%							15581	19329	24%

© 2019 Global Burden of Disease Cancer Collaboration. JAMA Oncol.

**Cause**	**VR GBD 2016**	**VR GBD 2017**	**VR change GBD 2016 to GBD 2017**	**VA GBD 2016**	**VA GBD 2017**	**VA change GBD 2016 to GBD 2017**	**CR GBD 2016**	**CR GBD 2017**	**CR change GBD 2016 to GBD 2017**	**Total GBD 2016**	**Total GBD 2017**	**Total change GBD 2016 to GBD 2017**
All malignant neoplasms	540770	656430	21%	6541	6900	5%	82457	129641	57%	629768	792971	26%
Non-melanoma skin cancer (squamous-cell carcinoma)	14204	18772	32%							14204	18772	32%
Breast cancer	16294	19618	20%	618	678	10%	2861	4458	56%	19773	24754	25%
Cervical cancer	16295	19618	20%	370	370		2809	4378	56%	19474	24366	25%
Uterine cancer	16280	19604	20%	374	374	0%	2831	4434	57%	19485	24412	25%
Ovarian cancer	15710	19318	23%				2845	4455	57%	18555	23773	28%
Prostate cancer	16247	19615	21%				2826	4455	58%	19073	24070	26%

© 2019 Global Burden of Disease Cancer Collaboration. JAMA Oncol.

**Cause**	**VR GBD 2016**	**VR GBD 2017**	**VR change GBD 2016 to GBD 2017**	**VA GBD 2016**	**VA GBD 2017**	**VA change GBD 2016 to GBD 2017**	**CR GBD 2016**	**CR GBD 2017**	**CR change GBD 2016 to GBD 2017**	**Total GBD 2016**	**Total GBD 2017**	**Total change GBD 2016 to GBD 2017**
All malignant neoplasms	540770	656430	21%	6541	6900	5%	82457	129641	57%	629768	792971	26%
Testicular cancer	14549	18774	29%	130	160	23%	2734	4425	62%	17413	23359	34%
Kidney cancer	16010	19318	21%				2716	4342	60%	18726	23660	26%
Bladder cancer	16010	19320	21%				2656	4279	61%	18666	23599	26%
Brain and nervous system cancer	15705	19321	23%	378	427	13%	2885	4478	55%	18968	24226	28%
Thyroid cancer	15634	19319	24%				2826	4457	58%	18460	23776	29%
Mesothelioma	7418	10944	48%							7418	10944	48%
Hodgkin lymphoma	15991	19319	21%				2762	4439	61%	18753	23758	27%

© 2019 Global Burden of Disease Cancer Collaboration. JAMA Oncol.

**Cause**	**VR GBD 2016**	**VR GBD 2017**	**VR change GBD 2016 to GBD 2017**	**VA GBD 2016**	**VA GBD 2017**	**VA change GBD 2016 to GBD 2017**	**CR GBD 2016**	**CR GBD 2017**	**CR change GBD 2016 to GBD 2017**	**Total GBD 2016**	**Total GBD 2017**	**Total change GBD 2016 to GBD 2017**
All malignant neoplasms	540770	656430	21%	6541	6900	5%	82457	129641	57%	629768	792971	26%
Non-Hodgkin lymphoma	16011	19321	21%				2860	4468	56%	18871	23789	26%
Multiple myeloma	16005	19318	21%				2737	4426	62%	18742	23744	27%
Leukemia	16851	19617	16%	585	584	0%	2925	4462	53%	20361	24663	21%
Acute lymphoid leukemia	13930	16763	20%				1130	1672	48%	15060	18435	22%

© 2019 Global Burden of Disease Cancer Collaboration. JAMA Oncol.

**Cause**	**VR GBD 2016**	**VR GBD 2017**	**VR change GBD 2016 to GBD 2017**	**VA GBD 2016**	**VA GBD 2017**	**VA change GBD 2016 to GBD 2017**	**CR GBD 2016**	**CR GBD 2017**	**CR change GBD 2016 to GBD 2017**	**Total GBD 2016**	**Total GBD 2017**	**Total change GBD 2016 to GBD 2017**
All malignant neoplasms	540770	656430	21%	6541	6900	5%	82457	129641	57%	629768	792971	26%
Chronic lymphoid leukemia	13926	16763	20%				1104	1651	50%	15030	18414	23%
Acute myeloid leukemia	13930	16763	20%				1118	2490	123%	15048	19253	28%
Chronic myeloid leukemia	13930	16763	20%				1124	1664	48%	15054	18427	22%
Other leukemia	15986	16765		211	210	0%	1913	3935		18110	20910	

© 2019 Global Burden of Disease Cancer Collaboration. JAMA Oncol.

Cause	VR GBD 2016	VR GBD 2017	VR change GBD 2016 to GBD 2017	VA GBD 2016	VA GBD 2017	VA change GBD 2016 to GBD 2017	CR GBD 2016	CR GBD 2017	CR change GBD 2016 to GBD 2017	Total GBD 2016	Total GBD 2017	Total change GBD 2016 to GBD 2017
All malignant neoplasms	540770	656430	21%	6541	6900	5%	82457	129641	57%	629768	792971	26%
Other malignant neoplasms	16149	19376	54%				2951	3140	21%	19100	22516	18%

eTable 4: List of International Classification of Diseases (ICD) codes mapped to the Global Burden of Disease cause list for cancer incidence data

Cause	ICD10	ICD9
Lip and oral cavity cancer	C00-C07, C08-C08.9, Z85.81-Z85.810	140-145.9, V76.42
Nasopharynx cancer	C11-C11.9	147-147.9
Other pharynx cancer	C09-C10.9, C12-C13.9	146-146.9, 148-148.9
Esophageal cancer	C15-C15.9, Z85.01	150-150.9
Stomach cancer	C16-C16.9, Z12.0, Z85.02-Z85.028	151-151.9, 209.23, V10.04
Colon and rectum cancer	C18-C19.0, C20, C21-C21.8, Z12.1-Z12.13, Z85.03-Z85.048, Z86.010	153-154.9, 209.1-209.17, V10.05-V10.06, V76.41, V76.5-V76.52
Liver cancer	C22-C22.4, C22.7-C22.9, Z85.05	155-155.9, V10.07
Gallbladder and biliary tract cancer	C23, C24-C24.9	156-156.9
Pancreatic cancer	C25-C25.9, Z85.07	157-157.9
Larynx cancer	C32-C32.9, Z85.21	161-161.9, V10.21
Tracheal, bronchus, and lung cancer	C33, C34-C34.92, Z12.2, Z80.1-Z80.2, Z85.1-Z85.20	162-162.9, 209.21, V10.1-V10.20, V16.1-V16.2, V16.4-V16.40
Malignant skin melanoma	C43-C43.9, Z85.82-Z85.828	172-172.9
Non-melanoma skin cancer	C44-C44.99	173-173.99, 216-216.9, 232-232.9
Non-melanoma skin cancer (squamous-cell carcinoma)	C44.02, C44.12-C44.129, C44.22-C44.229, C44.32-C44.329, C44.42, C44.52-C44.529, C44.62-C44.629, C44.72-C44.729, C44.82, C44.92	173.02, 173.12, 173.22, 173.32, 173.42, 173.52, 173.62, 173.72, 173.82, 173.92
Non-melanoma skin cancer (basal-cell carcinoma)	C44.01, C44.11-C44.119, C44.21-C44.219, C44.31-C44.319, C44.41, C44.51-C44.519, C44.61-C44.619, C44.71-C44.719, C44.81, C44.91	173.01, 173.11, 173.21, 173.31, 173.41, 173.51, 173.60-173.61, 173.71, 173.81, 173.91
Breast cancer	C50-C50.629, C50.8-C50.929, Z12.3-Z12.39, Z80.3, Z85.3, Z86.000	174-175.9, V10.3, V16.3
Cervical cancer	C53-C53.9, Z12.4, Z85.41	180-180.9, V10.41, V72.32
Uterine cancer	C54-C54.3, C54.8-C54.9, Z85.42, Z86.001	182-182.9
Ovarian cancer	C56-C56.2, C56.9, Z80.41, Z85.43	183-183.0, 183.8-183.9, V10.43, V16.41
Prostate cancer	C61-C61.9, Z12.5, Z80.42, Z85.46	185-185.9, V10.46, V16.42, V76.44

**Cause**	**ICD10**	**ICD9**
Testicular cancer	C62-C62.92, Z80.43, Z85.47-Z85.48	186-186.9, V10.47-V10.48, V16.43
Kidney cancer	C64-C64.2, C64.9-C65.9, Z80.51, Z85.52-Z85.54	189-189.1, 189.5-189.6, 209.24
Bladder cancer	C67-C67.9, Z12.6-Z12.79, Z80.52, Z85.51	188-188.9, V10.51, V16.52, V76.3
Brain and nervous system cancer	C70-C70.1, C70.9-C72.9, Z85.841-Z85.848, Z86.011	191-191.9
Thyroid cancer	C73, Z85.850	193-193.9
Mesothelioma	C45-C45.2, C45.7, C45.9	
Hodgkin lymphoma	C81-C81.49, C81.7-C81.79, C81.9-C81.99, Z85.71-Z85.72	201-201.98, V10.72
Non-Hodgkin lymphoma	C82-C85.29, C85.7-C86.6, C96-C96.9	200-200.9, 202-202.98
Multiple myeloma	C88-C90.32	203-203.9
Leukemia	C91-C93.7, C93.9-C95.2, C95.7-C95.92, Z80.6, Z85.6	204-208.92, V10.59-V10.69, V16.6
Acute lymphoid leukemia	C91.0-C91.02	204.0-204.02
Chronic lymphoid leukemia	C91.1-C91.12	204.1-204.12
Acute myeloid leukemia	C92.0-C92.02, C92.3-C92.62, C93.0-C93.02, C94.0-C94.02, C94.2-C94.22, C94.4-C94.5	205.0-205.02, 205.3-205.32, 206.0-206.02, 207.0
Chronic myeloid leukemia	C92.1-C92.12	205.1-205.12, 206.1-206.12, 207.1
Other leukemia	C91.2-C91.9, C92.2, C92.7-C92.9, C93.1-C93.9, C94.1, C94.3, C94.6-C95.9	204.2-204.9, 205.2, 205.8-205.9, 206.2-207, 207.2-208.9
Other malignant neoplasms	C17, C30-C31, C37, C38, C40-C41, C47-C49, C4A, C51-C52, C57-C58, C60, C63, C66, C68, C69, C74-C75	152-152.9, 158-158.9, 160-160.9, 163-164.9, 170-171.9, 181-181.9, 183.2-183.5, 184-184.9, 187-187.9, 189.2-189.4, 189.8-190.9, 192-192.9, 194-194.8, 209-209.03, 209.22, 209.25-209.27, 209.31-209.36

© 2019 Global Burden of Disease Cancer Collaboration. JAMA Oncol.

eTable 5: List of International Classification of Diseases (ICD) codes mapped to the Global Burden of Disease cause list for cancer mortality data

Lip and oral cavity cancer	C00-C08.9, D10.0-D10.5, D11-D11.9	140-145.9, 210.0-210.6, 235.0
Nasopharynx cancer	C11-C11.9, D10.6	147-147.9, 210.7-210.9
Other pharynx cancer	C09-C10.9, C12-C13.9, D10.7	146-146.9, 148-148.9
Esophageal cancer	C15-C15.9, D00.1, D13.0	150-150.9, 211.0, 230.1
Stomach cancer	C16-C16.9, D00.2, D13.1, D37.1	151-151.9, 211.1, 230.2
Colon and rectum cancer	C18-C21.9, D01.0-D01.3, D12-D12.9, D37.3-D37.5	153-154.9, 209.1, 209.5, 211.3-211.4, 230.3-230.6, 569.0
Liver cancer	C22-C22.9, D13.4	155-155.9, 211.5
Liver cancer due to hepatitis B		
Liver cancer due to hepatitis C		
Liver cancer due to alcohol use		
Liver cancer due to NASH		
Liver cancer due to other causes		
Gallbladder and biliary tract cancer	C23-C24.9, D13.5	156-156.9
Pancreatic cancer	C25-C25.9, D13.6-D13.7	157-157.9, 211.6-211.7
Larynx cancer	C32-C32.9, D02.0, D14.1, D38.0	161-161.9, 212.1, 231.0, 235.6
Tracheal, bronchus, and lung cancer	C33-C34.9, D02.1-D02.3, D14.2-D14.3, D38.1	162-162.9, 212.2-212.3, 231.1-231.2, 235.7
Malignant skin melanoma	C43-C43.9, D03-D03.9, D22-D23.9, D48.5	172-172.9
Non-melanoma skin cancer	C44-C44.9, D04-D04.9, D49.2	173-173.9, 222.4, 232-232.9, 238.2
Non-melanoma skin cancer (squamous-cell carcinoma)	C44-C44.9, D04-D04.9, D49.2	173-173.9, 222.4, 232-232.9, 238.2
Breast cancer	C50-C50.9, D05-D05.9, D24-D24.9, D48.6, D49.3	174-175.9, 217-217.8, 233.0, 238.3, 239.3, 610-610.9
Cervical cancer	C53-C53.9, D06-D06.9, D26.0	180-180.9, 219.0, 233.1, 622.1-622.2, 622.7
Uterine cancer	C54-C54.9, D07.0-D07.2, D26.1-D26.9	182-182.9, 233.2
Ovarian cancer	C56-C56.9, D27-D27.9, D39.1	183-183.0, 220-220.9, 236.2

Prostate cancer	C61-C61.9, D07.5, D29.1, D40.0	185-185.9, 222.2, 236.5
Testicular cancer	C62-C62.9, D29.2-D29.8, D40.1-D40.8	186-186.9, 222.0, 222.3, 236.4
Kidney cancer	C64-C65.9, D30.0-D30.1, D41.0-D41.1	189.0-189.1, 189.5-189.6, 223.0-223.1
Bladder cancer	C67-C67.9, D09.0, D30.3, D41.4-D41.8, D49.4	188-188.9, 223.3, 233.7, 236.7, 239.4
Brain and nervous system cancer	C70-C72.9	191-192.9
Thyroid cancer	C73-C73.9, D09.3, D09.8, D34-D34.9, D44.0	193-193.9, 226-226.9
Mesothelioma	C45-C45.9	
Hodgkin lymphoma	C81-C81.9	201-201.9
Non-Hodgkin lymphoma	C82-C86.6, C96-C96.9	200-200.9, 202-202.9
Multiple myeloma	C88-C90.9	203-203.9
Leukemia	C91-C95.9	204-208.9
Acute lymphoid leukemia	C91.0	204.0
Chronic lymphoid leukemia	C91.1	204.1
Acute myeloid leukemia	C92.0, C92.3-C92.6, C93.0, C94.0, C94.2, C94.4-C94.5	205.0, 205.3, 206.0, 207.0
Chronic myeloid leukemia	C92.1	205.1, 206.1, 207.1
Other leukemia	C91, C91.2-C92, C92.2, C92.7-C93, C93.1-C94, C94.1, C94.3, C94.6-C95.9	204.2-204.9, 205.2, 205.8-205.9, 206.2-207, 207.2-208.9
Other malignant cancers	C17-C17.9, C30-C31.9, C37-C38.8, C40-C41.9, C47-C4A, C51-C52.9, C57-C57.8, C58-C58.0, C60-C60.9, C63-C63.8, C66-C66.9, C68.0-C68.8, C69-C69.9, C74-C75.8, D07.4, D09.2, D13.2-D13.3, D14.0, D15-D16.9, D28.0-D28.1, D28.7, D29.0, D30.2, D30.4-D30.8, D31-D31.9, D35-D35.2, D35.5-D36, D36.1-D36.7, D37.2, D38.2-D38.5, D39.2, D39.8, D41.2-D41.3, D44.1-D44.8, D48.0-D48.4	152-152.9, 158-158.9, 160-160.9, 163-164.9, 170-171.9, 181-181.9, 183.2-183.8, 184.0-184.4, 184.8, 187.1-187.8, 189.2-189.4, 189.8, 190-190.9, 194-194.8, 209.0, 209.4, 211.2, 211.8, 212.0, 212.4-212.8, 213-213.9, 221.0-221.8, 222.1, 222.8, 223.2, 223.8, 224-224.9, 227-228.9, 229.0, 229.8, 230.7-230.8, 233.4-233.5, 234.0-234.8, 235.4, 235.8, 236.1, 238.0-238.1, 239.2

eTable 6: Undefined cancer code categories (ICD-10) and respective target codes for cancer registry incidence data

**Unspecified site cancer codes**	**Target codes for redistribution of these unspecified site cancer**
C14,C14.0-C14.3,C14.8	C00-C13.99
C26,C26.0,C26.1,C26.8,C26.9	C15.00-C25.99
C39,C39.0,C39.8,C39.9	C30.00-C38.99, C45.00-C45.99
C55,C55.1,C55.9	C53.00-C54.99
C57.9	C51.00-C54.99, C56.00-C58.99
C68.9	C64.00-C68.89
C63.9	C60.00-C63.89
C75.9	C73.00-C75.89
C76,C76.4,C76.5,C76.8,C77,C77.3-C77.5,C77.8,C77.9,C78,C79,C79.2-C79.9,C80,C80.0,C80.2	C00-C99 (Except any unspecified site cancer codes)
C76.0,C76.1,C77.0,C77.1,C78.0-C78.3	C00-C13.99,C15, C30-C34.99,C37-C38.99, C40-C42.99, C43-C50.99, C69-C73.9
C76.2,C76.3,C77.2,C7.5,C78.4-C78.8,C79.0,C79.1	C15.00-C25.99, C45.00-C45.99, C48.00-C54.99, C56.00-C58.99, C61, C63.00-C63.89, C64.00-C68.99, C74.00-C75.89, C81.00-C88.99

eTable 7: Socio-demographic Index groupings by geography, based on 2017 values

**Country Name**	**SDI Quintile**
Afghanistan	Low SDI
Albania	Middle SDI
Algeria	Middle SDI
American Samoa	High-middle SDI
Andorra	High SDI
Angola	Low-middle SDI
Antigua and Barbuda	High-middle SDI
Argentina	High-middle SDI
Armenia	High-middle SDI
Australia	High SDI
Austria	High SDI
Azerbaijan	High-middle SDI
Bahrain	High-middle SDI
Bangladesh	Low SDI
Barbados	High-middle SDI
Belarus	High-middle SDI
Belgium	High SDI
Belize	Low-middle SDI
Benin	Low SDI

 © 2019 Global Burden of Disease Cancer Collaboration. JAMA Oncol.

**Country Name**	**SDI Quintile**
Bermuda	High-middle SDI
Bhutan	Low-middle SDI
Bolivia	Low-middle SDI
Bosnia and Herzegovina	High-middle SDI
Botswana	Middle SDI
Brazil	Middle SDI
Brunei	High SDI
Bulgaria	High-middle SDI
Burkina Faso	Low SDI
Burundi	Low SDI
Cambodia	Low-middle SDI
Cameroon	Low-middle SDI
Canada	High SDI
Cape Verde	Low-middle SDI
Central African Republic	Low SDI
Chad	Low SDI
Chile	High-middle SDI
China	High-middle SDI
Colombia	Middle SDI
Comoros	Low SDI
Congo	Low-middle SDI
Costa Rica	Middle SDI
Cote d'Ivoire	Low SDI
Croatia	High SDI
Cuba	Middle SDI
Cyprus	High SDI
Czech Republic	High SDI
Democratic Republic of the Congo	Low SDI
Denmark	High SDI
Djibouti	Low-middle SDI
Dominica	Middle SDI
Dominican Republic	Low-middle SDI
Ecuador	Middle SDI
Egypt	Low-middle SDI
El Salvador	Low-middle SDI
Equatorial Guinea	Middle SDI
Eritrea	Low SDI
Estonia	High SDI
Ethiopia	Low SDI
Federated States of Micronesia	Low-middle SDI
Fiji	Middle SDI
Finland	High SDI
France	High SDI
Gabon	Middle SDI
Georgia	High-middle SDI

 © 2019 Global Burden of Disease Cancer Collaboration. JAMA Oncol.

Country Name	SDI Quintile
Germany	High SDI
Ghana	Low-middle SDI
Greece	High SDI
Greenland	High-middle SDI
Grenada	Middle SDI
Guam	High-middle SDI
Guatemala	Low-middle SDI
Guinea	Low SDI
Guinea-Bissau	Low SDI
Guyana	Low-middle SDI
Haiti	Low SDI
Honduras	Low-middle SDI
Hungary	High-middle SDI
Iceland	High SDI
India	Low-middle SDI
Indonesia	Middle SDI
Iran	High-middle SDI
Iraq	Low-middle SDI
Ireland	High SDI
Israel	High-middle SDI
Italy	High SDI
Jamaica	Middle SDI
Japan	High SDI
Jordan	Middle SDI
Kazakhstan	High-middle SDI
Kenya	Low-middle SDI
Kiribati	Low SDI
Kuwait	High-middle SDI
Kyrgyzstan	Low-middle SDI
Laos	Low-middle SDI
Latvia	High SDI
Lebanon	High-middle SDI
Lesotho	Low-middle SDI
Liberia	Low SDI
Libya	High-middle SDI
Lithuania	High SDI
Luxembourg	High SDI
Macedonia	High-middle SDI
Madagascar	Low SDI
Malawi	Low SDI
Malaysia	High-middle SDI
Maldives	Middle SDI
Mali	Low SDI
Malta	High SDI
Marshall Islands	Low-middle SDI

**Country Name**	**SDI Quintile**
Mauritania	Low-middle SDI
Mauritius	High-middle SDI
Mexico	Middle SDI
Moldova	Middle SDI
Mongolia	Middle SDI
Montenegro	High-middle SDI
Morocco	Low-middle SDI
Mozambique	Low SDI
Myanmar	Low-middle SDI
Namibia	Middle SDI
Nepal	Low SDI
Netherlands	High SDI
New Zealand	High SDI
Nicaragua	Low-middle SDI
Niger	Low SDI
Nigeria	Low-middle SDI
North Korea	Low-middle SDI
Northern Mariana Islands	High-middle SDI
Norway	High SDI
Oman	High-middle SDI
Pakistan	Low-middle SDI
Palestine	Low-middle SDI
Panama	Middle SDI
Papua New Guinea	Low SDI
Paraguay	Middle SDI
Peru	Middle SDI
Philippines	Middle SDI
Poland	High SDI
Portugal	High-middle SDI
Puerto Rico	High-middle SDI
Qatar	High-middle SDI
Romania	High-middle SDI
Russian Federation	High-middle SDI
Rwanda	Low SDI
Saint Lucia	Middle SDI
Saint Vincent and the Grenadines	Middle SDI
Samoa	Low-middle SDI
Sao Tome and Principe	Low-middle SDI
Saudi Arabia	High-middle SDI
Senegal	Low SDI
Serbia	High-middle SDI
Seychelles	Middle SDI
Sierra Leone	Low SDI
Singapore	High SDI
Slovakia	High SDI

**Country Name**	**SDI Quintile**
Slovenia	High SDI
Solomon Islands	Low SDI
Somalia	Low SDI
South Africa	Middle SDI
South Korea	High SDI
South Sudan	Low SDI
Spain	High SDI
Sri Lanka	Middle SDI
Sudan	Low-middle SDI
Suriname	Middle SDI
Swaziland	Low-middle SDI
Sweden	High SDI
Switzerland	High SDI
Syria	Middle SDI
Taiwan	High SDI
Tajikistan	Low-middle SDI
Tanzania	Low SDI
Thailand	Middle SDI
The Bahamas	High-middle SDI
The Gambia	Low SDI
Timor-Leste	Low-middle SDI
Togo	Low SDI
Tonga	Middle SDI
Trinidad and Tobago	Middle SDI
Tunisia	Middle SDI
Turkey	High-middle SDI
Turkmenistan	Middle SDI
Uganda	Low SDI
Ukraine	High-middle SDI
United Arab Emirates	High-middle SDI
United Kingdom	High SDI
United States	High SDI
Uruguay	High-middle SDI
Uzbekistan	Middle SDI
Vanuatu	Low-middle SDI
Venezuela	Middle SDI
Vietnam	Middle SDI
Virgin Islands, US	High-middle SDI
Yemen	Low SDI
Zambia	Low-middle SDI
Zimbabwe	Low-middle SDI

© 2019 Global Burden of Disease Cancer Collaboration. JAMA Oncol.

eTable 8: Covariates selected for CODEm for each GBD cancer group and expected direction of covariate

Cause	Sex	Age start	Age end	Direction	Level	Covariate
Acute lymphoid leukemia	Male	0-6 days	95+ years	0	3	LDI (I$ per capita)
Acute lymphoid leukemia	Female	0-6 days	95+ years	0	3	LDI (I$ per capita)
Acute lymphoid leukemia	Male	0-6 days	95+ years	0	3	Socio-demographic Index
Acute lymphoid leukemia	Female	0-6 days	95+ years	0	3	Socio-demographic Index
Acute lymphoid leukemia	Male	0-6 days	95+ years	-1	2	Healthcare access and quality index
Acute lymphoid leukemia	Female	0-6 days	95+ years	-1	2	Healthcare access and quality index
Acute lymphoid leukemia	Male	0-6 days	95+ years	-1	3	Education (years per capita)
Acute lymphoid leukemia	Female	0-6 days	95+ years	-1	3	Education (years per capita)
Acute lymphoid leukemia	Male	0-6 days	95+ years	1	2	Alcohol (liters per capita)
Acute lymphoid leukemia	Female	0-6 days	95+ years	1	2	Alcohol (liters per capita)
Acute lymphoid leukemia	Male	0-6 days	95+ years	1	2	Smoking Prevalence
Acute lymphoid leukemia	Female	0-6 days	95+ years	1	2	Smoking Prevalence
Acute lymphoid leukemia	Male	0-6 days	95+ years	1	2	Tobacco (cigarettes per capita)
Acute lymphoid leukemia	Female	0-6 days	95+ years	1	2	Tobacco (cigarettes per capita)
Acute lymphoid leukemia	Male	0-6 days	95+ years	1	2	Cumulative Cigarettes (10 Years)
Acute lymphoid leukemia	Female	0-6 days	95+ years	1	2	Cumulative Cigarettes (10 Years)
Acute lymphoid leukemia	Male	0-6 days	95+ years	1	2	Cumulative Cigarettes (15 Years)
Acute lymphoid leukemia	Female	0-6 days	95+ years	1	2	Cumulative Cigarettes (15 Years)
Acute lymphoid leukemia	Male	0-6 days	95+ years	1	2	Cumulative Cigarettes (20 Years)
Acute lymphoid leukemia	Female	0-6 days	95+ years	1	2	Cumulative Cigarettes (20 Years)
Acute lymphoid leukemia	Male	0-6 days	95+ years	1	2	Cumulative Cigarettes (5 Years)
Acute lymphoid leukemia	Female	0-6 days	95+ years	1	2	Cumulative Cigarettes (5 Years)
Acute lymphoid leukemia	Male	0-6 days	95+ years	1	1	Log-transformed SEV scalar: Leukemia
Acute lymphoid leukemia	Female	0-6 days	95+ years	1	1	Log-transformed SEV scalar: Leukemia
Acute lymphoid leukemia	Male	0-6 days	95+ years	1	1	Log-transformed age-standardized SEV scalar: Leukemia
Acute lymphoid leukemia	Female	0-6 days	95+ years	1	1	Log-transformed age-standardized SEV scalar: Leukemia
Acute myeloid leukemia	Male	0-6 days	95+ years	0	3	LDI (I$ per capita)
Acute myeloid leukemia	Female	0-6 days	95+ years	0	3	LDI (I$ per capita)
Acute myeloid leukemia	Male	0-6 days	95+ years	0	3	Socio-demographic Index

© 2019 Global Burden of Disease Cancer Collaboration. JAMA Oncol.

Acute myeloid leukemia	Female	0-6 days	95+ years	0	3	Socio-demographic Index
Acute myeloid leukemia	Male	0-6 days	95+ years	-1	2	Healthcare access and quality index
Acute myeloid leukemia	Female	0-6 days	95+ years	-1	2	Healthcare access and quality index
Acute myeloid leukemia	Male	0-6 days	95+ years	-1	3	Education (years per capita)
Acute myeloid leukemia	Female	0-6 days	95+ years	-1	3	Education (years per capita)
Acute myeloid leukemia	Male	0-6 days	95+ years	1	2	Alcohol (liters per capita)
Acute myeloid leukemia	Female	0-6 days	95+ years	1	2	Alcohol (liters per capita)
Acute myeloid leukemia	Male	0-6 days	95+ years	1	2	Smoking Prevalence
Acute myeloid leukemia	Female	0-6 days	95+ years	1	2	Smoking Prevalence
Acute myeloid leukemia	Male	0-6 days	95+ years	1	2	Tobacco (cigarettes per capita)
Acute myeloid leukemia	Female	0-6 days	95+ years	1	2	Tobacco (cigarettes per capita)
Acute myeloid leukemia	Male	0-6 days	95+ years	1	2	Cumulative Cigarettes (10 Years)
Acute myeloid leukemia	Female	0-6 days	95+ years	1	2	Cumulative Cigarettes (10 Years)
Acute myeloid leukemia	Male	0-6 days	95+ years	1	2	Cumulative Cigarettes (15 Years)
Acute myeloid leukemia	Female	0-6 days	95+ years	1	2	Cumulative Cigarettes (15 Years)
Acute myeloid leukemia	Male	0-6 days	95+ years	1	2	Cumulative Cigarettes (20 Years)
Acute myeloid leukemia	Female	0-6 days	95+ years	1	2	Cumulative Cigarettes (20 Years)
Acute myeloid leukemia	Male	0-6 days	95+ years	1	2	Cumulative Cigarettes (5 Years)
Acute myeloid leukemia	Female	0-6 days	95+ years	1	2	Cumulative Cigarettes (5 Years)
Acute myeloid leukemia	Male	0-6 days	95+ years	1	1	Log-transformed SEV scalar: Leukemia
Acute myeloid leukemia	Female	0-6 days	95+ years	1	1	Log-transformed SEV scalar: Leukemia
Acute myeloid leukemia	Male	0-6 days	95+ years	1	1	Log-transformed age-standardized SEV scalar: Leukemia
Acute myeloid leukemia	Female	0-6 days	95+ years	1	1	Log-transformed age-standardized SEV scalar: Leukemia
Bladder cancer	Male	15-19 years	95+ years	0	3	LDI (I$ per capita)
Bladder cancer	Female	15-19 years	95+ years	0	3	LDI (I$ per capita)
Bladder cancer	Male	15-19 years	95+ years	0	3	Socio-demographic Index
Bladder cancer	Female	15-19 years	95+ years	0	3	Socio-demographic Index
Bladder cancer	Male	15-19 years	95+ years	-1	2	Healthcare access and quality index
Bladder cancer	Female	15-19 years	95+ years	-1	2	Healthcare access and quality index
Bladder cancer	Male	15-19 years	95+ years	-1	3	Education (years per capita)
Bladder cancer	Female	15-19 years	95+ years	-1	3	Education (years per capita)

© 2019 Global Burden of Disease Cancer Collaboration. JAMA Oncol.

Bladder cancer	Male	15-19 years	95+ years	1	2	Alcohol (liters per capita)
Bladder cancer	Female	15-19 years	95+ years	1	2	Alcohol (liters per capita)
Bladder cancer	Male	15-19 years	95+ years	1	1	Smoking Prevalence
Bladder cancer	Female	15-19 years	95+ years	1	1	Smoking Prevalence
Bladder cancer	Male	15-19 years	95+ years	-1	2	fruits adjusted (g)
Bladder cancer	Female	15-19 years	95+ years	-1	2	fruits adjusted (g)
Bladder cancer	Male	15-19 years	95+ years	-1	2	vegetables adjusted (g)
Bladder cancer	Female	15-19 years	95+ years	-1	2	vegetables adjusted (g)
Bladder cancer	Male	15-19 years	95+ years	1	1	Cumulative Cigarettes (10 Years)
Bladder cancer	Female	15-19 years	95+ years	1	1	Cumulative Cigarettes (10 Years)
Bladder cancer	Male	15-19 years	95+ years	1	1	Cumulative Cigarettes (15 Years)
Bladder cancer	Female	15-19 years	95+ years	1	1	Cumulative Cigarettes (15 Years)
Bladder cancer	Male	15-19 years	95+ years	1	1	Cumulative Cigarettes (5 Years)
Bladder cancer	Female	15-19 years	95+ years	1	1	Cumulative Cigarettes (5 Years)
Bladder cancer	Male	15-19 years	95+ years	1	1	Schistosomiasis Prevalence (proportion)
Bladder cancer	Female	15-19 years	95+ years	1	1	Schistosomiasis Prevalence (proportion)
Bladder cancer	Male	15-19 years	95+ years	1	1	Log-transformed SEV scalar: Bladder C
Bladder cancer	Female	15-19 years	95+ years	1	1	Log-transformed SEV scalar: Bladder C
Brain and nervous system cancer	Male	0-6 days	95+ years	0	3	LDI (I$ per capita)
Brain and nervous system cancer	Female	0-6 days	95+ years	0	3	LDI (I$ per capita)
Brain and nervous system cancer	Male	0-6 days	95+ years	0	3	Socio-demographic Index
Brain and nervous system cancer	Female	0-6 days	95+ years	0	3	Socio-demographic Index
Brain and nervous system cancer	Male	0-6 days	95+ years	-1	2	Healthcare access and quality index
Brain and nervous system cancer	Female	0-6 days	95+ years	-1	2	Healthcare access and quality index
Brain and nervous system cancer	Male	0-6 days	95+ years	-1	3	Education (years per capita)
Brain and nervous system cancer	Female	0-6 days	95+ years	-1	3	Education (years per capita)
Brain and nervous system cancer	Male	0-6 days	95+ years	1	1	Alcohol (liters per capita)
Brain and nervous system cancer	Female	0-6 days	95+ years	1	1	Alcohol (liters per capita)
Brain and nervous system cancer	Male	0-6 days	95+ years	1	1	Smoking Prevalence
Brain and nervous system cancer	Female	0-6 days	95+ years	1	1	Smoking Prevalence
Brain and nervous system cancer	Male	0-6 days	95+ years	-1	2	fruits adjusted (g)

© 2019 Global Burden of Disease Cancer Collaboration. JAMA Oncol.

Brain and nervous system cancer	Female	0-6 days	95+ years	-1	2	fruits adjusted (g)
Brain and nervous system cancer	Male	0-6 days	95+ years	-1	2	vegetables adjusted (g)
Brain and nervous system cancer	Female	0-6 days	95+ years	-1	2	vegetables adjusted (g)
Brain and nervous system cancer	Male	0-6 days	95+ years	1	1	Cumulative Cigarettes (10 Years)
Brain and nervous system cancer	Female	0-6 days	95+ years	1	1	Cumulative Cigarettes (10 Years)
Brain and nervous system cancer	Male	0-6 days	95+ years	1	1	Cumulative Cigarettes (15 Years)
Brain and nervous system cancer	Female	0-6 days	95+ years	1	1	Cumulative Cigarettes (15 Years)
Brain and nervous system cancer	Male	0-6 days	95+ years	1	2	red meats adjusted (g)
Brain and nervous system cancer	Female	0-6 days	95+ years	1	2	red meats adjusted (g)
Brain and nervous system cancer	Male	0-6 days	95+ years	1	2	Systolic Blood Pressure (mmHg)
Brain and nervous system cancer	Female	0-6 days	95+ years	1	2	Systolic Blood Pressure (mmHg)
Brain and nervous system cancer	Male	0-6 days	95+ years	1	2	Cholesterol (total, mean per capita)
Brain and nervous system cancer	Female	0-6 days	95+ years	1	2	Cholesterol (total, mean per capita)
Breast cancer	Male	15-19 years	95+ years	0	3	LDI (I$ per capita)
Breast cancer	Female	15-19 years	95+ years	0	3	LDI (I$ per capita)
Breast cancer	Male	15-19 years	95+ years	0	3	Socio-demographic Index
Breast cancer	Female	15-19 years	95+ years	0	3	Socio-demographic Index
Breast cancer	Male	15-19 years	95+ years	-1	2	Healthcare access and quality index
Breast cancer	Female	15-19 years	95+ years	-1	2	Healthcare access and quality index
Breast cancer	Male	15-19 years	95+ years	-1	3	Education (years per capita)
Breast cancer	Female	15-19 years	95+ years	-1	3	Education (years per capita)
Breast cancer	Male	15-19 years	95+ years	1	1	Mean BMI
Breast cancer	Female	15-19 years	95+ years	1	1	Mean BMI
Breast cancer	Male	15-19 years	95+ years	1	1	Alcohol (liters per capita)
Breast cancer	Female	15-19 years	95+ years	1	1	Alcohol (liters per capita)
Breast cancer	Male	15-19 years	95+ years	1	2	Smoking Prevalence
Breast cancer	Female	15-19 years	95+ years	1	2	Smoking Prevalence
Breast cancer	Female	15-19 years	95+ years	-1	2	Total Fertility Rate
Breast cancer	Female	15-19 years	95+ years	-1	2	Age-Specific Fertility Rate
Breast cancer	Male	15-19 years	95+ years	-1	2	fruits adjusted (g)
Breast cancer	Female	15-19 years	95+ years	-1	2	fruits adjusted (g)
Breast cancer	Male	15-19 years	95+ years	-1	2	vegetables adjusted (g)
Breast cancer	Female	15-19 years	95+ years	-1	2	vegetables adjusted (g)

© 2019 Global Burden of Disease Cancer Collaboration. JAMA Oncol.

Breast cancer	Male	15-19 years	95+ years	1	2	Cumulative Cigarettes (10 Years)
Breast cancer	Female	15-19 years	95+ years	1	2	Cumulative Cigarettes (10 Years)
Breast cancer	Male	15-19 years	95+ years	1	2	Cumulative Cigarettes (15 Years)
Breast cancer	Female	15-19 years	95+ years	1	2	Cumulative Cigarettes (15 Years)
Breast cancer	Male	15-19 years	95+ years	1	2	Cumulative Cigarettes (20 Years)
Breast cancer	Female	15-19 years	95+ years	1	2	Cumulative Cigarettes (20 Years)
Breast cancer	Male	15-19 years	95+ years	1	2	Cumulative Cigarettes (5 Years)
Breast cancer	Female	15-19 years	95+ years	1	2	Cumulative Cigarettes (5 Years)
Breast cancer	Male	15-19 years	95+ years	1	1	Log-transformed SEV scalar: Breast C
Breast cancer	Female	15-19 years	95+ years	1	1	Log-transformed SEV scalar: Breast C
Cervical cancer	Female	15-19 years	95+ years	0	3	LDI (I$ per capita)
Cervical cancer	Female	15-19 years	95+ years	0	3	Socio-demographic Index
Cervical cancer	Female	15-19 years	95+ years	-1	2	Healthcare access and quality index
Cervical cancer	Female	15-19 years	95+ years	-1	3	Education (years per capita)
Cervical cancer	Female	15-19 years	95+ years	1	2	Smoking Prevalence
Cervical cancer	Female	15-19 years	95+ years	1	2	Total Fertility Rate
Cervical cancer	Female	15-19 years	95+ years	1	2	Age-Specific Fertility Rate
Cervical cancer	Female	15-19 years	95+ years	-1	2	fruits adjusted (g)
Cervical cancer	Female	15-19 years	95+ years	-1	2	vegetables adjusted (g)
Cervical cancer	Female	15-19 years	95+ years	1	1	Cumulative Cigarettes (10 Years)
Cervical cancer	Female	15-19 years	95+ years	1	1	Cumulative Cigarettes (15 Years)
Cervical cancer	Female	15-19 years	95+ years	1	1	Cumulative Cigarettes (5 Years)
Cervical cancer	Female	15-19 years	95+ years	1	1	HIV age-standardized prevalence
Chronic lymphoid leukemia	Female	15-19 years	95+ years	0	3	LDI (I$ per capita)
Chronic lymphoid leukemia	Male	15-19 years	95+ years	0	3	LDI (I$ per capita)
Chronic lymphoid leukemia	Female	15-19 years	95+ years	0	3	Socio-demographic Index
Chronic lymphoid leukemia	Male	15-19 years	95+ years	0	3	Socio-demographic Index
Chronic lymphoid leukemia	Female	15-19 years	95+ years	-1	2	Healthcare access and quality index
Chronic lymphoid leukemia	Female	15-19 years	95+ years	1	1	Healthcare access and quality index
Chronic lymphoid leukemia	Male	15-19 years	95+ years	-1	2	Healthcare access and quality index
Chronic lymphoid leukemia	Female	15-19 years	95+ years	-1	3	Education (years per capita)
Chronic lymphoid leukemia	Male	15-19 years	95+ years	-1	3	Education (years per capita)
Chronic lymphoid leukemia	Female	15-19 years	95+ years	1	2	Alcohol (liters per capita)

 © 2019 Global Burden of Disease Cancer Collaboration. JAMA Oncol.

Chronic lymphoid leukemia	Male	15-19 years	95+ years	1	2	Alcohol (liters per capita)
Chronic lymphoid leukemia	Female	15-19 years	95+ years	1	2	Smoking Prevalence
Chronic lymphoid leukemia	Male	15-19 years	95+ years	1	2	Smoking Prevalence
Chronic lymphoid leukemia	Female	15-19 years	95+ years	1	2	Tobacco (cigarettes per capita)
Chronic lymphoid leukemia	Male	15-19 years	95+ years	1	2	Tobacco (cigarettes per capita)
Chronic lymphoid leukemia	Female	15-19 years	95+ years	1	2	Cumulative Cigarettes (10 Years)
Chronic lymphoid leukemia	Male	15-19 years	95+ years	1	2	Cumulative Cigarettes (10 Years)
Chronic lymphoid leukemia	Female	15-19 years	95+ years	1	2	Cumulative Cigarettes (15 Years)
Chronic lymphoid leukemia	Male	15-19 years	95+ years	1	2	Cumulative Cigarettes (15 Years)
Chronic lymphoid leukemia	Female	15-19 years	95+ years	1	2	Cumulative Cigarettes (20 Years)
Chronic lymphoid leukemia	Male	15-19 years	95+ years	1	2	Cumulative Cigarettes (20 Years)
Chronic lymphoid leukemia	Female	15-19 years	95+ years	1	2	Cumulative Cigarettes (5 Years)
Chronic lymphoid leukemia	Male	15-19 years	95+ years	1	2	Cumulative Cigarettes (5 Years)
Chronic lymphoid leukemia	Female	15-19 years	95+ years	1	1	Log-transformed SEV scalar: Leukemia
Chronic lymphoid leukemia	Male	15-19 years	95+ years	1	1	Log-transformed SEV scalar: Leukemia
Chronic lymphoid leukemia	Female	15-19 years	95+ years	1	1	Log-transformed age-standardized SEV scalar: Leukemia
Chronic lymphoid leukemia	Male	15-19 years	95+ years	1	1	Log-transformed age-standardized SEV scalar: Leukemia
Chronic myeloid leukemia	Female	28-364 days	95+ years	0	3	LDI (I$ per capita)
Chronic myeloid leukemia	Male	28-364 days	95+ years	0	3	LDI (I$ per capita)
Chronic myeloid leukemia	Female	28-364 days	95+ years	0	3	Socio-demographic Index
Chronic myeloid leukemia	Male	28-364 days	95+ years	0	3	Socio-demographic Index
Chronic myeloid leukemia	Female	28-364 days	95+ years	-1	2	Healthcare access and quality index
Chronic myeloid leukemia	Male	28-364 days	95+ years	-1	2	Healthcare access and quality index
Chronic myeloid leukemia	Female	28-364 days	95+ years	-1	3	Education (years per capita)
Chronic myeloid leukemia	Male	28-364 days	95+ years	-1	3	Education (years per capita)
Chronic myeloid leukemia	Female	28-364 days	95+ years	1	2	Alcohol (liters per capita)
Chronic myeloid leukemia	Male	28-364 days	95+ years	1	2	Alcohol (liters per capita)
Chronic myeloid leukemia	Female	28-364 days	95+ years	1	2	Smoking Prevalence
Chronic myeloid leukemia	Male	28-364 days	95+ years	1	2	Smoking Prevalence
Chronic myeloid leukemia	Female	28-364 days	95+ years	1	2	Tobacco (cigarettes per capita)
Chronic myeloid leukemia	Male	28-364 days	95+ years	1	2	Tobacco (cigarettes per capita)

 © 2019 Global Burden of Disease Cancer Collaboration. JAMA Oncol.

Chronic myeloid leukemia	Female	28-364 days	95+ years	1	2	Cumulative Cigarettes (10 Years)
Chronic myeloid leukemia	Male	28-364 days	95+ years	1	2	Cumulative Cigarettes (10 Years)
Chronic myeloid leukemia	Female	28-364 days	95+ years	1	2	Cumulative Cigarettes (15 Years)
Chronic myeloid leukemia	Male	28-364 days	95+ years	1	2	Cumulative Cigarettes (15 Years)
Chronic myeloid leukemia	Female	28-364 days	95+ years	1	2	Cumulative Cigarettes (20 Years)
Chronic myeloid leukemia	Male	28-364 days	95+ years	1	2	Cumulative Cigarettes (20 Years)
Chronic myeloid leukemia	Female	28-364 days	95+ years	1	2	Cumulative Cigarettes (5 Years)
Chronic myeloid leukemia	Male	28-364 days	95+ years	1	2	Cumulative Cigarettes (5 Years)
Chronic myeloid leukemia	Female	28-364 days	95+ years	1	1	Log-transformed SEV scalar: Leukemia
Chronic myeloid leukemia	Male	28-364 days	95+ years	1	1	Log-transformed SEV scalar: Leukemia
Chronic myeloid leukemia	Female	28-364 days	95+ years	1	1	Log-transformed age-standardized SEV scalar: Leukemia
Chronic myeloid leukemia	Male	28-364 days	95+ years	1	1	Log-transformed age-standardized SEV scalar: Leukemia
Colon and rectum cancer	Male	15-19 years	95+ years	0	3	LDI (I$ per capita)
Colon and rectum cancer	Female	15-19 years	95+ years	0	3	LDI (I$ per capita)
Colon and rectum cancer	Male	15-19 years	95+ years	0	3	Socio-demographic Index
Colon and rectum cancer	Female	15-19 years	95+ years	0	3	Socio-demographic Index
Colon and rectum cancer	Male	15-19 years	95+ years	-1	2	Healthcare access and quality index
Colon and rectum cancer	Female	15-19 years	95+ years	-1	2	Healthcare access and quality index
Colon and rectum cancer	Male	15-19 years	95+ years	-1	3	Education (years per capita)
Colon and rectum cancer	Female	15-19 years	95+ years	-1	3	Education (years per capita)
Colon and rectum cancer	Male	15-19 years	95+ years	1	1	Mean BMI
Colon and rectum cancer	Female	15-19 years	95+ years	1	1	Mean BMI
Colon and rectum cancer	Male	15-19 years	95+ years	1	1	Alcohol (liters per capita)
Colon and rectum cancer	Female	15-19 years	95+ years	1	1	Alcohol (liters per capita)
Colon and rectum cancer	Male	15-19 years	95+ years	1	1	Smoking Prevalence
Colon and rectum cancer	Female	15-19 years	95+ years	1	1	Smoking Prevalence
Colon and rectum cancer	Male	15-19 years	95+ years	1	1	Tobacco (cigarettes per capita)
Colon and rectum cancer	Female	15-19 years	95+ years	1	1	Tobacco (cigarettes per capita)
Colon and rectum cancer	Male	15-19 years	95+ years	-1	2	fruits adjusted (g)
Colon and rectum cancer	Female	15-19 years	95+ years	-1	2	fruits adjusted (g)
Colon and rectum cancer	Male	15-19 years	95+ years	-1	2	vegetables adjusted (g)

 © 2019 Global Burden of Disease Cancer Collaboration. JAMA Oncol.

Colon and rectum cancer	Female	15-19 years	95+ years	-1	2	vegetables adjusted (g)
Colon and rectum cancer	Male	15-19 years	95+ years	1	2	Cumulative Cigarettes (10 Years)
Colon and rectum cancer	Female	15-19 years	95+ years	1	2	Cumulative Cigarettes (10 Years)
Colon and rectum cancer	Male	15-19 years	95+ years	1	2	Cumulative Cigarettes (15 Years)
Colon and rectum cancer	Female	15-19 years	95+ years	1	2	Cumulative Cigarettes (15 Years)
Colon and rectum cancer	Male	15-19 years	95+ years	1	2	Cumulative Cigarettes (20 Years)
Colon and rectum cancer	Female	15-19 years	95+ years	1	2	Cumulative Cigarettes (20 Years)
Colon and rectum cancer	Male	15-19 years	95+ years	1	2	Cumulative Cigarettes (5 Years)
Colon and rectum cancer	Female	15-19 years	95+ years	1	2	Cumulative Cigarettes (5 Years)
Colon and rectum cancer	Male	15-19 years	95+ years	1	1	red meats adjusted (g)
Colon and rectum cancer	Female	15-19 years	95+ years	1	1	red meats adjusted (g)
Colon and rectum cancer	Male	15-19 years	95+ years	1	2	Diabetes Age-Specific Prevalence (proportion)
Colon and rectum cancer	Female	15-19 years	95+ years	1	2	Diabetes Age-Specific Prevalence (proportion)
Colon and rectum cancer	Male	15-19 years	95+ years	1	1	Log-transformed SEV scalar: Colorect C
Colon and rectum cancer	Female	15-19 years	95+ years	1	1	Log-transformed SEV scalar: Colorect C
Colon and rectum cancer	Male	15-19 years	95+ years	-1	2	milk adjusted (g)
Colon and rectum cancer	Female	15-19 years	95+ years	-1	2	milk adjusted (g)
Colon and rectum cancer	Male	15-19 years	95+ years	-1	2	nuts seeds adjusted (g)
Colon and rectum cancer	Female	15-19 years	95+ years	-1	2	nuts seeds adjusted (g)
Colon and rectum cancer	Male	15-19 years	95+ years	-1	2	pufa adjusted(percent)
Colon and rectum cancer	Female	15-19 years	95+ years	-1	2	pufa adjusted(percent)
Esophageal cancer	Male	15-19 years	95+ years	0	3	LDI (I$ per capita)
Esophageal cancer	Female	15-19 years	95+ years	0	3	LDI (I$ per capita)
Esophageal cancer	Male	15-19 years	95+ years	-1	2	Sanitation (proportion with access)
Esophageal cancer	Female	15-19 years	95+ years	-1	2	Sanitation (proportion with access)
Esophageal cancer	Male	15-19 years	95+ years	-1	2	Improved Water Source (proportion with access)
Esophageal cancer	Female	15-19 years	95+ years	-1	2	Improved Water Source (proportion with access)
Esophageal cancer	Male	15-19 years	95+ years	0	3	Socio-demographic Index
Esophageal cancer	Female	15-19 years	95+ years	0	3	Socio-demographic Index

Esophageal cancer	Male	15-19 years	95+ years	-1	2	Healthcare access and quality index
Esophageal cancer	Female	15-19 years	95+ years	-1	2	Healthcare access and quality index
Esophageal cancer	Male	15-19 years	95+ years	-1	3	Education (years per capita)
Esophageal cancer	Female	15-19 years	95+ years	-1	3	Education (years per capita)
Esophageal cancer	Male	15-19 years	95+ years	1	1	Mean BMI
Esophageal cancer	Female	15-19 years	95+ years	1	1	Mean BMI
Esophageal cancer	Male	15-19 years	95+ years	1	2	Indoor Air Pollution (All Cooking Fuels)
Esophageal cancer	Female	15-19 years	95+ years	1	2	Indoor Air Pollution (All Cooking Fuels)
Esophageal cancer	Male	15-19 years	95+ years	1	1	Alcohol (liters per capita)
Esophageal cancer	Female	15-19 years	95+ years	1	1	Alcohol (liters per capita)
Esophageal cancer	Male	15-19 years	95+ years	1	1	Smoking Prevalence
Esophageal cancer	Female	15-19 years	95+ years	1	1	Smoking Prevalence
Esophageal cancer	Male	15-19 years	95+ years	1	1	Tobacco (cigarettes per capita)
Esophageal cancer	Female	15-19 years	95+ years	1	1	Tobacco (cigarettes per capita)
Esophageal cancer	Male	15-19 years	95+ years	1	1	Log-transformed SEV scalar: Esophag C
Esophageal cancer	Female	15-19 years	95+ years	1	1	Log-transformed SEV scalar: Esophag C
Esophageal cancer	Male	15-19 years	95+ years	1	1	Log-transformed age-standardized SEV scalar: Esophag C
Esophageal cancer	Female	15-19 years	95+ years	1	1	Log-transformed age-standardized SEV scalar: Esophag C
Esophageal cancer	Male	15-19 years	95+ years	-1	1	fruits adjusted (g)
Esophageal cancer	Female	15-19 years	95+ years	-1	1	fruits adjusted (g)
Esophageal cancer	Male	15-19 years	95+ years	-1	2	vegetables adjusted (g)
Esophageal cancer	Female	15-19 years	95+ years	-1	2	vegetables adjusted (g)
Gallbladder and biliary tract cancer	Male	15-19 years	95+ years	0	3	LDI (I$ per capita)
Gallbladder and biliary tract cancer	Female	15-19 years	95+ years	0	3	LDI (I$ per capita)
Gallbladder and biliary tract cancer	Male	15-19 years	95+ years	0	3	Socio-demographic Index
Gallbladder and biliary tract cancer	Female	15-19 years	95+ years	0	3	Socio-demographic Index
Gallbladder and biliary tract cancer	Male	15-19 years	95+ years	-1	2	Healthcare access and quality index
Gallbladder and biliary tract cancer	Female	15-19 years	95+ years	-1	2	Healthcare access and quality index
Gallbladder and biliary tract cancer	Male	15-19 years	95+ years	-1	3	Education (years per capita)
Gallbladder and biliary tract cancer	Female	15-19 years	95+ years	-1	3	Education (years per capita)
Gallbladder and biliary tract cancer	Male	15-19 years	95+ years	1	1	Mean BMI

© 2019 Global Burden of Disease Cancer Collaboration. JAMA Oncol.

Gallbladder and biliary tract cancer	Female	15-19 years	95+ years	1	1	Mean BMI
Gallbladder and biliary tract cancer	Male	15-19 years	95+ years	1	2	Alcohol (liters per capita)
Gallbladder and biliary tract cancer	Female	15-19 years	95+ years	1	2	Alcohol (liters per capita)
Gallbladder and biliary tract cancer	Male	15-19 years	95+ years	1	2	Smoking Prevalence
Gallbladder and biliary tract cancer	Female	15-19 years	95+ years	1	2	Smoking Prevalence
Gallbladder and biliary tract cancer	Male	15-19 years	95+ years	1	2	Tobacco (cigarettes per capita)
Gallbladder and biliary tract cancer	Female	15-19 years	95+ years	1	2	Tobacco (cigarettes per capita)
Gallbladder and biliary tract cancer	Male	15-19 years	95+ years	-1	2	fruits adjusted (g)
Gallbladder and biliary tract cancer	Female	15-19 years	95+ years	-1	2	fruits adjusted (g)
Gallbladder and biliary tract cancer	Male	15-19 years	95+ years	-1	2	vegetables adjusted (g)
Gallbladder and biliary tract cancer	Female	15-19 years	95+ years	-1	2	vegetables adjusted (g)
Gallbladder and biliary tract cancer	Male	15-19 years	95+ years	1	2	Cumulative Cigarettes (10 Years)
Gallbladder and biliary tract cancer	Female	15-19 years	95+ years	1	2	Cumulative Cigarettes (10 Years)
Gallbladder and biliary tract cancer	Male	15-19 years	95+ years	1	2	Cumulative Cigarettes (5 Years)
Gallbladder and biliary tract cancer	Female	15-19 years	95+ years	1	2	Cumulative Cigarettes (5 Years)
Gallbladder and biliary tract cancer	Male	15-19 years	95+ years	1	2	Diabetes Age-Standardized Prevalence (proportion)
Gallbladder and biliary tract cancer	Female	15-19 years	95+ years	1	2	Diabetes Age-Standardized Prevalence (proportion)
Gallbladder and biliary tract cancer	Male	15-19 years	95+ years	1	1	Log-transformed SEV scalar: Gallblad C
Gallbladder and biliary tract cancer	Female	15-19 years	95+ years	1	1	Log-transformed SEV scalar: Gallblad C
Hodgkin lymphoma	Male	0-6 days	95+ years	0	3	LDI (I$ per capita)
Hodgkin lymphoma	Female	0-6 days	95+ years	0	3	LDI (I$ per capita)
Hodgkin lymphoma	Male	0-6 days	95+ years	0	3	Socio-demographic Index
Hodgkin lymphoma	Female	0-6 days	95+ years	0	3	Socio-demographic Index
Hodgkin lymphoma	Male	0-6 days	95+ years	-1	2	Healthcare access and quality index
Hodgkin lymphoma	Female	0-6 days	95+ years	-1	2	Healthcare access and quality index
Hodgkin lymphoma	Male	0-6 days	95+ years	-1	3	Education (years per capita)
Hodgkin lymphoma	Female	0-6 days	95+ years	-1	3	Education (years per capita)
Kidney cancer	Male	0-6 days	95+ years	0	3	LDI (I$ per capita)
Kidney cancer	Female	0-6 days	95+ years	0	3	LDI (I$ per capita)
Kidney cancer	Male	0-6 days	95+ years	0	3	Socio-demographic Index
Kidney cancer	Female	0-6 days	95+ years	0	3	Socio-demographic Index

Kidney cancer	Male	0-6 days	95+ years	-1	2	Healthcare access and quality index
Kidney cancer	Female	0-6 days	95+ years	-1	2	Healthcare access and quality index
Kidney cancer	Male	0-6 days	95+ years	-1	3	Education (years per capita)
Kidney cancer	Female	0-6 days	95+ years	-1	3	Education (years per capita)
Kidney cancer	Male	0-6 days	95+ years	1	1	Mean BMI
Kidney cancer	Female	0-6 days	95+ years	1	1	Mean BMI
Kidney cancer	Male	0-6 days	95+ years	1	2	Alcohol (liters per capita)
Kidney cancer	Female	0-6 days	95+ years	1	2	Alcohol (liters per capita)
Kidney cancer	Male	0-6 days	95+ years	1	2	Smoking Prevalence
Kidney cancer	Female	0-6 days	95+ years	1	2	Smoking Prevalence
Kidney cancer	Male	0-6 days	95+ years	1	1	Cumulative Cigarettes (10 Years)
Kidney cancer	Female	0-6 days	95+ years	1	1	Cumulative Cigarettes (10 Years)
Kidney cancer	Male	0-6 days	95+ years	1	1	Cumulative Cigarettes (15 Years)
Kidney cancer	Female	0-6 days	95+ years	1	1	Cumulative Cigarettes (15 Years)
Kidney cancer	Male	0-6 days	95+ years	1	1	Cumulative Cigarettes (5 Years)
Kidney cancer	Female	0-6 days	95+ years	1	1	Cumulative Cigarettes (5 Years)
Kidney cancer	Male	0-6 days	95+ years	1	2	Diabetes Age-Standardized Prevalence (proportion)
Kidney cancer	Female	0-6 days	95+ years	1	2	Diabetes Age-Standardized Prevalence (proportion)
Kidney cancer	Male	0-6 days	95+ years	1	2	Systolic Blood Pressure (mmHg)
Kidney cancer	Female	0-6 days	95+ years	1	2	Systolic Blood Pressure (mmHg)
Kidney cancer	Male	0-6 days	95+ years	1	1	Log-transformed SEV scalar: Kidney C
Kidney cancer	Female	0-6 days	95+ years	1	1	Log-transformed SEV scalar: Kidney C
Larynx cancer	Male	15-19 years	95+ years	0	3	LDI (I$ per capita)
Larynx cancer	Female	15-19 years	95+ years	0	3	LDI (I$ per capita)
Larynx cancer	Male	15-19 years	95+ years	0	3	Socio-demographic Index
Larynx cancer	Female	15-19 years	95+ years	0	3	Socio-demographic Index
Larynx cancer	Male	15-19 years	95+ years	-1	2	Healthcare access and quality index
Larynx cancer	Female	15-19 years	95+ years	-1	2	Healthcare access and quality index
Larynx cancer	Male	15-19 years	95+ years	-1	3	Education (years per capita)
Larynx cancer	Female	15-19 years	95+ years	-1	3	Education (years per capita)

© 2019 Global Burden of Disease Cancer Collaboration. JAMA Oncol.

Larynx cancer	Male	15-19 years	95+ years	1	2	Population Density (over 1000 ppl/sqkm, proportion)
Larynx cancer	Female	15-19 years	95+ years	1	2	Population Density (over 1000 ppl/sqkm, proportion)
Larynx cancer	Male	15-19 years	95+ years	1	1	Alcohol (liters per capita)
Larynx cancer	Female	15-19 years	95+ years	1	1	Alcohol (liters per capita)
Larynx cancer	Male	15-19 years	95+ years	1	2	Smoking Prevalence
Larynx cancer	Female	15-19 years	95+ years	1	2	Smoking Prevalence
Larynx cancer	Male	15-19 years	95+ years	1	2	Population Density (under 150 ppl/sqkm, proportion)
Larynx cancer	Female	15-19 years	95+ years	1	2	Population Density (under 150 ppl/sqkm, proportion)
Larynx cancer	Male	15-19 years	95+ years	1	2	Tobacco (cigarettes per capita)
Larynx cancer	Female	15-19 years	95+ years	1	2	Tobacco (cigarettes per capita)
Larynx cancer	Male	15-19 years	95+ years	-1	2	fruits adjusted (g)
Larynx cancer	Female	15-19 years	95+ years	-1	2	fruits adjusted (g)
Larynx cancer	Male	15-19 years	95+ years	-1	2	vegetables adjusted (g)
Larynx cancer	Female	15-19 years	95+ years	-1	2	vegetables adjusted (g)
Larynx cancer	Male	15-19 years	95+ years	1	2	Cumulative Cigarettes (10 Years)
Larynx cancer	Female	15-19 years	95+ years	1	2	Cumulative Cigarettes (10 Years)
Larynx cancer	Male	15-19 years	95+ years	1	2	Cumulative Cigarettes (15 Years)
Larynx cancer	Female	15-19 years	95+ years	1	2	Cumulative Cigarettes (15 Years)
Larynx cancer	Male	15-19 years	95+ years	1	2	Cumulative Cigarettes (20 Years)
Larynx cancer	Female	15-19 years	95+ years	1	2	Cumulative Cigarettes (20 Years)
Larynx cancer	Male	15-19 years	95+ years	1	2	Cumulative Cigarettes (5 Years)
Larynx cancer	Female	15-19 years	95+ years	1	2	Cumulative Cigarettes (5 Years)
Larynx cancer	Male	15-19 years	95+ years	1	1	Log-transformed SEV scalar: Larynx C
Larynx cancer	Female	15-19 years	95+ years	1	1	Log-transformed SEV scalar: Larynx C
Leukemia	Male	0-6 days	95+ years	0	3	LDI (I$ per capita)
Leukemia	Female	0-6 days	95+ years	0	3	LDI (I$ per capita)
Leukemia	Male	0-6 days	95+ years	0	3	Socio-demographic Index
Leukemia	Female	0-6 days	95+ years	0	3	Socio-demographic Index
Leukemia	Male	0-6 days	95+ years	-1	2	Healthcare access and quality index

Leukemia	Female	0-6 days	95+ years	-1	2	Healthcare access and quality index
Leukemia	Male	0-6 days	95+ years	-1	3	Education (years per capita)
Leukemia	Female	0-6 days	95+ years	-1	3	Education (years per capita)
Leukemia	Male	0-6 days	95+ years	1	2	Alcohol (liters per capita)
Leukemia	Female	0-6 days	95+ years	1	2	Alcohol (liters per capita)
Leukemia	Male	0-6 days	95+ years	1	2	Smoking Prevalence
Leukemia	Female	0-6 days	95+ years	1	2	Smoking Prevalence
Leukemia	Male	0-6 days	95+ years	1	2	Tobacco (cigarettes per capita)
Leukemia	Female	0-6 days	95+ years	1	2	Tobacco (cigarettes per capita)
Leukemia	Male	0-6 days	95+ years	1	2	Cumulative Cigarettes (10 Years)
Leukemia	Female	0-6 days	95+ years	1	2	Cumulative Cigarettes (10 Years)
Leukemia	Male	0-6 days	95+ years	1	2	Cumulative Cigarettes (15 Years)
Leukemia	Female	0-6 days	95+ years	1	2	Cumulative Cigarettes (15 Years)
Leukemia	Male	0-6 days	95+ years	1	2	Cumulative Cigarettes (20 Years)
Leukemia	Female	0-6 days	95+ years	1	2	Cumulative Cigarettes (20 Years)
Leukemia	Male	0-6 days	95+ years	1	2	Cumulative Cigarettes (5 Years)
Leukemia	Female	0-6 days	95+ years	1	2	Cumulative Cigarettes (5 Years)
Leukemia	Male	0-6 days	95+ years	1	1	Log-transformed SEV scalar: Leukemia
Leukemia	Female	0-6 days	95+ years	1	1	Log-transformed SEV scalar: Leukemia
Leukemia	Male	0-6 days	95+ years	1	1	Log-transformed age-standardized SEV scalar: Leukemia
Leukemia	Female	0-6 days	95+ years	1	1	Log-transformed age-standardized SEV scalar: Leukemia
Lip and oral cavity cancer	Male	15-19 years	95+ years	0	3	LDI (I$ per capita)
Lip and oral cavity cancer	Female	15-19 years	95+ years	0	3	LDI (I$ per capita)
Lip and oral cavity cancer	Male	15-19 years	95+ years	0	3	Socio-demographic Index
Lip and oral cavity cancer	Female	15-19 years	95+ years	0	3	Socio-demographic Index
Lip and oral cavity cancer	Male	15-19 years	95+ years	-1	2	Healthcare access and quality index
Lip and oral cavity cancer	Female	15-19 years	95+ years	-1	2	Healthcare access and quality index
Lip and oral cavity cancer	Male	15-19 years	95+ years	-1	3	Education (years per capita)
Lip and oral cavity cancer	Female	15-19 years	95+ years	-1	3	Education (years per capita)
Lip and oral cavity cancer	Male	15-19 years	95+ years	1	1	Alcohol (liters per capita)
Lip and oral cavity cancer	Female	15-19 years	95+ years	1	1	Alcohol (liters per capita)

© 2019 Global Burden of Disease Cancer Collaboration. JAMA Oncol.

Lip and oral cavity cancer	Male	15-19 years	95+ years	1	1	Smoking Prevalence
Lip and oral cavity cancer	Female	15-19 years	95+ years	1	1	Smoking Prevalence
Lip and oral cavity cancer	Female	15-19 years	95+ years	-1	2	Health System Access 2 (unitless)
Lip and oral cavity cancer	Male	15-19 years	95+ years	1	1	Tobacco (cigarettes per capita)
Lip and oral cavity cancer	Female	15-19 years	95+ years	1	1	Tobacco (cigarettes per capita)
Lip and oral cavity cancer	Male	15-19 years	95+ years	-1	2	fruits adjusted (g)
Lip and oral cavity cancer	Female	15-19 years	95+ years	-1	2	fruits adjusted (g)
Lip and oral cavity cancer	Male	15-19 years	95+ years	-1	1	vegetables adjusted (g)
Lip and oral cavity cancer	Female	15-19 years	95+ years	-1	1	vegetables adjusted (g)
Lip and oral cavity cancer	Male	15-19 years	95+ years	1	1	Cumulative Cigarettes (10 Years)
Lip and oral cavity cancer	Female	15-19 years	95+ years	1	1	Cumulative Cigarettes (10 Years)
Lip and oral cavity cancer	Male	15-19 years	95+ years	1	1	Cumulative Cigarettes (15 Years)
Lip and oral cavity cancer	Female	15-19 years	95+ years	1	1	Cumulative Cigarettes (15 Years)
Lip and oral cavity cancer	Male	15-19 years	95+ years	1	1	Cumulative Cigarettes (20 Years)
Lip and oral cavity cancer	Female	15-19 years	95+ years	1	1	Cumulative Cigarettes (20 Years)
Lip and oral cavity cancer	Male	15-19 years	95+ years	1	1	Cumulative Cigarettes (5 Years)
Lip and oral cavity cancer	Female	15-19 years	95+ years	1	1	Cumulative Cigarettes (5 Years)
Lip and oral cavity cancer	Male	15-19 years	95+ years	1	2	red meats adjusted (g)
Lip and oral cavity cancer	Female	15-19 years	95+ years	1	2	red meats adjusted (g)
Lip and oral cavity cancer	Male	15-19 years	95+ years	1	1	Log-transformed SEV scalar: Mouth C
Lip and oral cavity cancer	Female	15-19 years	95+ years	1	1	Log-transformed SEV scalar: Mouth C
Liver cancer	Male	5-9 years	95+ years	0	3	LDI (I$ per capita)
Liver cancer	Female	5-9 years	95+ years	0	3	LDI (I$ per capita)
Liver cancer	Male	5-9 years	95+ years	0	3	Socio-demographic Index
Liver cancer	Female	5-9 years	95+ years	0	3	Socio-demographic Index
Liver cancer	Male	5-9 years	95+ years	-1	2	Healthcare access and quality index
Liver cancer	Female	5-9 years	95+ years	-1	2	Healthcare access and quality index
Liver cancer	Male	5-9 years	95+ years	-1	3	Education (years per capita)
Liver cancer	Female	5-9 years	95+ years	-1	3	Education (years per capita)
Liver cancer	Male	5-9 years	95+ years	1	2	Mean BMI
Liver cancer	Female	5-9 years	95+ years	1	2	Mean BMI
Liver cancer	Male	5-9 years	95+ years	1	1	Alcohol (liters per capita)
Liver cancer	Female	5-9 years	95+ years	1	1	Alcohol (liters per capita)

© 2019 Global Burden of Disease Cancer Collaboration. JAMA Oncol.

Liver cancer	Male	5-9 years	95+ years	1	1	Hepatitis B (HBsAg) Seroprevalence
Liver cancer	Female	5-9 years	95+ years	1	1	Hepatitis B (HBsAg) Seroprevalence
Liver cancer	Male	5-9 years	95+ years	1	1	Hepatitis C (IgG) Seroprevalence
Liver cancer	Female	5-9 years	95+ years	1	1	Hepatitis C (IgG) Seroprevalence
Liver cancer	Male	5-9 years	95+ years	-1	2	Hepatitis B 3-dose coverage (proportion)
Liver cancer	Female	5-9 years	95+ years	-1	2	Hepatitis B 3-dose coverage (proportion)
Liver cancer	Male	5-9 years	95+ years	-1	2	Hepatitis B 3-dose coverage (proportion), lagged 5 years
Liver cancer	Female	5-9 years	95+ years	-1	2	Hepatitis B 3-dose coverage (proportion), lagged 5 years
Liver cancer	Male	5-9 years	95+ years	-1	2	Hepatitis B 3-dose coverage (proportion), lagged 10 years
Liver cancer	Female	5-9 years	95+ years	-1	2	Hepatitis B 3-dose coverage (proportion), lagged 10 years
Liver cancer	Male	5-9 years	95+ years	1	2	Tobacco (cigarettes per capita)
Liver cancer	Female	5-9 years	95+ years	1	2	Tobacco (cigarettes per capita)
Liver cancer	Male	5-9 years	95+ years	1	2	Cumulative Cigarettes (10 Years)
Liver cancer	Female	5-9 years	95+ years	1	2	Cumulative Cigarettes (10 Years)
Liver cancer	Male	5-9 years	95+ years	1	2	Cumulative Cigarettes (15 Years)
Liver cancer	Female	5-9 years	95+ years	1	2	Cumulative Cigarettes (15 Years)
Liver cancer	Male	5-9 years	95+ years	1	2	Cumulative Cigarettes (20 Years)
Liver cancer	Female	5-9 years	95+ years	1	2	Cumulative Cigarettes (20 Years)
Liver cancer	Male	5-9 years	95+ years	1	2	Cumulative Cigarettes (5 Years)
Liver cancer	Female	5-9 years	95+ years	1	2	Cumulative Cigarettes (5 Years)
Liver cancer	Male	5-9 years	95+ years	1	2	Diabetes Fasting Plasma Glucose (mmol/L)
Liver cancer	Female	5-9 years	95+ years	1	2	Diabetes Fasting Plasma Glucose (mmol/L)
Liver cancer	Male	5-9 years	95+ years	1	2	Diabetes Age-Standardized Prevalence (proportion)

 © 2019 Global Burden of Disease Cancer Collaboration. JAMA Oncol.

Liver cancer	Female	5-9 years	95+ years	1	2	Diabetes Age-Standardized Prevalence (proportion)
Liver cancer	Male	5-9 years	95+ years	1	1	Log-transformed SEV scalar: Liver C
Liver cancer	Female	5-9 years	95+ years	1	1	Log-transformed SEV scalar: Liver C
Liver cancer	Male	5-9 years	95+ years	1	2	red meats adjusted (g)
Liver cancer	Female	5-9 years	95+ years	1	2	red meats adjusted (g)
Liver cancer	Male	5-9 years	95+ years	1	1	HIV age-standardized prevalence
Liver cancer	Female	5-9 years	95+ years	1	1	HIV age-standardized prevalence
Malignant skin melanoma	Female	15-19 years	95+ years	0	3	LDI (I$ per capita)
Malignant skin melanoma	Male	15-19 years	95+ years	0	3	LDI (I$ per capita)
Malignant skin melanoma	Female	15-19 years	95+ years	0	3	Socio-demographic Index
Malignant skin melanoma	Male	15-19 years	95+ years	0	3	Socio-demographic Index
Malignant skin melanoma	Female	15-19 years	95+ years	-1	2	Healthcare access and quality index
Malignant skin melanoma	Male	15-19 years	95+ years	-1	2	Healthcare access and quality index
Malignant skin melanoma	Female	15-19 years	95+ years	-1	3	Education (years per capita)
Malignant skin melanoma	Male	15-19 years	95+ years	-1	3	Education (years per capita)
Malignant skin melanoma	Female	15-19 years	95+ years	1	1	Alcohol (liters per capita)
Malignant skin melanoma	Male	15-19 years	95+ years	1	2	Alcohol (liters per capita)
Malignant skin melanoma	Male	15-19 years	95+ years	1	1	Alcohol (liters per capita)
Malignant skin melanoma	Female	15-19 years	95+ years	0	2	Latitude Under 15 (proportion)
Malignant skin melanoma	Male	15-19 years	95+ years	0	2	Latitude Under 15 (proportion)
Malignant skin melanoma	Female	15-19 years	95+ years	0	2	Latitude 15 to 30 (proportion)
Malignant skin melanoma	Male	15-19 years	95+ years	0	2	Latitude 15 to 30 (proportion)
Malignant skin melanoma	Female	15-19 years	95+ years	-1	2	Latitude 30 to 45 (proportion)
Malignant skin melanoma	Male	15-19 years	95+ years	-1	2	Latitude 30 to 45 (proportion)
Malignant skin melanoma	Female	15-19 years	95+ years	-1	2	Latitude Over 45 (proportion)
Malignant skin melanoma	Male	15-19 years	95+ years	-1	2	Latitude Over 45 (proportion)
Malignant skin melanoma	Female	15-19 years	95+ years	-1	2	fruits adjusted (g)
Malignant skin melanoma	Male	15-19 years	95+ years	-1	2	fruits adjusted (g)
Malignant skin melanoma	Female	15-19 years	95+ years	-1	2	vegetables adjusted (g)
Malignant skin melanoma	Male	15-19 years	95+ years	-1	2	vegetables adjusted (g)
Mesothelioma	Female	15-19 years	95+ years	0	3	LDI (I$ per capita)
Mesothelioma	Male	15-19 years	95+ years	0	3	LDI (I$ per capita)

© 2019 Global Burden of Disease Cancer Collaboration. JAMA Oncol.

Mesothelioma	Female	15-19 years	95+ years	0	3	Socio-demographic Index
Mesothelioma	Male	15-19 years	95+ years	0	3	Socio-demographic Index
Mesothelioma	Female	15-19 years	95+ years	-1	2	Healthcare access and quality index
Mesothelioma	Male	15-19 years	95+ years	-1	2	Healthcare access and quality index
Mesothelioma	Female	15-19 years	95+ years	-1	3	Education (years per capita)
Mesothelioma	Male	15-19 years	95+ years	-1	3	Education (years per capita)
Mesothelioma	Female	15-19 years	95+ years	1	2	Population Density (over 1000 ppl/sqkm, proportion)
Mesothelioma	Male	15-19 years	95+ years	1	2	Population Density (over 1000 ppl/sqkm, proportion)
Mesothelioma	Female	15-19 years	95+ years	1	1	Indoor Air Pollution (All Cooking Fuels)
Mesothelioma	Male	15-19 years	95+ years	1	1	Indoor Air Pollution (All Cooking Fuels)
Mesothelioma	Female	15-19 years	95+ years	1	1	Smoking Prevalence
Mesothelioma	Male	15-19 years	95+ years	1	1	Smoking Prevalence
Mesothelioma	Female	15-19 years	95+ years	1	1	Cumulative Cigarettes (5 Years)
Mesothelioma	Male	15-19 years	95+ years	1	1	Cumulative Cigarettes (5 Years)
Mesothelioma	Female	15-19 years	95+ years	1	1	Asbestos consumption (metric tons per year per capita)
Mesothelioma	Male	15-19 years	95+ years	1	1	Asbestos consumption (metric tons per year per capita)
Mesothelioma	Female	15-19 years	95+ years	1	1	Asbestos production (binary)
Mesothelioma	Male	15-19 years	95+ years	1	1	Asbestos production (binary)
Mesothelioma	Female	15-19 years	95+ years	1	2	Asbestos production (kg) per capita
Mesothelioma	Male	15-19 years	95+ years	1	2	Asbestos production (kg) per capita
Mesothelioma	Female	15-19 years	95+ years	1	2	Gold production (binary)
Mesothelioma	Male	15-19 years	95+ years	1	2	Gold production (binary)
Mesothelioma	Female	15-19 years	95+ years	1	2	Gold production (kg) per capita
Mesothelioma	Male	15-19 years	95+ years	1	2	Gold production (kg) per capita
Multiple myeloma	Male	15-19 years	95+ years	0	3	LDI (I$ per capita)
Multiple myeloma	Female	15-19 years	95+ years	0	3	LDI (I$ per capita)
Multiple myeloma	Male	15-19 years	95+ years	-1	2	Sanitation (proportion with access)
Multiple myeloma	Female	15-19 years	95+ years	-1	2	Sanitation (proportion with access)

© 2019 Global Burden of Disease Cancer Collaboration. JAMA Oncol.

Multiple myeloma	Male	15-19 years	95+ years	-1	2	Improved Water Source (proportion with access)
Multiple myeloma	Female	15-19 years	95+ years	-1	2	Improved Water Source (proportion with access)
Multiple myeloma	Male	15-19 years	95+ years	0	3	Socio-demographic Index
Multiple myeloma	Female	15-19 years	95+ years	0	3	Socio-demographic Index
Multiple myeloma	Male	15-19 years	95+ years	-1	2	Healthcare access and quality index
Multiple myeloma	Female	15-19 years	95+ years	-1	2	Healthcare access and quality index
Multiple myeloma	Male	15-19 years	95+ years	-1	3	Education (years per capita)
Multiple myeloma	Female	15-19 years	95+ years	-1	3	Education (years per capita)
Multiple myeloma	Male	15-19 years	95+ years	1	2	Mean BMI
Multiple myeloma	Female	15-19 years	95+ years	1	2	Mean BMI
Multiple myeloma	Male	15-19 years	95+ years	1	1	Alcohol (liters per capita)
Multiple myeloma	Female	15-19 years	95+ years	1	1	Alcohol (liters per capita)
Multiple myeloma	Male	15-19 years	95+ years	1	1	Smoking Prevalence
Multiple myeloma	Female	15-19 years	95+ years	1	1	Smoking Prevalence
Multiple myeloma	Male	15-19 years	95+ years	1	1	Tobacco (cigarettes per capita)
Multiple myeloma	Female	15-19 years	95+ years	1	1	Tobacco (cigarettes per capita)
Multiple myeloma	Male	15-19 years	95+ years	-1	2	fruits adjusted (g)
Multiple myeloma	Female	15-19 years	95+ years	-1	2	fruits adjusted (g)
Multiple myeloma	Male	15-19 years	95+ years	-1	2	vegetables adjusted (g)
Multiple myeloma	Female	15-19 years	95+ years	-1	2	vegetables adjusted (g)
Multiple myeloma	Male	15-19 years	95+ years	1	2	red meats adjusted (g)
Multiple myeloma	Female	15-19 years	95+ years	1	2	red meats adjusted (g)
Myelodysplastic, myeloproliferative, and other hematopoietic neoplasms	Male	0-6 days	95+ years	0	3	LDI (I$ per capita)
Myelodysplastic, myeloproliferative, and other hematopoietic neoplasms	Female	0-6 days	95+ years	0	3	LDI (I$ per capita)
Myelodysplastic, myeloproliferative, and other hematopoietic neoplasms	Male	0-6 days	95+ years	0	3	Socio-demographic Index
Myelodysplastic, myeloproliferative, and other hematopoietic neoplasms	Female	0-6 days	95+ years	0	3	Socio-demographic Index

© 2019 Global Burden of Disease Cancer Collaboration. JAMA Oncol.

Myelodysplastic, myeloproliferative, and other hematopoietic neoplasms	Male	0-6 days	95+ years	-1	2	Healthcare access and quality index
Myelodysplastic, myeloproliferative, and other hematopoietic neoplasms	Female	0-6 days	95+ years	-1	2	Healthcare access and quality index
Myelodysplastic, myeloproliferative, and other hematopoietic neoplasms	Male	0-6 days	95+ years	-1	3	Education (years per capita)
Myelodysplastic, myeloproliferative, and other hematopoietic neoplasms	Female	0-6 days	95+ years	-1	3	Education (years per capita)
Myelodysplastic, myeloproliferative, and other hematopoietic neoplasms	Male	0-6 days	95+ years	1	2	Alcohol (liters per capita)
Myelodysplastic, myeloproliferative, and other hematopoietic neoplasms	Female	0-6 days	95+ years	1	2	Alcohol (liters per capita)
Myelodysplastic, myeloproliferative, and other hematopoietic neoplasms	Male	0-6 days	95+ years	1	2	Smoking Prevalence
Myelodysplastic, myeloproliferative, and other hematopoietic neoplasms	Female	0-6 days	95+ years	1	2	Smoking Prevalence
Myelodysplastic, myeloproliferative, and other hematopoietic neoplasms	Male	0-6 days	95+ years	1	2	Tobacco (cigarettes per capita)
Myelodysplastic, myeloproliferative, and other hematopoietic neoplasms	Female	0-6 days	95+ years	1	2	Tobacco (cigarettes per capita)
Myelodysplastic, myeloproliferative, and other hematopoietic neoplasms	Male	0-6 days	95+ years	1	2	Cumulative Cigarettes (10 Years)
Myelodysplastic, myeloproliferative, and other hematopoietic neoplasms	Female	0-6 days	95+ years	1	2	Cumulative Cigarettes (10 Years)
Myelodysplastic, myeloproliferative, and other hematopoietic neoplasms	Male	0-6 days	95+ years	1	2	Cumulative Cigarettes (15 Years)
Myelodysplastic, myeloproliferative, and other hematopoietic neoplasms	Female	0-6 days	95+ years	1	2	Cumulative Cigarettes (15 Years)
Myelodysplastic, myeloproliferative, and other hematopoietic neoplasms	Male	0-6 days	95+ years	1	2	Cumulative Cigarettes (20 Years)
Myelodysplastic, myeloproliferative, and other hematopoietic neoplasms	Female	0-6 days	95+ years	1	2	Cumulative Cigarettes (20 Years)
Myelodysplastic, myeloproliferative, and other hematopoietic neoplasms	Male	0-6 days	95+ years	1	2	Cumulative Cigarettes (5 Years)

 © 2019 Global Burden of Disease Cancer Collaboration. JAMA Oncol.

Myelodysplastic, myeloproliferative, and other hematopoietic neoplasms	Female	0-6 days	95+ years	1	2	Cumulative Cigarettes (5 Years)
Myelodysplastic, myeloproliferative, and other hematopoietic neoplasms	Male	0-6 days	95+ years	1	1	Log-transformed SEV scalar: Leukemia
Myelodysplastic, myeloproliferative, and other hematopoietic neoplasms	Female	0-6 days	95+ years	1	1	Log-transformed SEV scalar: Leukemia
Myelodysplastic, myeloproliferative, and other hematopoietic neoplasms	Male	0-6 days	95+ years	1	1	Log-transformed age-standardized SEV scalar: Leukemia
Myelodysplastic, myeloproliferative, and other hematopoietic neoplasms	Female	0-6 days	95+ years	1	1	Log-transformed age-standardized SEV scalar: Leukemia
Nasopharynx cancer	Male	5-9 years	95+ years	0	3	LDI (I$ per capita)
Nasopharynx cancer	Female	5-9 years	95+ years	0	3	LDI (I$ per capita)
Nasopharynx cancer	Male	5-9 years	95+ years	0	3	Socio-demographic Index
Nasopharynx cancer	Female	5-9 years	95+ years	0	3	Socio-demographic Index
Nasopharynx cancer	Male	5-9 years	95+ years	-1	2	Healthcare access and quality index
Nasopharynx cancer	Female	5-9 years	95+ years	-1	2	Healthcare access and quality index
Nasopharynx cancer	Male	5-9 years	95+ years	-1	3	Education (years per capita)
Nasopharynx cancer	Female	5-9 years	95+ years	-1	3	Education (years per capita)
Nasopharynx cancer	Male	5-9 years	95+ years	1	2	Population Density (over 1000 ppl/sqkm, proportion)
Nasopharynx cancer	Female	5-9 years	95+ years	1	2	Population Density (over 1000 ppl/sqkm, proportion)
Nasopharynx cancer	Male	5-9 years	95+ years	1	1	Alcohol (liters per capita)
Nasopharynx cancer	Female	5-9 years	95+ years	1	1	Alcohol (liters per capita)
Nasopharynx cancer	Male	5-9 years	95+ years	1	1	Smoking Prevalence
Nasopharynx cancer	Female	5-9 years	95+ years	1	1	Smoking Prevalence
Nasopharynx cancer	Male	5-9 years	95+ years	1	2	Population Density (under 150 ppl/sqkm, proportion)
Nasopharynx cancer	Female	5-9 years	95+ years	1	2	Population Density (under 150 ppl/sqkm, proportion)
Nasopharynx cancer	Male	5-9 years	95+ years	1	1	Tobacco (cigarettes per capita)
Nasopharynx cancer	Female	5-9 years	95+ years	1	1	Tobacco (cigarettes per capita)
Nasopharynx cancer	Male	5-9 years	95+ years	-1	2	fruits adjusted (g)

 © 2019 Global Burden of Disease Cancer Collaboration. JAMA Oncol.

Nasopharynx cancer	Female	5-9 years	95+ years	-1	2	fruits adjusted (g)
Nasopharynx cancer	Male	5-9 years	95+ years	-1	2	vegetables adjusted (g)
Nasopharynx cancer	Female	5-9 years	95+ years	-1	2	vegetables adjusted (g)
Nasopharynx cancer	Male	5-9 years	95+ years	1	1	Cumulative Cigarettes (10 Years)
Nasopharynx cancer	Female	5-9 years	95+ years	1	1	Cumulative Cigarettes (10 Years)
Nasopharynx cancer	Male	5-9 years	95+ years	1	1	Cumulative Cigarettes (15 Years)
Nasopharynx cancer	Female	5-9 years	95+ years	1	1	Cumulative Cigarettes (15 Years)
Nasopharynx cancer	Male	5-9 years	95+ years	1	1	Cumulative Cigarettes (20 Years)
Nasopharynx cancer	Female	5-9 years	95+ years	1	1	Cumulative Cigarettes (20 Years)
Nasopharynx cancer	Male	5-9 years	95+ years	1	1	Cumulative Cigarettes (5 Years)
Nasopharynx cancer	Female	5-9 years	95+ years	1	1	Cumulative Cigarettes (5 Years)
Nasopharynx cancer	Male	5-9 years	95+ years	1	1	Log-transformed SEV scalar: Nasoph C
Nasopharynx cancer	Female	5-9 years	95+ years	1	1	Log-transformed SEV scalar: Nasoph C
Non-Hodgkin lymphoma	Male	0-6 days	95+ years	0	3	LDI (I$ per capita)
Non-Hodgkin lymphoma	Female	0-6 days	95+ years	0	3	LDI (I$ per capita)
Non-Hodgkin lymphoma	Male	0-6 days	95+ years	0	3	Socio-demographic Index
Non-Hodgkin lymphoma	Female	0-6 days	95+ years	0	3	Socio-demographic Index
Non-Hodgkin lymphoma	Male	0-6 days	95+ years	-1	2	Healthcare access and quality index
Non-Hodgkin lymphoma	Female	0-6 days	95+ years	-1	2	Healthcare access and quality index
Non-Hodgkin lymphoma	Male	0-6 days	95+ years	1	2	Alcohol (liters per capita)
Non-Hodgkin lymphoma	Female	0-6 days	95+ years	1	2	Alcohol (liters per capita)
Non-Hodgkin lymphoma	Male	0-6 days	95+ years	1	2	Smoking Prevalence
Non-Hodgkin lymphoma	Female	0-6 days	95+ years	1	2	Smoking Prevalence
Non-Hodgkin lymphoma	Female	0-6 days	95+ years	0	3	Total Fertility Rate
Non-Hodgkin lymphoma	Male	0-6 days	95+ years	1	2	Cumulative Cigarettes (10 Years)
Non-Hodgkin lymphoma	Female	0-6 days	95+ years	1	2	Cumulative Cigarettes (10 Years)
Non-Hodgkin lymphoma	Male	0-6 days	95+ years	1	2	Cumulative Cigarettes (15 Years)
Non-Hodgkin lymphoma	Female	0-6 days	95+ years	1	2	Cumulative Cigarettes (15 Years)
Non-Hodgkin lymphoma	Male	0-6 days	95+ years	1	2	Cumulative Cigarettes (20 Years)
Non-Hodgkin lymphoma	Female	0-6 days	95+ years	1	2	Cumulative Cigarettes (20 Years)
Non-Hodgkin lymphoma	Male	0-6 days	95+ years	1	2	Cumulative Cigarettes (5 Years)
Non-Hodgkin lymphoma	Female	0-6 days	95+ years	1	2	Cumulative Cigarettes (5 Years)
Non-melanoma skin cancer	Male	15-19 years	95+ years	0	3	LDI (I$ per capita)

© 2019 Global Burden of Disease Cancer Collaboration. JAMA Oncol.

Non-melanoma skin cancer	Female	15-19 years	95+ years	0	3	LDI (I$ per capita)
Non-melanoma skin cancer	Male	15-19 years	95+ years	0	3	Socio-demographic Index
Non-melanoma skin cancer	Female	15-19 years	95+ years	0	3	Socio-demographic Index
Non-melanoma skin cancer	Male	15-19 years	95+ years	-1	2	Healthcare access and quality index
Non-melanoma skin cancer	Female	15-19 years	95+ years	-1	2	Healthcare access and quality index
Non-melanoma skin cancer	Male	15-19 years	95+ years	-1	3	Education (years per capita)
Non-melanoma skin cancer	Female	15-19 years	95+ years	-1	3	Education (years per capita)
Non-melanoma skin cancer	Male	15-19 years	95+ years	1	1	Smoking Prevalence
Non-melanoma skin cancer	Female	15-19 years	95+ years	1	1	Smoking Prevalence
Non-melanoma skin cancer	Male	15-19 years	95+ years	1	1	Cumulative Cigarettes (10 Years)
Non-melanoma skin cancer	Female	15-19 years	95+ years	1	1	Cumulative Cigarettes (10 Years)
Non-melanoma skin cancer	Male	15-19 years	95+ years	1	1	Cumulative Cigarettes (15 Years)
Non-melanoma skin cancer	Female	15-19 years	95+ years	1	1	Cumulative Cigarettes (15 Years)
Non-melanoma skin cancer	Male	15-19 years	95+ years	1	1	Cumulative Cigarettes (5 Years)
Non-melanoma skin cancer	Female	15-19 years	95+ years	1	1	Cumulative Cigarettes (5 Years)
Non-melanoma skin cancer	Male	15-19 years	95+ years	0	2	Average latitude
Non-melanoma skin cancer	Female	15-19 years	95+ years	0	2	Average latitude
Non-melanoma skin cancer (squamous-cell carcinoma)	Male	28-364 days	95+ years	0	3	LDI (I$ per capita)
Non-melanoma skin cancer (squamous-cell carcinoma)	Female	28-364 days	95+ years	0	3	LDI (I$ per capita)
Non-melanoma skin cancer (squamous-cell carcinoma)	Male	28-364 days	95+ years	0	3	Socio-demographic Index
Non-melanoma skin cancer (squamous-cell carcinoma)	Female	28-364 days	95+ years	0	3	Socio-demographic Index
Non-melanoma skin cancer (squamous-cell carcinoma)	Male	28-364 days	95+ years	-1	2	Healthcare access and quality index
Non-melanoma skin cancer (squamous-cell carcinoma)	Female	28-364 days	95+ years	-1	2	Healthcare access and quality index
Non-melanoma skin cancer (squamous-cell carcinoma)	Male	28-364 days	95+ years	-1	3	Education (years per capita)
Non-melanoma skin cancer (squamous-cell carcinoma)	Female	28-364 days	95+ years	-1	3	Education (years per capita)

© 2019 Global Burden of Disease Cancer Collaboration. JAMA Oncol.

Non-melanoma skin cancer (squamous-cell carcinoma)	Male	28-364 days	95+ years	1	1	Smoking Prevalence
Non-melanoma skin cancer (squamous-cell carcinoma)	Female	28-364 days	95+ years	1	1	Smoking Prevalence
Non-melanoma skin cancer (squamous-cell carcinoma)	Male	28-364 days	95+ years	1	1	Cumulative Cigarettes (10 Years)
Non-melanoma skin cancer (squamous-cell carcinoma)	Female	28-364 days	95+ years	1	1	Cumulative Cigarettes (10 Years)
Non-melanoma skin cancer (squamous-cell carcinoma)	Male	28-364 days	95+ years	1	1	Cumulative Cigarettes (15 Years)
Non-melanoma skin cancer (squamous-cell carcinoma)	Female	28-364 days	95+ years	1	1	Cumulative Cigarettes (15 Years)
Non-melanoma skin cancer (squamous-cell carcinoma)	Male	28-364 days	95+ years	1	1	Cumulative Cigarettes (5 Years)
Non-melanoma skin cancer (squamous-cell carcinoma)	Female	28-364 days	95+ years	1	1	Cumulative Cigarettes (5 Years)
Non-melanoma skin cancer (squamous-cell carcinoma)	Male	28-364 days	95+ years	0	2	Average latitude
Non-melanoma skin cancer (squamous-cell carcinoma)	Female	28-364 days	95+ years	0	2	Average latitude
Other leukemia	Male	0-6 days	95+ years	0	3	LDI (I$ per capita)
Other leukemia	Female	0-6 days	95+ years	0	3	LDI (I$ per capita)
Other leukemia	Male	0-6 days	95+ years	0	3	Socio-demographic Index
Other leukemia	Female	0-6 days	95+ years	0	3	Socio-demographic Index
Other leukemia	Male	0-6 days	95+ years	-1	2	Healthcare access and quality index
Other leukemia	Female	0-6 days	95+ years	-1	2	Healthcare access and quality index
Other leukemia	Male	0-6 days	95+ years	-1	3	Education (years per capita)
Other leukemia	Female	0-6 days	95+ years	-1	3	Education (years per capita)
Other leukemia	Male	0-6 days	95+ years	1	2	Alcohol (liters per capita)
Other leukemia	Female	0-6 days	95+ years	1	2	Alcohol (liters per capita)
Other leukemia	Male	0-6 days	95+ years	1	2	Smoking Prevalence
Other leukemia	Female	0-6 days	95+ years	1	2	Smoking Prevalence
Other leukemia	Male	0-6 days	95+ years	1	2	Tobacco (cigarettes per capita)

© 2019 Global Burden of Disease Cancer Collaboration. JAMA Oncol.

Other leukemia	Female	0-6 days	95+ years	1	2	Tobacco (cigarettes per capita)
Other leukemia	Male	0-6 days	95+ years	1	2	Cumulative Cigarettes (10 Years)
Other leukemia	Female	0-6 days	95+ years	1	2	Cumulative Cigarettes (10 Years)
Other leukemia	Male	0-6 days	95+ years	1	2	Cumulative Cigarettes (15 Years)
Other leukemia	Female	0-6 days	95+ years	1	2	Cumulative Cigarettes (15 Years)
Other leukemia	Male	0-6 days	95+ years	1	2	Cumulative Cigarettes (20 Years)
Other leukemia	Female	0-6 days	95+ years	1	2	Cumulative Cigarettes (20 Years)
Other leukemia	Male	0-6 days	95+ years	1	2	Cumulative Cigarettes (5 Years)
Other leukemia	Female	0-6 days	95+ years	1	2	Cumulative Cigarettes (5 Years)
Other leukemia	Male	0-6 days	95+ years	1	1	Log-transformed SEV scalar: Leukemia
Other leukemia	Female	0-6 days	95+ years	1	1	Log-transformed SEV scalar: Leukemia
Other leukemia	Male	0-6 days	95+ years	1	1	Log-transformed age-standardized SEV scalar: Leukemia
Other leukemia	Female	0-6 days	95+ years	1	1	Log-transformed age-standardized SEV scalar: Leukemia
Other malignant neoplasms	Male	0-6 days	95+ years	0	3	LDI (I$ per capita)
Other malignant neoplasms	Female	0-6 days	95+ years	0	3	LDI (I$ per capita)
Other malignant neoplasms	Male	0-6 days	95+ years	0	3	Socio-demographic Index
Other malignant neoplasms	Female	0-6 days	95+ years	0	3	Socio-demographic Index
Other malignant neoplasms	Male	0-6 days	95+ years	-1	2	Healthcare access and quality index
Other malignant neoplasms	Female	0-6 days	95+ years	-1	2	Healthcare access and quality index
Other malignant neoplasms	Male	0-6 days	95+ years	-1	3	Education (years per capita)
Other malignant neoplasms	Female	0-6 days	95+ years	-1	3	Education (years per capita)
Other malignant neoplasms	Male	0-6 days	95+ years	1	1	Smoking Prevalence
Other malignant neoplasms	Female	0-6 days	95+ years	1	1	Smoking Prevalence
Other malignant neoplasms	Male	0-6 days	95+ years	1	1	Tobacco (cigarettes per capita)
Other malignant neoplasms	Female	0-6 days	95+ years	1	1	Tobacco (cigarettes per capita)
Other malignant neoplasms	Male	0-6 days	95+ years	-1	2	fruits adjusted (g)
Other malignant neoplasms	Female	0-6 days	95+ years	-1	2	fruits adjusted (g)
Other malignant neoplasms	Male	0-6 days	95+ years	-1	2	vegetables adjusted (g)
Other malignant neoplasms	Female	0-6 days	95+ years	-1	2	vegetables adjusted (g)
Other malignant neoplasms	Male	0-6 days	95+ years	-1	2	nuts seeds adjusted (g)
Other malignant neoplasms	Female	0-6 days	95+ years	-1	2	nuts seeds adjusted (g)

© 2019 Global Burden of Disease Cancer Collaboration. JAMA Oncol.

Other malignant neoplasms	Male	0-6 days	95+ years	-1	2	pufa adjusted(percent)
Other malignant neoplasms	Female	0-6 days	95+ years	-1	2	pufa adjusted(percent)
Other pharynx cancer	Male	15-19 years	95+ years	0	3	LDI (I$ per capita)
Other pharynx cancer	Female	15-19 years	95+ years	0	3	LDI (I$ per capita)
Other pharynx cancer	Male	15-19 years	95+ years	0	3	Socio-demographic Index
Other pharynx cancer	Female	15-19 years	95+ years	0	3	Socio-demographic Index
Other pharynx cancer	Male	15-19 years	95+ years	-1	2	Healthcare access and quality index
Other pharynx cancer	Female	15-19 years	95+ years	-1	2	Healthcare access and quality index
Other pharynx cancer	Male	15-19 years	95+ years	-1	3	Education (years per capita)
Other pharynx cancer	Female	15-19 years	95+ years	-1	3	Education (years per capita)
Other pharynx cancer	Male	15-19 years	95+ years	1	2	Population Density (over 1000 ppl/sqkm, proportion)
Other pharynx cancer	Female	15-19 years	95+ years	1	2	Population Density (over 1000 ppl/sqkm, proportion)
Other pharynx cancer	Male	15-19 years	95+ years	1	1	Alcohol (liters per capita)
Other pharynx cancer	Female	15-19 years	95+ years	1	1	Alcohol (liters per capita)
Other pharynx cancer	Male	15-19 years	95+ years	1	1	Smoking Prevalence
Other pharynx cancer	Female	15-19 years	95+ years	1	1	Smoking Prevalence
Other pharynx cancer	Male	15-19 years	95+ years	1	2	Population Density (under 150 ppl/sqkm, proportion)
Other pharynx cancer	Female	15-19 years	95+ years	1	2	Population Density (under 150 ppl/sqkm, proportion)
Other pharynx cancer	Male	15-19 years	95+ years	-1	2	fruits adjusted (g)
Other pharynx cancer	Female	15-19 years	95+ years	-1	2	fruits adjusted (g)
Other pharynx cancer	Male	15-19 years	95+ years	-1	2	vegetables adjusted (g)
Other pharynx cancer	Female	15-19 years	95+ years	-1	2	vegetables adjusted (g)
Other pharynx cancer	Male	15-19 years	95+ years	1	2	Cumulative Cigarettes (5 Years)
Other pharynx cancer	Female	15-19 years	95+ years	1	2	Cumulative Cigarettes (5 Years)
Other pharynx cancer	Male	15-19 years	95+ years	1	1	Log-transformed SEV scalar: Oth Phar C
Other pharynx cancer	Female	15-19 years	95+ years	1	1	Log-transformed SEV scalar: Oth Phar C
Ovarian cancer	Female	15-19 years	95+ years	0	3	LDI (I$ per capita)

© 2019 Global Burden of Disease Cancer Collaboration. JAMA Oncol.

Ovarian cancer	Female	15-19 years	95+ years	0	3	Socio-demographic Index
Ovarian cancer	Female	15-19 years	95+ years	-1	2	Healthcare access and quality index
Ovarian cancer	Female	15-19 years	95+ years	-1	3	Education (years per capita)
Ovarian cancer	Female	15-19 years	95+ years	1	2	Mean BMI
Ovarian cancer	Female	15-19 years	95+ years	1	1	Alcohol (liters per capita)
Ovarian cancer	Female	15-19 years	95+ years	1	2	Smoking Prevalence
Ovarian cancer	Female	15-19 years	95+ years	0	2	Total Fertility Rate
Ovarian cancer	Female	15-19 years	95+ years	1	2	energy unadjusted(kcal)
Ovarian cancer	Female	15-19 years	95+ years	1	1	Tobacco (cigarettes per capita)
Ovarian cancer	Female	15-19 years	95+ years	-1	2	fruits adjusted (g)
Ovarian cancer	Female	15-19 years	95+ years	-1	2	vegetables adjusted (g)
Ovarian cancer	Female	15-19 years	95+ years	1	1	Cumulative Cigarettes (10 Years)
Ovarian cancer	Female	15-19 years	95+ years	1	1	Cumulative Cigarettes (15 Years)
Ovarian cancer	Female	15-19 years	95+ years	1	1	Cumulative Cigarettes (20 Years)
Ovarian cancer	Female	15-19 years	95+ years	1	1	Cumulative Cigarettes (5 Years)
Ovarian cancer	Female	15-19 years	95+ years	1	2	Diabetes Age-Standardized Prevalence (proportion)
Ovarian cancer	Female	15-19 years	95+ years	-1	1	Contraception (Modern) Prevalence (proportion)
Ovarian cancer	Female	15-19 years	95+ years	1	1	Log-transformed SEV scalar: Ovary C
Pancreatic cancer	Male	15-19 years	95+ years	0	3	LDI (I$ per capita)
Pancreatic cancer	Female	15-19 years	95+ years	0	3	LDI (I$ per capita)
Pancreatic cancer	Male	15-19 years	95+ years	0	3	Socio-demographic Index
Pancreatic cancer	Female	15-19 years	95+ years	0	3	Socio-demographic Index
Pancreatic cancer	Male	15-19 years	95+ years	-1	2	Healthcare access and quality index
Pancreatic cancer	Female	15-19 years	95+ years	-1	2	Healthcare access and quality index
Pancreatic cancer	Male	15-19 years	95+ years	-1	3	Education (years per capita)
Pancreatic cancer	Female	15-19 years	95+ years	-1	3	Education (years per capita)
Pancreatic cancer	Male	15-19 years	95+ years	1	1	Mean BMI
Pancreatic cancer	Female	15-19 years	95+ years	1	1	Mean BMI
Pancreatic cancer	Male	15-19 years	95+ years	1	1	Alcohol (liters per capita)
Pancreatic cancer	Female	15-19 years	95+ years	1	1	Alcohol (liters per capita)
Pancreatic cancer	Male	15-19 years	95+ years	1	1	Smoking Prevalence

© 2019 Global Burden of Disease Cancer Collaboration. JAMA Oncol.

Pancreatic cancer	Female	15-19 years	95+ years	1	1	Smoking Prevalence
Pancreatic cancer	Male	15-19 years	95+ years	1	2	energy unadjusted(kcal)
Pancreatic cancer	Female	15-19 years	95+ years	1	2	energy unadjusted(kcal)
Pancreatic cancer	Male	15-19 years	95+ years	1	1	Tobacco (cigarettes per capita)
Pancreatic cancer	Female	15-19 years	95+ years	1	1	Tobacco (cigarettes per capita)
Pancreatic cancer	Male	15-19 years	95+ years	-1	2	fruits adjusted (g)
Pancreatic cancer	Female	15-19 years	95+ years	-1	2	fruits adjusted (g)
Pancreatic cancer	Male	15-19 years	95+ years	-1	2	vegetables adjusted (g)
Pancreatic cancer	Female	15-19 years	95+ years	-1	2	vegetables adjusted (g)
Pancreatic cancer	Male	15-19 years	95+ years	1	1	Cumulative Cigarettes (10 Years)
Pancreatic cancer	Female	15-19 years	95+ years	1	1	Cumulative Cigarettes (10 Years)
Pancreatic cancer	Male	15-19 years	95+ years	1	1	Cumulative Cigarettes (15 Years)
Pancreatic cancer	Female	15-19 years	95+ years	1	1	Cumulative Cigarettes (15 Years)
Pancreatic cancer	Male	15-19 years	95+ years	1	1	Cumulative Cigarettes (20 Years)
Pancreatic cancer	Female	15-19 years	95+ years	1	1	Cumulative Cigarettes (20 Years)
Pancreatic cancer	Male	15-19 years	95+ years	1	1	Cumulative Cigarettes (5 Years)
Pancreatic cancer	Female	15-19 years	95+ years	1	1	Cumulative Cigarettes (5 Years)
Pancreatic cancer	Male	15-19 years	95+ years	1	2	Diabetes Age-Standardized Prevalence (proportion)
Pancreatic cancer	Female	15-19 years	95+ years	1	2	Diabetes Age-Standardized Prevalence (proportion)
Pancreatic cancer	Male	15-19 years	95+ years	1	2	red meats adjusted (g)
Pancreatic cancer	Female	15-19 years	95+ years	1	2	red meats adjusted (g)
Pancreatic cancer	Male	15-19 years	95+ years	1	1	Log-transformed SEV scalar: Pancreas C
Pancreatic cancer	Female	15-19 years	95+ years	1	1	Log-transformed SEV scalar: Pancreas C
Prostate cancer	Male	15-19 years	95+ years	0	3	LDI (I$ per capita)
Prostate cancer	Male	15-19 years	95+ years	0	3	Socio-demographic Index
Prostate cancer	Male	15-19 years	95+ years	-1	2	Healthcare access and quality index
Prostate cancer	Male	15-19 years	95+ years	-1	3	Education (years per capita)
Prostate cancer	Male	15-19 years	95+ years	1	1	Log-transformed SEV scalar: Prostate C
Stomach cancer	Female	15-19 years	95+ years	0	3	LDI (I$ per capita)

© 2019 Global Burden of Disease Cancer Collaboration. JAMA Oncol.

Stomach cancer	Male	15-19 years	95+ years	0	3	LDI (I$ per capita)
Stomach cancer	Female	15-19 years	95+ years	-1	2	Sanitation (proportion with access)
Stomach cancer	Male	15-19 years	95+ years	-1	2	Sanitation (proportion with access)
Stomach cancer	Female	15-19 years	95+ years	-1	2	Improved Water Source (proportion with access)
Stomach cancer	Male	15-19 years	95+ years	-1	2	Improved Water Source (proportion with access)
Stomach cancer	Female	15-19 years	95+ years	1	1	SEV unsafe water
Stomach cancer	Male	15-19 years	95+ years	1	1	SEV unsafe water
Stomach cancer	Female	15-19 years	95+ years	1	1	SEV unsafe sanitation
Stomach cancer	Male	15-19 years	95+ years	1	1	SEV unsafe sanitation
Stomach cancer	Female	15-19 years	95+ years	0	3	Socio-demographic Index
Stomach cancer	Male	15-19 years	95+ years	0	3	Socio-demographic Index
Stomach cancer	Female	15-19 years	95+ years	-1	2	Healthcare access and quality index
Stomach cancer	Male	15-19 years	95+ years	-1	2	Healthcare access and quality index
Stomach cancer	Female	15-19 years	95+ years	-1	3	Education (years per capita)
Stomach cancer	Male	15-19 years	95+ years	-1	3	Education (years per capita)
Stomach cancer	Female	15-19 years	95+ years	1	2	Mean BMI
Stomach cancer	Male	15-19 years	95+ years	1	2	Mean BMI
Stomach cancer	Female	15-19 years	95+ years	1	1	Smoking Prevalence
Stomach cancer	Male	15-19 years	95+ years	1	1	Smoking Prevalence
Stomach cancer	Female	15-19 years	95+ years	1	1	Tobacco (cigarettes per capita)
Stomach cancer	Male	15-19 years	95+ years	1	1	Tobacco (cigarettes per capita)
Stomach cancer	Female	15-19 years	95+ years	-1	2	fruits adjusted (g)
Stomach cancer	Male	15-19 years	95+ years	-1	2	fruits adjusted (g)
Stomach cancer	Female	15-19 years	95+ years	-1	2	vegetables adjusted (g)
Stomach cancer	Male	15-19 years	95+ years	-1	2	vegetables adjusted (g)
Stomach cancer	Female	15-19 years	95+ years	1	1	Cumulative Cigarettes (10 Years)
Stomach cancer	Male	15-19 years	95+ years	1	1	Cumulative Cigarettes (10 Years)
Stomach cancer	Female	15-19 years	95+ years	1	1	Cumulative Cigarettes (15 Years)
Stomach cancer	Male	15-19 years	95+ years	1	1	Cumulative Cigarettes (15 Years)
Stomach cancer	Female	15-19 years	95+ years	1	1	Cumulative Cigarettes (20 Years)
Stomach cancer	Male	15-19 years	95+ years	1	1	Cumulative Cigarettes (20 Years)

© 2019 Global Burden of Disease Cancer Collaboration. JAMA Oncol.

Stomach cancer	Female	15-19 years	95+ years	1	1	Cumulative Cigarettes (5 Years)
Stomach cancer	Male	15-19 years	95+ years	1	1	Cumulative Cigarettes (5 Years)
Stomach cancer	Female	15-19 years	95+ years	1	1	Log-transformed SEV scalar: Stomach C
Stomach cancer	Male	15-19 years	95+ years	1	1	Log-transformed SEV scalar: Stomach C
Stomach cancer	Female	15-19 years	95+ years	1	1	Diet high in sodium
Stomach cancer	Male	15-19 years	95+ years	1	1	Diet high in sodium
Testicular cancer	Male	15-19 years	95+ years	0	3	LDI (I$ per capita)
Testicular cancer	Male	15-19 years	95+ years	0	3	Socio-demographic Index
Testicular cancer	Male	15-19 years	95+ years	-1	2	Healthcare access and quality index
Testicular cancer	Male	15-19 years	95+ years	-1	3	Education (years per capita)
Testicular cancer	Male	15-19 years	95+ years	1	2	Smoking Prevalence
Testicular cancer	Male	15-19 years	95+ years	1	2	Tobacco (cigarettes per capita)
Testicular cancer	Male	15-19 years	95+ years	-1	2	fruits adjusted (g)
Testicular cancer	Male	15-19 years	95+ years	-1	2	vegetables adjusted (g)
Testicular cancer	Male	15-19 years	95+ years	1	2	Cumulative Cigarettes (10 Years)
Testicular cancer	Male	15-19 years	95+ years	1	2	Cumulative Cigarettes (15 Years)
Testicular cancer	Male	15-19 years	95+ years	1	2	Cumulative Cigarettes (20 Years)
Testicular cancer	Male	15-19 years	95+ years	1	2	Cumulative Cigarettes (5 Years)
Thyroid cancer	Male	10-14 years	95+ years	0	3	LDI (I$ per capita)
Thyroid cancer	Female	10-14 years	95+ years	0	3	LDI (I$ per capita)
Thyroid cancer	Male	10-14 years	95+ years	-1	2	Sanitation (proportion with access)
Thyroid cancer	Female	10-14 years	95+ years	-1	2	Sanitation (proportion with access)
Thyroid cancer	Male	10-14 years	95+ years	-1	2	Improved Water Source (proportion with access)
Thyroid cancer	Female	10-14 years	95+ years	-1	2	Improved Water Source (proportion with access)
Thyroid cancer	Male	10-14 years	95+ years	0	3	Socio-demographic Index
Thyroid cancer	Female	10-14 years	95+ years	0	3	Socio-demographic Index
Thyroid cancer	Male	10-14 years	95+ years	-1	2	Healthcare access and quality index
Thyroid cancer	Female	10-14 years	95+ years	-1	2	Healthcare access and quality index
Thyroid cancer	Male	10-14 years	95+ years	-1	3	Education (years per capita)
Thyroid cancer	Female	10-14 years	95+ years	-1	3	Education (years per capita)
Thyroid cancer	Male	10-14 years	95+ years	1	2	Mean BMI

 © 2019 Global Burden of Disease Cancer Collaboration. JAMA Oncol.

Thyroid cancer	Female	10-14 years	95+ years	1	2	Mean BMI
Thyroid cancer	Male	10-14 years	95+ years	1	1	Alcohol (liters per capita)
Thyroid cancer	Female	10-14 years	95+ years	1	1	Alcohol (liters per capita)
Thyroid cancer	Male	10-14 years	95+ years	2	1	Smoking Prevalence
Thyroid cancer	Female	10-14 years	95+ years	1	2	Smoking Prevalence
Thyroid cancer	Male	10-14 years	95+ years	1	2	Smoking Prevalence
Thyroid cancer	Male	10-14 years	95+ years	1	2	Tobacco (cigarettes per capita)
Thyroid cancer	Female	10-14 years	95+ years	1	2	Tobacco (cigarettes per capita)
Thyroid cancer	Male	10-14 years	95+ years	-1	2	fruits adjusted (g)
Thyroid cancer	Female	10-14 years	95+ years	-1	2	fruits adjusted (g)
Thyroid cancer	Male	10-14 years	95+ years	-1	2	vegetables adjusted (g)
Thyroid cancer	Female	10-14 years	95+ years	-1	2	vegetables adjusted (g)
Thyroid cancer	Male	10-14 years	95+ years	1	2	red meats adjusted (g)
Thyroid cancer	Female	10-14 years	95+ years	1	2	red meats adjusted (g)
Thyroid cancer	Male	10-14 years	95+ years	1	1	Log-transformed SEV scalar: Thyroid C
Thyroid cancer	Female	10-14 years	95+ years	1	1	Log-transformed SEV scalar: Thyroid C
Tracheal, bronchus, and lung cancer	Female	15-19 years	95+ years	0	3	LDI (I$ per capita)
Tracheal, bronchus, and lung cancer	Male	15-19 years	95+ years	0	3	LDI (I$ per capita)
Tracheal, bronchus, and lung cancer	Female	15-19 years	95+ years	0	3	Socio-demographic Index
Tracheal, bronchus, and lung cancer	Male	15-19 years	95+ years	0	3	Socio-demographic Index
Tracheal, bronchus, and lung cancer	Female	15-19 years	95+ years	-1	2	Healthcare access and quality index
Tracheal, bronchus, and lung cancer	Male	15-19 years	95+ years	-1	2	Healthcare access and quality index
Tracheal, bronchus, and lung cancer	Female	15-19 years	95+ years	-1	3	Education (years per capita)
Tracheal, bronchus, and lung cancer	Male	15-19 years	95+ years	-1	3	Education (years per capita)
Tracheal, bronchus, and lung cancer	Female	15-19 years	95+ years	1	2	Indoor Air Pollution (All Cooking Fuels)
Tracheal, bronchus, and lung cancer	Male	15-19 years	95+ years	1	2	Indoor Air Pollution (All Cooking Fuels)
Tracheal, bronchus, and lung cancer	Female	15-19 years	95+ years	1	2	Outdoor Air Pollution (PM2.5)
Tracheal, bronchus, and lung cancer	Male	15-19 years	95+ years	1	2	Outdoor Air Pollution (PM2.5)
Tracheal, bronchus, and lung cancer	Female	15-19 years	95+ years	1	1	Secondhand smoke
Tracheal, bronchus, and lung cancer	Male	15-19 years	95+ years	1	1	Secondhand smoke
Tracheal, bronchus, and lung cancer	Female	15-19 years	95+ years	1	1	Smoking Prevalence
Tracheal, bronchus, and lung cancer	Male	15-19 years	95+ years	1	1	Smoking Prevalence
Tracheal, bronchus, and lung cancer	Female	15-19 years	95+ years	1	1	Tobacco (cigarettes per capita)

 © 2019 Global Burden of Disease Cancer Collaboration. JAMA Oncol.

Tracheal, bronchus, and lung cancer	Male	15-19 years	95+ years	1	1	Tobacco (cigarettes per capita)
Tracheal, bronchus, and lung cancer	Female	15-19 years	95+ years	1	1	Cumulative Cigarettes (10 Years)
Tracheal, bronchus, and lung cancer	Male	15-19 years	95+ years	1	1	Cumulative Cigarettes (10 Years)
Tracheal, bronchus, and lung cancer	Female	15-19 years	95+ years	1	1	Cumulative Cigarettes (15 Years)
Tracheal, bronchus, and lung cancer	Male	15-19 years	95+ years	1	1	Cumulative Cigarettes (15 Years)
Tracheal, bronchus, and lung cancer	Female	15-19 years	95+ years	1	1	Cumulative Cigarettes (20 Years)
Tracheal, bronchus, and lung cancer	Male	15-19 years	95+ years	1	1	Cumulative Cigarettes (20 Years)
Tracheal, bronchus, and lung cancer	Female	15-19 years	95+ years	1	1	Cumulative Cigarettes (5 Years)
Tracheal, bronchus, and lung cancer	Male	15-19 years	95+ years	1	1	Cumulative Cigarettes (5 Years)
Tracheal, bronchus, and lung cancer	Female	15-19 years	95+ years	1	1	Log-transformed SEV scalar: Lung C
Tracheal, bronchus, and lung cancer	Male	15-19 years	95+ years	1	1	Log-transformed SEV scalar: Lung C
Tracheal, bronchus, and lung cancer	Female	15-19 years	95+ years	1	1	Log-transformed age-standardized SEV scalar: Lung C
Tracheal, bronchus, and lung cancer	Male	15-19 years	95+ years	1	1	Log-transformed age-standardized SEV scalar: Lung C
Tracheal, bronchus, and lung cancer	Female	15-19 years	95+ years	1	1	Asbestos consumption (metric tons per year per capita)
Tracheal, bronchus, and lung cancer	Male	15-19 years	95+ years	1	1	Asbestos consumption (metric tons per year per capita)
Uterine cancer	Female	15-19 years	95+ years	0	3	LDI (I$ per capita)
Uterine cancer	Female	15-19 years	95+ years	0	3	Socio-demographic Index
Uterine cancer	Female	15-19 years	95+ years	-1	2	Healthcare access and quality index
Uterine cancer	Female	15-19 years	95+ years	-1	3	Education (years per capita)
Uterine cancer	Female	15-19 years	95+ years	1	1	Mean BMI
Uterine cancer	Female	15-19 years	95+ years	1	2	Smoking Prevalence
Uterine cancer	Female	15-19 years	95+ years	0	2	Total Fertility Rate
Uterine cancer	Female	15-19 years	95+ years	1	2	Tobacco (cigarettes per capita)
Uterine cancer	Female	15-19 years	95+ years	-1	2	fruits adjusted (g)
Uterine cancer	Female	15-19 years	95+ years	-1	2	vegetables adjusted (g)
Uterine cancer	Female	15-19 years	95+ years	1	2	Cumulative Cigarettes (10 Years)
Uterine cancer	Female	15-19 years	95+ years	1	2	Cumulative Cigarettes (5 Years)
Uterine cancer	Female	15-19 years	95+ years	1	2	Diabetes Age-Standardized Prevalence (proportion)

© 2019 Global Burden of Disease Cancer Collaboration. JAMA Oncol.

Uterine cancer	Female	15-19 years	95+ years	1	1	Log-transformed SEV scalar: Uterus C

eTable 9: Comparison of GBD 2016 and GBD 2017 covariates used and level of covariates

Cause	Sex	Covariate	GBD 2016			GBD 2017		
			Level 1	Level 2	Level 3	Level 1	Level 2	Level 3
Acute lymphoid leukemia	Female	Alcohol (liters per capita)		X			X	
Acute lymphoid leukemia	Male	Alcohol (liters per capita)		X			X	
Acute lymphoid leukemia	Female	Education (years per capita)			X			X
Acute lymphoid leukemia	Male	Education (years per capita)			X			X
Acute lymphoid leukemia	Female	LDI (I$ per capita)			X			X
Acute lymphoid leukemia	Male	LDI (I$ per capita)			X			X
Acute lymphoid leukemia	Female	Smoking Prevalence		X			X	
Acute lymphoid leukemia	Male	Smoking Prevalence		X			X	
Acute lymphoid leukemia	Female	Socio-demographic Index			X			X
Acute lymphoid leukemia	Male	Socio-demographic Index			X			X
Acute lymphoid leukemia	Female	Tobacco (cigarettes per capita)		X			X	
Acute lymphoid leukemia	Male	Tobacco (cigarettes per capita)		X			X	
Acute lymphoid leukemia	Female	Cumulative Cigarettes (10 Years)		X			X	
Acute lymphoid leukemia	Male	Cumulative Cigarettes (10 Years)		X			X	
Acute lymphoid leukemia	Female	Cumulative Cigarettes (15 Years)		X			X	
Acute lymphoid leukemia	Male	Cumulative Cigarettes (15 Years)		X			X	
Acute lymphoid leukemia	Female	Cumulative Cigarettes (20 Years)		X			X	
Acute lymphoid leukemia	Male	Cumulative Cigarettes (20 Years)		X			X	
Acute lymphoid leukemia	Female	Cumulative Cigarettes (5 Years)		X			X	
Acute lymphoid leukemia	Male	Cumulative Cigarettes (5 Years)		X			X	
Acute lymphoid leukemia	Female	Log-transformed SEV scalar: Leukemia	X			X		
Acute lymphoid leukemia	Male	Log-transformed SEV scalar: Leukemia	X			X		
Acute lymphoid leukemia	Female	Log-transformed age-standardized SEV scalar: Leukemia	X			X		
Acute lymphoid leukemia	Male	Log-transformed age-standardized SEV scalar: Leukemia	X			X		
Acute myeloid leukemia	Female	Alcohol (liters per capita)		X			X	

© 2019 Global Burden of Disease Cancer Collaboration. JAMA Oncol.

Cause	Sex	Covariate	GBD 2016			GBD 2017		
			Level 1	Level 2	Level 3	Level 1	Level 2	Level 3
Acute myeloid leukemia	Male	Alcohol (liters per capita)		X			X	
Acute myeloid leukemia	Female	Education (years per capita)			X			X
Acute myeloid leukemia	Male	Education (years per capita)			X			X
Acute myeloid leukemia	Female	LDI (I$ per capita)			X			X
Acute myeloid leukemia	Male	LDI (I$ per capita)			X			X
Acute myeloid leukemia	Female	Smoking Prevalence		X			X	
Acute myeloid leukemia	Male	Smoking Prevalence		X			X	
Acute myeloid leukemia	Female	Socio-demographic Index			X			X
Acute myeloid leukemia	Male	Socio-demographic Index			X			X
Acute myeloid leukemia	Male	Healthcare access and quality index		X			X	
Acute myeloid leukemia	Female	Tobacco (cigarettes per capita)		X			X	
Acute myeloid leukemia	Male	Tobacco (cigarettes per capita)		X			X	
Acute myeloid leukemia	Female	Cumulative Cigarettes (10 Years)		X			X	
Acute myeloid leukemia	Male	Cumulative Cigarettes (10 Years)		X			X	
Acute myeloid leukemia	Female	Cumulative Cigarettes (15 Years)		X			X	
Acute myeloid leukemia	Male	Cumulative Cigarettes (15 Years)		X			X	
Acute myeloid leukemia	Female	Cumulative Cigarettes (20 Years)		X			X	
Acute myeloid leukemia	Male	Cumulative Cigarettes (20 Years)		X			X	
Acute myeloid leukemia	Female	Cumulative Cigarettes (5 Years)		X			X	
Acute myeloid leukemia	Male	Cumulative Cigarettes (5 Years)		X			X	
Acute myeloid leukemia	Female	Log-transformed SEV scalar: Leukemia	X			X		
Acute myeloid leukemia	Male	Log-transformed SEV scalar: Leukemia	X			X		
Acute myeloid leukemia	Female	Log-transformed age-standardized SEV scalar: Leukemia	X			X		
Acute myeloid leukemia	Male	Log-transformed age-standardized SEV scalar: Leukemia	X			X		
Bladder cancer	Male	Alcohol (liters per capita)		X			X	
Bladder cancer	Female	Alcohol (liters per capita)		X			X	
Bladder cancer	Male	Education (years per capita)			X			X
Bladder cancer	Female	Education (years per capita)			X			X

© 2019 Global Burden of Disease Cancer Collaboration. JAMA Oncol.

Cause	Sex	Covariate	GBD 2016			GBD 2017		
			Level 1	Level 2	Level 3	Level 1	Level 2	Level 3
Bladder cancer	Male	LDI (I$ per capita)			X			X
Bladder cancer	Female	LDI (I$ per capita)			X			X
Bladder cancer	Male	Smoking Prevalence	X			X		
Bladder cancer	Female	Smoking Prevalence	X			X		
Bladder cancer	Male	Socio-demographic Index			X			X
Bladder cancer	Female	Socio-demographic Index			X			X
Bladder cancer	Male	Healthcare access and quality index		X			X	
Bladder cancer	Female	Healthcare access and quality index		X			X	
Bladder cancer	Male	fruits adjusted (g)		X			X	
Bladder cancer	Female	fruits adjusted (g)		X			X	
Bladder cancer	Male	vegetables adjusted (g)		X			X	
Bladder cancer	Female	vegetables adjusted (g)		X			X	
Bladder cancer	Male	Cumulative Cigarettes (10 Years)	X			X		
Bladder cancer	Female	Cumulative Cigarettes (10 Years)	X			X		
Bladder cancer	Male	Cumulative Cigarettes (15 Years)	X			X		
Bladder cancer	Female	Cumulative Cigarettes (15 Years)	X			X		
Bladder cancer	Male	Cumulative Cigarettes (5 Years)	X			X		
Bladder cancer	Female	Cumulative Cigarettes (5 Years)	X			X		
Bladder cancer	Male	Log-transformed SEV scalar: Bladder C	X			X		
Bladder cancer	Female	Log-transformed SEV scalar: Bladder C	X			X		
Brain and nervous system cancer	Female	Alcohol (liters per capita)	X			X		
Brain and nervous system cancer	Male	Alcohol (liters per capita)	X			X		
Brain and nervous system cancer	Female	Education (years per capita)			X			X
Brain and nervous system cancer	Male	Education (years per capita)			X			X
Brain and nervous system cancer	Female	LDI (I$ per capita)			X			X
Brain and nervous system cancer	Male	LDI (I$ per capita)			X			X
Brain and nervous system cancer	Female	Smoking Prevalence	X			X		
Brain and nervous system cancer	Male	Smoking Prevalence	X			X		
Brain and nervous system cancer	Female	Socio-demographic Index			X			X
Brain and nervous system cancer	Male	Socio-demographic Index			X			X

© 2019 Global Burden of Disease Cancer Collaboration. JAMA Oncol.

Cause	Sex	Covariate	GBD 2016			GBD 2017		
			Level 1	Level 2	Level 3	Level 1	Level 2	Level 3
Brain and nervous system cancer	Female	Healthcare access and quality index		X			X	
Brain and nervous system cancer	Male	Healthcare access and quality index		X			X	
Brain and nervous system cancer	Female	fruits adjusted (g)		X			X	
Brain and nervous system cancer	Male	fruits adjusted (g)		X			X	
Brain and nervous system cancer	Female	vegetables adjusted (g)		X			X	
Brain and nervous system cancer	Male	vegetables adjusted (g)		X			X	
Brain and nervous system cancer	Female	Cumulative Cigarettes (10 Years)	X			X		
Brain and nervous system cancer	Male	Cumulative Cigarettes (10 Years)	X			X		
Brain and nervous system cancer	Female	Cumulative Cigarettes (15 Years)	X			X		
Brain and nervous system cancer	Male	Cumulative Cigarettes (15 Years)	X			X		
Brain and nervous system cancer	Female	red meats adjusted (g)		X			X	
Brain and nervous system cancer	Male	red meats adjusted (g)		X			X	
Brain and nervous system cancer	Female	Systolic Blood Pressure (mmHg)		X			X	
Brain and nervous system cancer	Male	Systolic Blood Pressure (mmHg)		X			X	
Brain and nervous system cancer	Female	Cholesterol (total, mean per capita)		X			X	
Brain and nervous system cancer	Male	Cholesterol (total, mean per capita)		X			X	
Breast cancer	Male	Alcohol (liters per capita)	X			X		
Breast cancer	Female	Alcohol (liters per capita)	X			X		
Breast cancer	Male	Education (years per capita)			X			X
Breast cancer	Female	Education (years per capita)			X			X
Breast cancer	Male	LDI (I$ per capita)			X			X
Breast cancer	Female	LDI (I$ per capita)			X			X
Breast cancer	Male	Socio-demographic Index			X			X
Breast cancer	Female	Socio-demographic Index			X			X
Breast cancer	Male	Healthcare access and quality index		X			X	
Breast cancer	Female	Healthcare access and quality index		X			X	
Breast cancer	Male	Mean BMI	X			X		
Breast cancer	Female	Mean BMI	X			X		
Breast cancer	Female	Total Fertility Rate		X			X	
Breast cancer	Female	Age-Specific Fertility Rate		X			X	

 © 2019 Global Burden of Disease Cancer Collaboration. JAMA Oncol.

Cause	Sex	Covariate	GBD 2016			GBD 2017		
			Level 1	Level 2	Level 3	Level 1	Level 2	Level 3
Breast cancer	Male	fruits adjusted (g)		X			X	
Breast cancer	Female	fruits adjusted (g)		X			X	
Breast cancer	Male	vegetables adjusted (g)		X			X	
Breast cancer	Female	vegetables adjusted (g)		X			X	
Breast cancer	Male	Cumulative Cigarettes (10 Years)		X			X	
Breast cancer	Female	Cumulative Cigarettes (10 Years)		X			X	
Breast cancer	Male	Log-transformed SEV scalar: Breast C	X			X		
Breast cancer	Female	Log-transformed SEV scalar: Breast C	X			X		
Cervical cancer	Female	Education (years per capita)			X			X
Cervical cancer	Female	LDI (I$ per capita)			X			X
Cervical cancer	Female	Smoking Prevalence		X			X	
Cervical cancer	Female	Socio-demographic Index			X			X
Cervical cancer	Female	Healthcare access and quality index		X			X	
Cervical cancer	Female	Total Fertility Rate		X			X	
Cervical cancer	Female	Age-Specific Fertility Rate		X			X	
Cervical cancer	Female	fruits adjusted (g)		X			X	
Cervical cancer	Female	vegetables adjusted (g)		X			X	
Cervical cancer	Female	Cumulative Cigarettes (10 Years)	X			X		
Cervical cancer	Female	Cumulative Cigarettes (15 Years)	X			X		
Cervical cancer	Female	Cumulative Cigarettes (5 Years)	X			X		
Cervical cancer	Female	HIV age-standardized prevalence	X			X		
Chronic lymphoid leukemia	Male	Alcohol (liters per capita)		X			X	
Chronic lymphoid leukemia	Female	Alcohol (liters per capita)		X			X	
Chronic lymphoid leukemia	Male	Education (years per capita)			X			X
Chronic lymphoid leukemia	Female	Education (years per capita)			X			X
Chronic lymphoid leukemia	Male	LDI (I$ per capita)			X			X
Chronic lymphoid leukemia	Female	LDI (I$ per capita)			X			X
Chronic lymphoid leukemia	Male	Smoking Prevalence		X			X	
Chronic lymphoid leukemia	Female	Smoking Prevalence		X			X	
Chronic lymphoid leukemia	Male	Socio-demographic Index			X			X

**Cause**	**Sex**	**Covariate**	**GBD 2016**			**GBD 2017**		
			**Level 1**	**Level 2**	**Level 3**	**Level 1**	**Level 2**	**Level 3**
Chronic lymphoid leukemia	Female	Socio-demographic Index			X			X
Chronic lymphoid leukemia	Male	Tobacco (cigarettes per capita)		X			X	
Chronic lymphoid leukemia	Female	Tobacco (cigarettes per capita)		X			X	
Chronic lymphoid leukemia	Male	Cumulative Cigarettes (10 Years)		X			X	
Chronic lymphoid leukemia	Female	Cumulative Cigarettes (10 Years)		X			X	
Chronic lymphoid leukemia	Male	Cumulative Cigarettes (15 Years)		X			X	
Chronic lymphoid leukemia	Female	Cumulative Cigarettes (15 Years)		X			X	
Chronic lymphoid leukemia	Male	Cumulative Cigarettes (20 Years)		X			X	
Chronic lymphoid leukemia	Female	Cumulative Cigarettes (20 Years)		X			X	
Chronic lymphoid leukemia	Male	Cumulative Cigarettes (5 Years)		X			X	
Chronic lymphoid leukemia	Female	Cumulative Cigarettes (5 Years)		X			X	
Chronic lymphoid leukemia	Male	Log-transformed SEV scalar: Leukemia	X			X		
Chronic lymphoid leukemia	Female	Log-transformed SEV scalar: Leukemia	X			X		
Chronic lymphoid leukemia	Male	Log-transformed age-standardized SEV scalar: Leukemia	X			X		
Chronic lymphoid leukemia	Female	Log-transformed age-standardized SEV scalar: Leukemia	X			X		
Chronic myeloid leukemia	Female	Alcohol (liters per capita)		X			X	
Chronic myeloid leukemia	Male	Alcohol (liters per capita)		X			X	
Chronic myeloid leukemia	Female	Education (years per capita)			X			X
Chronic myeloid leukemia	Male	Education (years per capita)			X			X
Chronic myeloid leukemia	Female	LDI (I$ per capita)			X			X
Chronic myeloid leukemia	Male	LDI (I$ per capita)			X			X
Chronic myeloid leukemia	Female	Smoking Prevalence		X			X	
Chronic myeloid leukemia	Male	Smoking Prevalence		X			X	
Chronic myeloid leukemia	Female	Socio-demographic Index			X			X
Chronic myeloid leukemia	Male	Socio-demographic Index			X			X
Chronic myeloid leukemia	Female	Healthcare access and quality index		X			X	
Chronic myeloid leukemia	Male	Healthcare access and quality index		X			X	
Chronic myeloid leukemia	Female	Tobacco (cigarettes per capita)		X			X	

Cause	Sex	Covariate	GBD 2016			GBD 2017		
			Level 1	Level 2	Level 3	Level 1	Level 2	Level 3
Chronic myeloid leukemia	Male	Tobacco (cigarettes per capita)		X			X	
Chronic myeloid leukemia	Female	Cumulative Cigarettes (10 Years)		X			X	
Chronic myeloid leukemia	Male	Cumulative Cigarettes (10 Years)		X			X	
Chronic myeloid leukemia	Female	Cumulative Cigarettes (15 Years)		X			X	
Chronic myeloid leukemia	Male	Cumulative Cigarettes (15 Years)		X			X	
Chronic myeloid leukemia	Female	Cumulative Cigarettes (20 Years)		X			X	
Chronic myeloid leukemia	Male	Cumulative Cigarettes (20 Years)		X			X	
Chronic myeloid leukemia	Female	Cumulative Cigarettes (5 Years)		X			X	
Chronic myeloid leukemia	Male	Cumulative Cigarettes (5 Years)		X			X	
Chronic myeloid leukemia	Female	Log-transformed age-standardized SEV scalar: Leukemia	X			X		
Chronic myeloid leukemia	Male	Log-transformed age-standardized SEV scalar: Leukemia	X			X		
Colon and rectum cancer	Male	Alcohol (liters per capita)	X			X		
Colon and rectum cancer	Female	Alcohol (liters per capita)	X			X		
Colon and rectum cancer	Male	Education (years per capita)			X			X
Colon and rectum cancer	Female	Education (years per capita)			X			X
Colon and rectum cancer	Male	LDI (I$ per capita)			X			X
Colon and rectum cancer	Female	LDI (I$ per capita)			X			X
Colon and rectum cancer	Male	Smoking Prevalence	X			X		
Colon and rectum cancer	Female	Smoking Prevalence		X		X		
Colon and rectum cancer	Male	Socio-demographic Index			X			X
Colon and rectum cancer	Female	Socio-demographic Index			X			X
Colon and rectum cancer	Male	Healthcare access and quality index		X			X	
Colon and rectum cancer	Female	Healthcare access and quality index		X			X	
Colon and rectum cancer	Male	Mean BMI	X			X		
Colon and rectum cancer	Female	Mean BMI	X			X		
Colon and rectum cancer	Male	Tobacco (cigarettes per capita)	X			X		
Colon and rectum cancer	Female	Tobacco (cigarettes per capita)		X		X		
Colon and rectum cancer	Male	fruits adjusted (g)		X			X	

 © 2019 Global Burden of Disease Cancer Collaboration. JAMA Oncol.

Cause	Sex	Covariate	GBD 2016			GBD 2017		
			Level 1	Level 2	Level 3	Level 1	Level 2	Level 3
Colon and rectum cancer	Female	fruits adjusted (g)	X				X	
Colon and rectum cancer	Female	fruits adjusted (g)		X			X	
Colon and rectum cancer	Male	vegetables adjusted (g)		X			X	
Colon and rectum cancer	Female	vegetables adjusted (g)	X				X	
Colon and rectum cancer	Female	vegetables adjusted (g)		X			X	
Colon and rectum cancer	Male	red meats adjusted (g)	X			X		
Colon and rectum cancer	Female	red meats adjusted (g)	X			X		
Colon and rectum cancer	Male	Log-transformed SEV scalar: Colorect C	X			X		
Colon and rectum cancer	Female	Log-transformed SEV scalar: Colorect C	X			X		
Colon and rectum cancer	Male	milk adjusted (g)		X			X	
Colon and rectum cancer	Female	milk adjusted (g)		X			X	
Colon and rectum cancer	Male	nuts seeds adjusted (g)		X			X	
Colon and rectum cancer	Female	nuts seeds adjusted (g)		X			X	
Colon and rectum cancer	Male	PUFA adjusted(percent)		X			X	
Colon and rectum cancer	Female	PUFA adjusted(percent)		X			X	
Esophageal cancer	Male	Alcohol (liters per capita)	X			X		
Esophageal cancer	Female	Alcohol (liters per capita)	X			X		
Esophageal cancer	Male	Education (years per capita)			X			X
Esophageal cancer	Female	Education (years per capita)			X			X
Esophageal cancer	Male	LDI (I$ per capita)			X			X
Esophageal cancer	Female	LDI (I$ per capita)			X			X
Esophageal cancer	Male	Indoor Air Pollution (All Cooking Fuels)		X			X	
Esophageal cancer	Female	Indoor Air Pollution (All Cooking Fuels)		X			X	
Esophageal cancer	Male	Smoking Prevalence	X			X		
Esophageal cancer	Female	Smoking Prevalence	X			X		
Esophageal cancer	Male	Socio-demographic Index		X				X
Esophageal cancer	Female	Socio-demographic Index			X			X
Esophageal cancer	Female	Socio-demographic Index		X				X
Esophageal cancer	Male	Healthcare access and quality index		X			X	
Esophageal cancer	Female	Healthcare access and quality index		X			X	

© 2019 Global Burden of Disease Cancer Collaboration. JAMA Oncol.

Cause	Sex	Covariate	GBD 2016			GBD 2017		
			Level 1	Level 2	Level 3	Level 1	Level 2	Level 3
Esophageal cancer	Male	Mean BMI	X			X		
Esophageal cancer	Female	Mean BMI	X			X		
Esophageal cancer	Male	Sanitation (proportion with access)		X			X	
Esophageal cancer	Female	Sanitation (proportion with access)		X			X	
Esophageal cancer	Male	Improved Water Source (proportion with access)		X			X	
Esophageal cancer	Female	Improved Water Source (proportion with access)		X			X	
Esophageal cancer	Male	Tobacco (cigarettes per capita)	X			X		
Esophageal cancer	Female	Tobacco (cigarettes per capita)	X			X		
Esophageal cancer	Male	Log-transformed age-standardized SEV scalar: Esophag C	X			X		
Esophageal cancer	Female	Log-transformed age-standardized SEV scalar: Esophag C	X			X		
Esophageal cancer	Female	fruits adjusted (g)		X		X		
Esophageal cancer	Male	fruits adjusted (g)		X		X		
Esophageal cancer	Female	vegetables adjusted (g)		X			X	
Esophageal cancer	Male	vegetables adjusted (g)		X			X	
Esophageal cancer	Male	Log-transformed SEV scalar: Esophag C	X			X		
Gallbladder and biliary tract cancer	Female	Alcohol (liters per capita)		X			X	
Gallbladder and biliary tract cancer	Male	Alcohol (liters per capita)		X			X	
Gallbladder and biliary tract cancer	Female	Education (years per capita)			X			X
Gallbladder and biliary tract cancer	Male	Education (years per capita)			X			X
Gallbladder and biliary tract cancer	Female	LDI (I$ per capita)			X			X
Gallbladder and biliary tract cancer	Male	LDI (I$ per capita)			X			X
Gallbladder and biliary tract cancer	Female	Smoking Prevalence		X			X	
Gallbladder and biliary tract cancer	Male	Smoking Prevalence		X			X	
Gallbladder and biliary tract cancer	Female	Socio-demographic Index			X			X
Gallbladder and biliary tract cancer	Male	Socio-demographic Index			X			X
Gallbladder and biliary tract cancer	Female	Healthcare access and quality index		X			X	

© 2019 Global Burden of Disease Cancer Collaboration. JAMA Oncol.

Cause	Sex	Covariate	GBD 2016			GBD 2017		
			Level 1	Level 2	Level 3	Level 1	Level 2	Level 3
Gallbladder and biliary tract cancer	Male	Healthcare access and quality index		X			X	
Gallbladder and biliary tract cancer	Female	Mean BMI	X			X		
Gallbladder and biliary tract cancer	Male	Mean BMI	X			X		
Gallbladder and biliary tract cancer	Female	Tobacco (cigarettes per capita)		X			X	
Gallbladder and biliary tract cancer	Male	Tobacco (cigarettes per capita)		X			X	
Gallbladder and biliary tract cancer	Female	fruits adjusted (g)		X			X	
Gallbladder and biliary tract cancer	Male	fruits adjusted (g)		X			X	
Gallbladder and biliary tract cancer	Female	vegetables adjusted (g)		X			X	
Gallbladder and biliary tract cancer	Male	vegetables adjusted (g)		X			X	
Gallbladder and biliary tract cancer	Female	Cumulative Cigarettes (10 Years)		X			X	
Gallbladder and biliary tract cancer	Male	Cumulative Cigarettes (10 Years)		X			X	
Gallbladder and biliary tract cancer	Female	Diabetes Age-Standardized Prevalence (proportion)		X			X	
Gallbladder and biliary tract cancer	Male	Diabetes Age-Standardized Prevalence (proportion)		X			X	
Gallbladder and biliary tract cancer	Female	Cumulative Cigarettes (5 Years)		X			X	
Gallbladder and biliary tract cancer	Male	Cumulative Cigarettes (5 Years)		X			X	
Gallbladder and biliary tract cancer	Female	Log-transformed SEV scalar: Gallblad C	X			X		
Gallbladder and biliary tract cancer	Male	Log-transformed SEV scalar: Gallblad C	X			X		
Hodgkin lymphoma	Male	Education (years per capita)			X			X
Hodgkin lymphoma	Female	Education (years per capita)			X			X
Hodgkin lymphoma	Male	LDI (I$ per capita)			X			X
Hodgkin lymphoma	Female	LDI (I$ per capita)			X			X
Hodgkin lymphoma	Male	Socio-demographic Index			X			X
Hodgkin lymphoma	Female	Socio-demographic Index			X			X
Hodgkin lymphoma	Male	Healthcare access and quality index		X			X	
Hodgkin lymphoma	Female	Healthcare access and quality index		X			X	
Kidney cancer	Male	Alcohol (liters per capita)		X			X	
Kidney cancer	Female	Alcohol (liters per capita)		X			X	
Kidney cancer	Male	Education (years per capita)			X			X

© 2019 Global Burden of Disease Cancer Collaboration. JAMA Oncol.

Cause	Sex	Covariate	GBD 2016			GBD 2017		
			Level 1	Level 2	Level 3	Level 1	Level 2	Level 3
Kidney cancer	Female	Education (years per capita)			X			X
Kidney cancer	Male	LDI (I$ per capita)			X			X
Kidney cancer	Female	LDI (I$ per capita)			X			X
Kidney cancer	Male	Smoking Prevalence		X			X	
Kidney cancer	Female	Smoking Prevalence		X			X	
Kidney cancer	Male	Socio-demographic Index			X			X
Kidney cancer	Female	Socio-demographic Index			X			X
Kidney cancer	Male	Mean BMI	X			X		
Kidney cancer	Female	Mean BMI	X			X		
Kidney cancer	Male	Cumulative Cigarettes (10 Years)	X			X		
Kidney cancer	Female	Cumulative Cigarettes (10 Years)	X			X		
Kidney cancer	Male	Cumulative Cigarettes (15 Years)	X			X		
Kidney cancer	Female	Cumulative Cigarettes (15 Years)	X			X		
Kidney cancer	Male	Diabetes Age-Standardized Prevalence (proportion)		X			X	
Kidney cancer	Female	Diabetes Age-Standardized Prevalence (proportion)		X			X	
Kidney cancer	Male	Cumulative Cigarettes (5 Years)	X			X		
Kidney cancer	Female	Cumulative Cigarettes (5 Years)	X			X		
Kidney cancer	Male	Systolic Blood Pressure (mmHg)		X			X	
Kidney cancer	Female	Systolic Blood Pressure (mmHg)		X			X	
Kidney cancer	Male	Log-transformed SEV scalar: Kidney C	X			X		
Kidney cancer	Female	Log-transformed SEV scalar: Kidney C	X			X		
Larynx cancer	Male	Alcohol (liters per capita)	X			X		
Larynx cancer	Female	Alcohol (liters per capita)	X			X		
Larynx cancer	Male	Education (years per capita)			X			X
Larynx cancer	Female	Education (years per capita)			X			X
Larynx cancer	Male	LDI (I$ per capita)			X			X
Larynx cancer	Female	LDI (I$ per capita)			X			X

Cause	Sex	Covariate	GBD 2016			GBD 2017		
			Level 1	Level 2	Level 3	Level 1	Level 2	Level 3
Larynx cancer	Male	Population Density (over 1000 ppl/sqkm, proportion)		X			X	
Larynx cancer	Female	Population Density (over 1000 ppl/sqkm, proportion)		X			X	
Larynx cancer	Male	Smoking Prevalence		X			X	
Larynx cancer	Female	Smoking Prevalence		X			X	
Larynx cancer	Male	Socio-demographic Index			X			X
Larynx cancer	Female	Socio-demographic Index			X			X
Larynx cancer	Male	Healthcare access and quality index		X			X	
Larynx cancer	Female	Healthcare access and quality index		X			X	
Larynx cancer	Male	Population Density (under 150 ppl/sqkm, proportion)		X			X	
Larynx cancer	Female	Population Density (under 150 ppl/sqkm, proportion)		X			X	
Larynx cancer	Male	Tobacco (cigarettes per capita)		X			X	
Larynx cancer	Female	Tobacco (cigarettes per capita)		X			X	
Larynx cancer	Male	fruits adjusted (g)		X			X	
Larynx cancer	Female	fruits adjusted (g)		X			X	
Larynx cancer	Male	vegetables adjusted (g)		X			X	
Larynx cancer	Female	vegetables adjusted (g)		X			X	
Larynx cancer	Male	Cumulative Cigarettes (10 Years)		X			X	
Larynx cancer	Female	Cumulative Cigarettes (10 Years)		X			X	
Larynx cancer	Male	Cumulative Cigarettes (15 Years)		X			X	
Larynx cancer	Female	Cumulative Cigarettes (15 Years)		X			X	
Larynx cancer	Male	Cumulative Cigarettes (20 Years)		X			X	
Larynx cancer	Female	Cumulative Cigarettes (20 Years)		X			X	
Larynx cancer	Male	Cumulative Cigarettes (5 Years)		X			X	
Larynx cancer	Female	Cumulative Cigarettes (5 Years)		X			X	
Larynx cancer	Male	Log-transformed SEV scalar: Larynx C	X			X		
Larynx cancer	Female	Log-transformed SEV scalar: Larynx C	X			X		

Cause	Sex	Covariate	GBD 2016			GBD 2017		
			Level 1	Level 2	Level 3	Level 1	Level 2	Level 3
Leukemia	Female	Alcohol (liters per capita)		X			X	
Leukemia	Male	Alcohol (liters per capita)		X			X	
Leukemia	Female	Education (years per capita)			X			X
Leukemia	Male	Education (years per capita)			X			X
Leukemia	Female	LDI (I$ per capita)			X			X
Leukemia	Male	LDI (I$ per capita)			X			X
Leukemia	Female	Smoking Prevalence		X			X	
Leukemia	Male	Smoking Prevalence		X			X	
Leukemia	Female	Socio-demographic Index			X			X
Leukemia	Male	Socio-demographic Index			X			X
Leukemia	Female	Healthcare access and quality index		X			X	
Leukemia	Female	Tobacco (cigarettes per capita)		X			X	
Leukemia	Male	Tobacco (cigarettes per capita)		X			X	
Leukemia	Female	Cumulative Cigarettes (10 Years)		X			X	
Leukemia	Male	Cumulative Cigarettes (10 Years)		X			X	
Leukemia	Female	Cumulative Cigarettes (15 Years)		X			X	
Leukemia	Male	Cumulative Cigarettes (15 Years)		X			X	
Leukemia	Female	Cumulative Cigarettes (20 Years)		X			X	
Leukemia	Male	Cumulative Cigarettes (20 Years)		X			X	
Leukemia	Female	Cumulative Cigarettes (5 Years)		X			X	
Leukemia	Male	Cumulative Cigarettes (5 Years)		X			X	
Leukemia	Female	Log-transformed SEV scalar: Leukemia	X			X		
Leukemia	Male	Log-transformed SEV scalar: Leukemia	X			X		
Leukemia	Female	Log-transformed age-standardized SEV scalar: Leukemia	X			X		
Leukemia	Male	Log-transformed age-standardized SEV scalar: Leukemia	X			X		
Lip and oral cavity cancer	Male	Alcohol (liters per capita)	X			X		
Lip and oral cavity cancer	Female	Alcohol (liters per capita)	X			X		
Lip and oral cavity cancer	Male	Education (years per capita)			X			X

© 2019 Global Burden of Disease Cancer Collaboration. JAMA Oncol.

**Cause**	**Sex**	**Covariate**	**GBD 2016**			**GBD 2017**		
			**Level 1**	**Level 2**	**Level 3**	**Level 1**	**Level 2**	**Level 3**
Lip and oral cavity cancer	Female	Education (years per capita)			X			X
Lip and oral cavity cancer	Male	LDI (I$ per capita)			X			X
Lip and oral cavity cancer	Female	LDI (I$ per capita)			X			X
Lip and oral cavity cancer	Male	Smoking Prevalence	X			X		
Lip and oral cavity cancer	Female	Smoking Prevalence	X			X		
Lip and oral cavity cancer	Male	Socio-demographic Index			X			X
Lip and oral cavity cancer	Female	Socio-demographic Index			X			X
Lip and oral cavity cancer	Male	Healthcare access and quality index		X			X	
Lip and oral cavity cancer	Female	Healthcare access and quality index		X			X	
Lip and oral cavity cancer	Female	Health System Access 2 (unitless)		X			X	
Lip and oral cavity cancer	Male	Tobacco (cigarettes per capita)	X			X		
Lip and oral cavity cancer	Male	fruits adjusted (g)		X			X	
Lip and oral cavity cancer	Male	vegetables adjusted (g)		X		X		
Lip and oral cavity cancer	Male	Cumulative Cigarettes (10 Years)	X			X		
Lip and oral cavity cancer	Female	Cumulative Cigarettes (10 Years)	X			X		
Lip and oral cavity cancer	Male	Cumulative Cigarettes (15 Years)	X			X		
Lip and oral cavity cancer	Male	Cumulative Cigarettes (20 Years)	X			X		
Lip and oral cavity cancer	Female	Cumulative Cigarettes (20 Years)	X			X		
Lip and oral cavity cancer	Male	red meats adjusted (g)		X			X	
Lip and oral cavity cancer	Male	Cumulative Cigarettes (5 Years)	X			X		
Lip and oral cavity cancer	Male	Log-transformed SEV scalar: Mouth C	X			X		
Liver cancer	Male	Alcohol (liters per capita)	X			X		
Liver cancer	Female	Alcohol (liters per capita)	X			X		
Liver cancer	Male	Education (years per capita)			X			X
Liver cancer	Female	Education (years per capita)			X			X
Liver cancer	Male	LDI (I$ per capita)			X			X
Liver cancer	Female	LDI (I$ per capita)			X			X
Liver cancer	Male	Socio-demographic Index			X			X
Liver cancer	Female	Socio-demographic Index			X			X
Liver cancer	Male	Healthcare access and quality index		X			X	

 © 2019 Global Burden of Disease Cancer Collaboration. JAMA Oncol.

Cause	Sex	Covariate	GBD 2016			GBD 2017		
			Level 1	Level 2	Level 3	Level 1	Level 2	Level 3
Liver cancer	Female	Healthcare access and quality index		X			X	
Liver cancer	Male	Mean BMI		X			X	
Liver cancer	Female	Mean BMI		X			X	
Liver cancer	Male	Hepatitis B (HBsAg) Seroprevalence	X			X		
Liver cancer	Female	Hepatitis B (HBsAg) Seroprevalence	X			X		
Liver cancer	Male	Hepatitis C (IgG) Seroprevalence	X			X		
Liver cancer	Female	Hepatitis C (IgG) Seroprevalence	X			X		
Liver cancer	Male	Tobacco (cigarettes per capita)	X				X	
Liver cancer	Female	Tobacco (cigarettes per capita)	X				X	
Liver cancer	Male	Cumulative Cigarettes (15 Years)	X				X	
Liver cancer	Female	Cumulative Cigarettes (15 Years)	X				X	
Liver cancer	Male	Cumulative Cigarettes (20 Years)	X				X	
Liver cancer	Female	Cumulative Cigarettes (20 Years)	X				X	
Liver cancer	Male	Diabetes Age-Standardized Prevalence (proportion)		X			X	
Liver cancer	Female	Diabetes Age-Standardized Prevalence (proportion)		X			X	
Liver cancer	Male	Log-transformed SEV scalar: Liver C	X			X		
Liver cancer	Female	Log-transformed SEV scalar: Liver C	X			X		
Liver cancer	Male	red meats adjusted (g)		X			X	
Liver cancer	Female	red meats adjusted (g)		X			X	
Malignant skin melanoma	Male	Alcohol (liters per capita)		X			X	
Malignant skin melanoma	Male	Alcohol (liters per capita)		X		X		
Malignant skin melanoma	Male	Alcohol (liters per capita)	X				X	
Malignant skin melanoma	Male	Alcohol (liters per capita)	X			X		
Malignant skin melanoma	Female	Alcohol (liters per capita)	X			X		
Malignant skin melanoma	Male	Education (years per capita)			X			X
Malignant skin melanoma	Female	Education (years per capita)			X			X
Malignant skin melanoma	Male	LDI (I$ per capita)			X			X
Malignant skin melanoma	Female	LDI (I$ per capita)			X			X

© 2019 Global Burden of Disease Cancer Collaboration. JAMA Oncol.

Cause	Sex	Covariate	GBD 2016			GBD 2017		
			Level 1	Level 2	Level 3	Level 1	Level 2	Level 3
Malignant skin melanoma	Male	Socio-demographic Index			X			X
Malignant skin melanoma	Female	Socio-demographic Index			X			X
Malignant skin melanoma	Male	Healthcare access and quality index		X			X	
Malignant skin melanoma	Female	Healthcare access and quality index		X			X	
Malignant skin melanoma	Male	Latitude Under 15 (proportion)		X			X	
Malignant skin melanoma	Female	Latitude Under 15 (proportion)		X			X	
Malignant skin melanoma	Male	Latitude 30 to 45 (proportion)		X			X	
Malignant skin melanoma	Female	Latitude 30 to 45 (proportion)		X			X	
Malignant skin melanoma	Male	Latitude Over 45 (proportion)		X			X	
Malignant skin melanoma	Female	Latitude Over 45 (proportion)		X			X	
Malignant skin melanoma	Male	Latitude 15 to 30 (proportion)		X			X	
Malignant skin melanoma	Female	Latitude 15 to 30 (proportion)		X			X	
Malignant skin melanoma	Male	fruits adjusted (g)		X			X	
Malignant skin melanoma	Female	fruits adjusted (g)		X			X	
Malignant skin melanoma	Male	vegetables adjusted (g)		X			X	
Malignant skin melanoma	Female	vegetables adjusted (g)		X			X	
Mesothelioma	Female	Education (years per capita)			X			X
Mesothelioma	Male	Education (years per capita)			X			X
Mesothelioma	Female	LDI (I$ per capita)			X			X
Mesothelioma	Male	LDI (I$ per capita)			X			X
Mesothelioma	Female	Indoor Air Pollution (All Cooking Fuels)	X			X		
Mesothelioma	Male	Indoor Air Pollution (All Cooking Fuels)	X			X		
Mesothelioma	Female	Population Density (over 1000 ppl/sqkm, proportion)		X			X	
Mesothelioma	Male	Population Density (over 1000 ppl/sqkm, proportion)		X			X	
Mesothelioma	Female	Smoking Prevalence	X			X		
Mesothelioma	Male	Smoking Prevalence	X			X		
Mesothelioma	Female	Socio-demographic Index			X			X
Mesothelioma	Male	Socio-demographic Index			X			X

© 2019 Global Burden of Disease Cancer Collaboration. JAMA Oncol.

Cause	Sex	Covariate	GBD 2016			GBD 2017		
			Level 1	Level 2	Level 3	Level 1	Level 2	Level 3
Mesothelioma	Female	Healthcare access and quality index		X			X	
Mesothelioma	Male	Healthcare access and quality index		X			X	
Mesothelioma	Female	Cumulative Cigarettes (5 Years)	X			X		
Mesothelioma	Male	Cumulative Cigarettes (5 Years)	X			X		
Mesothelioma	Female	Asbestos production (binary)	X			X		
Mesothelioma	Female	Asbestos production (kg) per capita		X			X	
Mesothelioma	Female	Gold production (binary)		X			X	
Mesothelioma	Male	Gold production (binary)		X			X	
Mesothelioma	Female	Gold production (kg) per capita		X			X	
Mesothelioma	Male	Gold production (kg) per capita		X			X	
Mesothelioma	Female	Asbestos consumption (metric tons per year per capita)	X			X		
Mesothelioma	Male	Asbestos consumption (metric tons per year per capita)	X			X		
Multiple myeloma	Male	Alcohol (liters per capita)	X			X		
Multiple myeloma	Female	Alcohol (liters per capita)	X			X		
Multiple myeloma	Male	Education (years per capita)			X			X
Multiple myeloma	Female	Education (years per capita)			X			X
Multiple myeloma	Male	LDI (I$ per capita)			X			X
Multiple myeloma	Female	LDI (I$ per capita)			X			X
Multiple myeloma	Male	Smoking Prevalence	X			X		
Multiple myeloma	Female	Smoking Prevalence	X			X		
Multiple myeloma	Male	Socio-demographic Index			X			X
Multiple myeloma	Female	Socio-demographic Index			X			X
Multiple myeloma	Male	Healthcare access and quality index		X			X	
Multiple myeloma	Female	Healthcare access and quality index		X			X	
Multiple myeloma	Male	Mean BMI		X			X	
Multiple myeloma	Female	Mean BMI		X			X	
Multiple myeloma	Male	Sanitation (proportion with access)		X			X	
Multiple myeloma	Female	Sanitation (proportion with access)		X			X	

 © 2019 Global Burden of Disease Cancer Collaboration. JAMA Oncol.

Cause	Sex	Covariate	GBD 2016			GBD 2017		
			Level 1	Level 2	Level 3	Level 1	Level 2	Level 3
Multiple myeloma	Male	Improved Water Source (proportion with access)		X			X	
Multiple myeloma	Female	Improved Water Source (proportion with access)		X			X	
Multiple myeloma	Male	Tobacco (cigarettes per capita)	X			X		
Multiple myeloma	Female	Tobacco (cigarettes per capita)	X			X		
Multiple myeloma	Male	fruits adjusted (g)		X			X	
Multiple myeloma	Female	fruits adjusted (g)		X			X	
Multiple myeloma	Male	vegetables adjusted (g)		X			X	
Multiple myeloma	Female	vegetables adjusted (g)		X			X	
Multiple myeloma	Male	red meats adjusted (g)		X			X	
Multiple myeloma	Female	red meats adjusted (g)		X			X	
Nasopharynx cancer	Female	Alcohol (liters per capita)	X			X		
Nasopharynx cancer	Male	Alcohol (liters per capita)	X			X		
Nasopharynx cancer	Female	Education (years per capita)			X			X
Nasopharynx cancer	Male	Education (years per capita)			X			X
Nasopharynx cancer	Female	LDI (I$ per capita)			X			X
Nasopharynx cancer	Male	LDI (I$ per capita)			X			X
Nasopharynx cancer	Female	Population Density (over 1000 ppl/sqkm, proportion)		X			X	
Nasopharynx cancer	Male	Population Density (over 1000 ppl/sqkm, proportion)		X			X	
Nasopharynx cancer	Female	Smoking Prevalence	X			X		
Nasopharynx cancer	Male	Smoking Prevalence	X			X		
Nasopharynx cancer	Female	Socio-demographic Index			X			X
Nasopharynx cancer	Male	Socio-demographic Index			X			X
Nasopharynx cancer	Female	Population Density (under 150 ppl/sqkm, proportion)		X			X	
Nasopharynx cancer	Male	Population Density (under 150 ppl/sqkm, proportion)		X			X	

© 2019 Global Burden of Disease Cancer Collaboration. JAMA Oncol.

Cause	Sex	Covariate	GBD 2016			GBD 2017		
			Level 1	Level 2	Level 3	Level 1	Level 2	Level 3
Nasopharynx cancer	Female	Tobacco (cigarettes per capita)	X			X		
Nasopharynx cancer	Male	Tobacco (cigarettes per capita)	X			X		
Nasopharynx cancer	Female	fruits adjusted (g)		X			X	
Nasopharynx cancer	Male	fruits adjusted (g)		X			X	
Nasopharynx cancer	Female	vegetables adjusted (g)		X			X	
Nasopharynx cancer	Male	vegetables adjusted (g)		X			X	
Nasopharynx cancer	Female	Cumulative Cigarettes (10 Years)	X			X		
Nasopharynx cancer	Male	Cumulative Cigarettes (10 Years)	X			X		
Nasopharynx cancer	Female	Cumulative Cigarettes (15 Years)	X			X		
Nasopharynx cancer	Male	Cumulative Cigarettes (15 Years)	X			X		
Nasopharynx cancer	Female	Cumulative Cigarettes (20 Years)	X			X		
Nasopharynx cancer	Male	Cumulative Cigarettes (20 Years)	X			X		
Nasopharynx cancer	Female	Cumulative Cigarettes (5 Years)	X			X		
Nasopharynx cancer	Male	Cumulative Cigarettes (5 Years)	X			X		
Nasopharynx cancer	Female	Log-transformed SEV scalar: Nasoph C	X			X		
Nasopharynx cancer	Male	Log-transformed SEV scalar: Nasoph C	X			X		
Non-Hodgkin lymphoma	Male	Alcohol (liters per capita)		X			X	
Non-Hodgkin lymphoma	Female	Alcohol (liters per capita)		X			X	
Non-Hodgkin lymphoma	Male	LDI (I$ per capita)			X			X
Non-Hodgkin lymphoma	Female	LDI (I$ per capita)			X			X
Non-Hodgkin lymphoma	Male	Smoking Prevalence		X			X	
Non-Hodgkin lymphoma	Female	Smoking Prevalence		X			X	
Non-Hodgkin lymphoma	Male	Socio-demographic Index			X			X
Non-Hodgkin lymphoma	Female	Socio-demographic Index			X			X
Non-Hodgkin lymphoma	Male	Healthcare access and quality index		X			X	
Non-Hodgkin lymphoma	Female	Healthcare access and quality index		X			X	
Non-Hodgkin lymphoma	Female	Total Fertility Rate			X			X
Non-Hodgkin lymphoma	Male	Cumulative Cigarettes (10 Years)		X			X	
Non-Hodgkin lymphoma	Female	Cumulative Cigarettes (10 Years)		X			X	
Non-melanoma skin cancer	Male	Education (years per capita)			X			X

Cause	Sex	Covariate	GBD 2016			GBD 2017		
			Level 1	Level 2	Level 3	Level 1	Level 2	Level 3
Non-melanoma skin cancer	Female	Education (years per capita)			X			X
Non-melanoma skin cancer	Male	LDI (I$ per capita)			X			X
Non-melanoma skin cancer	Female	LDI (I$ per capita)			X			X
Non-melanoma skin cancer	Male	Smoking Prevalence	X			X		
Non-melanoma skin cancer	Female	Smoking Prevalence	X			X		
Non-melanoma skin cancer	Male	Socio-demographic Index			X			X
Non-melanoma skin cancer	Female	Socio-demographic Index			X			X
Non-melanoma skin cancer	Male	Healthcare access and quality index		X			X	
Non-melanoma skin cancer	Female	Healthcare access and quality index		X			X	
Non-melanoma skin cancer	Male	Cumulative Cigarettes (10 Years)	X			X		
Non-melanoma skin cancer	Female	Cumulative Cigarettes (10 Years)	X			X		
Non-melanoma skin cancer	Male	Cumulative Cigarettes (15 Years)	X			X		
Non-melanoma skin cancer	Female	Cumulative Cigarettes (15 Years)	X			X		
Non-melanoma skin cancer	Male	Cumulative Cigarettes (5 Years)	X			X		
Non-melanoma skin cancer	Female	Cumulative Cigarettes (5 Years)	X			X		
Non-melanoma skin cancer	Male	Average latitude		X			X	
Non-melanoma skin cancer	Female	Average latitude		X			X	
Non-melanoma skin cancer (squamous-cell carcinoma)	Male	Education (years per capita)			X			X
Non-melanoma skin cancer (squamous-cell carcinoma)	Female	Education (years per capita)			X			X
Non-melanoma skin cancer (squamous-cell carcinoma)	Male	LDI (I$ per capita)			X			X
Non-melanoma skin cancer (squamous-cell carcinoma)	Female	LDI (I$ per capita)			X			X
Non-melanoma skin cancer (squamous-cell carcinoma)	Male	Smoking Prevalence	X			X		
Non-melanoma skin cancer (squamous-cell carcinoma)	Female	Smoking Prevalence	X			X		

 © 2019 Global Burden of Disease Cancer Collaboration. JAMA Oncol.

Cause	Sex	Covariate	GBD 2016			GBD 2017		
			Level 1	Level 2	Level 3	Level 1	Level 2	Level 3
Non-melanoma skin cancer (squamous-cell carcinoma)	Male	Socio-demographic Index			X			X
Non-melanoma skin cancer (squamous-cell carcinoma)	Female	Socio-demographic Index			X			X
Non-melanoma skin cancer (squamous-cell carcinoma)	Male	Healthcare access and quality index		X			X	
Non-melanoma skin cancer (squamous-cell carcinoma)	Female	Healthcare access and quality index		X			X	
Non-melanoma skin cancer (squamous-cell carcinoma)	Male	Cumulative Cigarettes (10 Years)	X			X		
Non-melanoma skin cancer (squamous-cell carcinoma)	Female	Cumulative Cigarettes (10 Years)	X			X		
Non-melanoma skin cancer (squamous-cell carcinoma)	Male	Cumulative Cigarettes (15 Years)	X			X		
Non-melanoma skin cancer (squamous-cell carcinoma)	Female	Cumulative Cigarettes (15 Years)	X			X		
Non-melanoma skin cancer (squamous-cell carcinoma)	Male	Cumulative Cigarettes (5 Years)	X			X		
Non-melanoma skin cancer (squamous-cell carcinoma)	Female	Cumulative Cigarettes (5 Years)	X			X		
Non-melanoma skin cancer (squamous-cell carcinoma)	Male	Average latitude		X			X	
Non-melanoma skin cancer (squamous-cell carcinoma)	Female	Average latitude		X			X	
Other leukemia	Female	Alcohol (liters per capita)		X			X	
Other leukemia	Male	Alcohol (liters per capita)		X			X	
Other leukemia	Female	Education (years per capita)			X			X
Other leukemia	Male	Education (years per capita)			X			X
Other leukemia	Female	LDI (I$ per capita)			X			X
Other leukemia	Male	LDI (I$ per capita)			X			X
Other leukemia	Female	Smoking Prevalence		X			X	

Cause	Sex	Covariate	GBD 2016			GBD 2017		
			Level 1	Level 2	Level 3	Level 1	Level 2	Level 3
Other leukemia	Male	Smoking Prevalence		X			X	
Other leukemia	Female	Socio-demographic Index			X			X
Other leukemia	Male	Socio-demographic Index			X			X
Other leukemia	Female	Tobacco (cigarettes per capita)		X			X	
Other leukemia	Male	Tobacco (cigarettes per capita)		X			X	
Other leukemia	Female	Cumulative Cigarettes (10 Years)		X			X	
Other leukemia	Male	Cumulative Cigarettes (10 Years)		X			X	
Other leukemia	Female	Cumulative Cigarettes (15 Years)		X			X	
Other leukemia	Male	Cumulative Cigarettes (15 Years)		X			X	
Other leukemia	Female	Cumulative Cigarettes (20 Years)		X			X	
Other leukemia	Male	Cumulative Cigarettes (20 Years)		X			X	
Other leukemia	Female	Cumulative Cigarettes (5 Years)		X			X	
Other leukemia	Male	Cumulative Cigarettes (5 Years)		X			X	
Other leukemia	Female	Log-transformed SEV scalar: Leukemia	X			X		
Other leukemia	Male	Log-transformed SEV scalar: Leukemia	X			X		
Other pharynx cancer	Male	Alcohol (liters per capita)	X			X		
Other pharynx cancer	Female	Alcohol (liters per capita)	X			X		
Other pharynx cancer	Male	Education (years per capita)			X			X
Other pharynx cancer	Female	Education (years per capita)			X			X
Other pharynx cancer	Male	LDI (I$ per capita)			X			X
Other pharynx cancer	Female	LDI (I$ per capita)			X			X
Other pharynx cancer	Male	Population Density (over 1000 ppl/sqkm, proportion)		X			X	
Other pharynx cancer	Female	Population Density (over 1000 ppl/sqkm, proportion)		X			X	
Other pharynx cancer	Male	Smoking Prevalence	X			X		
Other pharynx cancer	Female	Smoking Prevalence	X			X		
Other pharynx cancer	Male	Socio-demographic Index			X			X
Other pharynx cancer	Female	Socio-demographic Index			X			X

 © 2019 Global Burden of Disease Cancer Collaboration. JAMA Oncol.

Cause	Sex	Covariate	GBD 2016			GBD 2017		
			Level 1	Level 2	Level 3	Level 1	Level 2	Level 3
Other pharynx cancer	Male	Population Density (under 150 ppl/sqkm, proportion)		X			X	
Other pharynx cancer	Female	Population Density (under 150 ppl/sqkm, proportion)		X			X	
Other pharynx cancer	Male	fruits adjusted (g)		X			X	
Other pharynx cancer	Female	fruits adjusted (g)		X			X	
Other pharynx cancer	Male	vegetables adjusted (g)		X			X	
Other pharynx cancer	Female	vegetables adjusted (g)		X			X	
Other pharynx cancer	Male	Cumulative Cigarettes (5 Years)		X			X	
Other pharynx cancer	Female	Cumulative Cigarettes (5 Years)		X			X	
Other pharynx cancer	Male	Log-transformed SEV scalar: Oth Phar C	X			X		
Other pharynx cancer	Female	Log-transformed SEV scalar: Oth Phar C	X			X		
Ovarian cancer	Female	Alcohol (liters per capita)	X			X		
Ovarian cancer	Female	Education (years per capita)			X			X
Ovarian cancer	Female	LDI (I$ per capita)			X			X
Ovarian cancer	Female	Smoking Prevalence		X			X	
Ovarian cancer	Female	Socio-demographic Index			X			X
Ovarian cancer	Female	Healthcare access and quality index		X			X	
Ovarian cancer	Female	Mean BMI		X			X	
Ovarian cancer	Female	Total Fertility Rate		X			X	
Ovarian cancer	Female	energy unadjusted(kcal)		X			X	
Ovarian cancer	Female	Tobacco (cigarettes per capita)	X			X		
Ovarian cancer	Female	fruits adjusted (g)		X			X	
Ovarian cancer	Female	vegetables adjusted (g)		X			X	
Ovarian cancer	Female	Cumulative Cigarettes (20 Years)	X			X		
Ovarian cancer	Female	Diabetes Age-Standardized Prevalence (proportion)		X			X	
Ovarian cancer	Female	Contraception (Modern) Prevalence (proportion)	X			X		
Ovarian cancer	Female	Log-transformed SEV scalar: Ovary C	X			X		

 © 2019 Global Burden of Disease Cancer Collaboration. JAMA Oncol.

Cause	Sex	Covariate	GBD 2016			GBD 2017		
			Level 1	Level 2	Level 3	Level 1	Level 2	Level 3
Pancreatic cancer	Male	Alcohol (liters per capita)	X			X		
Pancreatic cancer	Female	Alcohol (liters per capita)		X		X		
Pancreatic cancer	Male	Education (years per capita)			X			X
Pancreatic cancer	Female	Education (years per capita)			X			X
Pancreatic cancer	Male	LDI (I$ per capita)			X			X
Pancreatic cancer	Female	LDI (I$ per capita)			X			X
Pancreatic cancer	Male	Smoking Prevalence	X			X		
Pancreatic cancer	Female	Smoking Prevalence	X			X		
Pancreatic cancer	Male	Socio-demographic Index			X			X
Pancreatic cancer	Female	Socio-demographic Index			X			X
Pancreatic cancer	Male	Healthcare access and quality index		X			X	
Pancreatic cancer	Female	Healthcare access and quality index		X			X	
Pancreatic cancer	Male	Mean BMI	X			X		
Pancreatic cancer	Female	Mean BMI	X			X		
Pancreatic cancer	Female	energy unadjusted(kcal)		X			X	
Pancreatic cancer	Male	energy unadjusted(kcal)		X			X	
Pancreatic cancer	Male	Tobacco (cigarettes per capita)	X			X		
Pancreatic cancer	Female	Tobacco (cigarettes per capita)	X			X		
Pancreatic cancer	Male	fruits adjusted (g)		X			X	
Pancreatic cancer	Female	fruits adjusted (g)		X			X	
Pancreatic cancer	Male	vegetables adjusted (g)		X			X	
Pancreatic cancer	Female	vegetables adjusted (g)		X			X	
Pancreatic cancer	Female	vegetables adjusted (g)	X				X	
Pancreatic cancer	Male	Cumulative Cigarettes (10 Years)	X			X		
Pancreatic cancer	Female	Cumulative Cigarettes (10 Years)	X			X		
Pancreatic cancer	Male	Cumulative Cigarettes (20 Years)	X			X		
Pancreatic cancer	Female	Cumulative Cigarettes (20 Years)	X			X		
Pancreatic cancer	Male	Diabetes Age-Standardized Prevalence (proportion)		X			X	

 © 2019 Global Burden of Disease Cancer Collaboration. JAMA Oncol.

Cause	Sex	Covariate	GBD 2016			GBD 2017		
			Level 1	Level 2	Level 3	Level 1	Level 2	Level 3
Pancreatic cancer	Female	Diabetes Age-Standardized Prevalence (proportion)		X			X	
Pancreatic cancer	Male	red meats adjusted (g)		X			X	
Pancreatic cancer	Female	red meats adjusted (g)		X			X	
Pancreatic cancer	Male	Cumulative Cigarettes (5 Years)		X		X		
Pancreatic cancer	Female	Cumulative Cigarettes (5 Years)	X			X		
Pancreatic cancer	Male	Log-transformed SEV scalar: Pancreas C	X			X		
Pancreatic cancer	Female	Log-transformed SEV scalar: Pancreas C	X			X		
Prostate cancer	Male	Education (years per capita)			X			X
Prostate cancer	Male	LDI (I$ per capita)			X			X
Prostate cancer	Male	Socio-demographic Index			X			X
Prostate cancer	Male	Healthcare access and quality index		X			X	
Prostate cancer	Male	Log-transformed SEV scalar: Prostate C	X			X		
Stomach cancer	Female	Education (years per capita)			X			X
Stomach cancer	Male	LDI (I$ per capita)			X			X
Stomach cancer	Female	LDI (I$ per capita)			X			X
Stomach cancer	Male	Smoking Prevalence	X			X		
Stomach cancer	Female	Smoking Prevalence	X			X		
Stomach cancer	Male	Socio-demographic Index			X			X
Stomach cancer	Female	Socio-demographic Index			X			X
Stomach cancer	Male	Healthcare access and quality index		X			X	
Stomach cancer	Female	Healthcare access and quality index		X			X	
Stomach cancer	Male	Mean BMI		X			X	
Stomach cancer	Female	Mean BMI		X			X	
Stomach cancer	Male	Sanitation (proportion with access)		X			X	
Stomach cancer	Female	Sanitation (proportion with access)		X			X	
Stomach cancer	Male	Improved Water Source (proportion with access)		X			X	
Stomach cancer	Female	Improved Water Source (proportion with access)		X			X	

© 2019 Global Burden of Disease Cancer Collaboration. JAMA Oncol.

Cause	Sex	Covariate	GBD 2016			GBD 2017		
			Level 1	Level 2	Level 3	Level 1	Level 2	Level 3
Stomach cancer	Male	Tobacco (cigarettes per capita)	X			X		
Stomach cancer	Female	Tobacco (cigarettes per capita)	X			X		
Stomach cancer	Male	fruits adjusted (g)		X			X	
Stomach cancer	Female	fruits adjusted (g)		X			X	
Stomach cancer	Male	vegetables adjusted (g)		X			X	
Stomach cancer	Female	vegetables adjusted (g)		X			X	
Stomach cancer	Male	Cumulative Cigarettes (10 Years)	X			X		
Stomach cancer	Female	Cumulative Cigarettes (10 Years)	X			X		
Stomach cancer	Male	Cumulative Cigarettes (15 Years)	X			X		
Stomach cancer	Male	Log-transformed SEV scalar: Stomach C	X			X		
Stomach cancer	Female	Log-transformed SEV scalar: Stomach C	X			X		
Stomach cancer	Male	Diet high in sodium	X			X		
Stomach cancer	Female	Diet high in sodium	X			X		
Testicular cancer	Male	Education (years per capita)			X			X
Testicular cancer	Male	LDI (I$ per capita)			X			X
Testicular cancer	Male	Socio-demographic Index			X			X
Testicular cancer	Male	Healthcare access and quality index		X			X	
Testicular cancer	Male	Cumulative Cigarettes (10 Years)		X			X	
Testicular cancer	Male	Cumulative Cigarettes (15 Years)		X			X	
Testicular cancer	Male	Cumulative Cigarettes (5 Years)		X			X	
Thyroid cancer	Female	Alcohol (liters per capita)	X			X		
Thyroid cancer	Male	Alcohol (liters per capita)	X			X		
Thyroid cancer	Female	Education (years per capita)			X			X
Thyroid cancer	Male	Education (years per capita)			X			X
Thyroid cancer	Female	LDI (I$ per capita)			X			X
Thyroid cancer	Male	LDI (I$ per capita)			X			X
Thyroid cancer	Female	Smoking Prevalence		X			X	
Thyroid cancer	Male	Smoking Prevalence		X		X		
Thyroid cancer	Male	Smoking Prevalence		X			X	
Thyroid cancer	Male	Smoking Prevalence	X			X		

 © 2019 Global Burden of Disease Cancer Collaboration. JAMA Oncol.

Cause	Sex	Covariate	GBD 2016			GBD 2017		
			Level 1	Level 2	Level 3	Level 1	Level 2	Level 3
Thyroid cancer	Male	Smoking Prevalence	X				X	
Thyroid cancer	Female	Socio-demographic Index			X			X
Thyroid cancer	Male	Socio-demographic Index			X			X
Thyroid cancer	Female	Healthcare access and quality index		X			X	
Thyroid cancer	Male	Healthcare access and quality index		X			X	
Thyroid cancer	Female	Mean BMI		X			X	
Thyroid cancer	Male	Mean BMI		X			X	
Thyroid cancer	Female	Sanitation (proportion with access)		X			X	
Thyroid cancer	Male	Sanitation (proportion with access)		X			X	
Thyroid cancer	Female	Improved Water Source (proportion with access)		X			X	
Thyroid cancer	Male	Improved Water Source (proportion with access)		X			X	
Thyroid cancer	Female	Tobacco (cigarettes per capita)		X			X	
Thyroid cancer	Male	Tobacco (cigarettes per capita)		X			X	
Thyroid cancer	Female	fruits adjusted (g)		X			X	
Thyroid cancer	Male	fruits adjusted (g)		X			X	
Thyroid cancer	Female	vegetables adjusted (g)		X			X	
Thyroid cancer	Male	vegetables adjusted (g)		X			X	
Thyroid cancer	Female	red meats adjusted (g)		X			X	
Thyroid cancer	Male	red meats adjusted (g)		X			X	
Thyroid cancer	Female	Log-transformed SEV scalar: Thyroid C	X			X		
Thyroid cancer	Male	Log-transformed SEV scalar: Thyroid C	X			X		
Tracheal, bronchus, and lung cancer	Female	Education (years per capita)			X			X
Tracheal, bronchus, and lung cancer	Male	Education (years per capita)			X			X
Tracheal, bronchus, and lung cancer	Female	LDI (I$ per capita)			X			X
Tracheal, bronchus, and lung cancer	Male	LDI (I$ per capita)			X			X
Tracheal, bronchus, and lung cancer	Female	Indoor Air Pollution (All Cooking Fuels)		X			X	
Tracheal, bronchus, and lung cancer	Male	Indoor Air Pollution (All Cooking Fuels)		X			X	
Tracheal, bronchus, and lung cancer	Female	Outdoor Air Pollution (PM2.5)		X			X	

Cause	Sex	Covariate	GBD 2016			GBD 2017		
			Level 1	Level 2	Level 3	Level 1	Level 2	Level 3
Tracheal, bronchus, and lung cancer	Male	Outdoor Air Pollution (PM2.5)		X			X	
Tracheal, bronchus, and lung cancer	Female	Smoking Prevalence	X			X		
Tracheal, bronchus, and lung cancer	Male	Smoking Prevalence	X			X		
Tracheal, bronchus, and lung cancer	Female	Socio-demographic Index			X			X
Tracheal, bronchus, and lung cancer	Male	Socio-demographic Index			X			X
Tracheal, bronchus, and lung cancer	Female	Healthcare access and quality index		X			X	
Tracheal, bronchus, and lung cancer	Male	Healthcare access and quality index		X			X	
Tracheal, bronchus, and lung cancer	Female	Tobacco (cigarettes per capita)	X			X		
Tracheal, bronchus, and lung cancer	Male	Tobacco (cigarettes per capita)	X			X		
Tracheal, bronchus, and lung cancer	Female	Cumulative Cigarettes (10 Years)	X			X		
Tracheal, bronchus, and lung cancer	Male	Cumulative Cigarettes (10 Years)	X			X		
Tracheal, bronchus, and lung cancer	Female	Cumulative Cigarettes (15 Years)	X			X		
Tracheal, bronchus, and lung cancer	Male	Cumulative Cigarettes (15 Years)	X			X		
Tracheal, bronchus, and lung cancer	Female	Cumulative Cigarettes (20 Years)	X			X		
Tracheal, bronchus, and lung cancer	Male	Cumulative Cigarettes (20 Years)	X			X		
Tracheal, bronchus, and lung cancer	Female	Cumulative Cigarettes (5 Years)	X			X		
Tracheal, bronchus, and lung cancer	Male	Cumulative Cigarettes (5 Years)	X			X		
Tracheal, bronchus, and lung cancer	Female	Log-transformed SEV scalar: Lung C	X			X		
Tracheal, bronchus, and lung cancer	Male	Log-transformed SEV scalar: Lung C	X			X		
Tracheal, bronchus, and lung cancer	Female	Log-transformed age-standardized SEV scalar: Lung C	X			X		
Tracheal, bronchus, and lung cancer	Male	Log-transformed age-standardized SEV scalar: Lung C	X			X		
Uterine cancer	Female	Education (years per capita)			X			X
Uterine cancer	Female	LDI (I$ per capita)			X			X
Uterine cancer	Female	Smoking Prevalence		X			X	
Uterine cancer	Female	Socio-demographic Index			X			X
Uterine cancer	Female	Healthcare access and quality index		X			X	
Uterine cancer	Female	Mean BMI	X			X		
Uterine cancer	Female	Total Fertility Rate		X			X	

© 2019 Global Burden of Disease Cancer Collaboration. JAMA Oncol.

Cause	Sex	Covariate	GBD 2016			GBD 2017		
			Level 1	Level 2	Level 3	Level 1	Level 2	Level 3
Uterine cancer	Female	Tobacco (cigarettes per capita)		X			X	
Uterine cancer	Female	fruits adjusted (g)		X			X	
Uterine cancer	Female	vegetables adjusted (g)		X			X	
Uterine cancer	Female	Cumulative Cigarettes (10 Years)		X			X	
Uterine cancer	Female	Diabetes Age-Standardized Prevalence (proportion)		X			X	
Uterine cancer	Female	Cumulative Cigarettes (5 Years)		X			X	
Uterine cancer	Female	Log-transformed SEV scalar: Uterus C	X			X		

eTable 10: Results for CODEm model testing

Cause	Sex	Age start	Age end	RMSE in	RMSE out	Trend in	Trend out	Coverage in	Coverage out
Esophageal cancer [Global]	Male	15-19 years	95+ years	0.271316	0.427854	0.205445	0.199855	0.997669	0.981185
Esophageal cancer [Global]	Female	15-19 years	95+ years	0.302361	0.493874	0.2317	0.224894	0.995681	0.980883
Esophageal cancer [Data Rich]	Male	15-19 years	95+ years	0.239895	0.300276	0.187005	0.203166	0.998625	0.997254
Esophageal cancer [Data Rich]	Female	15-19 years	95+ years	0.262599	0.334148	0.207727	0.236855	0.996943	0.996228
Stomach cancer [Global]	Female	15-19 years	95+ years	0.197066	0.311293	0.153993	0.158753	0.999106	0.986096
Stomach cancer [Data Rich]	Female	15-19 years	95+ years	0.171356	0.213965	0.13582	0.148335	0.99938	0.998545
Stomach cancer [Global]	Male	15-19 years	95+ years	0.203033	0.305319	0.158061	0.159925	0.99871	0.985866
Stomach cancer [Data Rich]	Male	15-19 years	95+ years	0.179392	0.221151	0.141635	0.151978	0.998922	0.997836
Liver cancer [Global]	Male	5-9 years	95+ years	0.267389	0.412643	0.209002	0.20667	0.998935	0.990285

© 2019 Global Burden of Disease Cancer Collaboration. JAMA Oncol.

Cause	Sex	Age start	Age end	RMSE in	RMSE out	Trend in	Trend out	Coverage in	Coverage out
Liver cancer [Data Rich]	Male	5-9 years	95+ years	0.229806	0.299408	0.188299	0.214908	0.999276	0.998608
Liver cancer [Global]	Female	5-9 years	95+ years	0.272486	0.405092	0.210121	0.213135	0.998713	0.99157
Liver cancer [Data Rich]	Female	5-9 years	95+ years	0.231145	0.303877	0.189712	0.213305	0.999019	0.998295
Larynx cancer [Global]	Male	15-19 years	95+ years	0.25327	0.37169	0.204343	0.206604	0.997733	0.990714
Larynx cancer [Global]	Female	15-19 years	95+ years	0.332425	0.529369	0.272032	0.272355	0.982841	0.977421
Larynx cancer [Data Rich]	Male	15-19 years	95+ years	0.213815	0.260863	0.172458	0.181282	0.99722	0.99693
Larynx cancer [Data Rich]	Female	15-19 years	95+ years	0.290319	0.365907	0.245204	0.279464	0.979378	0.980174
Tracheal, bronchus, and lung cancer [Data Rich]	Female	15-19 years	95+ years	0.241549	0.305833	0.19181	0.212529	0.997935	0.995941
Tracheal, bronchus, and lung cancer [Global]	Male	15-19 years	95+ years	0.241892	0.362338	0.188073	0.183458	0.998705	0.981847
Tracheal, bronchus, and lung cancer [Global]	Female	15-19 years	95+ years	0.266197	0.399225	0.20529	0.205262	0.997793	0.983526
Tracheal, bronchus, and lung cancer [Data Rich]	Male	15-19 years	95+ years	0.208587	0.259273	0.166474	0.182672	0.998632	0.997141
Breast cancer [Global]	Male	15-19 years	95+ years	0.422337	0.589378	0.323647	0.321288	0.979596	0.972194
Breast cancer [Global]	Female	15-19 years	95+ years	0.216748	0.310419	0.165176	0.166307	0.997027	0.98881
Breast cancer [Data Rich]	Male	15-19 years	95+ years	0.34414	0.423914	0.292649	0.322339	0.980114	0.978151
Breast cancer [Data Rich]	Female	15-19 years	95+ years	0.195491	0.240038	0.155341	0.166431	0.997292	0.99507
Cervical cancer [Global]	Female	15-19 years	95+ years	0.245094	0.352516	0.19563	0.192966	0.998476	0.989165

© 2019 Global Burden of Disease Cancer Collaboration. JAMA Oncol.

Cause	Sex	Age start	Age end	RMSE in	RMSE out	Trend in	Trend out	Coverage in	Coverage out
Cervical cancer [Data Rich]	Female	15-19 years	95+ years	0.241724	0.29846	0.186658	0.217324	0.9987	0.997897
Uterine cancer [Global]	Female	15-19 years	95+ years	0.391178	0.503325	0.32267	0.320455	0.993716	0.985594
Uterine cancer [Data Rich]	Female	15-19 years	95+ years	0.347638	0.432616	0.293526	0.339577	0.992896	0.990392
Prostate cancer [Global]	Male	15-19 years	95+ years	0.261885	0.334721	0.194583	0.189884	0.994471	0.989097
Prostate cancer [Data Rich]	Male	15-19 years	95+ years	0.253196	0.306624	0.200772	0.221651	0.99481	0.992986
Colon and rectum cancer [Global]	Male	15-19 years	95+ years	0.215815	0.307782	0.168321	0.166479	0.999167	0.991478
Colon and rectum cancer [Global]	Female	15-19 years	95+ years	0.207768	0.285991	0.162728	0.161992	0.9991	0.993835
Colon and rectum cancer [Data Rich]	Male	15-19 years	95+ years	0.186963	0.231209	0.148635	0.162856	0.999058	0.998396
Colon and rectum cancer [Data Rich]	Female	15-19 years	95+ years	0.185287	0.229259	0.149807	0.168456	0.998848	0.997696
Lip and oral cavity cancer [Global]	Male	15-19 years	95+ years	0.243983	0.329562	0.195246	0.192919	0.999138	0.995276
Lip and oral cavity cancer [Global]	Female	15-19 years	95+ years	0.229471	0.321962	0.17436	0.177688	0.999838	0.998323
Lip and oral cavity cancer [Data Rich]	Male	15-19 years	95+ years	0.222917	0.264831	0.184055	0.187256	0.999655	0.999148
Lip and oral cavity cancer [Data Rich]	Female	15-19 years	95+ years	0.200638	0.243518	0.163584	0.175329	0.999962	0.99985
Nasopharynx cancer [Global]	Male	5-9 years	95+ years	0.294744	0.434429	0.245704	0.244664	0.99937	0.988833
Nasopharynx cancer [Global]	Female	5-9 years	95+ years	0.310529	0.513749	0.251792	0.255842	0.994865	0.985828
Nasopharynx cancer [Data Rich]	Male	5-9 years	95+ years	0.273312	0.334949	0.23703	0.256489	0.999498	0.999131

© 2019 Global Burden of Disease Cancer Collaboration. JAMA Oncol.

Cause	Sex	Age start	Age end	RMSE in	RMSE out	Trend in	Trend out	Coverage in	Coverage out
Nasopharynx cancer [Data Rich]	Female	5-9 years	95+ years	0.275621	0.349798	0.236947	0.275996	0.995706	0.995532
Other pharynx cancer [Global]	Male	15-19 years	95+ years	0.272454	0.418083	0.21142	0.208013	0.997829	0.991554
Other pharynx cancer [Global]	Female	15-19 years	95+ years	0.27185	0.384483	0.216709	0.210653	0.997378	0.994978
Other pharynx cancer [Data Rich]	Male	15-19 years	95+ years	0.224387	0.285489	0.184834	0.189465	0.997791	0.997215
Other pharynx cancer [Data Rich]	Female	15-19 years	95+ years	0.241625	0.303076	0.201824	0.223991	0.997068	0.996908
Gallbladder and biliary tract cancer [Global]	Male	15-19 years	95+ years	0.254268	0.366804	0.193857	0.196686	0.999086	0.994061
Gallbladder and biliary tract cancer [Data Rich]	Male	15-19 years	95+ years	0.208396	0.261747	0.17115	0.183115	0.998903	0.998238
Gallbladder and biliary tract cancer [Data Rich]	Female	15-19 years	95+ years	0.208549	0.266142	0.165342	0.175829	0.997261	0.996325
Gallbladder and biliary tract cancer [Global]	Female	15-19 years	95+ years	0.260782	0.384139	0.19097	0.194698	0.997558	0.989035
Pancreatic cancer [Global]	Male	15-19 years	95+ years	0.230022	0.325349	0.17727	0.181061	0.998899	0.996541
Pancreatic cancer [Global]	Female	15-19 years	95+ years	0.226263	0.329164	0.173318	0.180608	0.99891	0.995753
Pancreatic cancer [Data Rich]	Male	15-19 years	95+ years	0.199472	0.251215	0.159138	0.170106	0.99881	0.998039
Pancreatic cancer [Data Rich]	Female	15-19 years	95+ years	0.205278	0.263711	0.163246	0.180398	0.998614	0.997617
Malignant skin melanoma [Global]	Female	15-19 years	95+ years	0.317541	0.425864	0.237954	0.233187	0.997069	0.992775
Malignant skin melanoma [Data Rich]	Male	15-19 years	95+ years	0.302786	0.362217	0.238219	0.244798	0.997655	0.996336
Malignant skin melanoma [Data Rich]	Female	15-19 years	95+ years	0.308657	0.349784	0.225476	0.22533	0.997276	0.996477

© 2019 Global Burden of Disease Cancer Collaboration. JAMA Oncol.

Cause	Sex	Age start	Age end	RMSE in	RMSE out	Trend in	Trend out	Coverage in	Coverage out
Malignant skin melanoma [Global]	Male	15-19 years	95+ years	0.324862	0.470987	0.247424	0.248056	0.997433	0.99238
Non-melanoma skin cancer [Global]	Male	15-19 years	95+ years	0.195688	0.319609	0.137537	0.140799	0.999637	0.996403
Non-melanoma skin cancer [Global]	Female	15-19 years	95+ years	0.287998	0.483215	0.206044	0.206916	0.995877	0.987457
Non-melanoma skin cancer [Data Rich]	Male	15-19 years	95+ years	0.158274	0.251075	0.119607	0.140712	0.99992	0.999745
Non-melanoma skin cancer [Data Rich]	Female	15-19 years	95+ years	0.208859	0.29464	0.163968	0.184091	0.998381	0.998243
Ovarian cancer [Global]	Female	15-19 years	95+ years	0.269338	0.379484	0.210419	0.21246	0.997991	0.992838
Ovarian cancer [Data Rich]	Female	15-19 years	95+ years	0.249656	0.314928	0.2025	0.223452	0.997926	0.996589
Testicular cancer [Global]	Male	15-19 years	95+ years	0.525353	0.75263	0.41868	0.413082	0.99175	0.979829
Testicular cancer [Data Rich]	Male	15-19 years	95+ years	0.44447	0.585815	0.380785	0.409933	0.994954	0.993704
Kidney cancer [Global]	Male	0-6 days	95+ years	0.289998	0.386281	0.22212	0.224688	0.998628	0.995196
Kidney cancer [Global]	Female	0-6 days	95+ years	0.315211	0.440159	0.250794	0.258895	0.9992	0.995614
Kidney cancer [Data Rich]	Male	0-6 days	95+ years	0.255827	0.338846	0.204055	0.215843	0.998978	0.99814
Kidney cancer [Data Rich]	Female	0-6 days	95+ years	0.285315	0.393522	0.233569	0.272496	0.999415	0.998674
Bladder cancer [Global]	Male	15-19 years	95+ years	0.287397	0.365265	0.230569	0.228823	0.998278	0.994441
Bladder cancer [Data Rich]	Male	15-19 years	95+ years	0.264631	0.315756	0.216242	0.237309	0.998019	0.996354
Bladder cancer [Global]	Female	15-19 years	95+ years	0.279008	0.378	0.223774	0.223645	0.998263	0.996579

© 2019 Global Burden of Disease Cancer Collaboration. JAMA Oncol.

Cause	Sex	Age start	Age end	RMSE in	RMSE out	Trend in	Trend out	Coverage in	Coverage out
Bladder cancer [Data Rich]	Female	15-19 years	95+ years	0.252722	0.308163	0.208564	0.23335	0.997751	0.997004
Brain and nervous system cancer [Global]	Male	0-6 days	95+ years	0.287997	0.40759	0.207832	0.210594	0.999286	0.995762
Brain and nervous system cancer [Global]	Female	0-6 days	95+ years	0.295571	0.441965	0.210233	0.213385	0.99934	0.994437
Brain and nervous system cancer [Data Rich]	Female	0-6 days	95+ years	0.25376	0.34089	0.198323	0.221916	0.99925	0.998585
Brain and nervous system cancer [Data Rich]	Male	0-6 days	95+ years	0.251652	0.330656	0.195936	0.221749	0.999177	0.99846
Thyroid cancer [Global]	Male	10-14 years	95+ years	0.32668	0.411793	0.258283	0.256036	0.999003	0.997237
Thyroid cancer [Global]	Female	10-14 years	95+ years	0.393795	0.469805	0.309749	0.297907	0.99732	0.99322
Thyroid cancer [Data Rich]	Male	10-14 years	95+ years	0.29961	0.343919	0.24192	0.242875	0.999315	0.998819
Thyroid cancer [Data Rich]	Female	10-14 years	95+ years	0.373407	0.409581	0.306816	0.270715	0.996954	0.995009
Mesothelioma [Global]	Female	15-19 years	95+ years	0.282864	0.467146	0.215374	0.224718	0.998947	0.992185
Mesothelioma [Data Rich]	Female	15-19 years	95+ years	0.237996	0.351665	0.1964	0.259248	0.999361	0.997967
Mesothelioma [Global]	Male	15-19 years	95+ years	0.426748	0.606294	0.328862	0.344277	0.991646	0.984781
Mesothelioma [Data Rich]	Male	15-19 years	95+ years	0.363144	0.488407	0.307957	0.383513	0.993075	0.991967
Hodgkin lymphoma [Global]	Male	0-6 days	95+ years	0.415824	0.517674	0.315878	0.307721	0.998828	0.995846
Hodgkin lymphoma [Data Rich]	Male	0-6 days	95+ years	0.329028	0.412763	0.268298	0.28595	0.999218	0.998668
Hodgkin lymphoma [Global]	Female	0-6 days	95+ years	0.569227	0.727116	0.389178	0.38162	0.985625	0.98131

Cause	Sex	Age start	Age end	RMSE in	RMSE out	Trend in	Trend out	Coverage in	Coverage out
Hodgkin lymphoma [Data Rich]	Female	0-6 days	95+ years	0.424797	0.609332	0.331318	0.327455	0.981233	0.977821
Non-Hodgkin lymphoma [Global]	Male	0-6 days	95+ years	0.305999	0.412638	0.247129	0.243252	0.999259	0.995427
Non-Hodgkin lymphoma [Global]	Female	0-6 days	95+ years	0.316899	0.423081	0.256181	0.250749	0.998991	0.994631
Non-Hodgkin lymphoma [Data Rich]	Male	0-6 days	95+ years	0.279909	0.349591	0.233112	0.240689	0.999379	0.998026
Non-Hodgkin lymphoma [Data Rich]	Female	0-6 days	95+ years	0.28747	0.361956	0.242374	0.256737	0.99904	0.996828
Multiple myeloma [Global]	Male	15-19 years	95+ years	0.364477	0.445523	0.286465	0.275877	0.997234	0.992632
Multiple myeloma [Global]	Female	15-19 years	95+ years	0.339401	0.424347	0.268622	0.260954	0.997969	0.995751
Multiple myeloma [Data Rich]	Male	15-19 years	95+ years	0.308363	0.37482	0.250986	0.260853	0.998218	0.99702
Multiple myeloma [Data Rich]	Female	15-19 years	95+ years	0.285548	0.352248	0.235543	0.245693	0.997842	0.996106
Leukemia [Global]	Male	0-6 days	95+ years	0.306475	0.377643	0.280105	0.285654	0.998208	0.995148
Leukemia [Global]	Female	0-6 days	95+ years	0.288726	0.351885	0.24126	0.236533	0.999433	0.996253
Leukemia [Data Rich]	Male	0-6 days	95+ years	0.236805	0.294968	0.198659	0.229014	0.999188	0.998035
Leukemia [Data Rich]	Female	0-6 days	95+ years	0.234065	0.284538	0.199422	0.225589	0.999161	0.997809
Other malignant neoplasms [Global]	Male	0-6 days	95+ years	0.272826	0.358933	0.225949	0.232273	0.997717	0.995307
Other malignant neoplasms [Global]	Female	0-6 days	95+ years	0.267153	0.34739	0.219567	0.224348	0.998184	0.995562
Other malignant neoplasms [Data Rich]	Male	0-6 days	95+ years	0.256434	0.30301	0.218515	0.223732	0.997282	0.995095

© 2019 Global Burden of Disease Cancer Collaboration. JAMA Oncol.

Cause	Sex	Age start	Age end	RMSE in	RMSE out	Trend in	Trend out	Coverage in	Coverage out
Other malignant neoplasms [Data Rich]	Female	0-6 days	95+ years	0.258453	0.303888	0.214106	0.22064	0.997583	0.995989
Myelodysplastic, myeloproliferative, and other hematopoietic neoplasms [Data Rich]	Male	0-6 days	95+ years	0.320492	0.399563	0.183437	0.216861	0.997662	0.997192
Myelodysplastic, myeloproliferative, and other hematopoietic neoplasms [Global]	Male	0-6 days	95+ years	0.393172	0.598192	0.224264	0.220657	0.994678	0.983857
Myelodysplastic, myeloproliferative, and other hematopoietic neoplasms [Data Rich]	Female	0-6 days	95+ years	0.234272	0.38783	0.166407	0.21485	0.997248	0.997123
Myelodysplastic, myeloproliferative, and other hematopoietic neoplasms [Global]	Female	0-6 days	95+ years	0.350205	0.569814	0.216349	0.228908	0.994822	0.987224

 © 2019 Global Burden of Disease Cancer Collaboration. JAMA Oncol.

eTable 11: Percent change before and after CoDCorrect by cancer for all ages, both sexes combined, 2017

Cause	CoDCorrect level	Percent change
Neoplasms	2	2.07<br>-1.93 to 6.19
Esophageal cancer	3	1.48<br>-3.85 to 6.95
Stomach cancer	3	0.42<br>-3.27 to 4.17
Liver cancer	3	-0.42<br>-9.0 to 6.49
Liver cancer due to hepatitis B	4	-17.92<br>-31.35 to -1.43
Liver cancer due to hepatitis C	4	-13.12<br>-23.78 to -2.2
Liver cancer due to alcohol use	4	-18.81<br>-33.09 to -1.12
Liver cancer due to other causes	4	43.8<br>22.91 to 70.2
Larynx cancer	3	6.57<br>1.28 to 12.12
Tracheal, bronchus, and lung cancer	3	-4.08<br>-8.66 to -0.0
Breast cancer	3	3.82<br>-2.11 to 11.11
Cervical cancer	3	5.27<br>-6.92 to 18.94
Uterine cancer	3	-2.97<br>-10.19 to 4.61
Prostate cancer	3	-0.21<br>-19.14 to 37.36
Colon and rectum cancer	3	1.93<br>-2.01 to 5.7
Lip and oral cavity cancer	3	-5.9<br>-12.23 to 0.97
Nasopharynx cancer	3	1.39<br>-6.45 to 9.27
Other pharynx cancer	3	13.97<br>2.17 to 27.95
Gallbladder and biliary tract cancer	3	-9.98<br>-25.56 to 5.56
Pancreatic cancer	3	1.33<br>-2.49 to 5.23
Malignant skin melanoma	3	8.25<br>-14.1 to 29.36
Non-melanoma skin cancer	3	-0.71<br>-5.86 to 5.52

 © 2019 Global Burden of Disease Cancer Collaboration. JAMA Oncol.

Ovarian cancer	3	-2.6<br>-8.48 to 5.49
Testicular cancer	3	-53.85<br>-64.01 to -35.82
Kidney cancer	3	7.11<br>1.24 to 15.21
Bladder cancer	3	6.99<br>1.58 to 14.65
Brain and nervous system cancer	3	-6.48<br>-16.09 to 17.93
Thyroid cancer	3	12.81<br>5.56 to 28.28
Mesothelioma	3	6.88<br>-1.22 to 15.53
Hodgkin lymphoma	3	27.42<br>11.82 to 60.49
Non-Hodgkin lymphoma	3	-1.97<br>-6.73 to 10.11
Multiple myeloma	3	-3.8<br>-14.7 to 12.64
Leukemia	3	0.82<br>-10.01 to 11.6
Other malignant cancers	3	89.59<br>69.04 to 106.25
Myelodysplastic, myeloproliferative, and other hematopoietic neoplasms	4	-100.0<br>-100.0 to -100.0

© 2019 Global Burden of Disease Cancer Collaboration. JAMA Oncol.

eTable 12: Duration of four prevalence phases by cancer

	Diagnosis/ Treatment (months)	Remission	Disseminated/metastatic (months)	Note	Terminal (months)
Esophageal cancer	5[41]	Calculated based on remainder of time after attributing other sequelae.	4.6[42]	SEER Summary Stage 1997 (Distant site/node involved) 1995-2000	1 months
Stomach cancer	5.2[41]		3.88[42]	SEER Summary Stage 1997 (Distant site/node involved) 1995-2000	
Liver cancer	4		2.51[42]	SEER Summary Stage 1997 (Distant site/node involved) 1995-2000	
Larynx cancer	5.3[41]		8.84[42]	SEER Stage IVc	
Lung cancer	3.3[43]		4.51[42]	SEER Summary Stage 1997 (Distant site/node involved) 1995-2000	
Breast cancer	3[43]		17.7[42]	SEER Summary Stage 1997 (Distant site/node involved) 1995-2000	
Cervical cancer	4.8[41]		9.21[42]	SEER Summary Stage 1997 (Distant site/node involved) 1995-2000	
Uterine cancer	4.6[41]		11.6[42]	SEER Summary Stage 1997 (Distant site/node involved) 1995-2000	
Prostate cancer	4[43]		30.35[42]	SEER Summary Stage 1997 (Distant site/node involved) 1995-2000	
Colorectal cancer	4[43]		9.69[42]	SEER Summary Stage 1997 (Distant site/node involved) 1995-2000	
Oral cancer	5.3[41]		9.33[42]	SEER Stage IVc	
Nasopharyngeal cancer	5.3[41]		13.19[42]	SEER Stage IVc	
Cancer of other part of pharynx	5.3[41]		7.91[42]	SEER Stage IVc	
Gallbladder cancer	4		3.47[42]	SEER Summary Stage 1997 (Distant site/node involved) 1995-2000	
Pancreas cancer	4.1[41]		2.54[42]	SEER Summary Stage 1997 (Distant site/node involved) 1995-2000	

 © 2019 Global Burden of Disease Cancer Collaboration. JAMA Oncol.

	Diagnosis/ Treatment (months)	Remission	Disseminated/metastatic (months)	Note	Terminal (months)
Melanoma	2.9[44]		7.18[42]	SEER Summary Stage 1997 (Distant site/node involved) 1995-2000	
NMSC (squamous cell carcinoma)	2.9[44]		17[45]		
Ovarian cancer	3.2[43]		25.6[42]	SEER Summary Stage 1997 (Distant site/node involved) 1995-2000	
Testicular cancer	3.7[41]		19.47[42]	SEER Stage III	
Kidney cancer	5.3[41]		5.38[42]	SEER Summary Stage 1997 (Distant site/node involved) 1995-2000	
Bladder cancer	5.1[41]		5.8[42]	SEER Summary Stage 1997 (Distant site/node involved) 1995-2000	
Brain cancer	5		6.93[42]	SEER Median age standardized survival all patients, all years	
Thyroid cancer	3		19.39[42]	SEER Stage IVc	
Mesothelioma	4		7.75[42]	SEER Summary Stage 1997 (Distant site/node involved) 1995-2000	
Hodgkin lymphoma	3.7[43]		26[46]		
Non-Hodgkin lymphoma	3.7[43]		7.7[46]		
Multiple myeloma	7[41]		36.82[42]	SEER Median age standardized survival all patients, all years	
Leukemia[41]	5		43.67[42]	SEER Median age standardized survival all patients, all years	
ALL	12		7.02[42]	SEER Median age standardized survival all patients, all years	
AML	6		4.6[42]	SEER Median age standardized survival all patients, all years	
CLL	6		48[47]		
CML	6		4.6[42]	SEER Median age standardized survival for AML (patients with CML die in blast	

© 2019 Global Burden of Disease Cancer Collaboration. JAMA Oncol.

	Diagnosis/ Treatment (months)	Remission	Disseminated/metastatic (months)	Note	Terminal (months)
				crisis, which is treated like AML) all patients, all years	
Other leukemia	6		48[47]		
Other	4.4 (mean of other cancer durations)		15.81[42]	SEER Median age standardized survival all patients, all years	

 © 2019 Global Burden of Disease Cancer Collaboration. JAMA Oncol.

eTable 13: Disability weights

**Health state**	**Lay description**	**Estimate**	**Uncertainty interval**	
Cancer, diagnosis and primary therapy	Has pain, nausea, fatigue, weight loss and high anxiety.	0.288	0.193	0.399
Cancer, controlled phase	Has a chronic disease that requires medication every day and causes some worry but minimal interference with daily activities.	0.049	0.031	0.072
Cancer, metastatic	Has severe pain, extreme fatigue, weight loss and high anxiety.	0.451	0.307	0.600
Terminal phase, with medication	Has lost a lot of weight and regularly uses strong medication to avoid constant pain. The person has no appetite, feels nauseous, and needs to spend most of the day in bed.	0.540	0.377	0.687
Mastectomy	Had one of the breasts removed and sometimes has pain or swelling in the arms.	0.036	0.020	0.057
Stoma	Has a pouch attached to an opening in the belly to collect and empty stools.	0.095	0.063	0.131
Laryngectomy	Has difficulty speaking, and others find it difficult to understand.	0.051	0.032	0.078
Urinary incontinence	Cannot control urinating.	0.139	0.094	0.198
Impotence	Has difficulty in obtaining or maintaining an erection.	0.017	0.009	0.030
Cutaneous squamous cell carcinoma, mild	Has a slight, visible physical deformity that others notice, which causes some worry and discomfort.	0.011	0.005	0.021
Cutaneous squamous cell carcinoma, moderate	Has a visible physical deformity that causes others to stare and comment. As a result, the person is worried and has trouble sleeping and concentrating.	0.067	0.044	0.096
Cutaneous squamous cell carcinoma, severe	Has an obvious physical deformity that is very painful and itchy. The physical deformity makes others uncomfortable, which causes the person to avoid social contact, feel worried, sleep poorly, and think about suicide.	0.576	0.401	0.731
Disfigurement due to basal cell carcinoma	Has a slight, visible physical deformity that others notice, which causes some worry and discomfort.	0.011	0.005	0.021

 © 2019 Global Burden of Disease Cancer Collaboration. JAMA Oncol.

eTable 14: Decomposition of trends in incidence globally, and by SDI quintile, both sexes, 2007 to 2017

Cause	Location	Incidence cases, No.		Expected incidence cases, 2017, No.		Change in incidence cases, 2007 to 2017, %			Overall change, %
		2007	2017	Given population growth alone	Given population growth and aging	Due to population growth	Due to change in age structure	Due to change in incidence rate	
Neoplasms	Global	18363006	24490934	20729426	23925301	12.9	17.4	3.1	33.4
Neoplasms	High SDI	10029569	12370677	10627733	12147895	6	15.2	2.2	23.3
Neoplasms	High-middle SDI	3289377	4655552	3549405	4194610	7.9	19.6	14	41.5
Neoplasms	Middle SDI	2977105	4521483	3266447	3977385	9.7	23.9	18.3	51.9
Neoplasms	Low-middle SDI	1305724	1872608	1550724	1713165	18.8	12.4	12.2	43.4
Neoplasms	Low SDI	703571	981047	873410	945594	24.1	10.3	5	39.4
Lip and oral cavity cancer	Global	277567	389760	313336	357481	12.9	15.9	11.6	40.4
Lip and oral cavity cancer	High SDI	83142	100223	88100	99310	6	13.5	1.1	20.5
Lip and oral cavity cancer	High-middle SDI	42729	56734	46107	54793	7.9	20.3	4.5	32.8
Lip and oral cavity cancer	Middle SDI	58703	93980	64407	78714	9.7	24.4	26	60.1
Lip and oral cavity cancer	Low-middle SDI	63087	95976	74923	83170	18.8	13.1	20.3	52.1
Lip and oral cavity cancer	Low SDI	29269	41778	36334	40044	24.1	12.7	5.9	42.7
Nasopharynx cancer	Global	91222	109781	102977	112020	12.9	9.9	-2.5	20.3
Nasopharynx cancer	High SDI	12978	12798	13751	14777	6	7.9	-15.2	-1.4
Nasopharynx cancer	High-middle SDI	23609	28643	25475	28308	7.9	12	1.4	21.3
Nasopharynx cancer	Middle SDI	32839	41135	36030	41216	9.7	15.8	-0.2	25.3
Nasopharynx cancer	Low-middle SDI	13457	16459	15981	17319	18.8	9.9	-6.4	22.3
Nasopharynx cancer	Low SDI	7621	9884	9460	10170	24.1	9.3	-3.8	29.7
Other pharynx cancer	Global	123808	179326	139763	160459	12.9	16.7	15.2	44.8
Other pharynx cancer	High SDI	39797	49665	42170	46917	6	11.9	6.9	24.8
Other pharynx cancer	High-middle SDI	17396	23803	18770	22505	7.9	21.5	7.5	36.8
Other pharynx cancer	Middle SDI	20427	34384	22412	27735	9.7	26.1	32.5	68.3
Other pharynx cancer	Low-middle SDI	28138	43214	33417	37475	18.8	14.4	20.4	53.6
Other pharynx cancer	Low SDI	17885	27982	22202	24486	24.1	12.8	19.5	56.5
Esophageal cancer	Global	404691	472525	456843	532885	12.9	18.8	-14.9	16.8

Cause	Location	Incidence cases, No.		Expected incidence cases, 2017, No.		Change in incidence cases, 2007 to 2017, %			Overall change, %
		2007	2017	Given population growth alone	Given population growth and aging	Due to population growth	Due to change in age structure	Due to change in incidence rate	
Esophageal cancer	High SDI	79937	92352	84704	97561	6	16.1	-6.5	15.5
Esophageal cancer	High-middle SDI	123504	145072	133266	161932	7.9	23.2	-13.7	17.5
Esophageal cancer	Middle SDI	134257	149107	147305	187944	9.7	30.3	-28.9	11.1
Esophageal cancer	Low-middle SDI	41816	52324	49661	56226	18.8	15.7	-9.3	25.1
Esophageal cancer	Low SDI	21519	29591	26713	29657	24.1	13.7	-0.3	37.5
Stomach cancer	Global	975825	1220662	1101578	1281878	12.9	18.5	-6.3	25.1
Stomach cancer	High SDI	274269	299043	290626	338145	6	17.3	-14.3	9
Stomach cancer	High-middle SDI	294238	382392	317498	381939	7.9	21.9	0.2	30
Stomach cancer	Middle SDI	259682	357404	284920	359140	9.7	28.6	-0.7	37.6
Stomach cancer	Low-middle SDI	89957	108761	106835	120683	18.8	15.4	-13.3	20.9
Stomach cancer	Low SDI	50783	63267	63042	69837	24.1	13.4	-12.9	24.6
Colon and rectum cancer	Global	1331164	1833451	1502709	1762209	12.9	19.5	5.4	37.7
Colon and rectum cancer	High SDI	724649	860022	767867	892171	6	17.2	-4.4	18.7
Colon and rectum cancer	High-middle SDI	291577	447220	314626	379201	7.9	22.1	23.3	53.4
Colon and rectum cancer	Middle SDI	199162	347697	218518	273508	9.7	27.6	37.3	74.6
Colon and rectum cancer	Low-middle SDI	72086	110706	85611	96920	18.8	15.7	19.1	53.6
Colon and rectum cancer	Low SDI	38950	59355	48352	53753	24.1	13.9	14.4	52.4
Liver cancer	Global	704940	953076	795785	912005	12.9	16.5	5.8	35.2
Liver cancer	High SDI	157164	189099	166537	190301	6	15.1	-0.8	20.3
Liver cancer	High-middle SDI	197377	287107	212979	252662	7.9	20.1	17.5	45.5
Liver cancer	Middle SDI	244715	340837	268499	330523	9.7	25.3	4.2	39.3
Liver cancer	Low-middle SDI	65251	84823	77493	86964	18.8	14.5	-3.3	30
Liver cancer	Low SDI	33899	42099	42081	46399	24.1	12.7	-12.7	24.2
Gallbladder and biliary tract cancer	Global	158681	210878	179129	212162	12.9	20.8	-0.8	32.9

 © 2019 Global Burden of Disease Cancer Collaboration. JAMA Oncol.

Cause	Location	Incidence cases, No.		Expected incidence cases, 2017, No.		Change in incidence cases, 2007 to 2017, %			Overall change, %
		2007	2017	Given population growth alone	Given population growth and aging	Due to population growth	Due to change in age structure	Due to change in incidence rate	
Gallbladder and biliary tract cancer	High SDI	73621	94448	78011	92351	6	19.5	2.8	28.3
Gallbladder and biliary tract cancer	High-middle SDI	28220	36134	30450	37228	7.9	24	-3.9	28
Gallbladder and biliary tract cancer	Middle SDI	28144	40177	30879	39348	9.7	30.1	2.9	42.8
Gallbladder and biliary tract cancer	Low-middle SDI	18012	24819	21391	24320	18.8	16.3	2.8	37.8
Gallbladder and biliary tract cancer	Low SDI	10236	14664	12707	14194	24.1	14.5	4.6	43.3
Pancreatic cancer	Global	321292	447665	362696	427701	12.9	20.2	6.2	39.3
Pancreatic cancer	High SDI	165383	210439	175246	204888	6	17.9	3.4	27.2
Pancreatic cancer	High-middle SDI	74693	104712	80598	97815	7.9	23.1	9.2	40.2
Pancreatic cancer	Middle SDI	47477	77874	52091	66062	9.7	29.4	24.9	64
Pancreatic cancer	Low-middle SDI	22777	36617	27050	30735	18.8	16.2	25.8	60.8
Pancreatic cancer	Low SDI	9955	16380	12357	13786	24.1	14.4	26	64.5
Larynx cancer	Global	159493	210606	180047	208823	12.9	18	1.1	32
Larynx cancer	High SDI	50931	57765	53968	61285	6	14.4	-6.9	13.4
Larynx cancer	High-middle SDI	36665	49547	39563	47810	7.9	22.5	4.7	35.1
Larynx cancer	Middle SDI	30851	49053	33848	42583	9.7	28.3	21	59
Larynx cancer	Low-middle SDI	24882	32596	29550	33340	18.8	15.2	-3	31
Larynx cancer	Low SDI	15696	20825	19485	21540	24.1	13.1	-4.6	32.7
Tracheal, bronchus, and lung cancer	Global	1585124	2163132	1789397	2092386	12.9	19.1	4.5	36.5
Tracheal, bronchus, and lung cancer	High SDI	683416	797425	724175	836522	6	16.4	-5.7	16.7
Tracheal, bronchus, and lung cancer	High-middle SDI	422818	622560	456241	552207	7.9	22.7	16.6	47.2

© 2019 Global Burden of Disease Cancer Collaboration. JAMA Oncol.

Cause	Location	Incidence cases, No.		Expected incidence cases, 2017, No.		Change in incidence cases, 2007 to 2017, %			Overall change, %
		2007	2017	Given population growth alone	Given population growth and aging	Due to population growth	Due to change in age structure	Due to change in incidence rate	
Tracheal, bronchus, and lung cancer	Middle SDI	333723	538927	366157	463901	9.7	29.3	22.5	61.5
Tracheal, bronchus, and lung cancer	Low-middle SDI	90553	121201	107544	121777	18.8	15.7	-0.6	33.8
Tracheal, bronchus, and lung cancer	Low SDI	45925	68425	57011	63337	24.1	13.8	11.1	49
Malignant skin melanoma	Global	233617	308684	263723	297053	12.9	14.3	5	32.1
Malignant skin melanoma	High SDI	183371	234273	194307	211575	6	9.4	12.4	27.8
Malignant skin melanoma	High-middle SDI	32911	46195	35512	40481	7.9	15.1	17.4	40.4
Malignant skin melanoma	Middle SDI	10324	17562	11327	13150	9.7	17.7	42.7	70.1
Malignant skin melanoma	Low-middle SDI	4328	6700	5139	5632	18.8	11.4	24.7	54.8
Malignant skin melanoma	Low SDI	2433	3530	3020	3287	24.1	11	10	45.1
Non-melanoma skin cancer	Global	5772527	7663589	6516426	7657115	12.9	19.8	0.1	32.8
Non-melanoma skin cancer	High SDI	4709887	6052504	4990784	5734870	6	15.8	6.7	28.5
Non-melanoma skin cancer	High-middle SDI	420157	602803	453370	545838	7.9	22	13.6	43.5
Non-melanoma skin cancer	Middle SDI	451039	692756	494874	610992	9.7	25.7	18.1	53.6
Non-melanoma skin cancer	Low-middle SDI	124305	221372	147628	164900	18.8	13.9	45.4	78.1
Non-melanoma skin cancer	Low SDI	63845	88481	79257	85848	24.1	10.3	4.1	38.6
Breast cancer	Global	1450335	1960682	1637237	1856152	12.9	15.1	7.2	35.2
Breast cancer	High SDI	696552	781346	738094	819742	6	11.7	-5.5	12.2
Breast cancer	High-middle SDI	301728	435967	325579	379636	7.9	17.9	18.7	44.5
Breast cancer	Middle SDI	254012	418855	278698	329932	9.7	20.2	35	64.9
Breast cancer	Low-middle SDI	140459	222633	166814	184239	18.8	12.4	27.3	58.5
Breast cancer	Low SDI	53064	94422	65873	71924	24.1	11.4	42.4	77.9
Cervical cancer	Global	505740	601186	570913	617377	12.9	9.2	-3.2	18.9
Cervical cancer	High SDI	73104	74634	77464	81638	6	5.7	-9.6	2.1
Cervical cancer	High-middle SDI	91379	102547	98602	110116	7.9	12.6	-8.3	12.2

© 2019 Global Burden of Disease Cancer Collaboration. JAMA Oncol.

Cause	Location	Incidence cases, No.		Expected incidence cases, 2017, No.		Change in incidence cases, 2007 to 2017, %			Overall change, %
		2007	2017	Given population growth alone	Given population growth and aging	Due to population growth	Due to change in age structure	Due to change in incidence rate	
Cervical cancer	Middle SDI	150996	186320	165671	188104	9.7	14.9	-1.2	23.4
Cervical cancer	Low-middle SDI	104512	129180	124122	134864	18.8	10.3	-5.4	23.6
Cervical cancer	Low SDI	83984	106167	104257	112493	24.1	9.8	-7.5	26.4
Uterine cancer	Global	299012	406793	337545	389361	12.9	17.3	5.8	36
Uterine cancer	High SDI	147048	197424	155818	176391	6	14	14.3	34.3
Uterine cancer	High-middle SDI	71130	91360	76753	91323	7.9	20.5	0.1	28.4
Uterine cancer	Middle SDI	53020	75409	58172	70595	9.7	23.4	9.1	42.2
Uterine cancer	Low-middle SDI	19596	30002	23273	26092	18.8	14.4	19.9	53.1
Uterine cancer	Low SDI	7052	11118	8753	9652	24.1	12.7	20.8	57.7
Ovarian cancer	Global	221787	286127	250368	281462	12.9	14	2.1	29
Ovarian cancer	High SDI	87410	91486	92622	103311	6	12.2	-13.5	4.7
Ovarian cancer	High-middle SDI	48609	59262	52452	60431	7.9	16.4	-2.4	21.9
Ovarian cancer	Middle SDI	43838	67615	48098	56055	9.7	18.1	26.4	54.2
Ovarian cancer	Low-middle SDI	29822	47695	35418	38704	18.8	11	30.1	59.9
Ovarian cancer	Low SDI	11541	19192	14327	15580	24.1	10.9	31.3	66.3
Prostate cancer	Global	939980	1334315	1061114	1258544	12.9	21	8.1	42
Prostate cancer	High SDI	587815	743876	622872	730191	6	18.3	2.3	26.5
Prostate cancer	High-middle SDI	142375	236637	153629	188805	7.9	24.7	33.6	66.2
Prostate cancer	Middle SDI	123479	217246	135479	175883	9.7	32.7	33.5	75.9
Prostate cancer	Low-middle SDI	58872	93572	69918	80936	18.8	18.7	21.5	58.9
Prostate cancer	Low SDI	25314	39260	31424	35835	24.1	17.4	13.5	55.1
Testicular cancer	Global	58427	71348	65956	66463	12.9	0.9	8.4	22.1
Testicular cancer	High SDI	33186	34764	35165	33725	6	-4.3	3.1	4.8
Testicular cancer	High-middle SDI	12514	16930	13502	13457	7.9	-0.4	27.8	35.3
Testicular cancer	Middle SDI	8338	13844	9148	9123	9.7	-0.3	56.6	66
Testicular cancer	Low-middle SDI	3387	4464	4023	4157	18.8	4	9	31.8

Cause	Location	Incidence cases, No.		Expected incidence cases, 2017, No.		Change in incidence cases, 2007 to 2017, %			Overall change, %
		2007	2017	Given population growth alone	Given population growth and aging	Due to population growth	Due to change in age structure	Due to change in incidence rate	
Testicular cancer	Low SDI	917	1208	1138	1195	24.1	6.3	1.3	31.7
Kidney cancer	Global	314508	393043	355038	401941	12.9	14.9	-2.8	25
Kidney cancer	High SDI	150974	176880	159977	180428	6	13.5	-2.4	17.2
Kidney cancer	High-middle SDI	72888	89416	78649	92066	7.9	18.4	-3.6	22.7
Kidney cancer	Middle SDI	48644	68749	53371	62527	9.7	18.8	12.8	41.3
Kidney cancer	Low-middle SDI	26047	36589	30934	32980	18.8	7.9	13.9	40.5
Kidney cancer	Low SDI	15168	20346	18829	19064	24.1	1.6	8.4	34.1
Bladder cancer	Global	357786	473800	403893	476186	12.9	20.2	-0.7	32.4
Bladder cancer	High SDI	192602	233055	204088	238898	6	18.1	-3	21
Bladder cancer	High-middle SDI	77922	105718	84081	102008	7.9	23	4.8	35.7
Bladder cancer	Middle SDI	45744	72734	50190	63468	9.7	29	20.3	59
Bladder cancer	Low-middle SDI	29174	43951	34648	39252	18.8	15.8	16.1	50.7
Bladder cancer	Low SDI	11346	16777	14085	15802	24.1	15.1	8.6	47.9
Brain and central nervous system cancer	Global	284977	405218	321701	344801	12.9	8.1	21.2	42.2
Brain and central nervous system cancer	High SDI	97970	127159	103813	110907	6	7.2	16.6	29.8
Brain and central nervous system cancer	High-middle SDI	70200	107805	75749	82600	7.9	9.8	35.9	53.6
Brain and central nervous system cancer	Middle SDI	61091	98199	67028	73997	9.7	11.4	39.6	60.7
Brain and central nervous system cancer	Low-middle SDI	33057	43442	39259	40667	18.8	4.3	8.4	31.4
Brain and central nervous system cancer	Low SDI	21378	26398	26538	26784	24.1	1.1	-1.8	23.5
Thyroid cancer	Global	185433	255489	209329	229100	12.9	10.7	14.2	37.8
Thyroid cancer	High SDI	79748	88070	84503	90190	6	7.1	-2.7	10.4

© 2019 Global Burden of Disease Cancer Collaboration. JAMA Oncol.

Cause	Location	Incidence cases, No.		Expected incidence cases, 2017, No.		Change in incidence cases, 2007 to 2017, %			Overall change, %
		2007	2017	Given population growth alone	Given population growth and aging	Due to population growth	Due to change in age structure	Due to change in incidence rate	
Thyroid cancer	High-middle SDI	39200	58681	42298	47428	7.9	13.1	28.7	49.7
Thyroid cancer	Middle SDI	36064	60932	39568	45061	9.7	15.2	44	69
Thyroid cancer	Low-middle SDI	19871	31536	23599	25207	18.8	8.1	31.8	58.7
Thyroid cancer	Low SDI	10040	15404	12464	13304	24.1	8.4	20.9	53.4
Mesothelioma	Global	27918	34615	31515	36409	12.9	17.5	-6.4	24
Mesothelioma	High SDI	15951	18960	16902	19562	6	16.7	-3.8	18.9
Mesothelioma	High-middle SDI	4435	5381	4785	5627	7.9	19	-5.5	21.3
Mesothelioma	Middle SDI	3766	5143	4132	4961	9.7	22	4.8	36.6
Mesothelioma	Low-middle SDI	2504	3322	2973	3296	18.8	12.9	1	32.7
Mesothelioma	Low SDI	1211	1736	1502	1646	24.1	11.9	7.4	43.4
Hodgkin lymphoma	Global	87794	101133	99108	102166	12.9	3.5	-1.2	15.2
Hodgkin lymphoma	High SDI	34464	34961	36519	36460	6	-0.2	-4.4	1.4
Hodgkin lymphoma	High-middle SDI	20497	26655	22116	22269	7.9	0.7	21.4	30
Hodgkin lymphoma	Middle SDI	11575	16529	12699	13590	9.7	7.7	25.4	42.8
Hodgkin lymphoma	Low-middle SDI	13682	15178	16249	16719	18.8	3.4	-11.3	10.9
Hodgkin lymphoma	Low SDI	7347	7447	9121	9441	24.1	4.4	-27.1	1.4
Non-Hodgkin lymphoma	Global	351576	487964	396883	448602	12.9	14.7	11.2	38.8
Non-Hodgkin lymphoma	High SDI	191484	237182	202903	228860	6	13.6	4.3	23.9
Non-Hodgkin lymphoma	High-middle SDI	54133	91503	58411	66875	7.9	15.6	45.5	69
Non-Hodgkin lymphoma	Middle SDI	48789	81774	53530	62603	9.7	18.6	39.3	67.6
Non-Hodgkin lymphoma	Low-middle SDI	34336	46916	40778	43519	18.8	8	9.9	36.6
Non-Hodgkin lymphoma	Low SDI	21959	28901	27259	28445	24.1	5.4	2.1	31.6
Multiple myeloma	Global	110991	152746	125294	146692	12.9	19.3	5.5	37.6
Multiple myeloma	High SDI	64888	81220	68757	79411	6	16.4	2.8	25.2
Multiple myeloma	High-middle SDI	18038	27453	19463	23436	7.9	22	22.3	52.2
Multiple myeloma	Middle SDI	14511	23630	15920	19865	9.7	27.2	25.9	62.8

Cause	Location	Incidence cases, No.		Expected incidence cases, 2017, No.		Change in incidence cases, 2007 to 2017, %			Overall change, %
		2007	2017	Given population growth alone	Given population growth and aging	Due to population growth	Due to change in age structure	Due to change in incidence rate	
Multiple myeloma	Low-middle SDI	8558	12879	10164	11470	18.8	15.3	16.5	50.5
Multiple myeloma	Low SDI	4737	7129	5880	6532	24.1	13.8	12.6	50.5
Acute lymphoid leukemia	Global	83156	107561	93872	93250	12.9	-0.7	17.2	29.3
Acute lymphoid leukemia	High SDI	16487	16688	17469	17423	6	-0.3	-4.5	1.2
Acute lymphoid leukemia	High-middle SDI	19943	31055	21519	21699	7.9	0.9	46.9	55.7
Acute lymphoid leukemia	Middle SDI	25199	35502	27648	27541	9.7	-0.4	31.6	40.9
Acute lymphoid leukemia	Low-middle SDI	12351	14036	14668	14312	18.8	-2.9	-2.2	13.6
Acute lymphoid leukemia	Low SDI	8775	9557	10892	10429	24.1	-5.3	-9.9	8.9
Chronic lymphoid leukemia	Global	81414	114416	91905	107695	12.9	19.4	8.3	40.5
Chronic lymphoid leukemia	High SDI	54984	63632	58262	67841	6	17.4	-7.7	15.7
Chronic lymphoid leukemia	High-middle SDI	15401	27373	16618	19518	7.9	18.8	51	77.7
Chronic lymphoid leukemia	Middle SDI	7739	17828	8491	10014	9.7	19.7	101	130.4
Chronic lymphoid leukemia	Low-middle SDI	1942	3343	2306	2591	18.8	14.7	38.7	72.1
Chronic lymphoid leukemia	Low SDI	1110	1710	1378	1539	24.1	14.6	15.3	54
Acute myeloid leukemia	Global	113481	139828	128105	138875	12.9	9.5	0.8	23.2
Acute myeloid leukemia	High SDI	44442	52043	47092	52457	6	12.1	-0.9	17.1
Acute myeloid leukemia	High-middle SDI	20337	23309	21944	23685	7.9	8.6	-1.9	14.6
Acute myeloid leukemia	Middle SDI	21525	28401	23617	25595	9.7	9.2	13	31.9
Acute myeloid leukemia	Low-middle SDI	15729	21049	18680	19265	18.8	3.7	11.3	33.8
Acute myeloid leukemia	Low SDI	11136	14648	13824	14013	24.1	1.7	5.7	31.5
Chronic myeloid leukemia	Global	38185	39845	43105	48162	12.9	13.2	-21.8	4.3
Chronic myeloid leukemia	High SDI	14771	12994	15651	17550	6	12.9	-30.8	-12
Chronic myeloid leukemia	High-middle SDI	7021	6843	7575	8531	7.9	13.6	-24.1	-2.5
Chronic myeloid leukemia	Middle SDI	6623	8118	7266	8366	9.7	16.6	-3.8	22.6
Chronic myeloid leukemia	Low-middle SDI	5094	6159	6050	6615	18.8	11.1	-9	20.9
Chronic myeloid leukemia	Low SDI	4582	5633	5687	6174	24.1	10.6	-11.8	22.9

© 2019 Global Burden of Disease Cancer Collaboration. JAMA Oncol.

Cause	Location	Incidence cases, No.		Expected incidence cases, 2017, No.		Change in incidence cases, 2007 to 2017, %			Overall change, %
		2007	2017	Given population growth alone	Given population growth and aging	Due to population growth	Due to change in age structure	Due to change in incidence rate	
Other leukemia	Global	226052	246145	255183	269995	12.9	6.6	-10.6	8.9
Other leukemia	High SDI	41432	45487	43902	49952	6	14.6	-10.8	9.8
Other leukemia	High-middle SDI	68357	73222	73760	78920	7.9	7.5	-8.3	7.1
Other leukemia	Middle SDI	71780	79705	78756	83635	9.7	6.8	-5.5	11
Other leukemia	Low-middle SDI	28595	30982	33960	34751	18.8	2.8	-13.2	8.3
Other leukemia	Low SDI	14076	14789	17474	17343	24.1	-0.9	-18.1	5.1
Other malignant neoplasms	Global	484500	715547	546937	597874	12.9	10.5	24.3	47.7
Other malignant neoplasms	High SDI	165713	208759	175596	192270	6	10.1	10	26
Other malignant neoplasms	High-middle SDI	127378	205510	137447	153445	7.9	12.6	40.9	61.3
Other malignant neoplasms	Middle SDI	89029	164056	97681	111638	9.7	15.7	58.9	84.3
Other malignant neoplasms	Low-middle SDI	59487	80112	70648	74054	18.8	5.7	10.2	34.7
Other malignant neoplasms	Low SDI	40819	52946	50672	52045	24.1	3.4	2.2	29.7

© 2019 Global Burden of Disease Cancer Collaboration. JAMA Oncol.

eTable 15: Contribution of YLDs and YLLs to DALYs by cancer, global, both sexes, 2017

Cause	Contribution YLLs (%)	Contribution YLDs (%)
All Malignant Neoplasms	97	3
Lip and Oral Cavity Cancer	97	3
Nasopharynx Cancer	97	3
Other Pharynx Cancer	98	2
Esophageal Cancer	99	1
Stomach Cancer	98	2
Colon and Rectum Cancer	95	5
Liver Cancer	99	1
Gallbladder and Biliary Tract Cancer	99	1
Pancreatic Cancer	99	1
Larynx Cancer	97	3
Tracheal, Bronchus, and Lung Cancer	99	1
Malignant Skin Melanoma	91	9
Non-Melanoma Skin Cancer	93	7
Non-Melanoma Skin Cancer (Squamous-Cell Carcinoma)	93	7
Non-Melanoma Skin Cancer (Basal-Cell Carcinoma)	0	100
Breast Cancer	93	7
Cervical Cancer	96	4
Uterine Cancer	90	10
Ovarian Cancer	96	4
Prostate Cancer	88	12
Testicular Cancer	90	10
Kidney Cancer	96	4
Bladder Cancer	93	7
Brain and Nervous System Cancer	98	2
Thyroid Cancer	88	12
Mesothelioma	98	2
Hodgkin Lymphoma	96	4
Non-Hodgkin Lymphoma	97	3
Multiple Myeloma	96	4
Acute Lymphoid Leukemia	98	2
Chronic Lymphoid Leukemia	89	11

© 2019 Global Burden of Disease Cancer Collaboration. JAMA Oncol.

Cause	Contribution YLLs (%)	Contribution YLDs (%)
Acute Myeloid Leukemia	99	1
Chronic Myeloid Leukemia	98	2
Other Leukemia	98	2
Other Malignant Neoplasms	94	6

eTable 16: Probability of developing cancer within selected age intervals, global, and by SDI quintile, by sex, 2007-2017 in % (odds)

Location/SDI Quintile	Cancer	Birth to age 49		Age 50 to 59		Age 60 to 69		Age 70 to 79		Age 30 to 70		Birth to age 79	
		Male	Female	Male	Female	Male	Female	Male	Female	Male	Female	Male	Female
Global	Neoplasms	3.18 (1 in 31)	4.55 (1 in 22)	5.61 (1 in 18)	5.18 (1 in 19)	13.79 (1 in 7)	9.02 (1 in 11)	22.60 (1 in 4)	13.08 (1 in 8)	38.62 (1 in 3)	27.90 (1 in 4)	39.02 (1 in 3)	28.43 (1 in 4)
Global	Lip and oral cavity cancer	0.10 (1 in 1025)	0.08 (1 in 1298)	0.16 (1 in 642)	0.08 (1 in 1307)	0.25 (1 in 396)	0.12 (1 in 845)	0.32 (1 in 316)	0.16 (1 in 611)	0.81 (1 in 123)	0.42 (1 in 238)	0.82 (1 in 122)	0.43 (1 in 230)
Global	Nasopharynx cancer	0.06 (1 in 1543)	0.03 (1 in 3941)	0.05 (1 in 1952)	0.02 (1 in 6354)	0.06 (1 in 1568)	0.02 (1 in 5891)	0.06 (1 in 1757)	0.02 (1 in 5737)	0.23 (1 in 439)	0.07 (1 in 1425)	0.24 (1 in 423)	0.08 (1 in 1325)
Global	Other pharynx cancer	0.04 (1 in 2292)	0.02 (1 in 4827)	0.11 (1 in 925)	0.03 (1 in 3062)	0.16 (1 in 615)	0.05 (1 in 2089)	0.15 (1 in 680)	0.05 (1 in 2084)	0.46 (1 in 218)	0.15 (1 in 683)	0.46 (1 in 217)	0.15 (1 in 670)
Global	Esophageal cancer	0.06 (1 in 1787)	0.03 (1 in 3825)	0.17 (1 in 586)	0.05 (1 in 1843)	0.43 (1 in 232)	0.14 (1 in 721)	0.64 (1 in 157)	0.25 (1 in 396)	1.29 (1 in 78)	0.47 (1 in 213)	1.29 (1 in 78)	0.47 (1 in 212)
Global	Stomach cancer	0.15 (1 in 654)	0.12 (1 in 851)	0.35 (1 in 282)	0.16 (1 in 632)	0.99 (1 in 101)	0.36 (1 in 275)	1.58 (1 in 63)	0.65 (1 in 154)	3.04 (1 in 33)	1.27 (1 in 79)	3.05 (1 in 33)	1.28 (1 in 78)
Global	Colon and rectum cancer	0.20 (1 in 492)	0.17 (1 in 581)	0.44 (1 in 226)	0.32 (1 in 308)	1.16 (1 in 86)	0.71 (1 in 140)	2.04 (1 in 49)	1.30 (1 in 77)	3.79 (1 in 26)	2.48 (1 in 40)	3.80 (1 in 26)	2.49 (1 in 40)
Global	Liver cancer	0.26 (1 in 378)	0.07 (1 in 1523)	0.43 (1 in 232)	0.11 (1 in 881)	0.78 (1 in 129)	0.26 (1 in 385)	0.94 (1 in 106)	0.41 (1 in 245)	2.38 (1 in 42)	0.84 (1 in 120)	2.40 (1 in 42)	0.84 (1 in 118)
Global	Gallbladder and biliary tract cancer	0.01 (1 in 8176)	0.02 (1 in 6110)	0.03 (1 in 3169)	0.04 (1 in 2483)	0.10 (1 in 1035)	0.11 (1 in 952)	0.20 (1 in 506)	0.20 (1 in 503)	0.34 (1 in 297)	0.36 (1 in 278)	0.34 (1 in 296)	0.36 (1 in 278)

 © 2019 Global Burden of Disease Cancer Collaboration. JAMA Oncol.

Location/SDI Quintile	Cancer	Birth to age 49		Age 50 to 59		Age 60 to 69		Age 70 to 79		Age 30 to 70		Birth to age 79	
		Male	Female	Male	Female	Male	Female	Male	Female	Male	Female	Male	Female
Global	Pancreatic cancer	0.04 (1 in 2650)	0.03 (1 in 3976)	0.10 (1 in 955)	0.07 (1 in 1436)	0.27 (1 in 371)	0.19 (1 in 520)	0.48 (1 in 210)	0.39 (1 in 257)	0.88 (1 in 113)	0.67 (1 in 149)	0.89 (1 in 113)	0.67 (1 in 148)
Global	Larynx cancer	0.04 (1 in 2587)	0.01 (1 in 9601)	0.12 (1 in 809)	0.02 (1 in 4960)	0.24 (1 in 417)	0.04 (1 in 2816)	0.27 (1 in 369)	0.04 (1 in 2427)	0.67 (1 in 149)	0.11 (1 in 943)	0.67 (1 in 149)	0.11 (1 in 932)
Global	Tracheal, bronchus, and lung cancer	0.18 (1 in 559)	0.11 (1 in 914)	0.64 (1 in 155)	0.28 (1 in 355)	1.93 (1 in 52)	0.73 (1 in 136)	3.10 (1 in 32)	1.21 (1 in 83)	5.75 (1 in 17)	2.31 (1 in 43)	5.76 (1 in 17)	2.32 (1 in 43)
Global	Malignant skin melanoma	0.08 (1 in 1324)	0.10 (1 in 1012)	0.08 (1 in 1272)	0.07 (1 in 1468)	0.14 (1 in 732)	0.10 (1 in 996)	0.23 (1 in 426)	0.15 (1 in 663)	0.51 (1 in 195)	0.40 (1 in 251)	0.52 (1 in 191)	0.42 (1 in 239)
Global	Non-melanoma skin cancer	0.76 (1 in 132)	0.87 (1 in 115)	1.74 (1 in 57)	1.44 (1 in 70)	4.68 (1 in 21)	2.97 (1 in 34)	8.69 (1 in 12)	4.77 (1 in 21)	15.05 (1 in 7)	9.63 (1 in 10)	15.12 (1 in 7)	9.72 (1 in 10)
Global	Breast cancer	0.01 (1 in 12912)	1.14 (1 in 88)	0.01 (1 in 8233)	1.20 (1 in 83)	0.03 (1 in 3636)	1.56 (1 in 64)	0.04 (1 in 2849)	1.71 (1 in 59)	0.08 (1 in 1225)	5.44 (1 in 18)	0.08 (1 in 1212)	5.49 (1 in 18)
Global	Cervical cancer	0	0.59 (1 in 168)	0	0.35 (1 in 289)	0	0.32 (1 in 312)	0	0.28 (1 in 357)	0	1.48 (1 in 68)	0	1.53 (1 in 65)
Global	Uterine cancer	0	0.13 (1 in 745)	0	0.29 (1 in 339)	0	0.43 (1 in 231)	0	0.44 (1 in 230)	0	1.29 (1 in 78)	0	1.29 (1 in 77)
Global	Ovarian cancer	0	0.18 (1 in 569)	0	0.17 (1 in 592)	0	0.22 (1 in 445)	0	0.25 (1 in 392)	0	0.79 (1 in 127)	0	0.82 (1 in 122)
Global	Prostate cancer	0.06 (1 in 1816)	0	0.41 (1 in 242)	0	1.69 (1 in 59)	0	3.43 (1 in 29)	0	5.50 (1 in 18)	0	5.50 (1 in 18)	0
Global	Testicular cancer	0.11 (1 in 935)	0	0.01 (1 in 8254)	0	0.01 (1 in 9551)	0	0.01 (1 in 9075)	0	0.10 (1 in 1046)	0	0.14 (1 in 712)	0

© 2019 Global Burden of Disease Cancer Collaboration. JAMA Oncol.

Location/SDI Quintile	Cancer	Birth to age 49		Age 50 to 59		Age 60 to 69		Age 70 to 79		Age 30 to 70		Birth to age 79	
		Male	Female	Male	Female	Male	Female	Male	Female	Male	Female	Male	Female
Global	Kidney cancer	0.09 (1 in 1081)	0.07 (1 in 1338)	0.14 (1 in 723)	0.07 (1 in 1409)	0.26 (1 in 391)	0.13 (1 in 779)	0.38 (1 in 266)	0.19 (1 in 523)	0.84 (1 in 119)	0.44 (1 in 228)	0.86 (1 in 116)	0.46 (1 in 215)
Global	Bladder cancer	0.05 (1 in 1872)	0.02 (1 in 4961)	0.14 (1 in 717)	0.04 (1 in 2603)	0.39 (1 in 254)	0.09 (1 in 1124)	0.77 (1 in 129)	0.18 (1 in 542)	1.35 (1 in 74)	0.33 (1 in 303)	1.36 (1 in 74)	0.33 (1 in 301)
Global	Brain and central nervous system cancer	0.19 (1 in 531)	0.15 (1 in 646)	0.09 (1 in 1087)	0.07 (1 in 1369)	0.15 (1 in 658)	0.11 (1 in 882)	0.19 (1 in 520)	0.14 (1 in 692)	0.54 (1 in 185)	0.42 (1 in 241)	0.62 (1 in 160)	0.48 (1 in 206)
Global	Thyroid cancer	0.05 (1 in 1918)	0.16 (1 in 636)	0.05 (1 in 1894)	0.10 (1 in 1023)	0.06 (1 in 1749)	0.12 (1 in 852)	0.07 (1 in 1388)	0.11 (1 in 921)	0.23 (1 in 442)	0.45 (1 in 221)	0.23 (1 in 427)	0.48 (1 in 208)
Global	Mesothelioma	0.01 (1 in 19175)	0.00 (1 in 24726)	0.01 (1 in 8471)	0.00 (1 in 22587)	0.02 (1 in 4224)	0.01 (1 in 12207)	0.05 (1 in 1840)	0.01 (1 in 6757)	0.09 (1 in 1059)	0.03 (1 in 3247)	0.10 (1 in 1052)	0.03 (1 in 3179)
Global	Hodgkin lymphoma	0.07 (1 in 1527)	0.05 (1 in 1944)	0.02 (1 in 4584)	0.01 (1 in 10520)	0.03 (1 in 3048)	0.01 (1 in 7985)	0.03 (1 in 3065)	0.02 (1 in 6377)	0.12 (1 in 825)	0.06 (1 in 1621)	0.15 (1 in 655)	0.09 (1 in 1122)
Global	Non-Hodgkin lymphoma	0.14 (1 in 722)	0.09 (1 in 1089)	0.13 (1 in 771)	0.08 (1 in 1200)	0.25 (1 in 402)	0.17 (1 in 593)	0.41 (1 in 244)	0.28 (1 in 362)	0.88 (1 in 114)	0.59 (1 in 170)	0.92 (1 in 108)	0.62 (1 in 162)
Global	Multiple myeloma	0.02 (1 in 5639)	0.01 (1 in 7797)	0.04 (1 in 2402)	0.03 (1 in 3206)	0.09 (1 in 1115)	0.07 (1 in 1465)	0.16 (1 in 627)	0.12 (1 in 844)	0.31 (1 in 326)	0.23 (1 in 436)	0.31 (1 in 324)	0.23 (1 in 434)
Global	Acute lymphoid leukemia	0.04 (1 in 2379)	0.03 (1 in 3165)	0.01 (1 in 10353)	0.01 (1 in 16497)	0.02 (1 in 6216)	0.01 (1 in 11060)	0.02 (1 in 5327)	0.01 (1 in 8953)	0.06 (1 in 1771)	0.03 (1 in 2902)	0.09 (1 in 1156)	0.06 (1 in 1728)
Global	Chronic lymphoid leukemia	0.01 (1 in 6980)	0.01 (1 in 9225)	0.02 (1 in 4431)	0.02 (1 in 6435)	0.05 (1 in 1954)	0.03 (1 in 3165)	0.09 (1 in 1059)	0.05 (1 in 1898)	0.18 (1 in 557)	0.11 (1 in 921)	0.18 (1 in 548)	0.11 (1 in 904)
Global	Acute myeloid leukemia	0.05 (1 in 2127)	0.04 (1 in 2503)	0.02 (1 in 4441)	0.02 (1 in 5929)	0.04 (1 in 2273)	0.03 (1 in 3410)	0.09 (1 in 1128)	0.05 (1 in 1848)	0.18 (1 in 562)	0.12 (1 in 834)	0.20 (1 in 495)	0.14 (1 in 713)

© 2019 Global Burden of Disease Cancer Collaboration. JAMA Oncol.

Location/SDI Quintile	Cancer	Birth to age 49		Age 50 to 59		Age 60 to 69		Age 70 to 79		Age 30 to 70		Birth to age 79	
		Male	Female	Male	Female	Male	Female	Male	Female	Male	Female	Male	Female
Global	Chronic myeloid leukemia	0.01 (1 in 8427)	0.01 (1 in 10182)	0.01 (1 in 12722)	0.01 (1 in 15174)	0.01 (1 in 7911)	0.01 (1 in 11334)	0.03 (1 in 3725)	0.01 (1 in 6796)	0.06 (1 in 1775)	0.04 (1 in 2674)	0.06 (1 in 1689)	0.04 (1 in 2504)
Global	Other leukemia	0.09 (1 in 1101)	0.07 (1 in 1344)	0.03 (1 in 3121)	0.02 (1 in 4140)	0.08 (1 in 1268)	0.05 (1 in 2085)	0.13 (1 in 742)	0.07 (1 in 1353)	0.27 (1 in 364)	0.17 (1 in 592)	0.34 (1 in 298)	0.22 (1 in 454)
Global	Other malignant neoplasms	0.26 (1 in 391)	0.23 (1 in 432)	0.15 (1 in 659)	0.12 (1 in 836)	0.30 (1 in 330)	0.22 (1 in 463)	0.46 (1 in 220)	0.32 (1 in 310)	1.05 (1 in 95)	0.78 (1 in 128)	1.16 (1 in 86)	0.89 (1 in 113)
High-middle SDI	Neoplasms	3.69 (1 in 27)	4.64 (1 in 22)	5.28 (1 in 19)	4.41 (1 in 23)	12.68 (1 in 8)	7.86 (1 in 13)	18.71 (1 in 5)	10.81 (1 in 9)	34.62 (1 in 3)	24.39 (1 in 4)	35.24 (1 in 3)	25.09 (1 in 4)
High-middle SDI	Lip and oral cavity cancer	0.07 (1 in 1513)	0.04 (1 in 2851)	0.12 (1 in 850)	0.04 (1 in 2670)	0.21 (1 in 481)	0.06 (1 in 1622)	0.25 (1 in 394)	0.09 (1 in 1124)	0.64 (1 in 156)	0.22 (1 in 459)	0.64 (1 in 155)	0.22 (1 in 449)
High-middle SDI	Nasopharynx cancer	0.09 (1 in 1138)	0.02 (1 in 4037)	0.06 (1 in 1576)	0.01 (1 in 7315)	0.08 (1 in 1222)	0.02 (1 in 5697)	0.06 (1 in 1664)	0.02 (1 in 5248)	0.28 (1 in 355)	0.07 (1 in 1412)	0.29 (1 in 341)	0.08 (1 in 1333)
High-middle SDI	Other pharynx cancer	0.03 (1 in 3517)	0.01 (1 in 13808)	0.08 (1 in 1219)	0.01 (1 in 9990)	0.12 (1 in 844)	0.02 (1 in 6474)	0.08 (1 in 1205)	0.01 (1 in 6702)	0.31 (1 in 322)	0.05 (1 in 2145)	0.31 (1 in 321)	0.05 (1 in 2100)
High-middle SDI	Esophageal cancer	0.07 (1 in 1440)	0.02 (1 in 4356)	0.22 (1 in 453)	0.05 (1 in 1979)	0.62 (1 in 162)	0.19 (1 in 537)	0.94 (1 in 107)	0.39 (1 in 254)	1.83 (1 in 55)	0.65 (1 in 154)	1.84 (1 in 54)	0.65 (1 in 153)
High-middle SDI	Stomach cancer	0.21 (1 in 465)	0.15 (1 in 661)	0.53 (1 in 188)	0.20 (1 in 499)	1.60 (1 in 63)	0.51 (1 in 196)	2.40 (1 in 42)	0.90 (1 in 111)	4.66 (1 in 21)	1.74 (1 in 57)	4.67 (1 in 21)	1.76 (1 in 57)
High-middle SDI	Colon and rectum cancer	0.26 (1 in 383)	0.22 (1 in 461)	0.52 (1 in 191)	0.37 (1 in 274)	1.40 (1 in 71)	0.87 (1 in 115)	2.26 (1 in 44)	1.44 (1 in 70)	4.36 (1 in 23)	2.84 (1 in 35)	4.38 (1 in 23)	2.86 (1 in 35)
High-middle SDI	Liver cancer	0.43 (1 in 232)	0.08 (1 in 1190)	0.62 (1 in 162)	0.14 (1 in 716)	1.08 (1 in 92)	0.34 (1 in 297)	1.18 (1 in 84)	0.48 (1 in 209)	3.25 (1 in 31)	1.03 (1 in 97)	3.27 (1 in 31)	1.04 (1 in 96)
High-middle SDI	Gallbladder and biliary tract cancer	0.01 (1 in 8051)	0.01 (1 in 6922)	0.03 (1 in 3702)	0.03 (1 in 3117)	0.08 (1 in 1258)	0.09 (1 in 1103)	0.15 (1 in 650)	0.17 (1 in 589)	0.27 (1 in 368)	0.31 (1 in 327)	0.27 (1 in 367)	0.31 (1 in 326)

© 2019 Global Burden of Disease Cancer Collaboration. JAMA Oncol.

Location/SDI Quintile	Cancer	Birth to age 49		Age 50 to 59		Age 60 to 69		Age 70 to 79		Age 30 to 70		Birth to age 79	
		Male	Female	Male	Female	Male	Female	Male	Female	Male	Female	Male	Female
High-middle SDI	Pancreatic cancer	0.05 (1 in 1903)	0.03 (1 in 3333)	0.13 (1 in 770)	0.07 (1 in 1337)	0.32 (1 in 308)	0.21 (1 in 467)	0.50 (1 in 199)	0.40 (1 in 252)	1.00 (1 in 100)	0.71 (1 in 140)	1.01 (1 in 99)	0.71 (1 in 140)
High-middle SDI	Larynx cancer	0.04 (1 in 2337)	0.01 (1 in 12849)	0.14 (1 in 725)	0.01 (1 in 7111)	0.27 (1 in 365)	0.03 (1 in 3178)	0.28 (1 in 351)	0.04 (1 in 2600)	0.74 (1 in 136)	0.09 (1 in 1102)	0.74 (1 in 136)	0.09 (1 in 1090)
High-middle SDI	Tracheal, bronchus, and lung cancer	0.26 (1 in 380)	0.15 (1 in 660)	0.93 (1 in 108)	0.32 (1 in 310)	2.75 (1 in 36)	0.94 (1 in 107)	4.03 (1 in 25)	1.43 (1 in 70)	7.77 (1 in 13)	2.81 (1 in 36)	7.78 (1 in 13)	2.82 (1 in 35)
High-middle SDI	Malignant skin melanoma	0.07 (1 in 1460)	0.09 (1 in 1078)	0.05 (1 in 1912)	0.05 (1 in 1953)	0.07 (1 in 1395)	0.07 (1 in 1360)	0.10 (1 in 998)	0.10 (1 in 984)	0.28 (1 in 357)	0.30 (1 in 335)	0.29 (1 in 342)	0.32 (1 in 314)
High-middle SDI	Non-melanoma skin cancer	0.38 (1 in 264)	0.43 (1 in 232)	0.62 (1 in 161)	0.59 (1 in 169)	1.44 (1 in 69)	1.22 (1 in 82)	2.46 (1 in 41)	2.03 (1 in 49)	4.76 (1 in 21)	4.14 (1 in 24)	4.82 (1 in 21)	4.21 (1 in 24)
High-middle SDI	Breast cancer	0.01 (1 in 13006)	1.27 (1 in 78)	0.01 (1 in 8278)	1.21 (1 in 82)	0.03 (1 in 3464)	1.54 (1 in 65)	0.03 (1 in 3151)	1.53 (1 in 65)	0.08 (1 in 1261)	5.39 (1 in 19)	0.08 (1 in 1245)	5.45 (1 in 18)
High-middle SDI	Cervical cancer	0	0.46 (1 in 216)	0	0.25 (1 in 397)	0	0.26 (1 in 383)	0	0.23 (1 in 426)	0	1.16 (1 in 86)	0	1.21 (1 in 83)
High-middle SDI	Uterine cancer	0	0.17 (1 in 603)	0	0.31 (1 in 322)	0	0.42 (1 in 240)	0	0.36 (1 in 276)	0	1.24 (1 in 81)	0	1.25 (1 in 80)
High-middle SDI	Ovarian cancer	0	0.17 (1 in 574)	0	0.16 (1 in 621)	0	0.22 (1 in 453)	0	0.23 (1 in 437)	0	0.75 (1 in 133)	0	0.78 (1 in 128)
High-middle SDI	Prostate cancer	0.06 (1 in 1609)	0	0.34 (1 in 294)	0	1.40 (1 in 71)	0	2.90 (1 in 34)	0	4.65 (1 in 22)	0	4.65 (1 in 21)	0
High-middle SDI	Testicular cancer	0.13 (1 in 794)	0	0.01 (1 in 8997)	0	0.01 (1 in 7240)	0	0.02 (1 in 6113)	0	0.11 (1 in 938)	0	0.17 (1 in 598)	0

 © 2019 Global Burden of Disease Cancer Collaboration. JAMA Oncol.

Location/SDI Quintile	Cancer	Birth to age 49		Age 50 to 59		Age 60 to 69		Age 70 to 79		Age 30 to 70		Birth to age 79	
		Male	Female	Male	Female	Male	Female	Male	Female	Male	Female	Male	Female
High-middle SDI	Kidney cancer	0.11 (1 in 923)	0.08 (1 in 1181)	0.16 (1 in 630)	0.07 (1 in 1334)	0.28 (1 in 360)	0.15 (1 in 683)	0.34 (1 in 291)	0.20 (1 in 503)	0.86 (1 in 116)	0.47 (1 in 212)	0.88 (1 in 113)	0.50 (1 in 198)
High-middle SDI	Bladder cancer	0.07 (1 in 1509)	0.02 (1 in 4587)	0.16 (1 in 625)	0.04 (1 in 2751)	0.45 (1 in 221)	0.09 (1 in 1118)	0.82 (1 in 122)	0.18 (1 in 567)	1.49 (1 in 67)	0.32 (1 in 311)	1.49 (1 in 67)	0.32 (1 in 309)
High-middle SDI	Brain and central nervous system cancer	0.31 (1 in 326)	0.25 (1 in 394)	0.10 (1 in 973)	0.08 (1 in 1196)	0.17 (1 in 579)	0.13 (1 in 779)	0.20 (1 in 499)	0.15 (1 in 660)	0.62 (1 in 163)	0.48 (1 in 206)	0.78 (1 in 128)	0.62 (1 in 162)
High-middle SDI	Thyroid cancer	0.07 (1 in 1487)	0.18 (1 in 571)	0.06 (1 in 1787)	0.10 (1 in 953)	0.04 (1 in 2252)	0.12 (1 in 802)	0.06 (1 in 1708)	0.10 (1 in 990)	0.22 (1 in 463)	0.48 (1 in 208)	0.23 (1 in 443)	0.50 (1 in 198)
High-middle SDI	Mesothelioma	0.01 (1 in 15938)	0.00 (1 in 26547)	0.01 (1 in 8883)	0.00 (1 in 21858)	0.01 (1 in 7418)	0.01 (1 in 11582)	0.02 (1 in 4770)	0.01 (1 in 8174)	0.05 (1 in 1953)	0.03 (1 in 3498)	0.05 (1 in 1924)	0.03 (1 in 3424)
High-middle SDI	Hodgkin lymphoma	0.09 (1 in 1094)	0.09 (1 in 1156)	0.02 (1 in 4099)	0.01 (1 in 9321)	0.04 (1 in 2544)	0.01 (1 in 7692)	0.03 (1 in 3078)	0.01 (1 in 7130)	0.14 (1 in 729)	0.07 (1 in 1344)	0.19 (1 in 533)	0.12 (1 in 805)
High-middle SDI	Non-Hodgkin lymphoma	0.16 (1 in 621)	0.11 (1 in 904)	0.13 (1 in 791)	0.08 (1 in 1308)	0.22 (1 in 449)	0.15 (1 in 665)	0.29 (1 in 346)	0.20 (1 in 506)	0.74 (1 in 135)	0.49 (1 in 202)	0.80 (1 in 125)	0.53 (1 in 187)
High-middle SDI	Multiple myeloma	0.02 (1 in 4792)	0.01 (1 in 7088)	0.04 (1 in 2443)	0.03 (1 in 3348)	0.08 (1 in 1218)	0.06 (1 in 1656)	0.11 (1 in 912)	0.08 (1 in 1203)	0.25 (1 in 399)	0.19 (1 in 540)	0.25 (1 in 395)	0.19 (1 in 534)
High-middle SDI	Acute lymphoid leukemia	0.05 (1 in 2027)	0.03 (1 in 2961)	0.01 (1 in 9284)	0.01 (1 in 14554)	0.02 (1 in 5153)	0.01 (1 in 9219)	0.02 (1 in 4358)	0.01 (1 in 7606)	0.07 (1 in 1493)	0.04 (1 in 2499)	0.10 (1 in 976)	0.06 (1 in 1547)
High-middle SDI	Chronic lymphoid leukemia	0.03 (1 in 3561)	0.02 (1 in 4831)	0.03 (1 in 3175)	0.02 (1 in 4527)	0.06 (1 in 1678)	0.04 (1 in 2469)	0.08 (1 in 1262)	0.05 (1 in 2076)	0.19 (1 in 521)	0.13 (1 in 787)	0.20 (1 in 504)	0.13 (1 in 761)
High-middle SDI	Acute myeloid leukemia	0.05 (1 in 1975)	0.04 (1 in 2260)	0.02 (1 in 5381)	0.01 (1 in 7318)	0.03 (1 in 2912)	0.02 (1 in 4581)	0.06 (1 in 1804)	0.03 (1 in 3445)	0.13 (1 in 759)	0.08 (1 in 1188)	0.16 (1 in 629)	0.11 (1 in 920)

© 2019 Global Burden of Disease Cancer Collaboration. JAMA Oncol.

Location/SDI Quintile	Cancer	Birth to age 49		Age 50 to 59		Age 60 to 69		Age 70 to 79		Age 30 to 70		Birth to age 79	
		Male	Female	Male	Female	Male	Female	Male	Female	Male	Female	Male	Female
High-middle SDI	Chronic myeloid leukemia	0.01 (1 in 8472)	0.01 (1 in 13849)	0.01 (1 in 13881)	0.00 (1 in 21501)	0.01 (1 in 8180)	0.01 (1 in 14812)	0.02 (1 in 4878)	0.01 (1 in 10156)	0.05 (1 in 2043)	0.03 (1 in 3749)	0.05 (1 in 1933)	0.03 (1 in 3513)
High-middle SDI	Other leukemia	0.20 (1 in 504)	0.15 (1 in 673)	0.05 (1 in 2145)	0.03 (1 in 3135)	0.12 (1 in 822)	0.07 (1 in 1518)	0.18 (1 in 553)	0.08 (1 in 1201)	0.39 (1 in 254)	0.21 (1 in 471)	0.55 (1 in 183)	0.33 (1 in 304)
High-middle SDI	Other malignant neoplasms	0.41 (1 in 242)	0.38 (1 in 262)	0.20 (1 in 502)	0.16 (1 in 642)	0.42 (1 in 239)	0.29 (1 in 348)	0.59 (1 in 169)	0.40 (1 in 250)	1.42 (1 in 70)	1.04 (1 in 96)	1.61 (1 in 62)	1.22 (1 in 82)
High SDI	Neoplasms	6.29 (1 in 16)	8.42 (1 in 12)	11.77 (1 in 8)	10.41 (1 in 10)	26.46 (1 in 4)	17.30 (1 in 6)	39.92 (1 in 3)	23.21 (1 in 4)	63.06 (1 in 2)	47.30 (1 in 2)	63.47 (1 in 2)	47.90 (1 in 2)
High SDI	Lip and oral cavity cancer	0.10 (1 in 977)	0.05 (1 in 2157)	0.20 (1 in 496)	0.06 (1 in 1545)	0.31 (1 in 326)	0.10 (1 in 975)	0.35 (1 in 284)	0.15 (1 in 646)	0.95 (1 in 105)	0.36 (1 in 276)	0.96 (1 in 104)	0.37 (1 in 272)
High SDI	Nasopharynx cancer	0.04 (1 in 2826)	0.01 (1 in 8970)	0.03 (1 in 3438)	0.01 (1 in 11484)	0.04 (1 in 2666)	0.01 (1 in 13981)	0.04 (1 in 2548)	0.01 (1 in 15063)	0.14 (1 in 737)	0.03 (1 in 3210)	0.14 (1 in 708)	0.03 (1 in 2972)
High SDI	Other pharynx cancer	0.05 (1 in 2019)	0.01 (1 in 8823)	0.16 (1 in 615)	0.03 (1 in 3365)	0.23 (1 in 437)	0.04 (1 in 2259)	0.18 (1 in 570)	0.04 (1 in 2666)	0.61 (1 in 163)	0.12 (1 in 820)	0.62 (1 in 163)	0.12 (1 in 815)
High SDI	Esophageal cancer	0.05 (1 in 2026)	0.01 (1 in 7776)	0.16 (1 in 634)	0.03 (1 in 3245)	0.35 (1 in 286)	0.06 (1 in 1578)	0.51 (1 in 196)	0.11 (1 in 933)	1.06 (1 in 94)	0.21 (1 in 469)	1.06 (1 in 94)	0.21 (1 in 467)
High SDI	Stomach cancer	0.13 (1 in 774)	0.10 (1 in 1016)	0.28 (1 in 353)	0.12 (1 in 817)	0.72 (1 in 139)	0.25 (1 in 394)	1.48 (1 in 67)	0.55 (1 in 183)	2.59 (1 in 39)	1.01 (1 in 99)	2.60 (1 in 39)	1.02 (1 in 98)
High SDI	Colon and rectum cancer	0.36 (1 in 278)	0.30 (1 in 328)	0.85 (1 in 118)	0.58 (1 in 173)	2.03 (1 in 49)	1.13 (1 in 88)	3.55 (1 in 28)	2.05 (1 in 49)	6.62 (1 in 15)	4.00 (1 in 25)	6.64 (1 in 15)	4.02 (1 in 25)
High SDI	Liver cancer	0.13 (1 in 771)	0.04 (1 in 2506)	0.32 (1 in 310)	0.08 (1 in 1333)	0.59 (1 in 171)	0.16 (1 in 610)	0.87 (1 in 115)	0.34 (1 in 292)	1.89 (1 in 53)	0.61 (1 in 163)	1.90 (1 in 53)	0.62 (1 in 161)
High SDI	Gallbladder and biliary tract cancer	0.02 (1 in 6123)	0.01 (1 in 6753)	0.05 (1 in 2155)	0.04 (1 in 2683)	0.17 (1 in 577)	0.12 (1 in 815)	0.35 (1 in 285)	0.27 (1 in 372)	0.58 (1 in 171)	0.44 (1 in 226)	0.59 (1 in 171)	0.44 (1 in 226)

 © 2019 Global Burden of Disease Cancer Collaboration. JAMA Oncol.

Location/SDI Quintile	Cancer	Birth to age 49		Age 50 to 59		Age 60 to 69		Age 70 to 79		Age 30 to 70		Birth to age 79	
		Male	Female	Male	Female	Male	Female	Male	Female	Male	Female	Male	Female
High SDI	Pancreatic cancer	0.06 (1 in 1800)	0.04 (1 in 2765)	0.17 (1 in 572)	0.11 (1 in 896)	0.45 (1 in 223)	0.30 (1 in 329)	0.82 (1 in 123)	0.63 (1 in 158)	1.49 (1 in 67)	1.08 (1 in 93)	1.49 (1 in 67)	1.08 (1 in 92)
High SDI	Larynx cancer	0.03 (1 in 2941)	0.01 (1 in 14111)	0.14 (1 in 696)	0.02 (1 in 4745)	0.28 (1 in 357)	0.03 (1 in 3072)	0.32 (1 in 312)	0.03 (1 in 3030)	0.77 (1 in 129)	0.09 (1 in 1078)	0.78 (1 in 129)	0.09 (1 in 1067)
High SDI	Tracheal, bronchus, and lung cancer	0.18 (1 in 547)	0.16 (1 in 632)	0.81 (1 in 124)	0.51 (1 in 194)	2.31 (1 in 43)	1.16 (1 in 86)	4.04 (1 in 25)	1.84 (1 in 54)	7.18 (1 in 14)	3.63 (1 in 28)	7.18 (1 in 14)	3.63 (1 in 28)
High SDI	Malignant skin melanoma	0.34 (1 in 298)	0.46 (1 in 216)	0.28 (1 in 359)	0.24 (1 in 412)	0.45 (1 in 223)	0.30 (1 in 332)	0.67 (1 in 148)	0.39 (1 in 260)	1.66 (1 in 60)	1.29 (1 in 78)	1.73 (1 in 58)	1.39 (1 in 72)
High SDI	Non-melanoma skin cancer	2.72 (1 in 37)	3.29 (1 in 30)	6.22 (1 in 16)	5.02 (1 in 20)	14.72 (1 in 7)	9.00 (1 in 11)	22.94 (1 in 4)	12.08 (1 in 8)	39.92 (1 in 3)	26.29 (1 in 4)	40.04 (1 in 2)	26.51 (1 in 4)
High SDI	Breast cancer	0.01 (1 in 10251)	1.87 (1 in 53)	0.02 (1 in 6620)	2.03 (1 in 49)	0.04 (1 in 2721)	2.75 (1 in 36)	0.05 (1 in 1873)	2.97 (1 in 34)	0.11 (1 in 884)	9.22 (1 in 11)	0.11 (1 in 870)	9.29 (1 in 11)
High SDI	Cervical cancer	0	0.40 (1 in 250)	0	0.18 (1 in 560)	0	0.18 (1 in 562)	0	0.19 (1 in 517)	0	0.90 (1 in 111)	0	0.95 (1 in 106)
High SDI	Uterine cancer	0	0.24 (1 in 420)	0	0.60 (1 in 168)	0	0.91 (1 in 110)	0	0.88 (1 in 114)	0	2.59 (1 in 39)	0	2.60 (1 in 38)
High SDI	Ovarian cancer	0	0.22 (1 in 461)	0	0.22 (1 in 452)	0	0.31 (1 in 320)	0	0.38 (1 in 260)	0	1.09 (1 in 91)	0	1.13 (1 in 88)
High SDI	Prostate cancer	0.13 (1 in 773)	0	1.08 (1 in 92)	0	3.77 (1 in 27)	0	6.21 (1 in 16)	0	10.83 (1 in 9)	0	10.84 (1 in 9)	0
High SDI	Testicular cancer	0.39 (1 in 260)	0	0.04 (1 in 2810)	0	0.02 (1 in 5353)	0	0.01 (1 in 7518)	0	0.29 (1 in 340)	0	0.45 (1 in 221)	0
High SDI	Kidney cancer	0.16 (1 in 620)	0.11 (1 in 950)	0.28 (1 in 362)	0.12 (1 in 810)	0.52 (1 in 194)	0.23 (1 in 443)	0.76 (1 in 131)	0.35 (1 in 285)	1.68 (1 in 59)	0.77 (1 in 129)	1.71 (1 in 59)	0.80 (1 in 124)

 © 2019 Global Burden of Disease Cancer Collaboration. JAMA Oncol.

Location/SDI Quintile	Cancer	Birth to age 49		Age 50 to 59		Age 60 to 69		Age 70 to 79		Age 30 to 70		Birth to age 79	
		Male	Female	Male	Female	Male	Female	Male	Female	Male	Female	Male	Female
High SDI	Bladder cancer	0.08 (1 in 1278)	0.03 (1 in 3391)	0.26 (1 in 383)	0.07 (1 in 1521)	0.71 (1 in 142)	0.15 (1 in 673)	1.37 (1 in 73)	0.30 (1 in 337)	2.40 (1 in 42)	0.54 (1 in 186)	2.40 (1 in 42)	0.54 (1 in 185)
High SDI	Brain and central nervous system cancer	0.34 (1 in 295)	0.27 (1 in 370)	0.14 (1 in 707)	0.11 (1 in 897)	0.22 (1 in 452)	0.17 (1 in 603)	0.29 (1 in 339)	0.22 (1 in 459)	0.85 (1 in 118)	0.64 (1 in 156)	0.99 (1 in 101)	0.76 (1 in 131)
High SDI	Thyroid cancer	0.09 (1 in 1055)	0.24 (1 in 417)	0.10 (1 in 965)	0.16 (1 in 629)	0.12 (1 in 843)	0.19 (1 in 519)	0.12 (1 in 827)	0.17 (1 in 587)	0.43 (1 in 234)	0.73 (1 in 136)	0.44 (1 in 229)	0.76 (1 in 132)
High SDI	Mesothelioma	0.01 (1 in 18257)	0.00 (1 in 33978)	0.02 (1 in 5194)	0.01 (1 in 17275)	0.06 (1 in 1625)	0.01 (1 in 7193)	0.14 (1 in 712)	0.03 (1 in 3716)	0.23 (1 in 442)	0.05 (1 in 2034)	0.23 (1 in 441)	0.05 (1 in 2019)
High SDI	Hodgkin lymphoma	0.17 (1 in 597)	0.13 (1 in 775)	0.04 (1 in 2521)	0.02 (1 in 5449)	0.04 (1 in 2377)	0.02 (1 in 4731)	0.04 (1 in 2450)	0.02 (1 in 4254)	0.21 (1 in 470)	0.12 (1 in 816)	0.29 (1 in 345)	0.19 (1 in 521)
High SDI	Non-Hodgkin lymphoma	0.26 (1 in 382)	0.18 (1 in 569)	0.26 (1 in 384)	0.18 (1 in 561)	0.49 (1 in 205)	0.34 (1 in 298)	0.85 (1 in 118)	0.56 (1 in 178)	1.78 (1 in 56)	1.20 (1 in 83)	1.85 (1 in 54)	1.25 (1 in 80)
High SDI	Multiple myeloma	0.03 (1 in 2928)	0.02 (1 in 4896)	0.09 (1 in 1128)	0.06 (1 in 1767)	0.18 (1 in 555)	0.12 (1 in 819)	0.34 (1 in 294)	0.22 (1 in 447)	0.64 (1 in 156)	0.42 (1 in 237)	0.64 (1 in 156)	0.42 (1 in 237)
High SDI	Acute lymphoid leukemia	0.03 (1 in 3724)	0.02 (1 in 5617)	0.01 (1 in 13621)	0.00 (1 in 22475)	0.01 (1 in 8383)	0.01 (1 in 14547)	0.02 (1 in 6507)	0.01 (1 in 10341)	0.04 (1 in 2296)	0.03 (1 in 3750)	0.06 (1 in 1627)	0.04 (1 in 2578)
High SDI	Chronic lymphoid leukemia	0.02 (1 in 5303)	0.01 (1 in 7521)	0.05 (1 in 2050)	0.03 (1 in 3323)	0.11 (1 in 895)	0.06 (1 in 1636)	0.21 (1 in 470)	0.11 (1 in 891)	0.39 (1 in 257)	0.21 (1 in 466)	0.39 (1 in 255)	0.22 (1 in 462)
High SDI	Acute myeloid leukemia	0.05 (1 in 1918)	0.05 (1 in 2070)	0.03 (1 in 3011)	0.03 (1 in 3500)	0.08 (1 in 1318)	0.05 (1 in 1921)	0.18 (1 in 567)	0.11 (1 in 940)	0.32 (1 in 315)	0.22 (1 in 460)	0.34 (1 in 296)	0.24 (1 in 425)
High SDI	Chronic myeloid leukemia	0.01 (1 in 6673)	0.01 (1 in 11058)	0.01 (1 in 10111)	0.01 (1 in 17175)	0.01 (1 in 6816)	0.01 (1 in 11748)	0.04 (1 in 2497)	0.02 (1 in 5382)	0.08 (1 in 1308)	0.04 (1 in 2494)	0.08 (1 in 1257)	0.04 (1 in 2384)

© 2019 Global Burden of Disease Cancer Collaboration. JAMA Oncol.

Location/SDI Quintile	Cancer	Birth to age 49		Age 50 to 59		Age 60 to 69		Age 70 to 79		Age 30 to 70		Birth to age 79	
		Male	Female	Male	Female	Male	Female	Male	Female	Male	Female	Male	Female
High SDI	Other leukemia	0.05 (1 in 1956)	0.04 (1 in 2814)	0.02 (1 in 4164)	0.02 (1 in 6167)	0.07 (1 in 1519)	0.04 (1 in 2498)	0.15 (1 in 648)	0.08 (1 in 1221)	0.27 (1 in 377)	0.15 (1 in 656)	0.30 (1 in 339)	0.17 (1 in 576)
High SDI	Other malignant neoplasms	0.43 (1 in 234)	0.40 (1 in 252)	0.18 (1 in 542)	0.16 (1 in 645)	0.31 (1 in 319)	0.26 (1 in 382)	0.52 (1 in 192)	0.41 (1 in 245)	1.25 (1 in 80)	1.04 (1 in 97)	1.44 (1 in 69)	1.22 (1 in 82)
Low-middle SDI	Neoplasms	1.88 (1 in 53)	3.54 (1 in 28)	2.71 (1 in 37)	3.36 (1 in 30)	5.69 (1 in 18)	4.74 (1 in 21)	8.84 (1 in 11)	6.10 (1 in 16)	17.55 (1 in 6)	16.04 (1 in 6)	17.92 (1 in 6)	16.61 (1 in 6)
Low-middle SDI	Lip and oral cavity cancer	0.13 (1 in 746)	0.17 (1 in 587)	0.19 (1 in 513)	0.16 (1 in 617)	0.30 (1 in 331)	0.26 (1 in 383)	0.36 (1 in 278)	0.30 (1 in 332)	0.98 (1 in 102)	0.85 (1 in 117)	0.99 (1 in 101)	0.89 (1 in 112)
Low-middle SDI	Nasopharynx cancer	0.05 (1 in 2169)	0.03 (1 in 3689)	0.04 (1 in 2410)	0.02 (1 in 5111)	0.05 (1 in 2024)	0.02 (1 in 5058)	0.05 (1 in 1934)	0.02 (1 in 5096)	0.18 (1 in 551)	0.08 (1 in 1259)	0.19 (1 in 530)	0.09 (1 in 1162)
Low-middle SDI	Other pharynx cancer	0.06 (1 in 1663)	0.04 (1 in 2429)	0.13 (1 in 774)	0.07 (1 in 1499)	0.21 (1 in 477)	0.11 (1 in 937)	0.21 (1 in 485)	0.11 (1 in 896)	0.60 (1 in 166)	0.32 (1 in 312)	0.60 (1 in 166)	0.33 (1 in 307)
Low-middle SDI	Esophageal cancer	0.05 (1 in 1892)	0.04 (1 in 2792)	0.14 (1 in 710)	0.07 (1 in 1360)	0.26 (1 in 391)	0.13 (1 in 799)	0.34 (1 in 292)	0.22 (1 in 449)	0.79 (1 in 127)	0.45 (1 in 221)	0.79 (1 in 127)	0.46 (1 in 219)
Low-middle SDI	Stomach cancer	0.10 (1 in 995)	0.10 (1 in 1030)	0.22 (1 in 462)	0.14 (1 in 731)	0.52 (1 in 191)	0.28 (1 in 361)	0.79 (1 in 126)	0.46 (1 in 219)	1.62 (1 in 62)	0.96 (1 in 105)	1.63 (1 in 62)	0.96 (1 in 104)
Low-middle SDI	Colon and rectum cancer	0.10 (1 in 1009)	0.10 (1 in 962)	0.18 (1 in 548)	0.18 (1 in 566)	0.42 (1 in 238)	0.32 (1 in 311)	0.70 (1 in 142)	0.59 (1 in 170)	1.39 (1 in 72)	1.18 (1 in 85)	1.40 (1 in 71)	1.19 (1 in 84)
Low-middle SDI	Liver cancer	0.12 (1 in 816)	0.06 (1 in 1815)	0.23 (1 in 431)	0.09 (1 in 1074)	0.43 (1 in 234)	0.19 (1 in 528)	0.54 (1 in 184)	0.30 (1 in 338)	1.31 (1 in 76)	0.62 (1 in 160)	1.32 (1 in 76)	0.63 (1 in 158)
Low-middle SDI	Gallbladder and biliary tract cancer	0.01 (1 in 9397)	0.02 (1 in 4618)	0.03 (1 in 3679)	0.06 (1 in 1812)	0.06 (1 in 1549)	0.12 (1 in 802)	0.11 (1 in 886)	0.19 (1 in 537)	0.21 (1 in 466)	0.39 (1 in 259)	0.22 (1 in 465)	0.39 (1 in 258)
Low-middle SDI	Pancreatic cancer	0.03 (1 in 3824)	0.02 (1 in 4622)	0.07 (1 in 1529)	0.05 (1 in 1821)	0.15 (1 in 665)	0.13 (1 in 759)	0.24 (1 in 415)	0.23 (1 in 440)	0.48 (1 in 208)	0.43 (1 in 231)	0.48 (1 in 207)	0.43 (1 in 230)

© 2019 Global Burden of Disease Cancer Collaboration. JAMA Oncol.

Location/SDI Quintile	Cancer	Birth to age 49		Age 50 to 59		Age 60 to 69		Age 70 to 79		Age 30 to 70		Birth to age 79	
		Male	Female	Male	Female	Male	Female	Male	Female	Male	Female	Male	Female
Low-middle SDI	Larynx cancer	0.05 (1 in 2201)	0.01 (1 in 6742)	0.13 (1 in 797)	0.03 (1 in 3680)	0.22 (1 in 457)	0.04 (1 in 2239)	0.23 (1 in 433)	0.05 (1 in 2002)	0.62 (1 in 162)	0.13 (1 in 742)	0.62 (1 in 161)	0.14 (1 in 732)
Low-middle SDI	Tracheal, bronchus, and lung cancer	0.10 (1 in 965)	0.06 (1 in 1715)	0.31 (1 in 324)	0.12 (1 in 861)	0.76 (1 in 131)	0.23 (1 in 426)	1.16 (1 in 86)	0.39 (1 in 259)	2.31 (1 in 43)	0.79 (1 in 127)	2.32 (1 in 43)	0.79 (1 in 126)
Low-middle SDI	Malignant skin melanoma	0.01 (1 in 8374)	0.02 (1 in 6227)	0.01 (1 in 9218)	0.01 (1 in 11106)	0.01 (1 in 7471)	0.01 (1 in 7957)	0.02 (1 in 4762)	0.02 (1 in 5321)	0.05 (1 in 1820)	0.05 (1 in 1926)	0.06 (1 in 1749)	0.06 (1 in 1773)
Low-middle SDI	Non-melanoma skin cancer	0.30 (1 in 332)	0.31 (1 in 323)	0.34 (1 in 293)	0.31 (1 in 321)	0.69 (1 in 144)	0.55 (1 in 182)	1.15 (1 in 87)	0.83 (1 in 121)	2.40 (1 in 42)	1.93 (1 in 52)	2.46 (1 in 41)	1.99 (1 in 50)
Low-middle SDI	Breast cancer	0.01 (1 in 15674)	0.94 (1 in 106)	0.01 (1 in 10377)	0.91 (1 in 110)	0.02 (1 in 5750)	0.95 (1 in 105)	0.02 (1 in 4455)	0.93 (1 in 108)	0.06 (1 in 1807)	3.61 (1 in 28)	0.06 (1 in 1791)	3.68 (1 in 27)
Low-middle SDI	Cervical cancer	0	0.69 (1 in 145)	0	0.46 (1 in 219)	0	0.41 (1 in 243)	0	0.34 (1 in 291)	0	1.83 (1 in 55)	0	1.89 (1 in 53)
Low-middle SDI	Uterine cancer	0	0.08 (1 in 1275)	0	0.14 (1 in 704)	0	0.20 (1 in 507)	0	0.18 (1 in 547)	0	0.59 (1 in 168)	0	0.60 (1 in 167)
Low-middle SDI	Ovarian cancer	0	0.20 (1 in 511)	0	0.18 (1 in 566)	0	0.21 (1 in 466)	0	0.21 (1 in 484)	0	0.75 (1 in 134)	0	0.79 (1 in 126)
Low-middle SDI	Prostate cancer	0.03 (1 in 3452)	0	0.17 (1 in 603)	0	0.74 (1 in 136)	0	1.86 (1 in 54)	0	2.77 (1 in 36)	0	2.77 (1 in 36)	0
Low-middle SDI	Testicular cancer	0.03 (1 in 3193)	0	0.00 (1 in 30027)	0	0.00 (1 in 29109)	0	0.00 (1 in 24458)	0	0.03 (1 in 3482)	0	0.04 (1 in 2371)	0
Low-middle SDI	Kidney cancer	0.07 (1 in 1517)	0.06 (1 in 1544)	0.07 (1 in 1416)	0.05 (1 in 1908)	0.11 (1 in 917)	0.07 (1 in 1378)	0.13 (1 in 745)	0.08 (1 in 1231)	0.36 (1 in 280)	0.25 (1 in 405)	0.38 (1 in 264)	0.27 (1 in 369)

© 2019 Global Burden of Disease Cancer Collaboration. JAMA Oncol.

Location/SDI Quintile	Cancer	Birth to age 49		Age 50 to 59		Age 60 to 69		Age 70 to 79		Age 30 to 70		Birth to age 79	
		Male	Female	Male	Female	Male	Female	Male	Female	Male	Female	Male	Female
Low-middle SDI	Bladder cancer	0.06 (1 in 1737)	0.02 (1 in 4421)	0.11 (1 in 952)	0.04 (1 in 2850)	0.25 (1 in 405)	0.07 (1 in 1418)	0.39 (1 in 258)	0.13 (1 in 774)	0.79 (1 in 127)	0.25 (1 in 394)	0.79 (1 in 126)	0.26 (1 in 389)
Low-middle SDI	Brain and central nervous system cancer	0.11 (1 in 931)	0.09 (1 in 1163)	0.06 (1 in 1649)	0.05 (1 in 2208)	0.09 (1 in 1131)	0.06 (1 in 1601)	0.10 (1 in 1016)	0.07 (1 in 1353)	0.30 (1 in 328)	0.22 (1 in 445)	0.35 (1 in 282)	0.27 (1 in 374)
Low-middle SDI	Thyroid cancer	0.03 (1 in 3345)	0.14 (1 in 730)	0.02 (1 in 4274)	0.06 (1 in 1671)	0.03 (1 in 3423)	0.07 (1 in 1538)	0.04 (1 in 2516)	0.07 (1 in 1534)	0.12 (1 in 859)	0.28 (1 in 351)	0.12 (1 in 818)	0.33 (1 in 306)
Low-middle SDI	Mesothelioma	0.00 (1 in 21707)	0.01 (1 in 18635)	0.01 (1 in 10863)	0.00 (1 in 21068)	0.01 (1 in 8902)	0.01 (1 in 16866)	0.02 (1 in 5641)	0.01 (1 in 10515)	0.04 (1 in 2367)	0.02 (1 in 4050)	0.04 (1 in 2338)	0.03 (1 in 3914)
Low-middle SDI	Hodgkin lymphoma	0.05 (1 in 2194)	0.04 (1 in 2842)	0.02 (1 in 6296)	0.01 (1 in 14543)	0.02 (1 in 4112)	0.01 (1 in 9909)	0.03 (1 in 3415)	0.01 (1 in 6778)	0.09 (1 in 1101)	0.05 (1 in 2199)	0.12 (1 in 869)	0.07 (1 in 1495)
Low-middle SDI	Non-Hodgkin lymphoma	0.10 (1 in 1038)	0.07 (1 in 1355)	0.07 (1 in 1417)	0.05 (1 in 2039)	0.13 (1 in 784)	0.10 (1 in 1050)	0.17 (1 in 582)	0.13 (1 in 771)	0.42 (1 in 236)	0.31 (1 in 318)	0.47 (1 in 215)	0.35 (1 in 288)
Low-middle SDI	Multiple myeloma	0.01 (1 in 8763)	0.01 (1 in 8659)	0.02 (1 in 4463)	0.02 (1 in 4185)	0.05 (1 in 2195)	0.05 (1 in 1928)	0.07 (1 in 1530)	0.08 (1 in 1311)	0.14 (1 in 696)	0.16 (1 in 617)	0.14 (1 in 691)	0.16 (1 in 612)
Low-middle SDI	Acute lymphoid leukemia	0.04 (1 in 2756)	0.03 (1 in 3300)	0.01 (1 in 12089)	0.00 (1 in 21580)	0.01 (1 in 7646)	0.01 (1 in 15398)	0.01 (1 in 6975)	0.01 (1 in 12792)	0.05 (1 in 2196)	0.03 (1 in 3865)	0.07 (1 in 1390)	0.05 (1 in 2031)
Low-middle SDI	Chronic lymphoid leukemia	0.00 (1 in 37009)	0.00 (1 in 37120)	0.00 (1 in 22035)	0.00 (1 in 27137)	0.01 (1 in 9032)	0.01 (1 in 11464)	0.02 (1 in 4488)	0.01 (1 in 6915)	0.04 (1 in 2505)	0.03 (1 in 3468)	0.04 (1 in 2464)	0.03 (1 in 3383)
Low-middle SDI	Acute myeloid leukemia	0.04 (1 in 2235)	0.04 (1 in 2654)	0.02 (1 in 4621)	0.02 (1 in 6584)	0.04 (1 in 2782)	0.02 (1 in 4012)	0.05 (1 in 1933)	0.04 (1 in 2631)	0.13 (1 in 770)	0.09 (1 in 1054)	0.15 (1 in 649)	0.12 (1 in 864)
Low-middle SDI	Chronic myeloid leukemia	0.01 (1 in 10052)	0.01 (1 in 8563)	0.01 (1 in 14045)	0.01 (1 in 10754)	0.01 (1 in 8035)	0.01 (1 in 8280)	0.02 (1 in 4433)	0.02 (1 in 5783)	0.05 (1 in 2017)	0.05 (1 in 2118)	0.05 (1 in 1921)	0.05 (1 in 1987)

© 2019 Global Burden of Disease Cancer Collaboration. JAMA Oncol.

Location/SDI Quintile	Cancer	Birth to age 49		Age 50 to 59		Age 60 to 69		Age 70 to 79		Age 30 to 70		Birth to age 79	
		Male	Female	Male	Female	Male	Female	Male	Female	Male	Female	Male	Female
Low-middle SDI	Other leukemia	0.06 (1 in 1815)	0.05 (1 in 1927)	0.03 (1 in 3932)	0.02 (1 in 4543)	0.05 (1 in 1844)	0.04 (1 in 2729)	0.09 (1 in 1167)	0.06 (1 in 1716)	0.19 (1 in 539)	0.14 (1 in 734)	0.22 (1 in 454)	0.17 (1 in 593)
Low-middle SDI	Other malignant neoplasms	0.15 (1 in 686)	0.14 (1 in 697)	0.10 (1 in 1006)	0.09 (1 in 1159)	0.19 (1 in 525)	0.15 (1 in 680)	0.27 (1 in 365)	0.21 (1 in 480)	0.63 (1 in 159)	0.50 (1 in 200)	0.71 (1 in 141)	0.58 (1 in 171)
Low SDI	Neoplasms	1.66 (1 in 60)	3.04 (1 in 33)	2.35 (1 in 43)	3.02 (1 in 33)	4.55 (1 in 22)	3.96 (1 in 25)	7.05 (1 in 14)	4.98 (1 in 20)	14.46 (1 in 7)	13.73 (1 in 7)	14.81 (1 in 7)	14.19 (1 in 7)
Low SDI	Lip and oral cavity cancer	0.10 (1 in 1032)	0.08 (1 in 1211)	0.15 (1 in 649)	0.11 (1 in 921)	0.24 (1 in 422)	0.16 (1 in 610)	0.31 (1 in 325)	0.24 (1 in 415)	0.78 (1 in 127)	0.59 (1 in 171)	0.79 (1 in 126)	0.59 (1 in 168)
Low SDI	Nasopharynx cancer	0.04 (1 in 2531)	0.03 (1 in 3942)	0.04 (1 in 2429)	0.02 (1 in 4699)	0.05 (1 in 2079)	0.02 (1 in 4632)	0.05 (1 in 2030)	0.02 (1 in 5094)	0.17 (1 in 580)	0.08 (1 in 1224)	0.18 (1 in 562)	0.09 (1 in 1138)
Low SDI	Other pharynx cancer	0.07 (1 in 1529)	0.04 (1 in 2567)	0.14 (1 in 703)	0.07 (1 in 1346)	0.22 (1 in 460)	0.11 (1 in 951)	0.25 (1 in 408)	0.12 (1 in 869)	0.67 (1 in 150)	0.33 (1 in 304)	0.67 (1 in 150)	0.33 (1 in 300)
Low SDI	Esophageal cancer	0.06 (1 in 1817)	0.03 (1 in 3182)	0.14 (1 in 712)	0.07 (1 in 1473)	0.23 (1 in 434)	0.11 (1 in 911)	0.32 (1 in 314)	0.21 (1 in 480)	0.74 (1 in 135)	0.42 (1 in 241)	0.74 (1 in 135)	0.42 (1 in 240)
Low SDI	Stomach cancer	0.11 (1 in 936)	0.13 (1 in 768)	0.21 (1 in 482)	0.18 (1 in 546)	0.39 (1 in 254)	0.29 (1 in 342)	0.58 (1 in 172)	0.44 (1 in 227)	1.28 (1 in 78)	1.03 (1 in 97)	1.29 (1 in 78)	1.04 (1 in 96)
Low SDI	Colon and rectum cancer	0.09 (1 in 1090)	0.09 (1 in 1077)	0.17 (1 in 574)	0.17 (1 in 573)	0.37 (1 in 273)	0.27 (1 in 367)	0.61 (1 in 165)	0.48 (1 in 207)	1.23 (1 in 82)	1.01 (1 in 99)	1.24 (1 in 81)	1.02 (1 in 98)
Low SDI	Liver cancer	0.10 (1 in 980)	0.05 (1 in 2070)	0.18 (1 in 545)	0.09 (1 in 1159)	0.31 (1 in 324)	0.17 (1 in 582)	0.43 (1 in 234)	0.26 (1 in 384)	1.01 (1 in 99)	0.56 (1 in 179)	1.02 (1 in 98)	0.57 (1 in 177)
Low SDI	Gallbladder and biliary tract cancer	0.01 (1 in 9517)	0.02 (1 in 5580)	0.03 (1 in 3281)	0.06 (1 in 1768)	0.07 (1 in 1415)	0.12 (1 in 815)	0.12 (1 in 843)	0.18 (1 in 542)	0.23 (1 in 435)	0.38 (1 in 263)	0.23 (1 in 435)	0.38 (1 in 262)
Low SDI	Pancreatic cancer	0.02 (1 in 4866)	0.01 (1 in 6672)	0.06 (1 in 1817)	0.04 (1 in 2447)	0.12 (1 in 844)	0.09 (1 in 1073)	0.19 (1 in 531)	0.16 (1 in 623)	0.38 (1 in 262)	0.31 (1 in 324)	0.38 (1 in 262)	0.31 (1 in 323)

© 2019 Global Burden of Disease Cancer Collaboration. JAMA Oncol.

Location/SDI Quintile	Cancer	Birth to age 49		Age 50 to 59		Age 60 to 69		Age 70 to 79		Age 30 to 70		Birth to age 79	
		Male	Female	Male	Female	Male	Female	Male	Female	Male	Female	Male	Female
Low SDI	Larynx cancer	0.04 (1 in 2267)	0.02 (1 in 5479)	0.13 (1 in 768)	0.04 (1 in 2567)	0.22 (1 in 461)	0.06 (1 in 1677)	0.23 (1 in 443)	0.06 (1 in 1572)	0.62 (1 in 163)	0.18 (1 in 560)	0.62 (1 in 162)	0.18 (1 in 554)
Low SDI	Tracheal, bronchus, and lung cancer	0.09 (1 in 1167)	0.06 (1 in 1705)	0.28 (1 in 358)	0.11 (1 in 888)	0.69 (1 in 145)	0.21 (1 in 466)	1.18 (1 in 84)	0.32 (1 in 314)	2.22 (1 in 45)	0.70 (1 in 143)	2.22 (1 in 45)	0.70 (1 in 142)
Low SDI	Malignant skin melanoma	0.01 (1 in 9973)	0.01 (1 in 8007)	0.01 (1 in 10055)	0.01 (1 in 11109)	0.01 (1 in 7978)	0.01 (1 in 8364)	0.02 (1 in 5759)	0.02 (1 in 6062)	0.05 (1 in 2075)	0.05 (1 in 2115)	0.05 (1 in 2006)	0.05 (1 in 2003)
Low SDI	Non-melanoma skin cancer	0.32 (1 in 317)	0.27 (1 in 365)	0.24 (1 in 424)	0.17 (1 in 578)	0.37 (1 in 270)	0.24 (1 in 418)	0.49 (1 in 206)	0.28 (1 in 359)	1.32 (1 in 76)	0.89 (1 in 112)	1.40 (1 in 71)	0.96 (1 in 104)
Low SDI	Breast cancer	0.01 (1 in 12946)	0.61 (1 in 165)	0.01 (1 in 10019)	0.66 (1 in 151)	0.02 (1 in 5730)	0.69 (1 in 144)	0.02 (1 in 4071)	0.73 (1 in 138)	0.06 (1 in 1688)	2.63 (1 in 38)	0.06 (1 in 1675)	2.66 (1 in 38)
Low SDI	Cervical cancer	0	0.89 (1 in 112)	0	0.63 (1 in 158)	0	0.56 (1 in 180)	0	0.44 (1 in 225)	0	2.43 (1 in 41)	0	2.51 (1 in 40)
Low SDI	Uterine cancer	0	0.04 (1 in 2750)	0	0.09 (1 in 1128)	0	0.14 (1 in 704)	0	0.14 (1 in 706)	0	0.41 (1 in 246)	0	0.41 (1 in 245)
Low SDI	Ovarian cancer	0	0.11 (1 in 888)	0	0.14 (1 in 739)	0	0.16 (1 in 637)	0	0.16 (1 in 637)	0	0.54 (1 in 184)	0	0.56 (1 in 178)
Low SDI	Prostate cancer	0.02 (1 in 6088)	0	0.11 (1 in 871)	0	0.51 (1 in 194)	0	1.30 (1 in 77)	0	1.93 (1 in 52)	0	1.93 (1 in 52)	0
Low SDI	Testicular cancer	0.01 (1 in 8592)	0	0.00 (1 in 56134)	0	0.00 (1 in 48142)	0	0.00 (1 in 52010)	0	0.01 (1 in 7747)	0	0.02 (1 in 5741)	0
Low SDI	Kidney cancer	0.05 (1 in 1905)	0.05 (1 in 1832)	0.06 (1 in 1801)	0.04 (1 in 2383)	0.09 (1 in 1162)	0.06 (1 in 1809)	0.10 (1 in 993)	0.06 (1 in 1674)	0.27 (1 in 364)	0.19 (1 in 535)	0.29 (1 in 340)	0.21 (1 in 473)

 © 2019 Global Burden of Disease Cancer Collaboration. JAMA Oncol.

Location/SDI Quintile	Cancer	Birth to age 49		Age 50 to 59		Age 60 to 69		Age 70 to 79		Age 30 to 70		Birth to age 79	
		Male	Female	Male	Female	Male	Female	Male	Female	Male	Female	Male	Female
Low SDI	Bladder cancer	0.02 (1 in 4347)	0.01 (1 in 8108)	0.06 (1 in 1794)	0.03 (1 in 3735)	0.15 (1 in 659)	0.06 (1 in 1759)	0.28 (1 in 363)	0.11 (1 in 920)	0.50 (1 in 198)	0.20 (1 in 492)	0.51 (1 in 198)	0.20 (1 in 489)
Low SDI	Brain and central nervous system cancer	0.08 (1 in 1210)	0.08 (1 in 1212)	0.05 (1 in 1865)	0.05 (1 in 2044)	0.07 (1 in 1491)	0.06 (1 in 1737)	0.08 (1 in 1187)	0.07 (1 in 1425)	0.25 (1 in 399)	0.22 (1 in 456)	0.29 (1 in 348)	0.26 (1 in 386)
Low SDI	Thyroid cancer	0.02 (1 in 4128)	0.10 (1 in 1039)	0.02 (1 in 5625)	0.05 (1 in 2218)	0.03 (1 in 3726)	0.05 (1 in 1938)	0.03 (1 in 2902)	0.06 (1 in 1768)	0.10 (1 in 1022)	0.22 (1 in 452)	0.10 (1 in 968)	0.25 (1 in 401)
Low SDI	Mesothelioma	0.00 (1 in 30426)	0.01 (1 in 19035)	0.01 (1 in 14308)	0.01 (1 in 19343)	0.01 (1 in 10433)	0.01 (1 in 17108)	0.01 (1 in 6696)	0.01 (1 in 13017)	0.03 (1 in 2898)	0.02 (1 in 4292)	0.03 (1 in 2874)	0.02 (1 in 4176)
Low SDI	Hodgkin lymphoma	0.03 (1 in 2901)	0.02 (1 in 4969)	0.01 (1 in 6730)	0.01 (1 in 18237)	0.02 (1 in 4705)	0.01 (1 in 12205)	0.03 (1 in 3756)	0.01 (1 in 8367)	0.08 (1 in 1228)	0.04 (1 in 2836)	0.10 (1 in 1029)	0.05 (1 in 2186)
Low SDI	Non-Hodgkin lymphoma	0.09 (1 in 1072)	0.06 (1 in 1750)	0.07 (1 in 1446)	0.05 (1 in 2153)	0.12 (1 in 811)	0.09 (1 in 1156)	0.17 (1 in 594)	0.12 (1 in 831)	0.41 (1 in 242)	0.29 (1 in 348)	0.45 (1 in 221)	0.31 (1 in 322)
Low SDI	Multiple myeloma	0.01 (1 in 10074)	0.01 (1 in 11065)	0.02 (1 in 4812)	0.02 (1 in 4575)	0.04 (1 in 2260)	0.05 (1 in 2064)	0.07 (1 in 1519)	0.07 (1 in 1364)	0.14 (1 in 714)	0.15 (1 in 659)	0.14 (1 in 711)	0.15 (1 in 655)
Low SDI	Acute lymphoid leukemia	0.03 (1 in 3073)	0.03 (1 in 3750)	0.01 (1 in 14143)	0.00 (1 in 29652)	0.01 (1 in 9179)	0.00 (1 in 22921)	0.01 (1 in 9372)	0.00 (1 in 20156)	0.04 (1 in 2757)	0.02 (1 in 5543)	0.06 (1 in 1635)	0.04 (1 in 2541)
Low SDI	Chronic lymphoid leukemia	0.00 (1 in 73582)	0.00 (1 in 70405)	0.00 (1 in 24685)	0.00 (1 in 30611)	0.01 (1 in 8784)	0.01 (1 in 10667)	0.03 (1 in 3910)	0.02 (1 in 5992)	0.04 (1 in 2373)	0.03 (1 in 3285)	0.04 (1 in 2360)	0.03 (1 in 3252)
Low SDI	Acute myeloid leukemia	0.04 (1 in 2234)	0.03 (1 in 2979)	0.02 (1 in 4085)	0.02 (1 in 6135)	0.04 (1 in 2366)	0.03 (1 in 3925)	0.06 (1 in 1733)	0.04 (1 in 2595)	0.15 (1 in 688)	0.10 (1 in 1046)	0.17 (1 in 591)	0.11 (1 in 879)
Low SDI	Chronic myeloid leukemia	0.01 (1 in 7125)	0.02 (1 in 5895)	0.01 (1 in 9900)	0.01 (1 in 6796)	0.02 (1 in 5468)	0.02 (1 in 4927)	0.03 (1 in 3029)	0.03 (1 in 3443)	0.07 (1 in 1396)	0.08 (1 in 1299)	0.08 (1 in 1326)	0.08 (1 in 1235)

Location/SDI Quintile	Cancer	Birth to age 49		Age 50 to 59		Age 60 to 69		Age 70 to 79		Age 30 to 70		Birth to age 79	
		Male	Female	Male	Female	Male	Female	Male	Female	Male	Female	Male	Female
Low SDI	Other leukemia	0.03 (1 in 2979)	0.04 (1 in 2726)	0.02 (1 in 5557)	0.02 (1 in 5638)	0.04 (1 in 2754)	0.03 (1 in 3654)	0.06 (1 in 1751)	0.05 (1 in 2198)	0.12 (1 in 810)	0.10 (1 in 973)	0.14 (1 in 690)	0.13 (1 in 786)
Low SDI	Other malignant neoplasms	0.14 (1 in 699)	0.13 (1 in 759)	0.11 (1 in 936)	0.10 (1 in 1044)	0.18 (1 in 544)	0.15 (1 in 669)	0.26 (1 in 391)	0.20 (1 in 512)	0.61 (1 in 163)	0.50 (1 in 199)	0.69 (1 in 145)	0.57 (1 in 175)
Middle SDI	Neoplasms	2.81 (1 in 36)	3.83 (1 in 26)	3.91 (1 in 26)	3.66 (1 in 27)	9.33 (1 in 11)	5.89 (1 in 17)	14.40 (1 in 7)	8.10 (1 in 12)	27.03 (1 in 4)	19.32 (1 in 5)	27.51 (1 in 4)	19.88 (1 in 5)
Middle SDI	Lip and oral cavity cancer	0.09 (1 in 1064)	0.05 (1 in 1890)	0.13 (1 in 752)	0.06 (1 in 1687)	0.22 (1 in 449)	0.09 (1 in 1053)	0.30 (1 in 330)	0.15 (1 in 685)	0.74 (1 in 135)	0.35 (1 in 289)	0.75 (1 in 133)	0.35 (1 in 284)
Middle SDI	Nasopharynx cancer	0.09 (1 in 1157)	0.03 (1 in 3291)	0.06 (1 in 1546)	0.02 (1 in 5491)	0.08 (1 in 1198)	0.02 (1 in 4507)	0.08 (1 in 1270)	0.03 (1 in 3775)	0.30 (1 in 332)	0.09 (1 in 1086)	0.31 (1 in 320)	0.10 (1 in 1028)
Middle SDI	Other pharynx cancer	0.03 (1 in 3119)	0.01 (1 in 7429)	0.07 (1 in 1449)	0.02 (1 in 4689)	0.10 (1 in 976)	0.03 (1 in 3123)	0.10 (1 in 964)	0.04 (1 in 2812)	0.31 (1 in 327)	0.10 (1 in 993)	0.31 (1 in 326)	0.10 (1 in 977)
Middle SDI	Esophageal cancer	0.05 (1 in 1982)	0.03 (1 in 3753)	0.16 (1 in 608)	0.06 (1 in 1711)	0.50 (1 in 200)	0.18 (1 in 557)	0.80 (1 in 125)	0.32 (1 in 308)	1.50 (1 in 67)	0.59 (1 in 170)	1.50 (1 in 66)	0.59 (1 in 170)
Middle SDI	Stomach cancer	0.17 (1 in 588)	0.11 (1 in 909)	0.38 (1 in 261)	0.15 (1 in 654)	1.14 (1 in 88)	0.40 (1 in 251)	1.76 (1 in 57)	0.70 (1 in 143)	3.40 (1 in 29)	1.34 (1 in 74)	3.41 (1 in 29)	1.35 (1 in 74)
Middle SDI	Colon and rectum cancer	0.19 (1 in 525)	0.15 (1 in 675)	0.32 (1 in 311)	0.24 (1 in 415)	0.82 (1 in 122)	0.53 (1 in 187)	1.33 (1 in 75)	0.91 (1 in 110)	2.62 (1 in 38)	1.81 (1 in 55)	2.63 (1 in 38)	1.82 (1 in 55)
Middle SDI	Liver cancer	0.36 (1 in 275)	0.08 (1 in 1274)	0.54 (1 in 187)	0.14 (1 in 729)	1.01 (1 in 99)	0.34 (1 in 296)	1.20 (1 in 84)	0.52 (1 in 193)	3.05 (1 in 33)	1.06 (1 in 94)	3.08 (1 in 33)	1.07 (1 in 94)
Middle SDI	Gallbladder and biliary tract cancer	0.01 (1 in 8501)	0.01 (1 in 6895)	0.03 (1 in 3694)	0.04 (1 in 2811)	0.07 (1 in 1469)	0.09 (1 in 1160)	0.13 (1 in 775)	0.15 (1 in 662)	0.24 (1 in 425)	0.29 (1 in 349)	0.24 (1 in 424)	0.29 (1 in 348)
Middle SDI	Pancreatic cancer	0.03 (1 in 3178)	0.02 (1 in 4531)	0.07 (1 in 1371)	0.05 (1 in 1896)	0.18 (1 in 563)	0.13 (1 in 744)	0.29 (1 in 343)	0.24 (1 in 409)	0.57 (1 in 175)	0.45 (1 in 221)	0.57 (1 in 175)	0.45 (1 in 221)

 © 2019 Global Burden of Disease Cancer Collaboration. JAMA Oncol.

Location/SDI Quintile	Cancer	Birth to age 49		Age 50 to 59		Age 60 to 69		Age 70 to 79		Age 30 to 70		Birth to age 79	
		Male	Female	Male	Female	Male	Female	Male	Female	Male	Female	Male	Female
Middle SDI	Larynx cancer	0.03 (1 in 3123)	0.01 (1 in 12857)	0.09 (1 in 1054)	0.01 (1 in 6815)	0.20 (1 in 511)	0.03 (1 in 3346)	0.24 (1 in 415)	0.04 (1 in 2403)	0.56 (1 in 178)	0.09 (1 in 1074)	0.56 (1 in 178)	0.09 (1 in 1065)
Middle SDI	Tracheal, bronchus, and lung cancer	0.20 (1 in 508)	0.10 (1 in 961)	0.60 (1 in 166)	0.22 (1 in 450)	1.90 (1 in 52)	0.58 (1 in 171)	2.92 (1 in 34)	0.97 (1 in 103)	5.52 (1 in 18)	1.86 (1 in 54)	5.53 (1 in 18)	1.87 (1 in 54)
Middle SDI	Malignant skin melanoma	0.02 (1 in 4174)	0.02 (1 in 4497)	0.02 (1 in 5335)	0.01 (1 in 8361)	0.02 (1 in 4846)	0.02 (1 in 5620)	0.03 (1 in 3256)	0.03 (1 in 3397)	0.09 (1 in 1119)	0.08 (1 in 1325)	0.09 (1 in 1064)	0.08 (1 in 1228)
Middle SDI	Non-melanoma skin cancer	0.47 (1 in 213)	0.51 (1 in 196)	0.60 (1 in 165)	0.55 (1 in 182)	1.27 (1 in 79)	0.99 (1 in 101)	2.19 (1 in 46)	1.53 (1 in 65)	4.38 (1 in 23)	3.44 (1 in 29)	4.47 (1 in 22)	3.53 (1 in 28)
Middle SDI	Breast cancer	0.01 (1 in 12716)	1.00 (1 in 100)	0.01 (1 in 8289)	0.94 (1 in 106)	0.03 (1 in 3763)	1.08 (1 in 93)	0.03 (1 in 3682)	1.07 (1 in 93)	0.07 (1 in 1373)	3.99 (1 in 25)	0.07 (1 in 1358)	4.04 (1 in 25)
Middle SDI	Cervical cancer	0	0.59 (1 in 169)	0	0.38 (1 in 260)	0	0.38 (1 in 264)	0	0.34 (1 in 297)	0	1.63 (1 in 61)	0	1.68 (1 in 59)
Middle SDI	Uterine cancer	0	0.13 (1 in 762)	0	0.22 (1 in 464)	0	0.24 (1 in 423)	0	0.20 (1 in 497)	0	0.77 (1 in 129)	0	0.78 (1 in 128)
Middle SDI	Ovarian cancer	0	0.17 (1 in 589)	0	0.15 (1 in 688)	0	0.18 (1 in 566)	0	0.18 (1 in 553)	0	0.64 (1 in 157)	0	0.67 (1 in 149)
Middle SDI	Prostate cancer	0.04 (1 in 2321)	0	0.22 (1 in 456)	0	0.95 (1 in 106)	0	2.18 (1 in 46)	0	3.36 (1 in 30)	0	3.36 (1 in 30)	0
Middle SDI	Testicular cancer	0.07 (1 in 1368)	0	0.01 (1 in 19072)	0	0.01 (1 in 13969)	0	0.01 (1 in 9199)	0	0.06 (1 in 1774)	0	0.10 (1 in 1038)	0
Middle SDI	Kidney cancer	0.08 (1 in 1314)	0.07 (1 in 1454)	0.09 (1 in 1114)	0.05 (1 in 1985)	0.14 (1 in 714)	0.08 (1 in 1297)	0.19 (1 in 540)	0.09 (1 in 1059)	0.47 (1 in 213)	0.26 (1 in 379)	0.49 (1 in 204)	0.29 (1 in 344)

© 2019 Global Burden of Disease Cancer Collaboration. JAMA Oncol.

Location/SDI Quintile	Cancer	Birth to age 49		Age 50 to 59		Age 60 to 69		Age 70 to 79		Age 30 to 70		Birth to age 79	
		Male	Female	Male	Female	Male	Female	Male	Female	Male	Female	Male	Female
Middle SDI	Bladder cancer	0.04 (1 in 2485)	0.02 (1 in 6328)	0.08 (1 in 1211)	0.03 (1 in 3829)	0.23 (1 in 438)	0.05 (1 in 1819)	0.43 (1 in 234)	0.11 (1 in 902)	0.77 (1 in 129)	0.21 (1 in 486)	0.78 (1 in 129)	0.21 (1 in 482)
Middle SDI	Brain and central nervous system cancer	0.18 (1 in 571)	0.14 (1 in 700)	0.08 (1 in 1271)	0.06 (1 in 1656)	0.13 (1 in 744)	0.10 (1 in 1036)	0.16 (1 in 642)	0.11 (1 in 900)	0.46 (1 in 218)	0.34 (1 in 294)	0.54 (1 in 184)	0.41 (1 in 244)
Middle SDI	Thyroid cancer	0.05 (1 in 2185)	0.14 (1 in 729)	0.04 (1 in 2379)	0.09 (1 in 1148)	0.04 (1 in 2605)	0.09 (1 in 1092)	0.06 (1 in 1752)	0.08 (1 in 1216)	0.18 (1 in 570)	0.38 (1 in 265)	0.18 (1 in 546)	0.40 (1 in 252)
Middle SDI	Mesothelioma	0.01 (1 in 18055)	0.00 (1 in 29947)	0.01 (1 in 10114)	0.00 (1 in 33846)	0.01 (1 in 10674)	0.00 (1 in 21954)	0.02 (1 in 6489)	0.01 (1 in 12984)	0.04 (1 in 2534)	0.02 (1 in 5579)	0.04 (1 in 2488)	0.02 (1 in 5391)
Middle SDI	Hodgkin lymphoma	0.03 (1 in 2893)	0.03 (1 in 3689)	0.01 (1 in 7571)	0.01 (1 in 19172)	0.03 (1 in 3621)	0.01 (1 in 13865)	0.03 (1 in 3651)	0.01 (1 in 10343)	0.08 (1 in 1189)	0.03 (1 in 2997)	0.10 (1 in 973)	0.05 (1 in 2033)
Middle SDI	Non-Hodgkin lymphoma	0.11 (1 in 929)	0.06 (1 in 1542)	0.09 (1 in 1088)	0.05 (1 in 1902)	0.16 (1 in 616)	0.10 (1 in 1016)	0.21 (1 in 483)	0.13 (1 in 751)	0.53 (1 in 188)	0.33 (1 in 306)	0.57 (1 in 176)	0.35 (1 in 287)
Middle SDI	Multiple myeloma	0.01 (1 in 7099)	0.01 (1 in 9793)	0.03 (1 in 3771)	0.02 (1 in 4673)	0.05 (1 in 1861)	0.04 (1 in 2409)	0.08 (1 in 1333)	0.06 (1 in 1637)	0.17 (1 in 597)	0.13 (1 in 754)	0.17 (1 in 591)	0.13 (1 in 745)
Middle SDI	Acute lymphoid leukemia	0.05 (1 in 1828)	0.04 (1 in 2507)	0.01 (1 in 8353)	0.01 (1 in 12268)	0.02 (1 in 4941)	0.01 (1 in 8268)	0.02 (1 in 4127)	0.01 (1 in 6781)	0.07 (1 in 1392)	0.05 (1 in 2182)	0.11 (1 in 900)	0.07 (1 in 1336)
Middle SDI	Chronic lymphoid leukemia	0.02 (1 in 6403)	0.01 (1 in 7974)	0.01 (1 in 7886)	0.01 (1 in 9866)	0.02 (1 in 4134)	0.02 (1 in 6352)	0.03 (1 in 3047)	0.02 (1 in 5747)	0.08 (1 in 1232)	0.05 (1 in 1892)	0.09 (1 in 1173)	0.06 (1 in 1792)
Middle SDI	Acute myeloid leukemia	0.04 (1 in 2303)	0.04 (1 in 2627)	0.02 (1 in 5488)	0.01 (1 in 8012)	0.03 (1 in 3535)	0.02 (1 in 5313)	0.04 (1 in 2253)	0.03 (1 in 3673)	0.11 (1 in 900)	0.08 (1 in 1313)	0.13 (1 in 745)	0.10 (1 in 1036)
Middle SDI	Chronic myeloid leukemia	0.01 (1 in 9380)	0.01 (1 in 14119)	0.01 (1 in 14873)	0.00 (1 in 21113)	0.01 (1 in 10395)	0.01 (1 in 17306)	0.02 (1 in 5859)	0.01 (1 in 11448)	0.04 (1 in 2414)	0.02 (1 in 4083)	0.04 (1 in 2269)	0.03 (1 in 3798)

© 2019 Global Burden of Disease Cancer Collaboration. JAMA Oncol.

Location/SDI Quintile	Cancer	Birth to age 49		Age 50 to 59		Age 60 to 69		Age 70 to 79		Age 30 to 70		Birth to age 79	
		Male	Female	Male	Female	Male	Female	Male	Female	Male	Female	Male	Female
Middle SDI	Other leukemia	0.13 (1 in 780)	0.11 (1 in 939)	0.03 (1 in 2952)	0.03 (1 in 3779)	0.08 (1 in 1238)	0.05 (1 in 1961)	0.13 (1 in 788)	0.07 (1 in 1385)	0.28 (1 in 363)	0.18 (1 in 563)	0.37 (1 in 271)	0.26 (1 in 391)
Middle SDI	Other malignant neoplasms	0.22 (1 in 460)	0.19 (1 in 535)	0.13 (1 in 747)	0.09 (1 in 1088)	0.30 (1 in 339)	0.17 (1 in 591)	0.43 (1 in 233)	0.25 (1 in 399)	0.98 (1 in 102)	0.60 (1 in 165)	1.07 (1 in 93)	0.70 (1 in 143)

© 2019 Global Burden of Disease Cancer Collaboration. JAMA Oncol.

eTable 17: List of 22 level 2 causes in the GBD cause hierarchy

**Level 2 Causes**
Cardivascular Diseases
Chronic Respiratory Diseases
Diabetes and Kidney Diseases
Digestive Diseases
Enteric Infections
HIV/AIDS and Sexually Transmitted Infections
Maternal and Neonatal Disorders
Mental Disorders
Musculoskeletal Disorders
Neglected Tropical Diseases and Malaria
Neoplasms
Neurological Disorders
Nutritional Deficiencies
Other Infectious Diseases
Other Non-communicable Diseases
Respiratory Infections and Tuberculosis
Self-harm and Interpersonal Violence
Sense Organ Diseases
Skin and Subcutaneous Diseases
Substance Use Disorders
Transport Injuries
Unintentional Injuries

 © 2019 Global Burden of Disease Cancer Collaboration. JAMA Oncol.

eTable 18: Global number of incidence, prevalence, YLDs, deaths, YLLs, DALYs for both sexes, 1990 and 2017 for all level 2 GBD causes

Measure	Cause Name	Year	Number	Lower 95% UI	Upper 95% UI
DALYs	Cardiovascular diseases	2017	365,869,825	355,162,644	376,747,292
DALYs	Neoplasms	2017	233,513,217	228,805,646	237,972,854
DALYs	Maternal and neonatal disorders	2017	197,578,932	186,854,664	209,023,949
DALYs	Respiratory infections and tuberculosis	2017	159,903,714	151,790,181	168,292,899
DALYs	Musculoskeletal disorders	2017	138,723,945	101,940,849	182,552,790
DALYs	Mental disorders	2017	122,763,812	91,637,784	157,901,472
DALYs	Other non-communicable diseases	2017	121,886,702	103,879,215	143,822,110
DALYs	Chronic respiratory diseases	2017	112,316,763	104,649,691	119,692,783
DALYs	Neurological disorders	2017	111,166,327	88,499,698	138,526,015
DALYs	Unintentional injuries	2017	105,940,182	94,243,724	119,496,121
DALYs	Diabetes and kidney diseases	2017	104,001,226	90,075,147	120,518,945
DALYs	Enteric infections	2017	95,209,183	83,914,445	112,177,985
DALYs	Digestive diseases	2017	85,288,128	77,994,367	94,532,822
DALYs	Transport injuries	2017	75,332,128	71,039,428	79,823,272
DALYs	Self-harm and interpersonal violence	2017	71,133,307	68,080,957	73,745,480
DALYs	Sense organ diseases	2017	66,576,077	44,700,752	95,675,085
DALYs	HIV/AIDS and sexually transmitted infections	2017	65,919,941	58,660,134	74,697,873
DALYs	Neglected tropical diseases and malaria	2017	62,279,047	48,556,871	79,850,662
DALYs	Nutritional deficiencies	2017	58,034,285	44,284,298	76,877,407
DALYs	Other infectious diseases	2017	57,065,192	48,750,501	67,270,886

 © 2019 Global Burden of Disease Cancer Collaboration. JAMA Oncol.

Measure	Cause Name	Year	Number	Lower 95% UI	Upper 95% UI
DALYs	Substance use disorders	2017	44,650,333	35,859,543	54,047,325
DALYs	Skin and subcutaneous diseases	2017	44,139,790	29,932,029	64,244,621
DALYs	Respiratory infections and tuberculosis	1990	309,249,358	287,109,014	328,732,086
DALYs	Maternal and neonatal disorders	1990	297,652,248	280,126,263	316,538,704
DALYs	Cardiovascular diseases	1990	266,817,904	260,266,782	273,590,216
DALYs	Enteric infections	1990	199,281,798	170,533,635	226,024,752
DALYs	Other infectious diseases	1990	159,996,995	123,375,053	217,837,555
DALYs	Neoplasms	1990	159,583,902	155,128,690	165,455,157
DALYs	Other non-communicable diseases	1990	128,171,244	113,077,150	148,016,766
DALYs	Unintentional injuries	1990	126,209,239	115,160,961	136,811,492
DALYs	Chronic respiratory diseases	1990	99,103,908	91,717,116	104,764,799
DALYs	Neglected tropical diseases and malaria	1990	92,881,784	61,865,316	147,002,267
DALYs	Nutritional deficiencies	1990	92,440,346	74,687,081	115,060,526
DALYs	Musculoskeletal disorders	1990	83,700,570	61,719,746	110,423,940
DALYs	Mental disorders	1990	82,174,005	61,252,297	106,197,991
DALYs	Transport injuries	1990	75,541,558	70,729,138	79,946,605
DALYs	Neurological disorders	1990	68,944,493	54,331,793	86,705,451
DALYs	Self-harm and interpersonal violence	1990	67,805,441	63,282,481	70,692,660
DALYs	Digestive diseases	1990	67,393,607	62,188,680	73,641,123
DALYs	Diabetes and kidney diseases	1990	54,837,205	48,420,026	62,647,669
DALYs	Sense organ diseases	1990	37,792,611	25,518,303	54,299,006

© 2019 Global Burden of Disease Cancer Collaboration. JAMA Oncol.

Measure	Cause Name	Year	Number	Lower 95% UI	Upper 95% UI
DALYs	HIV/AIDS and sexually transmitted infections	1990	33,662,794	26,056,389	43,963,291
DALYs	Skin and subcutaneous diseases	1990	31,244,325	21,035,604	45,486,196
DALYs	Substance use disorders	1990	27,524,799	21,814,247	33,432,553
Deaths	Cardiovascular diseases	2017	17,790,949	17,527,068	18,042,674
Deaths	Neoplasms	2017	9,556,245	9,395,666	9,692,259
Deaths	Chronic respiratory diseases	2017	3,914,196	3,790,579	4,044,819
Deaths	Respiratory infections and tuberculosis	2017	3,752,338	3,629,350	3,889,284
Deaths	Neurological disorders	2017	3,094,164	3,039,575	3,142,640
Deaths	Diabetes and kidney diseases	2017	2,611,200	2,557,844	2,667,162
Deaths	Digestive diseases	2017	2,377,685	2,295,066	2,517,953
Deaths	Maternal and neonatal disorders	2017	1,977,409	1,890,055	2,060,599
Deaths	Unintentional injuries	2017	1,804,870	1,695,711	1,872,031
Deaths	Enteric infections	2017	1,765,992	1,397,988	2,385,974
Deaths	Self-harm and interpersonal violence	2017	1,344,849	1,283,066	1,380,444
Deaths	Transport injuries	2017	1,335,004	1,289,145	1,369,543
Deaths	Other non-communicable diseases	2017	1,153,269	1,101,794	1,208,344
Deaths	HIV/AIDS and sexually transmitted infections	2017	1,073,585	983,342	1,182,438
Deaths	Other infectious diseases	2017	830,494	732,153	947,812
Deaths	Neglected tropical diseases and malaria	2017	720,060	530,675	938,820
Deaths	Substance use disorders	2017	351,547	334,103	362,720
Deaths	Nutritional deficiencies	2017	269,997	249,250	295,457

© 2019 Global Burden of Disease Cancer Collaboration. JAMA Oncol.

Measure	Cause Name	Year	Number	Lower 95% UI	Upper 95% UI
Deaths	Musculoskeletal disorders	2017	121,269	105,635	126,226
Deaths	Skin and subcutaneous diseases	2017	100,284	65,291	131,675
Deaths	Mental disorders	2017	326	296	357
Deaths	Cardiovascular diseases	1990	11,941,533	11,784,719	12,179,150
Deaths	Neoplasms	1990	5,753,106	5,660,626	5,902,948
Deaths	Respiratory infections and tuberculosis	1990	5,054,751	4,778,503	5,282,255
Deaths	Chronic respiratory diseases	1990	3,317,205	3,011,594	3,425,405
Deaths	Maternal and neonatal disorders	1990	3,311,471	3,128,143	3,516,311
Deaths	Enteric infections	1990	2,856,337	2,366,559	3,394,724
Deaths	Other infectious diseases	1990	2,018,856	1,588,420	2,690,138
Deaths	Unintentional injuries	1990	1,813,444	1,693,822	1,917,316
Deaths	Digestive diseases	1990	1,714,979	1,609,155	1,778,129
Deaths	Neurological disorders	1990	1,353,808	1,324,894	1,393,024
Deaths	Diabetes and kidney diseases	1990	1,235,855	1,202,582	1,277,753
Deaths	Transport injuries	1990	1,228,048	1,168,472	1,290,344
Deaths	Self-harm and interpersonal violence	1990	1,219,001	1,127,143	1,265,197
Deaths	Other non-communicable diseases	1990	1,216,750	1,125,135	1,384,186
Deaths	Neglected tropical diseases and malaria	1990	1,093,434	668,467	1,866,938
Deaths	Nutritional deficiencies	1990	568,075	486,089	638,305
Deaths	HIV/AIDS and sexually transmitted infections	1990	489,277	393,772	609,195
Deaths	Substance use disorders	1990	183,420	177,922	191,186

© 2019 Global Burden of Disease Cancer Collaboration. JAMA Oncol.

Measure	Cause Name	Year	Number	Lower 95% UI	Upper 95% UI
Deaths	Musculoskeletal disorders	1990	64,688	59,945	70,046
Deaths	Skin and subcutaneous diseases	1990	44,097	28,280	53,511
Deaths	Mental disorders	1990	180	165	218
Incidence	Respiratory infections and tuberculosis	2017	17,942,622,200	16,102,037,352	20,038,445,370
Incidence	Enteric infections	2017	6,307,792,414	5,822,111,274	6,830,241,351
Incidence	Other non-communicable diseases	2017	4,209,629,187	3,838,650,712	4,611,874,192
Incidence	Skin and subcutaneous diseases	2017	4,185,971,292	3,971,760,520	4,391,218,151
Incidence	Nutritional deficiencies	2017	1,186,745,815	1,089,728,901	1,283,530,022
Incidence	Neurological disorders	2017	1,006,294,496	907,590,708	1,098,468,897
Incidence	HIV/AIDS and sexually transmitted infections	2017	769,111,205	694,471,147	850,896,008
Incidence	Other infectious diseases	2017	478,720,559	450,498,254	511,601,597
Incidence	Digestive diseases	2017	465,978,615	429,600,439	500,015,074
Incidence	Unintentional injuries	2017	415,410,278	390,092,604	441,943,041
Incidence	Neglected tropical diseases and malaria	2017	357,652,091	301,519,177	431,965,055
Incidence	Mental disorders	2017	336,996,264	315,596,544	362,049,506
Incidence	Musculoskeletal disorders	2017	334,744,943	309,934,048	363,175,826
Incidence	Maternal and neonatal disorders	2017	101,960,798	94,724,853	109,282,804
Incidence	Cardiovascular diseases	2017	72,721,167	70,388,093	75,264,106
Incidence	Transport injuries	2017	63,920,593	56,848,496	71,592,218
Incidence	Chronic respiratory diseases	2017	62,161,350	55,134,836	69,320,715
Incidence	Substance use disorders	2017	60,099,555	53,685,605	67,048,695

 © 2019 Global Burden of Disease Cancer Collaboration. JAMA Oncol.

Measure	Cause Name	Year	Number	Lower 95% UI	Upper 95% UI
Incidence	Diabetes and kidney diseases	2017	43,444,563	40,700,271	46,375,597
Incidence	Self-harm and interpersonal violence	2017	41,379,417	37,344,547	45,834,432
Incidence	Neoplasms	2017	24,361,623	21,911,282	27,310,277
Incidence	Sense organ diseases	2017	-	-	-
Incidence	Respiratory infections and tuberculosis	1990	13,502,660,152	12,007,693,041	15,052,281,627
Incidence	Enteric infections	1990	4,162,053,408	3,839,162,594	4,529,163,204
Incidence	Other non-communicable diseases	1990	3,159,707,381	2,856,796,112	3,495,814,318
Incidence	Skin and subcutaneous diseases	1990	2,850,889,202	2,704,698,348	3,008,990,166
Incidence	Nutritional deficiencies	1990	895,796,227	822,831,524	969,303,058
Incidence	Neurological disorders	1990	688,458,508	617,026,906	756,703,630
Incidence	HIV/AIDS and sexually transmitted infections	1990	541,311,192	487,195,165	601,284,841
Incidence	Other infectious diseases	1990	453,739,592	401,443,447	541,991,902
Incidence	Unintentional injuries	1990	280,906,990	265,226,755	297,904,952
Incidence	Digestive diseases	1990	280,585,014	258,118,127	303,159,560
Incidence	Neglected tropical diseases and malaria	1990	276,404,045	236,287,640	321,720,611
Incidence	Mental disorders	1990	230,730,647	215,520,378	248,672,632
Incidence	Musculoskeletal disorders	1990	211,801,995	194,524,169	231,091,319
Incidence	Maternal and neonatal disorders	1990	95,589,927	88,248,268	103,319,698
Incidence	Chronic respiratory diseases	1990	49,341,401	43,429,933	55,491,013
Incidence	Substance use disorders	1990	41,669,778	37,393,249	46,338,692
Incidence	Transport injuries	1990	40,619,361	36,859,527	44,752,963

© 2019 Global Burden of Disease Cancer Collaboration. JAMA Oncol.

Measure	Cause Name	Year	Number	Lower 95% UI	Upper 95% UI
Incidence	Cardiovascular diseases	1990	39,828,826	38,585,410	41,150,293
Incidence	Self-harm and interpersonal violence	1990	32,537,950	29,978,817	35,293,359
Incidence	Diabetes and kidney diseases	1990	23,099,044	21,832,557	24,399,673
Incidence	Neoplasms	1990	12,144,252	10,595,310	14,146,112
Incidence	Sense organ diseases	1990	-	-	-
Prevalence	Other non-communicable diseases	2017	4,916,184,783	4,793,498,008	5,046,526,699
Prevalence	Neurological disorders	2017	3,121,435,268	2,951,124,541	3,316,267,996
Prevalence	Respiratory infections and tuberculosis	2017	2,187,289,983	1,979,143,104	2,449,760,681
Prevalence	Digestive diseases	2017	2,049,831,181	1,983,314,286	2,122,941,636
Prevalence	Sense organ diseases	2017	2,035,736,962	1,994,115,837	2,079,908,632
Prevalence	Skin and subcutaneous diseases	2017	1,974,238,419	1,916,671,830	2,034,645,664
Prevalence	Nutritional deficiencies	2017	1,862,030,823	1,806,258,916	1,921,493,541
Prevalence	Musculoskeletal disorders	2017	1,312,131,317	1,248,058,740	1,383,422,599
Prevalence	Neglected tropical diseases and malaria	2017	1,278,896,491	1,223,506,131	1,343,059,250
Prevalence	HIV/AIDS and sexually transmitted infections	2017	1,238,129,159	1,129,539,606	1,359,466,048
Prevalence	Diabetes and kidney diseases	2017	1,011,116,584	962,767,855	1,065,061,459
Prevalence	Mental disorders	2017	970,812,351	923,455,434	1,020,930,608
Prevalence	Unintentional injuries	2017	935,298,172	876,022,470	1,008,077,126
Prevalence	Chronic respiratory diseases	2017	544,899,165	506,937,518	584,858,359
Prevalence	Cardiovascular diseases	2017	485,620,950	468,031,728	504,964,407
Prevalence	Self-harm and interpersonal violence	2017	351,859,587	316,749,655	390,321,671

© 2019 Global Burden of Disease Cancer Collaboration. JAMA Oncol.

Measure	Cause Name	Year	Number	Lower 95% UI	Upper 95% UI
Prevalence	Transport injuries	2017	226,305,638	209,529,544	244,291,124
Prevalence	Substance use disorders	2017	175,588,843	161,747,600	189,304,254
Prevalence	Maternal and neonatal disorders	2017	158,835,793	140,427,731	179,076,822
Prevalence	Other infectious diseases	2017	101,451,535	97,425,139	105,559,560
Prevalence	Neoplasms	2017	100,482,889	98,189,805	102,850,506
Prevalence	Enteric infections	2017	93,304,411	86,780,482	99,732,495
Prevalence	Other non-communicable diseases	1990	3,474,628,536	3,374,989,918	3,580,497,968
Prevalence	Neurological disorders	1990	2,068,430,109	1,947,827,470	2,207,500,340
Prevalence	Respiratory infections and tuberculosis	1990	1,697,934,680	1,523,118,758	1,903,590,782
Prevalence	Nutritional deficiencies	1990	1,641,183,577	1,596,654,178	1,687,460,167
Prevalence	Neglected tropical diseases and malaria	1990	1,630,980,213	1,557,375,484	1,707,546,817
Prevalence	Skin and subcutaneous diseases	1990	1,363,280,022	1,318,958,274	1,410,152,783
Prevalence	Digestive diseases	1990	1,224,973,046	1,179,097,010	1,273,152,546
Prevalence	Sense organ diseases	1990	1,171,843,888	1,143,859,597	1,199,940,347
Prevalence	Musculoskeletal disorders	1990	770,756,735	728,826,864	816,462,318
Prevalence	HIV/AIDS and sexually transmitted infections	1990	740,179,879	676,989,227	812,521,414
Prevalence	Mental disorders	1990	670,158,920	632,356,809	710,324,373
Prevalence	Unintentional injuries	1990	580,747,757	543,305,900	624,026,558
Prevalence	Diabetes and kidney diseases	1990	519,023,373	494,200,000	546,128,664
Prevalence	Chronic respiratory diseases	1990	389,713,748	362,943,030	416,351,401
Prevalence	Cardiovascular diseases	1990	265,095,201	254,942,409	275,685,322

 © 2019 Global Burden of Disease Cancer Collaboration. JAMA Oncol.

Measure	Cause Name	Year	Number	Lower 95% UI	Upper 95% UI
Prevalence	Self-harm and interpersonal violence	1990	230,846,459	206,949,815	257,232,508
Prevalence	Transport injuries	1990	129,425,989	119,576,669	139,934,827
Prevalence	Substance use disorders	1990	117,560,678	107,615,832	126,965,440
Prevalence	Maternal and neonatal disorders	1990	91,996,021	82,385,132	102,937,270
Prevalence	Other infectious diseases	1990	89,491,596	85,874,032	93,739,482
Prevalence	Enteric infections	1990	64,260,896	60,005,607	68,832,846
Prevalence	Neoplasms	1990	45,616,387	44,766,119	46,545,262
YLDs	Musculoskeletal disorders	2017	135,881,289	99,022,584	179,645,044
YLDs	Mental disorders	2017	122,746,275	91,620,791	157,883,611
YLDs	Neurological disorders	2017	73,161,793	50,721,867	100,409,886
YLDs	Sense organ diseases	2017	66,576,077	44,700,752	95,675,085
YLDs	Other non-communicable diseases	2017	53,645,926	36,899,697	74,479,326
YLDs	Diabetes and kidney diseases	2017	45,884,350	32,018,931	62,235,263
YLDs	Chronic respiratory diseases	2017	44,311,836	36,751,640	51,407,142
YLDs	Nutritional deficiencies	2017	42,376,235	28,774,037	61,009,925
YLDs	Skin and subcutaneous diseases	2017	41,621,861	27,371,732	61,859,479
YLDs	Unintentional injuries	2017	36,509,677	26,384,700	49,052,539
YLDs	Cardiovascular diseases	2017	35,697,253	26,428,225	45,510,278
YLDs	Substance use disorders	2017	31,052,753	22,215,188	40,495,983
YLDs	Maternal and neonatal disorders	2017	29,894,299	22,429,873	38,381,611
YLDs	Digestive diseases	2017	19,939,736	13,858,238	27,973,060

© 2019 Global Burden of Disease Cancer Collaboration. JAMA Oncol.

Measure	Cause Name	Year	Number	Lower 95% UI	Upper 95% UI
YLDs	Neglected tropical diseases and malaria	2017	13,622,881	9,498,303	18,673,320
YLDs	Transport injuries	2017	13,394,368	9,586,914	17,860,967
YLDs	Respiratory infections and tuberculosis	2017	11,670,255	7,845,864	16,749,663
YLDs	Enteric infections	2017	10,583,692	7,283,322	14,516,077
YLDs	Neoplasms	2017	7,775,158	5,747,908	10,028,878
YLDs	Self-harm and interpersonal violence	2017	7,270,424	5,637,234	9,008,320
YLDs	HIV/AIDS and sexually transmitted infections	2017	5,369,731	3,783,642	7,272,173
YLDs	Other infectious diseases	2017	4,056,632	2,835,524	5,535,761
YLDs	Mental disorders	1990	82,163,903	61,242,855	106,188,391
YLDs	Musculoskeletal disorders	1990	81,879,380	59,968,022	108,618,752
YLDs	Nutritional deficiencies	1990	50,121,716	33,951,502	71,174,016
YLDs	Neurological disorders	1990	45,973,047	31,380,768	63,585,106
YLDs	Other non-communicable diseases	1990	37,794,080	26,384,847	52,599,757
YLDs	Sense organ diseases	1990	37,792,611	25,518,303	54,299,006
YLDs	Skin and subcutaneous diseases	1990	29,698,449	19,377,040	43,898,309
YLDs	Chronic respiratory diseases	1990	29,593,127	24,365,930	34,646,110
YLDs	Unintentional injuries	1990	24,103,866	17,493,924	32,208,061
YLDs	Diabetes and kidney diseases	1990	21,099,507	14,928,495	28,830,771
YLDs	Substance use disorders	1990	19,823,842	14,141,024	25,685,811
YLDs	Cardiovascular diseases	1990	19,023,712	14,033,706	24,347,894
YLDs	Maternal and neonatal disorders	1990	16,316,795	12,406,447	20,557,421

© 2019 Global Burden of Disease Cancer Collaboration. JAMA Oncol.

Measure	Cause Name	Year	Number	Lower 95% UI	Upper 95% UI
YLDs	Neglected tropical diseases and malaria	1990	14,829,129	10,337,134	20,437,316
YLDs	Digestive diseases	1990	12,623,835	8,751,361	17,722,376
YLDs	Respiratory infections and tuberculosis	1990	9,144,262	6,171,066	13,115,875
YLDs	Transport injuries	1990	8,290,585	6,015,733	10,939,425
YLDs	Enteric infections	1990	7,357,950	5,073,549	10,084,387
YLDs	Self-harm and interpersonal violence	1990	5,057,581	3,866,844	6,282,639
YLDs	Other infectious diseases	1990	3,882,144	2,707,877	5,327,236
YLDs	Neoplasms	1990	3,470,836	2,567,213	4,465,990
YLDs	HIV/AIDS and sexually transmitted infections	1990	1,879,849	1,292,767	2,693,337
YLLs	Cardiovascular diseases	2017	330,172,573	324,899,269	335,159,851
YLLs	Neoplasms	2017	225,738,059	221,608,831	229,322,358
YLLs	Maternal and neonatal disorders	2017	167,684,633	160,060,651	174,918,240
YLLs	Respiratory infections and tuberculosis	2017	148,233,459	141,335,115	155,291,373
YLLs	Enteric infections	2017	84,625,491	73,770,610	100,720,201
YLLs	Unintentional injuries	2017	69,430,505	64,685,078	72,366,778
YLLs	Other non-communicable diseases	2017	68,240,776	64,835,446	72,452,095
YLLs	Chronic respiratory diseases	2017	68,004,927	65,869,370	70,592,230
YLLs	Digestive diseases	2017	65,348,392	62,343,905	69,371,349
YLLs	Self-harm and interpersonal violence	2017	63,862,883	61,029,858	65,755,681
YLLs	Transport injuries	2017	61,937,760	60,031,222	63,736,519
YLLs	HIV/AIDS and sexually transmitted infections	2017	60,550,210	53,533,737	69,156,338

 © 2019 Global Burden of Disease Cancer Collaboration. JAMA Oncol.

Measure	Cause Name	Year	Number	Lower 95% UI	Upper 95% UI
YLLs	Diabetes and kidney diseases	2017	58,116,876	56,801,498	59,525,664
YLLs	Other infectious diseases	2017	53,008,560	44,785,978	63,000,380
YLLs	Neglected tropical diseases and malaria	2017	48,656,166	35,574,594	64,934,228
YLLs	Neurological disorders	2017	38,004,534	37,134,801	39,174,578
YLLs	Nutritional deficiencies	2017	15,658,050	14,051,478	17,506,635
YLLs	Substance use disorders	2017	13,597,580	12,979,527	14,033,332
YLLs	Musculoskeletal disorders	2017	2,842,656	2,440,732	2,953,062
YLLs	Skin and subcutaneous diseases	2017	2,517,929	1,703,324	3,283,772
YLLs	Mental disorders	2017	17,537	15,870	19,228
YLLs	Respiratory infections and tuberculosis	1990	300,105,095	278,005,075	318,858,702
YLLs	Maternal and neonatal disorders	1990	281,335,453	265,219,348	299,507,165
YLLs	Cardiovascular diseases	1990	247,794,192	243,663,436	252,576,620
YLLs	Enteric infections	1990	191,923,848	163,901,789	218,985,240
YLLs	Other infectious diseases	1990	156,114,851	119,890,267	213,154,068
YLLs	Neoplasms	1990	156,113,066	151,954,276	161,928,310
YLLs	Unintentional injuries	1990	102,105,373	93,583,569	109,892,883
YLLs	Other non-communicable diseases	1990	90,377,163	82,271,811	104,690,838
YLLs	Neglected tropical diseases and malaria	1990	78,052,655	47,529,739	131,964,510
YLLs	Chronic respiratory diseases	1990	69,510,781	63,240,096	71,850,084
YLLs	Transport injuries	1990	67,250,972	63,271,345	71,109,915
YLLs	Self-harm and interpersonal violence	1990	62,747,860	58,214,706	65,136,524

 © 2019 Global Burden of Disease Cancer Collaboration. JAMA Oncol.

Measure	Cause Name	Year	Number	Lower 95% UI	Upper 95% UI
YLLs	Digestive diseases	1990	54,769,772	51,254,564	58,374,920
YLLs	Nutritional deficiencies	1990	42,318,629	35,764,357	48,169,160
YLLs	Diabetes and kidney diseases	1990	33,737,699	32,568,164	35,023,610
YLLs	HIV/AIDS and sexually transmitted infections	1990	31,782,945	24,110,761	42,038,212
YLLs	Neurological disorders	1990	22,971,446	21,064,118	24,355,263
YLLs	Substance use disorders	1990	7,700,956	7,465,383	8,027,134
YLLs	Musculoskeletal disorders	1990	1,821,191	1,674,988	2,034,286
YLLs	Skin and subcutaneous diseases	1990	1,545,876	915,953	1,837,119
YLLs	Mental disorders	1990	10,101	9,233	12,330

© 2019 Global Burden of Disease Cancer Collaboration. JAMA Oncol.

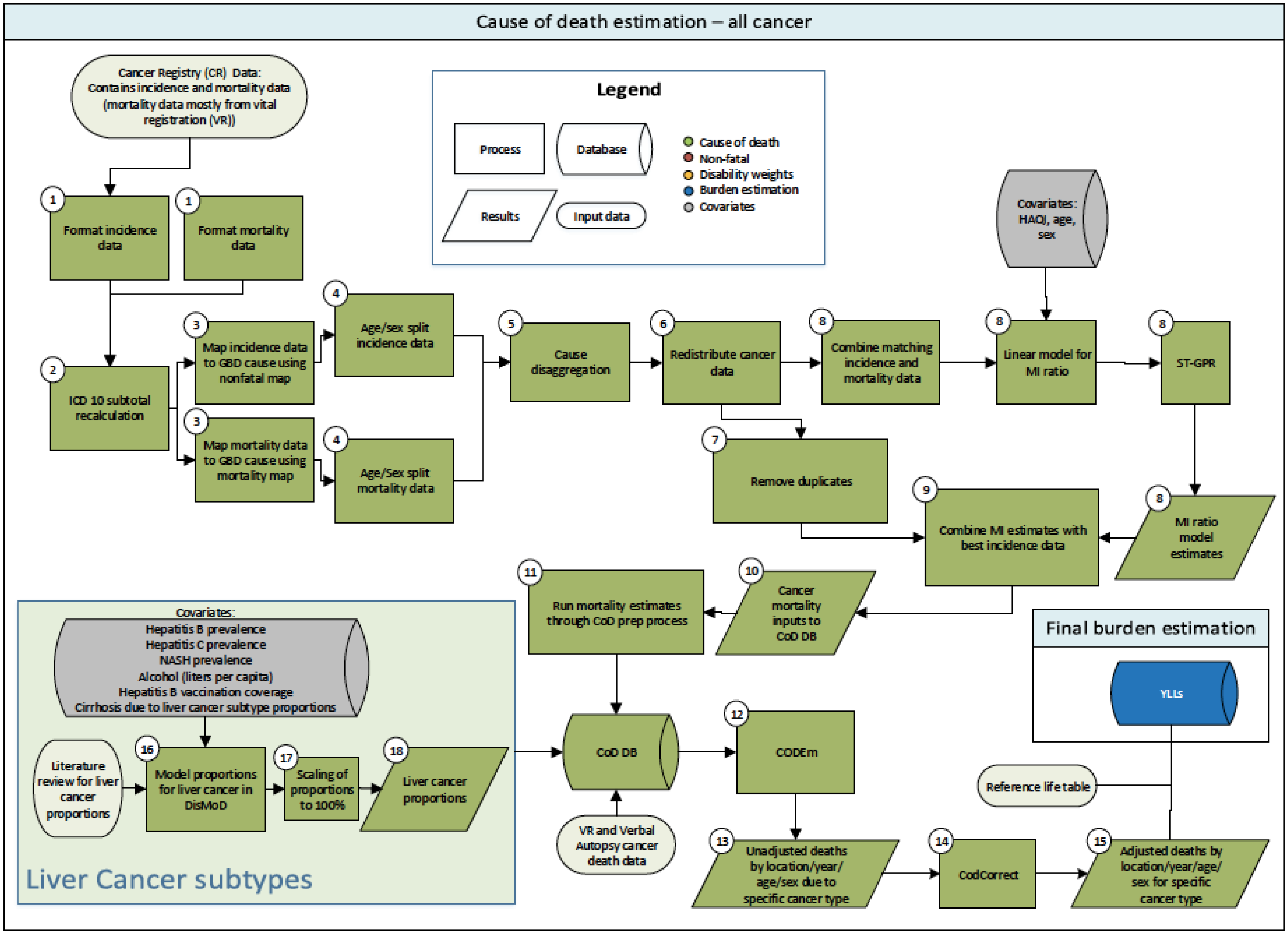

Abbreviations: ICD: International classification of diseases; DB: database, ST-GPR: Space-time smoothing, Gaussian process regression, COD: Causes of death

eFigure 1: Flowchart GBD cancer mortality, YLL estimation

© 2019 Global Burden of Disease Cancer Collaboration. JAMA Oncol.

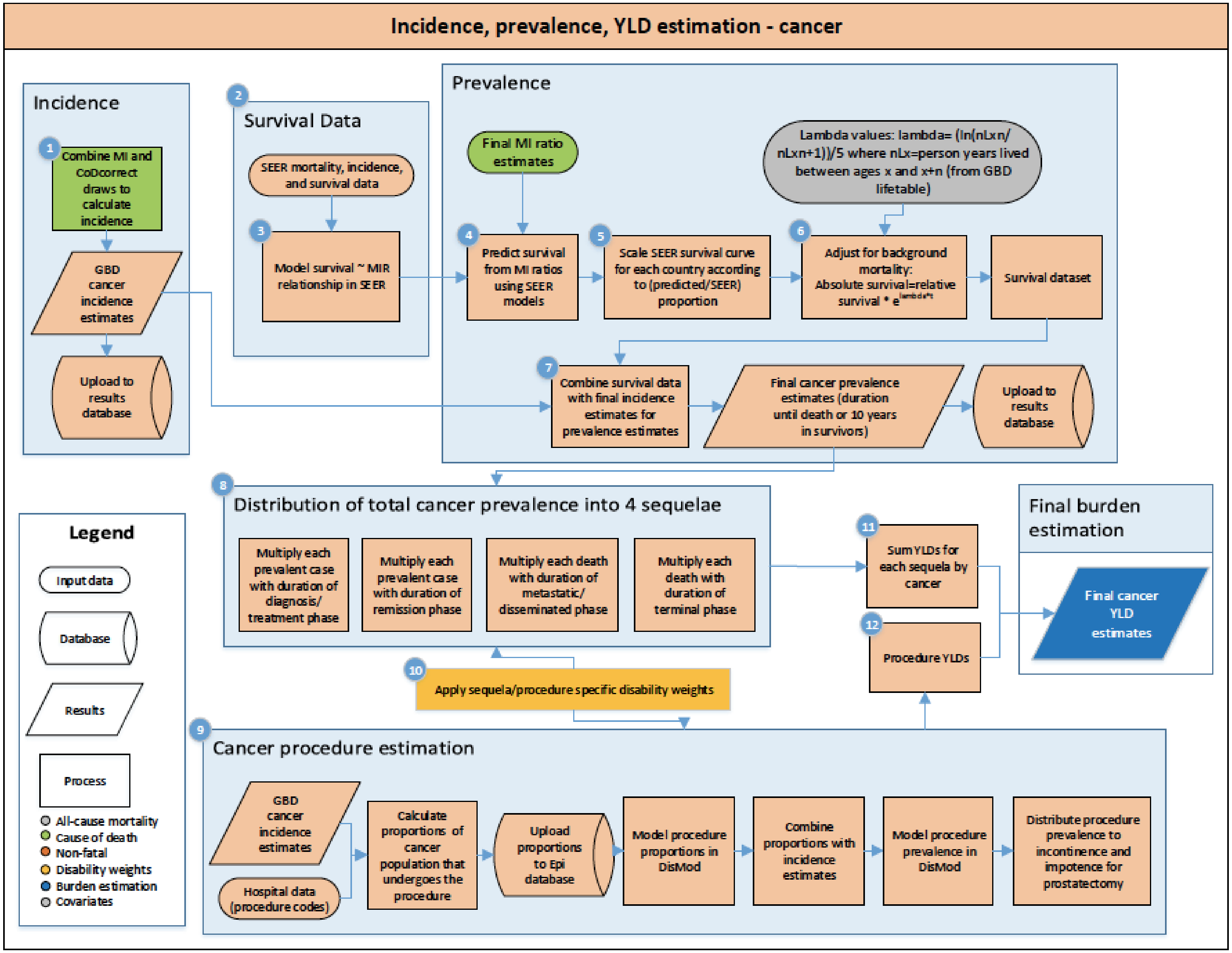

eFigure 2: Flowchart GBD cancer incidence, prevalence, YLD estimation

© 2019 Global Burden of Disease Cancer Collaboration. JAMA Oncol.

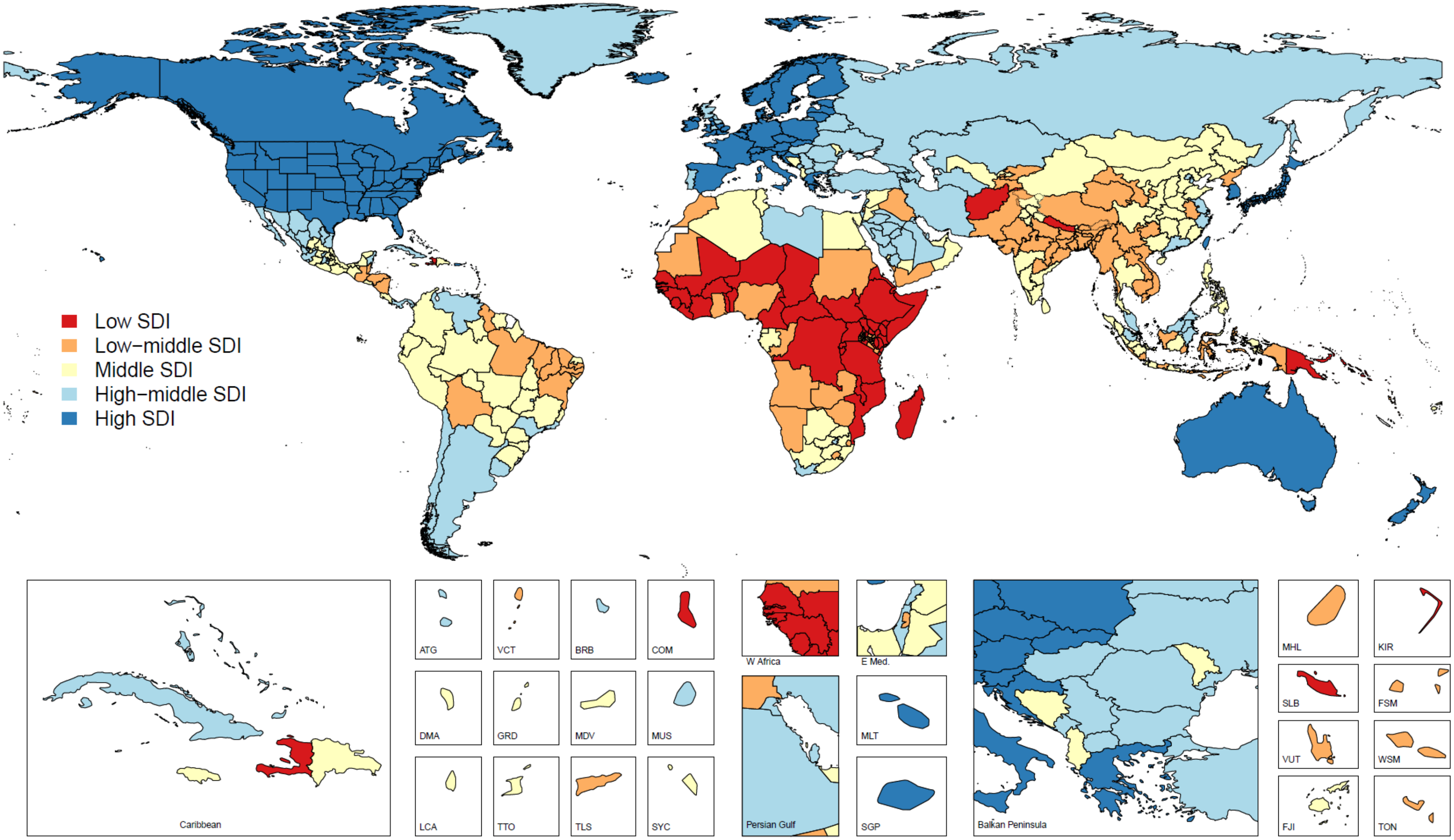

eFigure 3: Socio-demographic Index quintiles

© 2019 Global Burden of Disease Cancer Collaboration. JAMA Oncol.

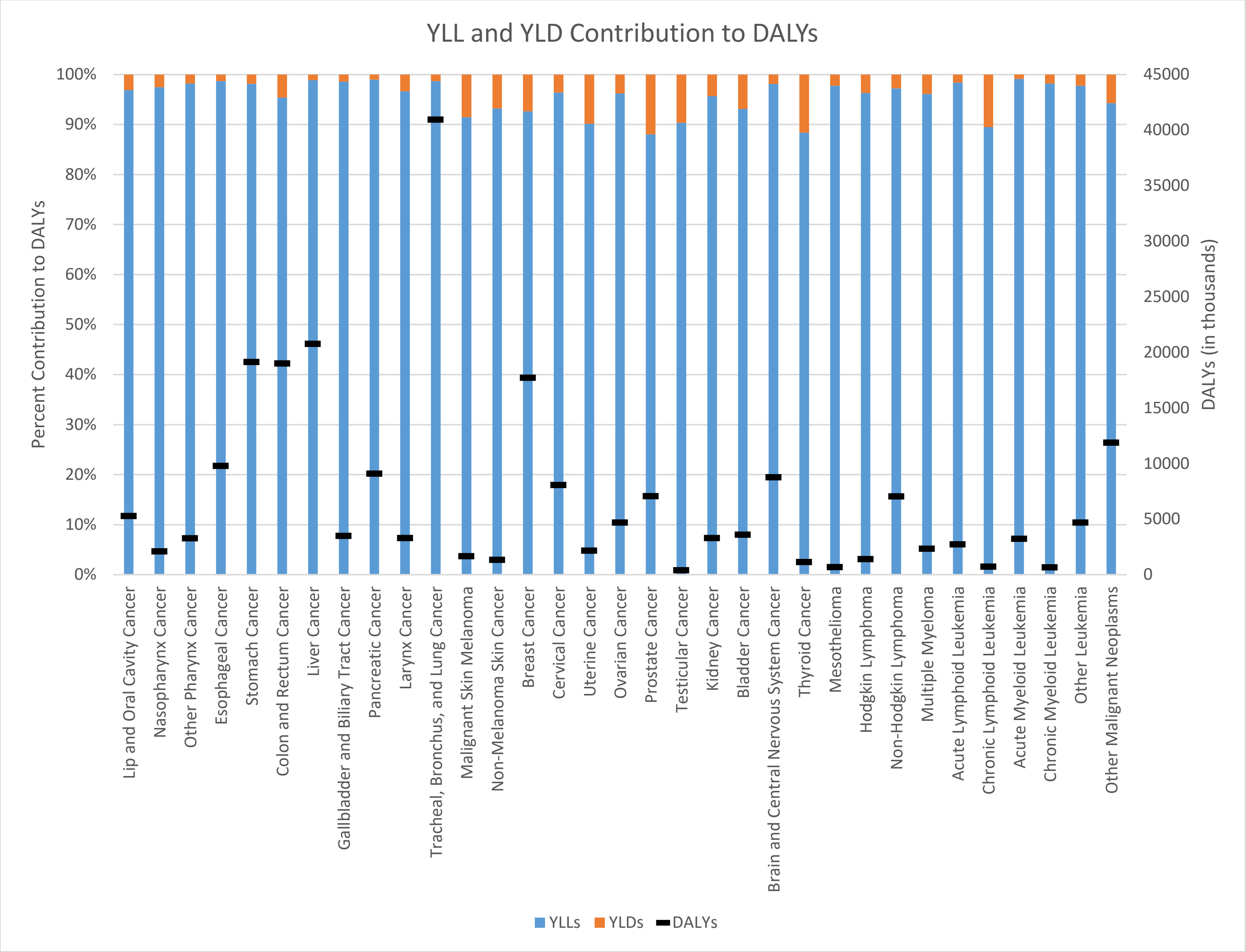

eFigure 4: Contribution of YLDs and YLLs to DALYs by cancer, global, both sexes, 2017

© 2019 Global Burden of Disease Cancer Collaboration. JAMA Oncol.

Country	Non-melanoma skin cancer	Tracheal, bronchus, and lung cancer	Breast cancer	Colon and rectum cancer	Prostate cancer	Stomach cancer	Liver cancer	Other malignant neoplasms	Cervical cancer	Non-Hodgkin lymphoma	Bladder cancer	Esophageal cancer	Pancreatic cancer	Uterine cancer	Brain and central nervous system cancer	Kidney cancer	Lip and oral cavity cancer	Malignant skin melanoma	Ovarian cancer	Thyroid cancer	Other leukemia	Gallbladder and biliary tract cancer	Larynx cancer	Other pharynx cancer	Multiple myeloma	Acute myeloid leukemia	Chronic lymphoid leukemia	Nasopharynx cancer	Acute lymphoid leukemia	Hodgkin lymphoma	Testicular cancer	Chronic myeloid leukemia	Mesothelioma
**Global**	1	2	3	4	5	6	7	8	9	10	11	12	13	14	15	16	17	18	19	20	21	22	23	24	25	26	27	28	29	30	31	32	33
**High SDI**	1	3	4	2	5	6	13	11	22	7	9	18	10	12	15	14	16	8	19	20	27	17	24	26	21	25	23	33	31	28	29	32	30
**High-middle SDI**	2	1	4	3	7	5	6	8	13	14	11	9	12	15	10	16	20	22	18	19	17	23	21	29	26	30	27	25	24	28	31	32	33
**Middle SDI**	1	2	3	5	7	4	6	9	8	13	17	10	15	16	11	18	12	29	19	20	14	23	21	25	27	26	28	22	24	30	31	32	33
**Low-middle SDI**	2	4	1	5	8	6	9	10	3	13	14	11	17	22	15	18	7	29	12	20	21	23	19	16	28	24	32	25	27	26	31	30	33
**Low SDI**	3	4	2	6	10	5	8	7	1	12	18	11	19	24	14	16	9	30	17	20	21	22	15	13	28	23	32	25	26	27	33	29	31
**South Asia**	8	3	1	5	12	6	14	9	4	15	20	10	19	22	16	21	2	31	13	17	24	18	11	7	26	23	33	25	27	28	30	29	32
India	8	6	1	4	10	5	13	9	3	16	21	11	18	22	15	20	2	31	14	19	25	17	12	7	26	23	33	24	27	29	30	28	32
Pakistan	25	3	1	7	19	17	13	5	10	6	11	9	26	15	12	24	2	30	4	8	23	18	14	16	28	20	33	27	22	21	29	31	32
Bangladesh	15	1	2	3	10	6	13	7	4	16	21	12	19	26	8	20	5	30	18	14	23	17	11	9	27	22	31	25	24	28	33	29	32
Nepal	19	2	1	6	10	4	12	8	3	17	21	7	14	23	15	20	5	30	16	18	24	13	11	9	26	22	33	25	27	28	32	29	31
Bhutan	19	5	1	3	8	6	11	7	4	14	21	10	16	24	12	20	2	31	18	15	23	17	13	9	27	22	33	25	26	28	30	29	32
**East Asia**	6	1	5	4	9	2	3	8	12	14	15	7	13	16	10	18	17	29	21	20	11	24	23	30	26	28	25	19	22	27	31	32	33
China	6	1	5	4	9	2	3	8	12	14	15	7	13	16	10	17	18	29	22	20	11	24	23	30	26	28	25	19	21	27	31	32	33
North Korea	19	2	4	5	8	3	1	9	6	18	12	7	15	11	14	13	20	29	16	21	10	22	23	27	26	24	30	17	25	28	33	31	32
Taiwan	9	3	4	1	5	7	2	8	17	11	12	10	14	13	19	15	6	29	20	18	26	22	23	16	25	24	31	21	30	28	27	32	33
**Southeast Asia**	5	2	1	3	7	8	4	9	6	14	13	21	19	18	15	16	10	30	11	12	17	22	23	26	27	24	33	20	25	28	31	29	32
Indonesia	1	3	2	5	8	7	6	9	4	15	11	26	16	17	13	14	19	30	10	20	12	22	21	25	27	23	32	18	24	28	33	29	31
Philippines	12	3	1	2	6	15	4	8	5	17	22	24	19	11	16	10	14	29	7	9	13	27	23	26	25	20	33	18	21	31	30	28	32
Vietnam	10	1	3	2	11	6	4	9	5	12	20	15	18	21	13	22	7	30	14	8	25	26	19	16	28	23	33	17	24	27	29	31	32
Thailand	10	2	3	4	6	11	1	8	5	20	12	17	14	19	15	21	7	29	13	18	16	9	22	26	27	24	32	23	25	30	31	28	33
Myanmar	10	2	1	4	8	6	5	11	3	16	14	7	17	15	18	13	19	31	9	21	12	23	22	26	27	24	32	20	25	28	33	30	29

© 2019 Global Burden of Disease Cancer Collaboration. JAMA Oncol.

Malaysia	9	3	1	2	5	7	8	6	4	12	14	22	19	15	18	17	13	31	16	11	27	26	23	25	28	21	32	10	20	29	24	30	33
Sri Lanka	8	6	1	2	5	9	17	4	10	13	16	11	20	14	21	7	3	30	15	12	18	22	25	19	27	24	33	26	23	28	32	31	29
Cambodia	7	1	2	4	8	5	6	9	3	14	13	20	19	16	17	15	12	30	11	21	10	23	22	26	27	25	32	18	24	28	33	29	31
Laos	7	1	2	4	9	6	5	10	3	14	15	20	17	19	13	12	16	30	11	21	8	25	23	26	28	24	32	18	22	27	33	29	31
Timor-Leste	8	1	2	4	5	7	6	9	3	12	11	22	17	18	14	15	16	30	13	21	10	25	24	26	28	23	32	19	20	27	33	29	31
Mauritius	9	4	1	2	3	5	14	8	6	16	12	19	13	7	20	15	10	29	11	18	17	23	21	25	22	26	32	24	28	27	30	31	33
Maldives	3	6	1	2	5	18	11	7	8	13	9	20	17	19	14	15	4	25	12	10	16	26	21	30	23	24	31	28	22	32	33	27	29
Seychelles	11	5	3	2	1	15	12	20	4	10	7	19	14	17	18	16	6	31	8	25	22	28	9	13	23	26	29	21	24	32	27	30	33
**North Africa and Middle East**	2	3	1	5	4	6	10	8	16	11	7	20	14	18	9	15	23	26	17	13	12	27	19	32	24	22	30	29	25	21	28	31	33
Egypt	10	5	2	4	6	14	1	7	19	13	3	20	17	18	9	12	22	28	11	15	8	24	21	27	23	25	32	29	26	16	33	30	31
Iran	1	6	2	5	3	4	15	8	19	11	9	13	14	20	7	16	24	25	17	12	10	28	18	32	26	21	29	33	23	22	27	31	30
Turkey	1	2	4	5	3	6	14	13	22	10	7	27	9	11	8	12	26	16	17	15	23	28	18	33	21	20	24	31	25	29	19	32	30
Iraq	7	2	1	4	10	12	11	8	19	17	6	24	13	16	3	9	22	32	14	15	5	25	21	27	23	18	31	29	20	26	30	28	33
Algeria	5	4	1	3	2	10	19	8	6	9	11	27	14	25	13	17	24	29	18	7	21	12	22	26	23	20	32	15	30	16	31	28	33
Sudan	1	6	3	4	5	2	14	9	13	15	10	11	19	26	8	12	22	28	21	20	7	24	18	31	25	16	30	27	17	23	32	29	33
Morocco	3	2	1	4	5	10	21	8	6	7	12	23	15	18	14	20	19	27	11	9	22	24	13	28	25	26	33	16	29	17	30	31	32
Saudi Arabia	14	8	1	3	2	13	10	6	20	5	11	24	16	18	7	15	17	30	19	4	9	27	25	31	23	26	32	21	28	12	22	29	33
Afghanistan	3	7	2	5	15	1	12	9	4	14	16	10	22	26	8	11	24	30	20	21	6	23	17	31	29	13	32	25	18	19	33	27	28
Yemen	2	4	3	5	6	1	15	8	10	14	11	12	20	25	9	13	22	29	21	19	7	24	17	31	27	16	30	26	18	23	32	28	33
Syria	3	6	1	5	2	9	10	12	18	14	8	25	13	19	7	15	22	28	17	21	4	27	23	29	26	11	20	30	16	31	33	24	32
Tunisia	3	2	1	4	5	9	23	8	14	7	6	26	15	19	13	18	17	25	16	10	12	20	11	28	24	27	31	22	30	21	29	32	33
Jordan	6	4	1	2	3	11	17	9	19	7	8	24	13	14	10	15	18	28	16	12	5	22	23	29	21	27	32	25	26	30	20	33	31
**United Arab Emirates**	3	8	2	4	5	13	15	1	17	9	7	11	14	27	6	10	18	25	20	12	19	29	16	30	23	21	32	26	28	22	24	31	33
Lebanon	8	7	1	3	2	14	23	4	19	5	6	28	18	13	10	22	24	20	15	11	12	25	17	31	21	26	29	30	27	9	16	32	33
Libya	8	3	1	2	4	14	16	7	12	6	5	26	15	20	9	17	22	29	18	10	11	23	19	32	24	25	30	21	28	13	27	31	33
Palestine	3	4	1	2	5	10	9	14	17	16	11	23	13	12	6	8	21	29	15	18	7	26	24	31	19	20	28	27	25	22	33	30	32
Oman	6	11	1	3	2	8	12	5	15	4	13	21	18	23	10	16	17	26	19	7	9	28	24	30	20	22	32	29	25	14	27	33	31
Kuwait	8	6	1	3	2	17	12	5	18	4	7	24	15	11	9	14	19	26	16	10	13	28	25	30	21	20	27	29	23	22	33	31	32
Qatar	3	6	1	4	2	11	9	5	19	8	10	25	16	18	7	13	22	26	17	12	15	28	23	31	24	14	27	32	20	21	30	29	33
Bahrain	5	4	1	3	2	11	16	9	17	7	6	22	12	10	14	8	20	29	13	15	18	26	25	30	21	19	27	28	24	23	33	31	32
**Western SSA**	4	8	1	7	3	9	5	6	2	10	15	13	12	21	14	11	20	27	16	28	19	24	23	30	22	17	31	25	26	18	33	29	32
Nigeria	5	7	1	6	2	14	13	4	3	8	19	16	12	23	11	9	21	26	15	28	18	24	22	31	20	17	30	25	27	10	33	29	32

© 2019 Global Burden of Disease Cancer Collaboration. JAMA Oncol.

Ghana	4	10	2	6	5	8	3	12	1	9	15	16	7	14	11	13	18	29	17	27	20	23	21	24	22	19	28	30	25	32	33	26	31
Cameroon	4	7	3	8	5	6	2	9	1	12	14	11	13	19	15	10	18	30	16	25	20	23	21	27	22	17	31	28	24	29	33	26	32
Cote d'Ivoire	3	8	2	9	1	10	5	7	4	6	11	22	15	20	17	13	14	30	12	25	23	24	19	27	18	16	32	21	28	26	33	29	31
Niger	3	8	6	9	5	4	2	7	1	11	17	12	16	20	15	10	18	29	19	28	13	22	21	30	23	14	31	27	24	25	33	26	32
Burkina Faso	4	9	2	5	7	6	3	8	1	13	15	11	14	19	10	12	20	30	18	25	17	23	21	29	22	16	31	28	24	26	33	27	32
Mali	3	10	6	8	9	5	1	4	2	11	7	16	13	22	19	12	17	24	20	18	15	27	26	31	23	14	32	28	25	21	30	29	33
Chad	2	7	6	9	4	5	3	8	1	11	13	12	17	20	15	10	18	30	19	28	14	22	21	29	24	16	31	26	23	25	33	27	32
Senegal	2	7	4	8	3	6	5	9	1	12	14	10	13	19	17	11	18	30	16	26	20	23	21	29	22	15	31	28	24	27	33	25	32
Guinea	5	8	3	9	7	4	1	6	2	12	11	17	15	16	19	13	10	24	14	27	21	23	20	22	28	18	33	26	29	25	31	30	32
Benin	2	8	4	9	5	6	3	7	1	12	14	10	13	20	16	11	19	30	17	25	18	23	22	29	21	15	31	28	24	27	33	26	32
Sierra Leone	3	7	2	8	5	6	4	9	1	12	14	11	13	20	15	10	19	30	17	25	18	23	21	29	22	16	31	27	24	28	33	26	32
Togo	3	7	2	8	6	5	4	9	1	12	14	10	13	18	16	11	17	30	15	25	20	23	21	29	22	19	31	27	26	28	33	24	32
Liberia	2	9	4	7	5	6	3	8	1	12	14	10	13	19	17	11	18	30	16	25	20	23	22	29	21	15	31	27	24	28	33	26	32
Mauritania	4	8	3	6	2	7	5	9	1	13	14	11	12	16	18	10	19	26	15	24	20	22	23	29	21	17	31	28	25	30	33	27	32
The Gambia	3	6	4	8	9	10	1	5	2	7	13	17	12	18	20	11	14	30	15	22	19	21	26	29	25	16	31	23	27	24	32	28	33
Guinea-Bissau	4	8	3	6	7	5	2	9	1	12	14	10	13	20	15	11	19	30	17	28	18	23	21	29	22	16	32	26	24	25	33	27	31
Cape Verde	4	6	3	9	1	2	7	13	5	16	14	8	10	17	15	11	12	29	18	24	20	25	23	22	21	19	30	28	27	32	31	26	33
Sao Tome and Principe	4	6	3	7	8	5	16	2	1	11	9	13	17	14	20	10	22	29	12	23	19	18	24	26	21	15	28	30	25	32	33	27	31
**Western Europe**	1	5	3	2	4	10	15	9	23	8	6	20	11	12	14	13	18	7	16	21	27	22	25	26	19	24	17	33	32	28	29	31	30
Germany	1	5	2	3	4	7	14	10	24	8	11	20	9	13	16	12	19	6	17	22	29	18	27	23	21	25	15	33	32	28	26	30	31
United Kingdom	1	4	2	3	5	9	19	8	22	7	10	15	11	12	14	13	20	6	16	24	30	28	27	25	18	21	17	33	31	26	29	32	23
France	1	4	2	3	5	12	14	7	24	9	6	22	10	11	13	15	16	8	18	25	23	27	21	19	20	26	17	33	32	29	28	31	30
Italy	1	4	3	2	5	7	13	9	25	8	6	27	12	10	15	14	21	11	20	16	24	19	22	29	18	23	17	33	31	26	28	32	30
England	1	5	2	3	4	9	19	8	22	7	10	15	11	12	14	13	20	6	16	24	30	28	27	25	18	21	17	33	31	26	29	32	23
Spain	1	3	4	2	5	7	15	11	22	9	6	24	13	10	14	8	16	12	18	23	25	20	17	26	19	27	21	31	29	28	30	32	33
Netherlands	1	5	3	2	4	11	20	8	24	9	7	15	12	10	13	14	19	6	17	21	16	23	25	29	18	22	30	33	31	26	27	32	28
Belgium	1	4	2	3	5	12	16	7	25	9	6	18	10	11	13	14	15	8	17	22	21	27	24	26	19	23	20	33	32	28	30	31	29
Portugal	1	5	4	2	3	6	16	10	17	8	7	22	11	9	12	14	15	13	20	18	27	25	23	19	24	26	21	29	31	28	30	32	33
Greece	1	3	2	4	5	8	13	11	21	15	6	28	10	9	7	14	24	12	16	26	19	25	18	32	23	22	17	30	31	20	27	29	33
Sweden	1	5	3	4	2	14	18	8	20	9	7	21	12	10	11	13	19	6	17	23	27	22	29	26	16	24	15	33	31	28	25	32	30
Israel	1	4	2	3	5	13	20	11	21	6	7	28	9	12	10	14	22	8	16	17	25	26	24	33	19	18	15	31	27	23	29	30	32
Austria	5	4	2	3	1	9	13	12	23	10	6	24	8	11	15	14	21	7	16	18	29	19	27	20	22	25	17	33	32	28	26	30	31

© 2019 Global Burden of Disease Cancer Collaboration. JAMA Oncol.

Switzerland	1	5	3	4	2	12	14	8	24	7	9	21	10	13	11	15	19	6	18	20	23	26	27	25	17	22	16	33	31	29	28	32	30
Denmark	1	3	4	2	5	14	20	9	22	10	7	17	11	12	8	13	18	6	16	24	26	25	27	21	19	23	15	33	32	29	28	31	30
Finland	1	5	3	4	2	14	15	8	25	6	13	22	9	12	10	11	18	7	16	20	26	21	29	27	17	23	19	33	31	24	28	32	30
Scotland	1	2	3	4	5	10	18	7	21	8	9	12	13	14	15	11	19	6	16	25	30	28	26	22	20	23	17	33	31	24	27	32	29
Norway	1	5	4	3	2	14	21	10	20	8	7	22	12	13	9	11	19	6	16	18	26	23	29	28	15	25	17	33	31	27	24	32	30
Ireland	1	5	2	3	4	11	20	8	19	7	10	15	13	12	9	14	21	6	16	22	26	27	25	29	18	23	17	33	30	24	28	31	32
Wales	1	3	4	2	5	9	19	8	22	7	10	14	12	11	15	13	20	6	16	25	30	29	27	23	18	21	17	33	31	24	28	32	26
Northern Ireland	1	3	2	4	5	8	20	9	21	7	10	16	11	12	13	14	19	6	15	25	30	27	26	29	17	22	18	33	31	23	24	32	28
Cyprus	1	5	2	4	3	10	16	8	22	7	6	28	13	9	12	17	21	11	15	19	23	26	24	30	18	20	14	31	27	25	33	32	29
Luxembourg	1	4	2	3	5	13	15	9	26	11	10	21	12	7	8	19	17	6	14	16	22	29	23	25	20	24	18	32	31	28	27	33	30
Malta	1	4	2	3	5	11	22	9	26	8	6	25	10	7	12	14	17	13	15	16	32	27	20	29	19	18	21	28	31	24	23	33	30
Iceland	1	4	3	5	2	13	20	7	24	11	9	17	14	15	6	10	18	8	19	12	22	27	29	30	16	23	21	33	28	25	26	31	32
Andorra	1	5	2	4	3	11	21	9	23	8	6	20	13	12	10	14	17	7	15	19	30	26	28	29	18	22	16	33	31	24	25	32	27
**Eastern SSA**	1	10	3	5	7	11	8	4	2	6	16	9	18	23	12	14	15	28	13	17	21	27	26	30	25	19	31	24	20	22	33	29	32
Ethiopia	1	7	3	4	12	9	15	5	2	6	19	14	22	26	8	13	16	30	10	11	24	25	28	31	27	18	29	23	17	20	33	21	32
Tanzania	3	9	4	6	5	10	8	2	1	7	16	14	15	20	12	11	22	27	13	21	18	29	26	28	23	17	31	24	19	25	33	30	32
Kenya	1	10	3	5	6	7	9	4	2	11	22	8	14	25	15	17	12	29	13	27	24	21	18	26	19	20	30	16	23	28	31	32	33
Uganda	9	10	4	8	3	11	7	1	2	5	20	6	16	19	15	12	14	27	13	17	23	29	28	25	24	21	32	18	26	22	31	30	33
Mozambique	4	6	3	5	18	10	2	7	1	11	19	8	22	20	9	15	17	26	13	21	12	27	24	28	25	14	30	31	16	23	33	29	32
Madagascar	3	10	2	5	6	8	11	4	1	9	16	7	18	19	13	14	15	27	12	21	17	29	25	28	26	20	31	22	24	23	33	30	32
Zambia	4	9	3	5	6	10	8	2	1	7	15	14	16	20	11	12	19	27	13	22	18	29	25	28	26	17	31	23	21	24	33	30	32
Malawi	6	12	5	9	8	13	10	2	1	4	7	3	16	20	19	11	14	15	17	18	22	28	27	32	23	21	31	26	24	25	29	30	33
Somalia	3	11	4	5	7	9	10	2	1	8	18	6	20	22	14	12	17	27	13	25	15	28	24	29	26	16	31	23	21	19	33	30	32
Rwanda	3	10	2	6	5	11	8	4	1	7	17	9	16	19	14	13	15	27	12	20	21	29	25	28	23	18	31	24	22	26	33	30	32
Burundi	2	10	4	7	6	9	11	3	1	8	18	5	17	23	13	12	14	28	15	20	16	29	21	27	26	19	31	22	25	24	33	30	32
South Sudan	3	10	4	6	8	11	9	2	1	7	18	5	16	20	13	12	17	27	14	24	15	29	25	28	26	19	31	22	23	21	32	30	33
Eritrea	4	11	2	5	7	8	9	3	1	6	16	10	18	20	13	14	15	26	12	23	19	29	25	28	27	17	31	21	24	22	33	30	32
Djibouti	3	7	2	5	6	11	9	4	1	8	14	10	15	18	16	13	17	27	12	20	24	29	23	28	22	19	31	21	25	26	33	30	32
Comoros	3	8	2	6	5	11	9	4	1	10	14	7	13	18	16	17	15	27	12	20	23	28	24	29	21	19	31	22	26	25	33	30	32
**High-income North America**	1	4	3	5	2	13	14	12	21	7	10	20	11	8	15	9	16	6	19	18	26	27	22	25	17	24	23	33	30	28	29	32	31
United States	1	4	3	5	2	14	13	12	20	7	11	21	10	8	16	9	15	6	19	17	26	27	22	25	18	24	23	32	30	28	29	33	31
Canada	1	3	4	2	5	9	15	10	22	6	8	18	12	11	13	14	19	7	17	21	27	23	24	29	16	26	20	33	31	25	28	32	30

© 2019 Global Burden of Disease Cancer Collaboration. JAMA Oncol.

**Central Latin America**	1	7	3	4	2	5	9	11	6	14	18	27	12	10	16	8	21	25	13	15	23	20	26	31	24	22	30	32	17	28	19	29	33
Mexico	1	7	3	4	2	6	9	10	5	16	19	27	13	12	18	8	21	23	11	14	25	20	26	31	24	22	30	33	17	28	15	29	32
Colombia	1	6	3	4	2	5	11	8	7	10	17	26	12	9	16	15	21	20	13	14	23	19	27	31	24	25	29	32	18	28	22	30	33
Venezuela	1	6	3	5	2	7	11	10	4	13	15	24	12	9	18	8	19	26	14	16	28	25	17	29	22	21	31	32	20	27	23	30	33
Guatemala	1	8	6	7	4	2	5	12	3	16	22	21	14	10	15	9	20	28	17	18	11	19	25	29	26	23	32	27	13	30	24	31	33
Honduras	1	6	2	5	3	7	20	11	8	18	21	26	15	4	19	14	17	29	9	23	13	16	27	24	22	12	30	28	10	32	33	25	31
Nicaragua	1	8	3	5	4	6	7	9	2	15	19	26	11	16	17	10	21	27	12	14	20	18	24	32	25	22	29	31	13	30	23	28	33
El Salvador	1	7	3	6	2	5	9	13	4	16	19	22	11	8	15	14	20	29	12	18	10	17	24	26	23	25	31	28	21	27	30	32	33
Costa Rica	1	7	3	5	2	4	10	12	6	9	14	27	11	8	16	13	22	18	17	15	28	24	26	31	20	23	30	32	21	25	19	29	33
Panama	3	7	2	4	1	5	10	9	6	13	17	25	12	8	14	11	18	26	16	15	23	24	22	28	20	21	31	29	19	30	27	32	33
**Tropical Latin America**	1	5	2	4	3	7	12	8	6	16	14	13	9	17	10	11	15	21	18	20	26	23	19	22	25	24	30	32	28	29	27	33	31
Brazil	1	5	2	4	3	7	12	8	6	16	14	13	9	17	10	11	15	21	18	20	26	23	19	22	25	24	30	32	28	29	27	33	31
Paraguay	8	4	1	5	3	6	15	7	2	14	18	13	10	11	20	9	17	27	12	16	19	22	23	26	28	21	30	32	24	29	25	31	33
**Eastern Europe**	1	4	3	2	6	5	19	7	12	14	11	21	10	9	16	8	17	13	15	18	27	26	20	22	24	28	25	32	29	23	30	31	33
Russian Federation	1	4	2	3	6	5	19	7	12	14	11	20	10	9	17	8	18	13	15	16	27	26	21	23	24	28	25	33	29	22	30	31	32
Ukraine	1	3	4	2	7	5	20	6	16	17	10	22	11	8	12	9	13	14	15	19	25	26	18	21	27	28	24	31	30	23	29	32	33
Belarus	1	4	5	2	3	6	22	7	9	14	11	21	12	13	16	8	15	10	17	19	26	27	18	20	24	28	25	32	29	23	30	31	33
Moldova	1	4	3	2	5	6	7	13	8	17	12	22	9	11	16	10	18	20	19	21	23	28	14	15	25	27	30	26	29	24	31	33	32
Lithuania	2	4	5	3	1	6	22	9	17	13	11	19	10	7	15	8	18	12	14	20	28	25	16	21	24	26	23	32	30	27	29	31	33
Latvia	1	3	4	2	5	6	21	14	20	13	8	17	10	7	15	9	16	11	12	18	27	26	19	24	22	28	23	32	30	25	29	31	33
Estonia	1	4	5	3	2	6	22	13	16	12	11	21	9	7	15	8	18	10	14	17	26	28	23	24	19	25	20	32	29	27	30	31	33
**High-income Asia Pacific**	9	2	4	1	5	3	6	10	16	11	12	13	7	18	17	15	19	24	20	14	26	8	22	25	21	23	32	31	28	29	27	33	30
Japan	9	2	4	1	5	3	6	11	17	10	12	13	7	15	19	14	16	25	20	18	26	8	24	23	21	22	31	32	28	30	27	33	29
South Korea	14	2	5	1	6	3	4	10	16	12	11	17	9	19	13	15	20	24	18	7	22	8	21	25	23	26	31	29	27	28	32	30	33
Singapore	7	3	2	1	4	6	5	8	13	9	16	20	12	11	10	17	19	21	15	14	29	24	23	27	25	22	30	18	28	26	31	32	33
Brunei	6	3	1	2	9	5	8	11	4	7	17	27	18	16	15	12	14	24	10	13	31	23	29	22	21	20	33	19	30	25	28	26	32
**Central SSA**	1	4	3	5	9	10	7	6	2	15	12	8	16	20	13	11	17	28	18	25	14	22	21	30	23	19	33	27	24	26	31	29	32
DRC	1	4	3	5	9	8	7	6	2	16	14	10	18	20	12	11	15	29	17	26	13	22	21	30	24	19	33	27	23	25	31	28	32
Angola	1	4	3	5	9	10	7	6	2	15	12	8	14	20	13	11	17	28	16	25	18	24	21	30	22	19	33	26	23	27	31	29	32
Congo	2	5	3	4	6	10	9	8	1	16	12	7	14	19	15	11	17	26	13	22	20	24	21	30	23	18	33	25	27	29	32	28	31
CAR	2	4	3	7	10	5	9	8	1	15	17	6	16	21	12	11	13	29	18	28	14	23	19	30	25	20	33	24	26	22	31	27	32

© 2019 Global Burden of Disease Cancer Collaboration. JAMA Oncol.

Gabon	1	4	3	5	6	11	8	9	2	16	10	7	12	19	17	13	14	26	15	24	21	23	20	25	22	18	33	27	28	30	32	29	31
Equatorial Guinea	1	5	3	4	8	14	6	9	2	12	15	7	11	19	17	10	16	25	13	21	20	24	23	27	22	18	32	28	26	29	31	30	33
**Central Europe**	1	2	4	3	5	8	17	10	13	14	6	24	9	7	15	11	18	12	16	22	29	20	19	21	26	28	23	32	30	27	25	31	33
Poland	1	2	4	3	5	9	22	8	16	13	6	23	10	7	12	11	17	15	14	19	29	20	18	24	26	28	21	33	31	27	25	32	30
Romania	1	3	4	2	5	8	9	11	6	17	7	22	10	12	14	13	15	19	18	21	25	26	16	20	27	28	23	30	31	29	24	32	33
Czech Republic	1	3	4	2	5	12	18	9	16	13	7	21	10	11	17	8	19	6	14	20	28	15	25	24	23	29	26	33	30	27	22	31	32
Hungary	1	3	4	2	5	9	19	15	16	13	6	22	7	10	20	8	12	11	18	24	28	21	17	14	26	27	23	30	31	29	25	32	33
Serbia	1	2	4	3	5	10	15	8	11	16	6	25	9	7	12	13	19	14	18	22	20	21	17	24	28	29	27	31	30	26	23	32	33
Bulgaria	1	4	3	2	5	8	13	11	10	14	7	24	9	6	12	15	19	18	16	20	25	23	17	21	29	27	28	32	30	22	26	31	33
Slovakia	1	3	4	2	5	6	20	15	13	12	10	24	9	8	17	7	18	11	16	26	29	14	25	19	21	28	22	31	30	27	23	32	33
Croatia	1	3	4	2	5	8	15	14	17	13	7	25	11	6	10	9	19	12	16	24	29	18	20	22	23	28	21	33	31	27	26	32	30
Bosnia and Herzegovina	1	2	4	3	5	6	9	13	14	17	8	23	7	11	12	10	21	16	15	20	30	19	18	24	26	25	22	32	29	28	27	31	33
Albania	1	2	3	5	4	7	9	6	13	15	17	25	10	11	8	12	18	20	19	16	26	27	14	29	30	22	23	32	28	24	21	31	33
Macedonia	1	2	3	4	5	6	11	13	14	18	7	26	10	8	12	17	21	9	16	19	27	23	15	28	29	25	22	32	30	24	20	31	33
Slovenia	1	3	5	2	4	8	15	10	21	7	12	26	9	11	17	13	20	6	19	22	29	16	24	18	23	25	14	33	31	30	27	28	32
Montenegro	1	2	3	4	5	11	14	19	12	17	6	23	7	10	8	13	20	15	18	16	31	24	9	27	28	26	21	33	29	22	25	30	32
**Central Asia**	1	3	2	5	11	4	7	9	6	17	15	13	14	10	12	8	18	23	16	20	21	27	19	26	29	22	30	31	25	24	28	32	33
Uzbekistan	1	5	2	6	14	3	8	10	4	13	16	12	17	9	7	11	15	26	18	25	20	31	19	22	29	21	30	27	23	24	28	32	33
Kazakhstan	1	3	2	4	10	5	12	8	6	19	15	11	13	9	16	7	18	20	14	17	27	26	21	25	30	22	29	31	24	23	28	32	33
Azerbaijan	1	3	2	5	7	4	9	10	11	19	15	8	14	13	12	6	21	25	17	20	16	24	18	28	27	23	29	31	26	22	32	30	33
Tajikistan	1	6	3	5	14	2	9	7	12	13	18	11	15	10	4	8	21	25	16	26	17	28	22	24	27	19	32	23	20	30	33	31	29
Kyrgyzstan	1	5	2	6	14	3	7	8	4	19	16	15	12	10	13	9	17	22	11	18	20	26	24	25	29	21	31	30	23	27	28	32	33
Turkmenistan	1	4	2	7	12	5	10	9	3	17	16	8	15	20	11	6	14	28	13	18	19	29	26	21	24	22	31	30	25	23	27	32	33
Georgia	1	3	2	4	7	5	10	11	8	18	9	22	13	6	15	12	17	20	14	21	19	23	16	25	28	27	29	32	30	26	24	33	31
Mongolia	2	4	7	8	17	3	1	9	6	16	20	5	11	13	12	10	15	30	14	21	24	19	22	25	26	18	32	27	23	29	33	28	31
Armenia	1	3	2	4	6	5	9	8	12	17	7	21	10	11	13	14	20	23	15	18	19	22	16	30	27	26	24	33	28	32	25	31	29
**Southern SSA**	1	5	3	6	4	10	8	9	2	16	12	7	11	18	19	15	13	23	14	24	17	25	21	26	20	22	31	28	32	29	30	33	27
South Africa	1	4	3	6	5	10	8	14	2	17	11	7	9	18	19	12	13	23	15	24	16	25	21	27	20	22	31	28	32	29	30	33	26
Zimbabwe	11	9	2	7	3	8	4	5	1	10	12	6	13	16	15	19	18	24	14	22	21	25	23	28	17	20	33	27	29	26	30	32	31
Namibia	1	8	2	6	4	13	11	5	3	9	18	14	12	20	19	16	7	10	17	24	21	29	15	22	25	23	32	28	31	26	30	33	27
Botswana	1	5	3	6	4	12	8	9	2	16	14	7	10	17	18	15	13	20	11	23	19	25	24	26	21	22	31	27	30	29	32	33	28
Lesotho	1	4	3	8	6	9	7	10	2	15	16	5	12	18	20	14	11	22	13	25	17	26	19	28	23	21	33	27	31	29	30	32	24

© 2019 Global Burden of Disease Cancer Collaboration. JAMA Oncol.

Swaziland	1	5	3	8	7	10	6	9	2	15	16	4	11	19	17	12	13	22	14	24	18	27	20	26	23	21	32	25	30	29	31	33	28
**Southern Latin America**	3	5	1	2	4	7	15	12	6	13	11	17	9	14	19	8	22	20	18	21	25	10	23	30	24	26	29	33	27	28	16	31	32
Argentina	3	4	1	2	5	8	17	12	6	14	10	15	9	13	18	7	20	21	16	23	24	11	22	30	25	26	29	33	27	28	19	32	31
Chile	2	6	4	3	1	5	16	13	8	14	11	18	12	15	22	9	23	20	19	17	25	7	26	30	21	24	29	33	27	28	10	31	32
Uruguay	1	4	2	3	5	7	22	15	8	12	10	14	9	13	20	6	17	18	16	24	23	11	19	28	21	27	26	32	31	29	25	30	33
**Andean Latin America**	1	7	4	5	2	3	8	12	6	9	19	25	14	10	16	11	21	24	15	13	18	17	27	28	23	22	31	33	20	29	26	30	32
Peru	1	7	4	3	2	5	8	12	6	10	19	26	13	11	16	9	20	24	15	14	18	17	28	27	22	23	31	33	21	29	25	30	32
Ecuador	1	7	4	5	2	3	8	12	6	11	20	26	14	9	15	13	22	23	16	10	19	18	27	28	24	21	31	32	17	29	25	30	33
Bolivia	1	7	5	6	4	2	8	9	3	10	19	22	13	12	18	11	21	25	16	15	17	14	26	27	24	23	33	32	20	28	29	30	31
**Caribbean**	4	5	2	3	1	8	11	9	6	12	10	17	14	7	19	15	16	25	18	20	21	26	13	23	22	24	28	30	29	27	32	31	33
Haiti	4	7	3	6	2	5	10	8	1	11	14	16	20	13	17	9	18	29	15	23	12	22	19	27	24	21	32	28	25	26	33	30	31
Cuba	5	2	4	3	1	10	17	11	9	13	8	15	14	6	18	16	12	23	20	19	28	27	7	22	21	26	25	32	30	24	31	29	33
Dominican Republic	3	6	2	4	1	8	7	9	5	15	19	21	10	11	16	14	12	28	18	17	13	26	20	22	23	24	30	25	27	29	33	31	32
Jamaica	6	4	2	3	1	7	15	10	5	9	12	19	14	8	22	16	20	25	11	17	13	23	21	26	18	24	29	27	30	31	32	28	33
Trinidad and Tobago	7	5	2	3	1	13	15	10	4	12	14	23	11	6	21	8	18	27	9	17	19	24	22	25	16	20	31	30	26	29	32	28	33
Guyana	4	7	2	5	1	8	12	11	3	14	16	19	13	6	20	10	15	26	9	17	18	24	22	27	23	31	30	32	21	28	33	25	29
Suriname	5	6	2	3	1	9	7	8	4	13	16	21	10	15	14	11	19	26	12	18	17	24	22	28	20	25	31	23	30	27	29	32	33
Belize	3	5	4	6	1	8	7	11	2	16	14	19	12	10	15	9	18	25	17	21	13	23	20	27	22	30	32	29	24	26	31	33	28
The Bahamas	4	6	1	3	2	8	13	11	5	12	20	16	18	7	21	10	14	22	9	17	26	25	19	23	15	24	27	30	31	29	33	28	32
Barbados	4	8	2	3	1	7	16	11	6	9	14	17	12	5	22	10	19	25	15	18	20	26	21	24	13	23	27	29	31	30	33	28	32
Saint Lucia	3	6	2	5	1	7	16	12	4	9	10	19	11	8	21	15	14	23	13	17	22	27	20	24	18	26	25	28	32	30	29	31	33
Saint Vincent and the Grenadines	4	7	2	5	1	6	12	10	3	9	15	20	14	8	22	13	11	24	16	17	19	27	18	23	21	26	25	30	31	29	32	28	33
Grenada	4	7	2	3	1	8	13	11	5	9	15	12	10	6	22	17	16	26	14	19	20	24	21	25	18	28	23	29	31	30	33	27	32
Antigua and Barbuda	4	8	2	3	1	6	10	9	5	12	14	21	15	7	20	13	19	22	11	16	17	26	23	25	18	24	32	30	29	31	27	28	33
Dominica	4	7	2	3	1	5	12	8	6	9	13	17	14	11	24	10	15	27	20	21	18	25	19	22	16	26	30	31	23	29	33	28	32
**Australasia**	1	6	5	3	2	9	17	8	24	7	11	18	10	13	15	12	14	4	20	19	25	23	30	27	16	22	21	33	31	28	29	32	26
Australia	1	6	5	3	2	9	17	8	25	7	11	18	10	13	15	12	14	4	20	19	26	23	29	27	16	22	21	33	31	28	30	32	24
New Zealand	1	6	4	3	2	11	18	8	23	7	10	16	12	13	15	9	17	5	19	21	28	22	27	29	14	24	20	33	31	26	25	32	30
**Oceania**	4	3	2	6	10	5	7	8	1	15	18	22	19	9	17	16	11	29	12	21	13	25	23	24	26	20	32	14	28	31	27	30	33
Papua New Guinea	6	2	3	7	10	4	5	8	1	15	18	23	20	9	17	16	14	30	12	21	11	25	22	24	27	19	33	13	26	29	31	28	32

© 2019 Global Burden of Disease Cancer Collaboration. JAMA Oncol.

Fiji	3	8	1	4	5	10	6	9	2	17	14	18	15	7	20	16	11	29	19	12	21	26	23	22	24	13	30	27	28	31	25	33	32
Solomon Islands	4	3	2	7	9	5	6	8	1	16	18	21	17	10	19	13	15	29	11	22	12	26	23	24	25	20	32	14	27	30	31	28	33
Vanuatu	1	4	2	7	6	8	5	9	3	17	14	23	18	10	20	15	11	26	13	21	12	24	22	25	28	16	31	19	27	29	33	30	32
Samoa	1	7	2	4	6	5	11	9	3	10	16	24	15	8	20	18	17	22	12	13	14	25	27	32	23	21	30	19	29	28	26	31	33
Kiribati	6	3	2	8	13	7	5	9	1	16	20	12	17	10	21	11	4	30	18	28	15	24	25	22	26	19	31	23	29	32	14	27	33
Federated States of Micronesia	3	2	1	5	9	7	6	10	4	16	15	22	14	8	19	12	13	27	11	20	18	26	23	24	25	21	30	17	28	33	32	29	31
Tonga	2	3	1	9	6	8	4	12	5	11	17	19	13	10	18	14	16	28	15	20	21	27	25	26	24	22	30	23	29	31	7	32	33
Marshall Islands	4	3	1	5	9	7	6	10	2	15	17	21	14	8	20	12	13	27	11	18	19	26	23	24	25	22	31	16	28	32	29	30	33

Colors correspond to the ranking, with dark red as the most common cancer and dark green as the least common cancer for the location indicated. Rankings do not include the "other malignant cancer" group. The numbers inside each box indicate the ranking. Abbreviations: SSA: Sub-Saharan Africa; DRC: Democratic Republic of Congo; CAR: Central African Republic

eFigure 5: Cancer ranking by total incidence based on global level for developing and developed regions and all countries, both sexes, 2017

© 2019 Global Burden of Disease Cancer Collaboration. JAMA Oncol.

Country	Tracheal, bronchus, and lung cancer	Colon and rectum cancer	Stomach cancer	Liver cancer	Breast cancer	Pancreatic cancer	Esophageal cancer	Prostate cancer	Other malignant neoplasms	Cervical cancer	Non-Hodgkin lymphoma	Brain and central nervous system cancer	Bladder cancer	Lip and oral cavity cancer	Ovarian cancer	Gallbladder and biliary tract cancer	Kidney cancer	Other leukemia	Larynx cancer	Other pharynx cancer	Multiple myeloma	Acute myeloid leukemia	Uterine cancer	Nasopharynx cancer	Non-melanoma skin cancer	Malignant skin melanoma	Acute lymphoid leukemia	Thyroid cancer	Chronic lymphoid leukemia	Hodgkin lymphoma	Mesothelioma	Chronic myeloid leukemia	Testicular cancer
**Global**	**1**	**2**	**3**	**4**	**5**	**6**	**7**	**8**	**9**	**10**	**11**	**12**	**13**	**14**	**15**	**16**	**17**	**18**	**19**	**20**	**21**	**22**	**23**	**24**	**25**	**26**	**27**	**28**	**29**	**30**	**31**	**32**	**33**
**High SDI**	**1**	**2**	**5**	**7**	**4**	**3**	**10**	**6**	**11**	**20**	**8**	**13**	**9**	**19**	**14**	**15**	**12**	**21**	**23**	**24**	**16**	**17**	**22**	**29**	**27**	**18**	**31**	**28**	**25**	**32**	**26**	**30**	**33**
**High-middle SDI**	**1**	**4**	**2**	**3**	**6**	**7**	**5**	**9**	**8**	**13**	**11**	**10**	**12**	**18**	**14**	**16**	**15**	**17**	**19**	**26**	**20**	**23**	**21**	**22**	**24**	**25**	**27**	**28**	**29**	**30**	**31**	**32**	**33**
**Middle SDI**	**1**	**4**	**3**	**2**	**6**	**9**	**5**	**7**	**8**	**10**	**12**	**11**	**17**	**13**	**16**	**15**	**21**	**14**	**18**	**20**	**24**	**25**	**23**	**19**	**22**	**28**	**26**	**27**	**30**	**29**	**32**	**31**	**33**
**Low-middle SDI**	**1**	**4**	**3**	**5**	**2**	**12**	**10**	**7**	**6**	**8**	**11**	**14**	**19**	**9**	**16**	**17**	**25**	**18**	**15**	**13**	**22**	**20**	**21**	**23**	**27**	**30**	**26**	**28**	**32**	**24**	**31**	**29**	**33**
**Low SDI**	**1**	**4**	**2**	**7**	**3**	**15**	**9**	**8**	**6**	**5**	**11**	**13**	**19**	**10**	**17**	**16**	**27**	**18**	**14**	**12**	**21**	**20**	**24**	**22**	**26**	**30**	**23**	**28**	**31**	**25**	**32**	**29**	**33**
**South Asia**	**1**	**5**	**3**	**10**	**2**	**16**	**8**	**13**	**7**	**9**	**14**	**15**	**18**	**4**	**17**	**12**	**28**	**20**	**11**	**6**	**21**	**19**	**23**	**22**	**26**	**30**	**24**	**25**	**33**	**27**	**31**	**29**	**32**
India	1	4	2	10	3	12	9	13	7	8	16	15	19	5	17	14	25	20	11	6	22	18	23	21	24	31	27	26	32	29	30	28	33
Pakistan	3	7	11	9	1	20	5	16	4	18	6	12	15	2	8	13	27	22	10	14	24	21	17	26	28	30	23	25	33	19	32	29	31
Bangladesh	1	2	3	13	4	15	11	10	6	8	16	7	20	5	18	14	25	19	12	9	22	17	26	23	24	30	21	27	31	28	32	29	33
Nepal	1	4	2	10	3	14	5	12	8	7	16	15	19	6	17	13	26	20	11	9	21	18	25	22	24	30	23	27	32	28	31	29	33
Bhutan	1	2	4	8	3	12	7	10	6	11	16	13	19	5	18	14	24	20	15	9	21	17	27	23	25	30	22	26	32	28	31	29	33
**East Asia**	**1**	**5**	**3**	**2**	**6**	**7**	**4**	**10**	**8**	**11**	**12**	**9**	**14**	**18**	**17**	**15**	**20**	**13**	**19**	**27**	**22**	**25**	**23**	**16**	**21**	**28**	**24**	**26**	**29**	**31**	**30**	**32**	**33**
China	1	5	3	2	6	7	4	10	8	11	12	9	14	18	17	15	20	13	19	27	22	25	23	16	21	28	24	26	29	31	30	32	33
North Korea	2	4	3	1	6	10	5	9	8	7	13	11	14	20	17	15	21	12	18	27	25	24	19	16	22	28	23	26	31	29	30	32	33
Taiwan	1	3	4	2	5	7	6	9	10	16	11	18	12	8	19	17	14	22	24	13	21	20	23	15	26	27	28	25	29	32	30	31	33
**Southeast Asia**	**1**	**3**	**5**	**2**	**4**	**9**	**13**	**8**	**6**	**7**	**11**	**10**	**18**	**15**	**14**	**16**	**23**	**12**	**19**	**24**	**27**	**20**	**22**	**17**	**26**	**30**	**21**	**25**	**32**	**28**	**31**	**29**	**33**
Indonesia	1	5	4	3	2	9	23	8	7	6	12	11	14	18	13	17	21	10	16	25	26	22	19	15	24	30	20	27	32	28	31	29	33
Philippines	1	3	8	4	2	9	21	6	5	7	13	11	23	14	10	26	20	12	22	27	24	16	19	15	25	28	17	18	33	30	31	29	32
Vietnam	1	3	4	2	5	12	9	13	6	7	10	11	22	8	15	20	26	19	17	14	27	18	24	16	25	29	23	21	33	28	30	31	32

© 2019 Global Burden of Disease Cancer Collaboration. JAMA Oncol.

Country	Tracheal, bronchus, and lung cancer	Colon and rectum cancer	Stomach cancer	Liver cancer	Breast cancer	Pancreatic cancer	Esophageal cancer	Prostate cancer	Other malignant neoplasms	Cervical cancer	Non-Hodgkin lymphoma	Brain and central nervous system cancer	Bladder cancer	Lip and oral cavity cancer	Ovarian cancer	Gallbladder and biliary tract cancer	Kidney cancer	Other leukemia	Larynx cancer	Other pharynx cancer	Multiple myeloma	Acute myeloid leukemia	Uterine cancer	Nasopharynx cancer	Non-melanoma skin cancer	Malignant skin melanoma	Acute lymphoid leukemia	Thyroid cancer	Chronic lymphoid leukemia	Hodgkin lymphoma	Mesothelioma	Chronic myeloid leukemia	Testicular cancer
Thailand	2	3	6	1	4	7	11	9	10	8	16	13	17	12	15	5	23	14	19	24	25	20	26	18	21	30	22	27	32	31	29	28	33
Myanmar	1	3	5	4	2	11	6	9	8	7	12	14	15	16	13	18	21	10	20	25	24	23	19	17	27	30	22	26	32	28	29	31	33
Malaysia	1	2	5	4	3	11	12	9	6	7	10	15	16	14	13	22	20	23	19	24	25	18	21	8	27	29	17	26	31	30	32	28	33
Sri Lanka	1	3	7	8	2	9	6	10	4	16	12	15	21	5	17	14	11	13	25	18	24	20	26	23	22	30	19	27	32	28	29	31	33
Cambodia	1	2	4	5	3	11	13	8	7	6	10	12	16	15	14	19	23	9	18	24	26	22	20	17	27	30	21	25	32	28	31	29	33
Laos	1	3	5	2	4	12	13	9	7	6	10	11	15	16	14	19	23	8	18	24	26	22	21	17	25	29	20	27	32	28	31	30	33
Timor-Leste	1	4	3	2	6	12	14	5	7	8	10	11	13	16	15	18	24	9	19	27	26	21	22	17	23	29	20	25	32	28	31	30	33
Mauritius	1	3	4	7	2	6	12	5	8	9	15	17	14	11	10	20	18	13	19	24	21	23	16	22	26	29	27	25	32	28	31	30	33
Maldives	1	2	9	3	4	8	16	6	7	12	11	14	10	5	13	20	18	15	22	30	21	23	29	26	25	27	19	17	31	32	24	28	33
Seychelles	3	1	9	7	4	8	10	2	17	5	11	13	15	6	14	23	21	18	12	16	19	24	25	20	29	28	22	31	27	30	32	26	33
**North Africa and Middle East**	**1**	**3**	**2**	**5**	**4**	**8**	**13**	**9**	**6**	**16**	**12**	**7**	**11**	**22**	**14**	**18**	**20**	**10**	**15**	**30**	**19**	**17**	**24**	**25**	**29**	**27**	**21**	**26**	**32**	**23**	**28**	**31**	**33**
Egypt	2	6	9	1	3	10	14	12	5	20	11	8	4	21	13	16	17	7	18	26	19	23	22	31	28	29	24	25	32	15	27	30	33
Iran	2	3	1	10	4	9	7	5	8	19	13	6	12	23	15	18	17	11	14	31	20	16	25	32	26	24	21	28	29	27	22	30	33
Turkey	1	2	3	8	5	4	18	7	10	22	11	6	9	26	13	19	17	16	15	32	12	14	21	28	25	20	23	29	27	30	24	31	33
Iraq	1	4	8	7	2	10	20	11	5	18	12	3	9	22	14	23	16	6	13	26	21	15	19	30	27	31	17	24	32	25	29	28	33
Algeria	1	3	5	11	2	9	20	8	4	10	6	12	13	22	19	7	24	18	15	23	17	16	28	14	29	30	27	25	31	21	32	26	33
Sudan	2	3	1	10	4	13	7	9	5	17	12	8	11	21	19	18	20	6	14	30	23	15	29	25	28	26	16	24	31	22	32	27	33
Morocco	1	3	6	13	2	9	17	7	4	8	5	10	14	19	12	20	24	16	11	26	21	25	22	15	29	27	28	23	32	18	30	31	33
Saudi Arabia	2	1	7	3	4	9	12	11	6	24	5	10	14	15	17	13	16	8	23	27	18	20	26	19	29	30	25	21	31	22	33	28	32
Afghanistan	4	3	1	10	2	18	8	12	7	9	11	6	16	22	20	19	21	5	13	30	24	14	27	25	31	29	15	26	32	17	28	23	33
Yemen	2	3	1	10	4	15	8	9	5	14	12	7	11	21	19	18	22	6	13	30	23	16	28	26	29	27	17	25	31	20	32	24	33

© 2019 Global Burden of Disease Cancer Collaboration. JAMA Oncol.

Country	Tracheal, bronchus, and lung cancer	Colon and rectum cancer	Stomach cancer	Liver cancer	Breast cancer	Pancreatic cancer	Esophageal cancer	Prostate cancer	Other malignant neoplasms	Cervical cancer	Non-Hodgkin lymphoma	Brain and central nervous system cancer	Bladder cancer	Lip and oral cavity cancer	Ovarian cancer	Gallbladder and biliary tract cancer	Kidney cancer	Other leukemia	Larynx cancer	Other pharynx cancer	Multiple myeloma	Acute myeloid leukemia	Uterine cancer	Nasopharynx cancer	Non-melanoma skin cancer	Malignant skin melanoma	Acute lymphoid leukemia	Thyroid cancer	Chronic lymphoid leukemia	Hodgkin lymphoma	Mesothelioma	Chronic myeloid leukemia	Testicular cancer
Syria	2	3	7	8	4	9	16	6	10	17	12	5	13	22	15	21	18	1	19	27	20	11	25	30	26	29	14	28	23	32	31	24	33
Tunisia	1	2	4	14	3	9	20	8	5	18	7	13	6	19	15	12	21	10	11	25	16	24	23	17	27	28	29	26	31	22	32	30	33
Jordan	1	2	7	11	3	8	18	9	6	19	5	10	12	15	13	14	17	4	21	27	16	24	20	22	25	28	26	23	33	31	29	32	30
**United Arab Emirates**	**2**	**4**	**8**	**9**	**6**	**7**	**5**	**16**	**1**	**18**	**10**	**3**	**12**	**17**	**20**	**21**	**14**	**13**	**11**	**25**	**19**	**15**	**32**	**24**	**29**	**27**	**22**	**26**	**31**	**23**	**30**	**28**	**33**
Lebanon	1	3	8	13	2	9	21	11	5	17	4	10	6	22	12	15	18	7	16	28	14	23	20	27	29	26	25	24	31	19	33	30	32
Libya	1	2	8	9	3	6	20	12	4	16	7	5	11	23	15	14	21	10	13	28	19	22	24	17	27	30	25	26	32	18	29	31	33
Palestine	1	2	7	6	3	8	18	9	10	17	11	4	12	22	13	20	15	5	21	28	16	19	14	27	26	31	24	25	29	23	32	30	33
Oman	2	3	1	6	5	9	12	11	7	16	4	10	14	15	17	20	18	8	22	27	13	19	29	28	25	30	23	24	32	21	26	31	33
Kuwait	1	2	11	4	3	5	13	7	8	19	6	9	12	21	15	18	14	10	22	26	17	16	20	25	27	32	23	24	29	30	28	31	33
Qatar	1	4	7	3	2	8	13	10	6	22	9	5	15	20	14	21	16	12	19	24	18	11	23	30	25	31	17	26	28	29	32	27	33
Bahrain	1	3	5	7	2	4	17	9	6	18	8	10	11	19	12	21	14	13	22	28	16	15	20	26	29	32	23	25	30	24	27	31	33
**Western SSA**	**6**	**8**	**7**	**3**	**2**	**10**	**11**	**1**	**5**	**4**	**9**	**12**	**13**	**19**	**14**	**21**	**22**	**16**	**20**	**29**	**18**	**17**	**23**	**26**	**27**	**25**	**24**	**31**	**30**	**15**	**32**	**28**	**33**
Nigeria	6	5	9	10	2	8	11	1	3	4	7	13	21	22	14	19	20	15	18	31	17	16	25	24	27	23	26	30	29	12	32	28	33
Ghana	8	6	7	1	2	5	12	4	10	3	9	11	13	16	15	21	22	20	18	23	19	17	14	31	26	28	24	30	27	32	29	25	33
Cameroon	5	7	3	1	6	10	9	2	8	4	11	12	13	16	15	22	18	17	19	24	20	14	21	28	27	29	23	31	30	25	32	26	33
Cote d'Ivoire	5	9	7	3	2	10	19	1	8	6	4	16	11	14	12	18	23	20	17	25	13	15	21	22	26	29	27	30	31	24	32	28	33
Niger	5	8	3	1	7	11	9	4	6	2	10	13	15	16	17	19	22	12	20	28	21	14	18	27	26	29	23	31	30	24	32	25	33
Burkina Faso	7	5	4	1	3	12	9	6	8	2	11	10	14	19	16	20	22	13	17	26	21	15	18	28	27	29	23	31	30	24	32	25	33
Mali	8	7	2	1	4	11	12	9	3	5	10	16	6	15	21	20	19	13	22	30	17	14	24	27	28	23	25	26	31	18	33	29	32
Chad	5	8	3	1	6	11	9	4	7	2	10	13	14	16	19	18	21	12	17	26	22	15	20	28	27	29	23	31	30	24	32	25	33
Senegal	5	7	3	2	6	10	9	1	8	4	11	13	12	17	16	19	22	14	18	27	21	15	20	29	24	28	23	31	30	26	32	25	33
Guinea	5	8	3	1	4	12	13	6	7	2	11	17	10	9	14	18	23	21	16	19	25	20	15	26	27	22	29	30	31	24	33	28	32

 © 2019 Global Burden of Disease Cancer Collaboration. JAMA Oncol.

Country	Tracheal, bronchus, and lung cancer	Colon and rectum cancer	Stomach cancer	Liver cancer	Breast cancer	Pancreatic cancer	Esophageal cancer	Prostate cancer	Other malignant neoplasms	Cervical cancer	Non-Hodgkin lymphoma	Brain and central nervous system cancer	Bladder cancer	Lip and oral cavity cancer	Ovarian cancer	Gallbladder and biliary tract cancer	Kidney cancer	Other leukemia	Larynx cancer	Other pharynx cancer	Multiple myeloma	Acute myeloid leukemia	Uterine cancer	Nasopharynx cancer	Non-melanoma skin cancer	Malignant skin melanoma	Acute lymphoid leukemia	Thyroid cancer	Chronic lymphoid leukemia	Hodgkin lymphoma	Mesothelioma	Chronic myeloid leukemia	Testicular cancer
Benin	6	8	4	1	5	10	9	3	7	2	11	12	13	17	16	22	18	15	21	27	20	14	19	28	26	29	23	31	30	24	32	25	33
Sierra Leone	6	7	3	1	5	11	9	4	8	2	10	13	12	17	16	20	22	14	18	25	21	15	19	28	27	29	23	31	30	26	32	24	33
Togo	6	7	3	1	4	10	9	5	8	2	11	12	13	16	14	21	22	15	19	26	20	17	18	27	28	29	23	31	30	25	32	24	33
Liberia	7	6	4	1	5	10	8	3	9	2	11	13	12	17	16	22	19	14	21	27	18	15	20	28	26	29	23	31	30	25	32	24	33
Mauritania	6	7	5	2	4	10	9	1	8	3	11	13	12	16	14	21	20	17	22	27	18	15	19	30	24	26	23	31	28	29	32	25	33
The Gambia	3	6	8	1	4	10	11	9	5	2	7	18	12	14	15	16	20	17	22	28	21	13	19	26	24	31	25	30	29	23	32	27	33
Guinea-Bissau	5	6	3	1	4	11	8	7	9	2	10	12	13	17	16	22	20	14	19	26	21	15	18	27	28	29	23	30	32	24	31	25	33
Cape Verde	3	6	1	5	8	7	4	2	10	9	13	11	14	12	18	23	16	17	22	21	20	19	15	27	26	29	24	30	28	31	32	25	33
Sao Tome and Principe	3	5	2	13	6	12	9	7	1	4	10	19	8	22	11	15	20	17	21	24	18	16	14	30	29	28	23	26	27	32	31	25	33
**Western Europe**	**1**	**2**	**6**	**7**	**3**	**4**	**12**	**5**	**9**	**21**	**10**	**13**	**8**	**20**	**14**	**16**	**11**	**19**	**25**	**26**	**15**	**17**	**22**	**29**	**27**	**18**	**31**	**30**	**24**	**32**	**23**	**28**	**33**
Germany	1	2	6	7	3	4	14	5	8	21	11	13	9	19	12	15	10	23	25	20	16	17	24	30	27	18	31	29	22	32	26	28	33
United Kingdom	1	2	7	11	3	5	6	4	13	22	9	14	8	21	10	19	12	25	27	26	16	15	20	28	24	18	32	30	23	31	17	29	33
France	1	2	7	6	3	4	12	5	9	22	10	14	8	18	13	19	11	16	24	21	15	17	23	29	27	20	31	30	26	32	25	28	33
Italy	1	2	5	6	3	4	17	7	10	22	9	13	8	20	14	12	11	18	24	27	15	16	23	29	26	19	31	30	25	32	21	28	33
England	1	2	7	11	3	5	6	4	13	21	9	14	8	22	10	19	12	25	27	26	16	15	20	28	24	18	32	30	23	31	17	29	33
Spain	1	2	4	8	6	5	15	3	9	22	10	11	7	19	13	16	12	17	18	25	14	20	21	28	24	23	31	29	26	32	27	30	33
Netherlands	1	2	6	13	3	4	7	5	10	23	9	14	8	22	12	18	11	16	24	25	15	19	21	27	26	17	30	28	31	32	20	29	33
Belgium	1	2	6	9	3	5	11	4	8	21	10	14	7	18	13	20	12	16	25	24	15	17	22	29	27	19	31	30	26	32	23	28	33
Portugal	2	1	3	7	5	6	12	4	11	19	10	9	8	17	14	16	15	22	18	24	13	20	21	27	26	23	31	28	25	32	30	29	33
Greece	1	2	6	7	3	5	21	4	10	19	16	9	8	25	12	14	11	13	18	32	17	15	20	27	24	22	31	29	23	26	30	28	33
Sweden	1	2	7	10	4	5	16	3	11	19	8	13	6	22	14	17	9	23	29	25	15	18	20	31	26	12	30	27	21	32	24	28	33

© 2019 Global Burden of Disease Cancer Collaboration. JAMA Oncol.

Country	Tracheal, bronchus, and lung cancer	Colon and rectum cancer	Stomach cancer	Liver cancer	Breast cancer	Pancreatic cancer	Esophageal cancer	Prostate cancer	Other malignant neoplasms	Cervical cancer	Non-Hodgkin lymphoma	Brain and central nervous system cancer	Bladder cancer	Lip and oral cavity cancer	Ovarian cancer	Gallbladder and biliary tract cancer	Kidney cancer	Other leukemia	Larynx cancer	Other pharynx cancer	Multiple myeloma	Acute myeloid leukemia	Uterine cancer	Nasopharynx cancer	Non-melanoma skin cancer	Malignant skin melanoma	Acute lymphoid leukemia	Thyroid cancer	Chronic lymphoid leukemia	Hodgkin lymphoma	Mesothelioma	Chronic myeloid leukemia	Testicular cancer
Israel	1	2	5	11	3	4	18	6	10	20	7	8	9	24	12	21	13	23	25	32	14	15	19	31	22	16	27	26	17	30	28	29	33
Austria	1	2	6	7	4	3	17	5	8	20	9	13	10	19	12	14	11	24	26	22	15	16	23	30	25	18	31	29	21	32	27	28	33
Switzerland	1	2	7	6	4	5	11	3	10	21	8	13	9	20	12	17	14	19	27	25	15	18	24	29	26	16	32	28	23	31	22	30	33
Denmark	1	2	7	12	4	5	11	3	9	21	13	8	6	20	10	18	14	22	27	23	16	17	24	29	26	15	31	30	19	32	25	28	33
Finland	1	2	6	8	5	3	15	4	9	22	7	12	13	20	11	16	10	24	28	25	14	18	19	32	26	17	29	27	21	31	23	30	33
Scotland	1	2	7	9	3	5	6	4	11	22	10	14	8	20	12	19	13	25	27	26	15	16	21	28	24	17	30	31	23	32	18	29	33
Norway	1	2	6	15	5	4	16	3	11	21	9	10	7	22	13	18	8	19	28	26	12	17	20	31	25	14	29	27	23	32	24	30	33
Ireland	1	2	6	11	3	5	7	4	12	20	8	10	14	21	9	18	13	19	25	26	15	17	24	28	22	16	31	30	23	32	27	29	33
Wales	1	2	7	10	3	5	6	4	13	22	9	14	8	21	11	20	12	26	27	25	16	15	19	28	24	17	32	30	23	31	18	29	33
Northern Ireland	1	2	6	11	3	5	7	4	13	20	9	12	8	21	10	19	14	25	26	27	15	16	22	28	24	17	31	30	23	32	18	29	33
Cyprus	1	2	6	8	3	5	22	4	10	21	9	11	7	23	12	16	17	15	25	31	13	14	19	29	24	18	27	28	20	30	26	32	33
Luxembourg	1	2	6	7	3	4	13	5	10	22	12	8	9	20	11	21	18	16	25	23	14	15	19	29	27	17	31	28	24	32	26	30	33
Malta	1	2	5	13	3	4	15	6	10	23	8	11	7	18	9	19	12	27	22	29	16	14	17	24	25	20	32	28	26	31	21	30	33
Iceland	1	2	7	14	5	4	11	3	9	22	12	8	10	20	15	19	6	16	27	30	13	18	24	31	29	17	28	21	25	32	23	26	33
Andorra	1	2	6	13	4	5	12	3	9	22	8	10	7	20	11	19	14	23	26	27	15	16	24	29	25	18	31	30	21	32	17	28	33
**Eastern SSA**	**9**	**4**	**10**	**5**	**3**	**12**	**6**	**7**	**1**	**2**	**8**	**11**	**15**	**14**	**13**	**22**	**26**	**17**	**21**	**29**	**20**	**16**	**24**	**23**	**25**	**30**	**18**	**27**	**31**	**19**	**32**	**28**	**33**
Ethiopia	4	1	7	11	2	13	10	9	5	3	6	8	19	14	12	21	26	23	27	30	24	17	29	25	22	31	15	20	28	18	32	16	33
Tanzania	7	5	9	8	4	12	10	3	1	2	6	11	14	20	13	26	25	15	19	28	17	16	21	24	22	27	18	29	31	23	32	30	33
Kenya	9	5	2	8	3	11	4	7	1	6	10	12	24	13	14	18	27	21	16	22	15	19	26	17	20	28	23	30	29	25	32	31	33
Uganda	9	8	10	7	5	11	3	2	1	6	4	13	17	12	14	29	27	22	23	20	15	21	18	16	26	24	25	28	31	19	33	30	32
Mozambique	5	3	7	1	4	14	6	11	8	2	10	9	19	16	13	24	26	12	18	28	21	15	22	32	23	25	17	27	30	20	31	29	33
Madagascar	8	4	6	10	3	13	5	7	2	1	9	11	16	14	12	26	28	15	18	27	22	17	20	21	24	25	23	29	31	19	32	30	33

© 2019 Global Burden of Disease Cancer Collaboration. JAMA Oncol.

Country	Tracheal, bronchus, and lung cancer	Colon and rectum cancer	Stomach cancer	Liver cancer	Breast cancer	Pancreatic cancer	Esophageal cancer	Prostate cancer	Other malignant neoplasms	Cervical cancer	Non-Hodgkin lymphoma	Brain and central nervous system cancer	Bladder cancer	Lip and oral cavity cancer	Ovarian cancer	Gallbladder and biliary tract cancer	Kidney cancer	Other leukemia	Larynx cancer	Other pharynx cancer	Multiple myeloma	Acute myeloid leukemia	Uterine cancer	Nasopharynx cancer	Non-melanoma skin cancer	Malignant skin melanoma	Acute lymphoid leukemia	Thyroid cancer	Chronic lymphoid leukemia	Hodgkin lymphoma	Mesothelioma	Chronic myeloid leukemia	Testicular cancer
Zambia	8	4	9	5	3	12	10	6	1	2	7	11	14	17	13	27	25	16	19	26	18	15	21	23	24	28	22	29	31	20	32	30	33
Malawi	10	9	11	7	5	12	1	8	2	4	3	15	6	13	16	23	21	20	25	32	17	19	22	28	18	14	26	27	31	24	33	29	30
Somalia	10	3	7	8	5	13	4	6	2	1	9	11	17	16	12	25	26	14	20	28	23	18	21	22	24	27	19	29	31	15	32	30	33
Rwanda	6	5	10	8	3	11	7	4	2	1	9	13	17	14	12	25	28	18	19	26	16	15	20	23	24	27	21	29	31	22	32	30	33
Burundi	9	7	5	10	4	13	3	6	2	1	8	11	17	12	14	26	27	15	16	24	20	19	22	21	25	28	23	29	31	18	32	30	33
South Sudan	5	4	10	7	8	12	3	6	2	1	9	11	16	15	13	26	24	14	18	27	20	17	23	21	25	28	22	29	31	19	32	30	33
Eritrea	10	4	7	8	3	13	6	9	2	1	5	11	17	14	12	26	28	16	20	27	24	15	19	21	25	23	22	29	31	18	32	30	33
Djibouti	6	3	10	8	4	11	7	5	2	1	9	13	14	15	12	27	24	21	18	26	17	16	19	20	22	28	25	29	31	23	32	30	33
Comoros	7	4	9	8	2	11	6	5	3	1	10	13	14	15	12	24	27	20	19	28	16	18	17	22	21	25	26	29	31	23	32	30	33
**High-income North America**	**1**	**2**	**8**	**6**	**3**	**4**	**10**	**5**	**12**	**20**	**7**	**11**	**9**	**21**	**14**	**23**	**13**	**19**	**24**	**26**	**15**	**16**	**18**	**32**	**25**	**17**	**29**	**28**	**22**	**31**	**27**	**30**	**33**
United States	1	2	12	6	3	4	9	5	10	20	7	11	8	21	14	24	13	19	23	26	15	16	18	32	25	17	29	28	22	31	27	30	33
Canada	1	2	6	9	3	4	10	5	12	20	7	11	8	21	14	16	13	19	26	27	15	17	22	32	25	18	30	28	23	31	24	29	33
**Central Latin America**	**1**	**3**	**2**	**6**	**5**	**8**	**16**	**4**	**9**	**7**	**10**	**11**	**18**	**24**	**12**	**13**	**14**	**19**	**23**	**28**	**17**	**20**	**22**	**32**	**21**	**26**	**15**	**25**	**31**	**27**	**33**	**29**	**30**
Mexico	1	2	3	6	4	7	16	5	9	8	10	13	17	23	12	14	11	21	22	31	18	19	24	33	20	26	15	25	32	27	29	30	28
Colombia	2	3	1	6	5	7	14	4	9	8	10	11	17	23	13	12	18	20	22	30	16	21	24	31	19	25	15	26	28	29	33	27	32
Venezuela	1	4	3	7	5	8	15	2	9	6	10	13	18	23	12	16	11	24	14	25	19	22	21	30	17	28	20	26	31	27	33	29	32
Guatemala	5	6	1	2	7	9	15	3	8	4	13	12	21	20	18	14	19	10	23	26	24	22	16	27	17	29	11	25	32	28	33	31	30
Honduras	1	3	2	14	4	8	19	5	6	15	16	17	21	20	12	10	23	11	26	22	18	13	7	28	24	30	9	27	29	32	31	25	33
Nicaragua	5	4	1	2	6	9	19	7	8	3	11	12	17	22	14	10	16	15	18	27	24	20	23	31	21	29	13	25	30	28	33	26	32
El Salvador	2	3	1	6	7	8	16	5	10	4	13	12	17	19	14	11	18	9	23	25	21	24	15	26	22	31	20	27	30	28	32	29	33
Costa Rica	4	2	1	6	5	7	17	3	9	10	8	11	14	23	15	13	18	25	22	28	12	19	20	30	21	24	16	31	29	27	33	26	32

© 2019 Global Burden of Disease Cancer Collaboration. JAMA Oncol.

Country	Tracheal, bronchus, and lung cancer	Colon and rectum cancer	Stomach cancer	Liver cancer	Breast cancer	Pancreatic cancer	Esophageal cancer	Prostate cancer	Other malignant neoplasms	Cervical cancer	Non-Hodgkin lymphoma	Brain and central nervous system cancer	Bladder cancer	Lip and oral cavity cancer	Ovarian cancer	Gallbladder and biliary tract cancer	Kidney cancer	Other leukemia	Larynx cancer	Other pharynx cancer	Multiple myeloma	Acute myeloid leukemia	Uterine cancer	Nasopharynx cancer	Non-melanoma skin cancer	Malignant skin melanoma	Acute lymphoid leukemia	Thyroid cancer	Chronic lymphoid leukemia	Hodgkin lymphoma	Mesothelioma	Chronic myeloid leukemia	Testicular cancer
Panama	1	3	4	7	5	9	16	2	8	6	11	10	19	21	12	14	13	23	22	24	17	20	15	28	25	27	18	26	30	31	32	29	33
**Tropical Latin America**	**1**	**2**	**3**	**7**	**5**	**6**	**9**	**4**	**8**	**11**	**12**	**10**	**16**	**13**	**17**	**14**	**18**	**23**	**15**	**19**	**20**	**21**	**24**	**32**	**22**	**25**	**26**	**27**	**29**	**30**	**28**	**31**	**33**
Brazil	1	2	3	7	5	6	8	4	9	11	12	10	16	13	17	14	18	23	15	19	20	21	24	32	22	25	26	27	29	30	28	31	33
Paraguay	1	2	6	10	4	8	9	3	7	5	11	15	21	16	12	13	17	14	19	24	25	20	18	33	22	27	23	26	29	28	32	30	31
**Eastern Europe**	**1**	**2**	**3**	**8**	**4**	**6**	**13**	**7**	**5**	**15**	**16**	**12**	**11**	**14**	**10**	**22**	**9**	**23**	**18**	**20**	**21**	**24**	**17**	**31**	**25**	**19**	**27**	**28**	**26**	**29**	**32**	**30**	**33**
Russian Federation	1	2	3	7	4	6	11	8	5	15	14	13	12	16	10	21	9	23	18	22	20	26	17	32	24	19	27	28	25	29	31	30	33
Ukraine	1	2	3	10	4	5	14	6	7	15	17	9	12	13	11	21	8	22	16	19	24	23	18	30	25	20	27	29	26	28	32	31	33
Belarus	1	2	3	10	4	5	14	7	6	11	16	12	15	13	9	20	8	25	17	19	21	22	23	31	24	18	27	28	26	29	32	30	33
Moldova	1	2	5	4	3	6	18	8	7	9	17	10	12	14	16	24	15	21	11	13	23	25	19	26	22	20	28	29	30	27	32	33	31
Lithuania	1	2	3	13	4	6	12	5	10	14	15	11	9	16	7	22	8	26	18	23	20	19	17	31	25	21	29	27	24	30	32	28	33
Latvia	1	2	3	12	4	5	13	6	9	17	15	11	7	16	8	24	10	26	19	23	20	25	14	32	22	18	29	27	21	30	31	28	33
Estonia	1	2	3	11	6	4	14	5	8	15	13	12	9	18	10	20	7	25	22	24	17	19	21	31	26	16	28	27	23	30	32	29	33
**High-income Asia Pacific**	**1**	**2**	**3**	**4**	**7**	**5**	**10**	**8**	**11**	**17**	**9**	**19**	**12**	**18**	**14**	**6**	**13**	**20**	**26**	**22**	**15**	**16**	**21**	**27**	**24**	**29**	**28**	**23**	**31**	**32**	**25**	**30**	**33**
Japan	1	2	3	5	7	4	9	8	11	18	10	19	12	17	14	6	13	21	26	22	15	16	20	27	25	29	28	23	31	32	24	30	33
South Korea	1	4	3	2	7	5	11	8	9	17	10	15	12	20	16	6	13	14	22	23	18	21	25	26	24	27	28	19	32	31	29	30	33
Singapore	1	2	5	3	4	6	14	7	9	16	10	13	17	21	11	18	15	24	22	23	20	12	19	8	26	29	25	28	32	31	27	30	33
Brunei	1	2	5	4	3	9	20	12	7	8	6	11	19	14	10	16	17	26	27	21	18	13	22	15	30	28	24	25	33	29	31	23	32
**Central SSA**	**3**	**4**	**7**	**5**	**2**	**10**	**6**	**9**	**8**	**1**	**12**	**11**	**14**	**15**	**16**	**19**	**22**	**13**	**18**	**29**	**21**	**17**	**20**	**28**	**23**	**26**	**25**	**30**	**32**	**24**	**31**	**27**	**33**
DRC	3	5	6	4	2	10	7	9	8	1	12	11	14	15	16	19	23	13	18	29	22	17	20	28	21	26	25	30	31	24	33	27	32
Angola	2	4	8	6	3	10	5	9	7	1	11	12	13	15	17	21	19	14	18	27	20	16	22	29	23	26	24	30	32	25	31	28	33
Congo	3	4	9	6	2	10	5	7	8	1	13	12	11	15	14	21	20	17	18	26	22	16	19	25	23	24	29	30	32	27	31	28	33

 © 2019 Global Burden of Disease Cancer Collaboration. JAMA Oncol.

Country	Tracheal, bronchus, and lung cancer	Colon and rectum cancer	Stomach cancer	Liver cancer	Breast cancer	Pancreatic cancer	Esophageal cancer	Prostate cancer	Other malignant neoplasms	Cervical cancer	Non-Hodgkin lymphoma	Brain and central nervous system cancer	Bladder cancer	Lip and oral cavity cancer	Ovarian cancer	Gallbladder and biliary tract cancer	Kidney cancer	Other leukemia	Larynx cancer	Other pharynx cancer	Multiple myeloma	Acute myeloid leukemia	Uterine cancer	Nasopharynx cancer	Non-melanoma skin cancer	Malignant skin melanoma	Acute lymphoid leukemia	Thyroid cancer	Chronic lymphoid leukemia	Hodgkin lymphoma	Mesothelioma	Chronic myeloid leukemia	Testicular cancer
CAR	3	6	4	7	2	10	5	9	8	1	12	11	16	13	17	20	22	14	15	29	23	18	19	26	24	28	27	30	33	21	31	25	32
Gabon	1	3	9	6	2	10	5	7	8	4	12	13	11	14	15	21	19	20	16	24	18	17	23	26	22	25	28	30	32	27	31	29	33
Equatorial Guinea	3	6	10	5	1	9	4	7	8	2	11	12	15	14	13	20	17	19	21	25	18	16	22	27	23	26	24	29	31	28	32	30	33
**Central Europe**	**1**	**2**	**5**	**7**	**3**	**4**	**16**	**6**	**9**	**13**	**15**	**10**	**8**	**17**	**12**	**14**	**11**	**26**	**18**	**21**	**22**	**23**	**19**	**30**	**24**	**20**	**28**	**27**	**25**	**29**	**32**	**31**	**33**
Poland	1	2	4	13	3	5	15	6	11	14	16	9	7	17	10	12	8	26	18	24	20	22	19	32	25	21	29	27	23	30	28	31	33
Romania	1	2	3	5	4	6	17	7	8	9	18	11	10	13	12	19	15	23	14	16	25	22	21	27	20	24	28	29	26	30	31	32	33
Czech Republic	1	2	6	9	4	3	15	5	11	16	14	13	10	18	12	8	7	22	25	23	17	21	20	32	26	19	29	27	24	28	31	30	33
Hungary	1	2	5	8	3	4	13	6	14	19	16	15	7	10	12	11	9	25	18	17	22	21	23	27	26	20	30	28	24	31	32	29	33
Serbia	1	2	6	7	3	4	18	5	10	11	15	8	9	19	12	14	13	17	16	23	24	25	20	30	22	21	29	27	26	28	32	31	33
Bulgaria	1	2	4	7	3	5	18	6	10	12	16	8	9	17	11	19	14	23	13	24	25	21	15	31	22	20	28	29	26	27	33	30	32
Slovakia	1	2	5	8	3	4	16	6	7	18	14	10	12	15	13	9	11	26	22	17	20	23	19	30	25	21	27	28	24	29	33	32	31
Croatia	1	2	5	7	3	6	15	4	10	20	12	8	9	16	13	14	11	26	18	23	19	22	21	32	25	17	30	28	24	31	27	29	33
Bosnia and Herzegovina	1	2	3	6	4	5	16	7	9	14	17	8	10	18	11	12	13	28	15	23	24	20	19	31	25	21	26	27	22	30	33	29	32
Albania	1	6	2	5	8	7	12	3	4	14	13	9	20	16	17	18	11	23	10	25	22	15	21	30	19	26	27	28	24	29	33	31	32
Macedonia	1	2	3	7	4	6	19	5	9	14	17	8	10	21	12	15	18	24	11	26	25	20	16	32	22	13	27	29	23	28	33	30	31
Slovenia	1	2	6	7	5	4	15	3	10	20	9	14	8	21	13	11	12	27	24	18	16	19	22	32	25	17	29	30	23	31	28	26	33
Montenegro	1	2	6	7	3	4	13	5	16	12	18	8	10	17	11	15	14	30	9	25	24	19	22	33	23	21	27	28	20	26	32	29	31
**Central Asia**	**1**	**5**	**2**	**3**	**4**	**8**	**6**	**11**	**7**	**10**	**14**	**9**	**15**	**16**	**13**	**21**	**12**	**19**	**17**	**22**	**25**	**20**	**18**	**28**	**24**	**26**	**23**	**29**	**30**	**27**	**32**	**31**	**33**
Uzbekistan	2	4	1	5	3	10	7	11	8	9	12	6	18	13	16	26	15	19	14	21	25	17	20	27	23	28	22	31	29	24	33	30	32
Kazakhstan	1	3	2	6	4	7	5	11	8	9	18	13	15	14	10	20	12	24	17	22	25	19	16	29	26	21	23	27	30	28	32	31	33
Azerbaijan	1	3	2	5	6	8	4	10	7	12	17	9	15	19	16	18	11	13	14	26	23	21	20	30	22	27	24	28	31	25	32	29	33

© 2019 Global Burden of Disease Cancer Collaboration. JAMA Oncol.

Country	Tracheal, bronchus, and lung cancer	Colon and rectum cancer	Stomach cancer	Liver cancer	Breast cancer	Pancreatic cancer	Esophageal cancer	Prostate cancer	Other malignant neoplasms	Cervical cancer	Non-Hodgkin lymphoma	Brain and central nervous system cancer	Bladder cancer	Lip and oral cavity cancer	Ovarian cancer	Gallbladder and biliary tract cancer	Kidney cancer	Other leukemia	Larynx cancer	Other pharynx cancer	Multiple myeloma	Acute myeloid leukemia	Uterine cancer	Nasopharynx cancer	Non-melanoma skin cancer	Malignant skin melanoma	Acute lymphoid leukemia	Thyroid cancer	Chronic lymphoid leukemia	Hodgkin lymphoma	Mesothelioma	Chronic myeloid leukemia	Testicular cancer
Tajikistan	3	5	1	7	6	9	8	11	4	13	10	2	18	20	15	24	12	14	21	25	22	17	16	23	26	27	19	32	31	28	29	30	33
Kyrgyzstan	2	4	1	3	5	8	9	12	7	6	15	10	16	14	11	20	13	18	22	24	26	19	17	28	21	25	23	27	31	29	33	30	32
Turkmenistan	1	5	2	6	4	10	3	12	7	8	15	9	17	13	14	21	11	16	22	18	20	19	27	28	25	26	23	29	32	24	33	31	30
Georgia	1	4	2	5	3	7	15	8	6	9	17	10	11	16	12	20	18	19	13	23	26	24	14	32	21	22	29	28	31	25	30	33	27
Mongolia	3	7	2	1	8	9	4	15	5	6	11	10	20	14	12	13	17	22	19	25	23	16	18	26	27	31	21	24	32	29	30	28	33
Armenia	1	2	3	5	4	6	16	8	7	11	18	9	10	20	12	19	13	17	14	29	22	23	15	31	21	27	26	24	28	32	25	30	33
**Southern SSA**	**1**	**6**	**8**	**7**	**5**	**9**	**4**	**2**	**10**	**3**	**11**	**17**	**13**	**12**	**14**	**24**	**23**	**15**	**19**	**26**	**18**	**20**	**21**	**27**	**16**	**22**	**30**	**28**	**31**	**29**	**25**	**33**	**32**
South Africa	1	6	9	7	5	8	3	2	10	4	14	17	16	12	13	23	21	11	19	26	18	20	22	27	15	24	31	28	30	29	25	33	32
Zimbabwe	8	9	7	2	6	11	5	3	4	1	10	15	12	21	13	23	27	16	17	28	14	19	18	26	20	22	29	25	31	24	32	33	30
Namibia	6	5	11	10	1	9	12	3	2	4	8	16	19	7	17	25	24	18	14	20	21	22	23	28	15	13	31	29	32	27	26	33	30
Botswana	1	5	10	7	3	9	6	2	8	4	11	16	15	13	12	24	23	14	20	25	18	19	21	27	17	22	30	28	31	29	26	32	33
Lesotho	2	8	7	5	4	10	3	6	9	1	12	16	17	11	14	23	25	13	15	26	20	21	19	27	18	22	31	29	33	28	24	32	30
Swaziland	2	6	9	3	5	10	1	7	8	4	12	15	17	11	13	24	22	14	16	25	20	19	21	26	18	23	30	29	32	28	27	33	31
**Southern Latin America**	**1**	**2**	**3**	**10**	**4**	**6**	**11**	**5**	**9**	**8**	**13**	**15**	**14**	**21**	**16**	**7**	**12**	**17**	**20**	**27**	**18**	**22**	**19**	**33**	**23**	**24**	**25**	**26**	**28**	**29**	**31**	**32**	**30**
Argentina	1	2	4	12	3	5	10	6	9	7	13	15	14	21	16	8	11	17	18	29	20	22	19	33	24	23	25	26	30	27	28	32	31
Chile	2	3	1	8	6	7	12	4	9	10	13	16	14	23	17	5	11	19	24	28	15	21	20	33	18	22	25	26	29	31	32	30	27
Uruguay	1	2	4	16	3	6	8	5	13	9	11	15	12	21	14	7	10	18	19	26	17	23	20	31	25	22	30	27	24	28	32	29	33
**Andean Latin America**	**2**	**4**	**1**	**5**	**7**	**9**	**18**	**3**	**8**	**6**	**10**	**12**	**20**	**22**	**14**	**11**	**15**	**13**	**26**	**27**	**17**	**21**	**16**	**33**	**23**	**25**	**19**	**24**	**32**	**28**	**31**	**29**	**30**
Peru	2	4	1	5	6	8	19	3	9	7	10	12	18	22	15	11	14	13	26	27	16	20	17	33	23	25	21	24	32	28	31	29	30
Ecuador	3	4	1	5	7	9	20	2	8	6	10	11	23	24	13	12	17	15	26	27	18	19	14	33	21	25	16	22	32	28	31	29	30
Bolivia	3	5	1	6	7	10	16	2	8	4	9	12	17	22	15	11	18	13	24	27	20	21	14	31	26	25	19	23	33	28	29	30	32
**Caribbean**	**1**	**3**	**5**	**7**	**4**	**9**	**10**	**2**	**8**	**6**	**11**	**14**	**13**	**16**	**19**	**22**	**20**	**17**	**12**	**24**	**18**	**23**	**15**	**25**	**21**	**27**	**26**	**28**	**31**	**29**	**32**	**30**	**33**

 © 2019 Global Burden of Disease Cancer Collaboration. JAMA Oncol.

Country	Tracheal, bronchus, and lung cancer	Colon and rectum cancer	Stomach cancer	Liver cancer	Breast cancer	Pancreatic cancer	Esophageal cancer	Prostate cancer	Other malignant neoplasms	Cervical cancer	Non-Hodgkin lymphoma	Brain and central nervous system cancer	Bladder cancer	Lip and oral cavity cancer	Ovarian cancer	Gallbladder and biliary tract cancer	Kidney cancer	Other leukemia	Larynx cancer	Other pharynx cancer	Multiple myeloma	Acute myeloid leukemia	Uterine cancer	Nasopharynx cancer	Non-melanoma skin cancer	Malignant skin melanoma	Acute lymphoid leukemia	Thyroid cancer	Chronic lymphoid leukemia	Hodgkin lymphoma	Mesothelioma	Chronic myeloid leukemia	Testicular cancer
Haiti	5	6	3	8	4	12	11	1	7	2	9	15	16	17	18	19	21	10	13	25	20	22	14	29	26	27	24	30	31	23	32	28	33
Cuba	1	3	5	8	4	6	9	2	10	12	13	14	11	16	19	22	20	24	7	21	17	23	15	30	18	27	31	28	26	29	32	25	33
Dominican Republic	2	3	6	5	4	9	14	1	8	7	13	11	21	12	17	22	23	10	15	18	16	24	20	25	19	28	26	27	30	29	32	31	33
Jamaica	2	3	5	10	4	9	13	1	7	6	8	18	16	20	15	19	21	11	17	23	14	22	12	27	25	26	29	24	30	31	32	28	33
Trinidad and Tobago	4	2	7	9	3	6	17	1	8	5	11	18	16	19	10	21	14	15	22	23	13	20	12	29	26	28	24	25	31	30	32	27	33
Guyana	5	4	6	7	2	9	14	1	8	3	12	17	19	16	10	18	15	13	20	26	21	29	11	32	24	27	22	25	31	28	30	23	33
Suriname	1	3	7	6	4	9	18	2	8	5	11	10	15	17	12	20	16	13	21	27	14	23	19	22	25	24	29	26	31	28	32	30	33
Belize	2	6	5	4	7	8	12	1	9	3	13	11	17	18	19	20	16	10	15	24	21	30	14	28	22	27	23	29	31	25	26	32	33
The Bahamas	4	3	5	8	2	10	9	1	7	6	12	17	20	14	13	19	18	23	16	22	11	21	15	29	25	24	30	28	26	31	32	27	33
Barbados	5	2	4	10	3	7	12	1	8	6	9	18	15	19	14	22	17	16	21	23	11	20	13	27	24	26	31	25	28	30	32	29	33
Saint Lucia	3	5	4	10	2	7	11	1	8	6	9	19	14	15	13	21	20	18	17	22	12	23	16	26	28	24	30	27	25	31	32	29	33
Saint Vincent and the Grenadines	4	3	5	9	2	10	16	1	8	6	7	19	17	11	15	23	20	14	13	21	18	24	12	28	22	27	31	26	25	30	32	29	33
Grenada	4	2	5	10	3	6	11	1	9	7	8	18	15	17	13	20	22	16	19	24	14	28	12	29	23	27	30	25	21	32	31	26	33
Antigua and Barbuda	5	3	4	6	2	8	12	1	9	7	11	16	15	20	10	21	19	13	18	25	14	22	17	27	23	24	29	26	31	30	32	28	33
Dominica	3	5	2	8	4	9	11	1	7	6	10	22	13	15	21	19	18	14	16	20	12	24	17	29	25	26	23	27	30	31	32	28	33
**Australasia**	**1**	**2**	**7**	**9**	**4**	**5**	**10**	**3**	**12**	**23**	**6**	**11**	**14**	**21**	**16**	**20**	**13**	**24**	**28**	**26**	**15**	**17**	**22**	**29**	**18**	**8**	**27**	**31**	**25**	**32**	**19**	**30**	**33**
Australia	1	2	7	8	4	5	10	3	12	24	6	11	14	21	16	20	13	23	28	25	15	17	22	29	19	9	27	31	26	32	18	30	33
New Zealand	1	2	7	9	3	5	11	4	10	24	6	12	14	23	16	19	13	25	28	26	15	17	20	29	18	8	27	30	21	32	22	31	33
**Oceania**	**1**	**6**	**2**	**5**	**3**	**12**	**17**	**8**	**7**	**4**	**11**	**13**	**20**	**14**	**16**	**22**	**25**	**9**	**19**	**21**	**23**	**18**	**10**	**15**	**24**	**27**	**28**	**26**	**32**	**29**	**33**	**30**	**31**
Papua New Guinea	1	6	2	5	4	14	17	8	7	3	11	12	21	15	16	22	25	9	18	20	24	19	10	13	23	27	26	30	32	28	33	29	31

© 2019 Global Burden of Disease Cancer Collaboration. JAMA Oncol.

Country	Tracheal, bronchus, and lung cancer	Colon and rectum cancer	Stomach cancer	Liver cancer	Breast cancer	Pancreatic cancer	Esophageal cancer	Prostate cancer	Other malignant neoplasms	Cervical cancer	Non-Hodgkin lymphoma	Brain and central nervous system cancer	Bladder cancer	Lip and oral cavity cancer	Ovarian cancer	Gallbladder and biliary tract cancer	Kidney cancer	Other leukemia	Larynx cancer	Other pharynx cancer	Multiple myeloma	Acute myeloid leukemia	Uterine cancer	Nasopharynx cancer	Non-melanoma skin cancer	Malignant skin melanoma	Acute lymphoid leukemia	Thyroid cancer	Chronic lymphoid leukemia	Hodgkin lymphoma	Mesothelioma	Chronic myeloid leukemia	Testicular cancer
Fiji	5	4	8	3	1	9	14	6	7	2	13	15	16	12	23	21	24	17	22	20	18	11	10	26	25	29	27	19	30	32	31	33	28
Solomon Islands	1	6	2	4	3	11	15	8	7	5	12	13	20	17	16	22	25	10	19	21	23	18	9	14	24	28	27	26	31	30	32	29	33
Vanuatu	1	6	5	2	3	10	20	4	7	8	11	15	17	13	18	21	25	9	19	24	26	14	12	16	22	23	27	28	31	29	32	30	33
Samoa	3	2	1	8	4	10	15	7	5	6	9	14	18	20	13	23	25	11	26	32	17	21	12	19	22	16	28	24	29	27	33	31	30
Kiribati	2	6	5	4	3	12	9	11	8	1	14	16	21	7	22	20	19	13	23	17	24	18	10	25	30	28	27	31	29	32	33	26	15
Federated States of Micronesia	1	5	4	3	2	9	16	8	7	6	11	15	18	14	12	24	23	13	20	22	21	19	10	17	25	27	28	26	30	32	31	29	33
Tonga	1	7	4	2	3	10	13	5	11	6	9	16	18	17	15	25	22	19	24	26	20	21	12	23	14	27	29	28	30	31	33	32	8
Marshall Islands	1	5	4	3	2	9	13	8	7	6	11	15	20	17	14	24	23	12	18	22	21	19	10	16	25	27	28	26	31	30	32	29	33

Colors correspond to the ranking, with dark red as the most common cancer and dark green as the least common cancer for the location indicated. Rankings do not include the “other malignant cancer” group. The numbers inside each box indicate the ranking. Abbreviations: SSA: Sub-Saharan Africa; DRC: Democratic Republic of Congo; CAR: Central African Republic

eFigure 6: Cancer ranking by total mortality based on global level for developing and developed regions and all countries, both sexes, 2017

© 2019 Global Burden of Disease Cancer Collaboration. JAMA Oncol.

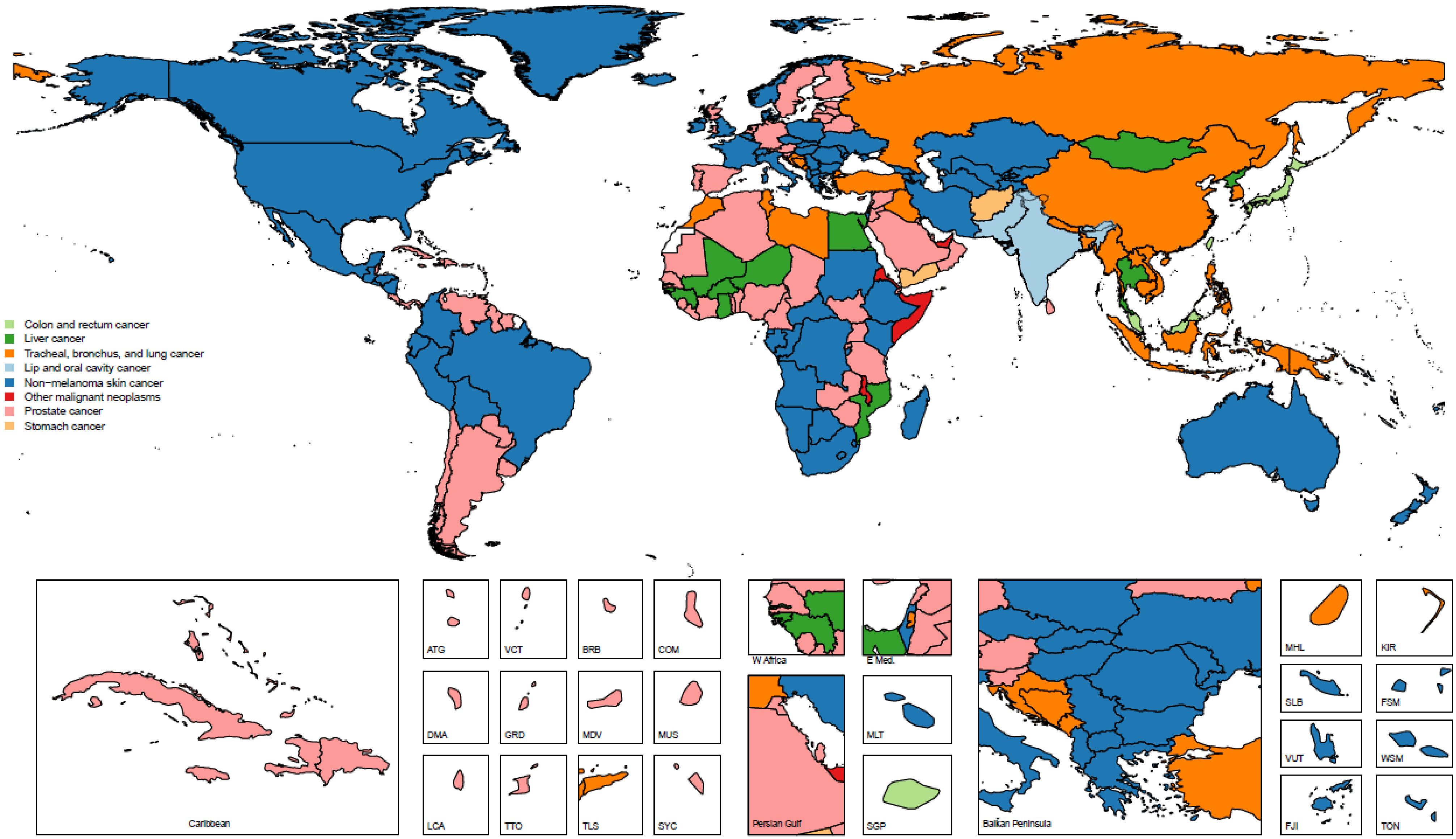

eFigure 7: Top-ranked cancers by absolute incident cases for all ages in males, 2017

© 2019 Global Burden of Disease Cancer Collaboration. JAMA Oncol.

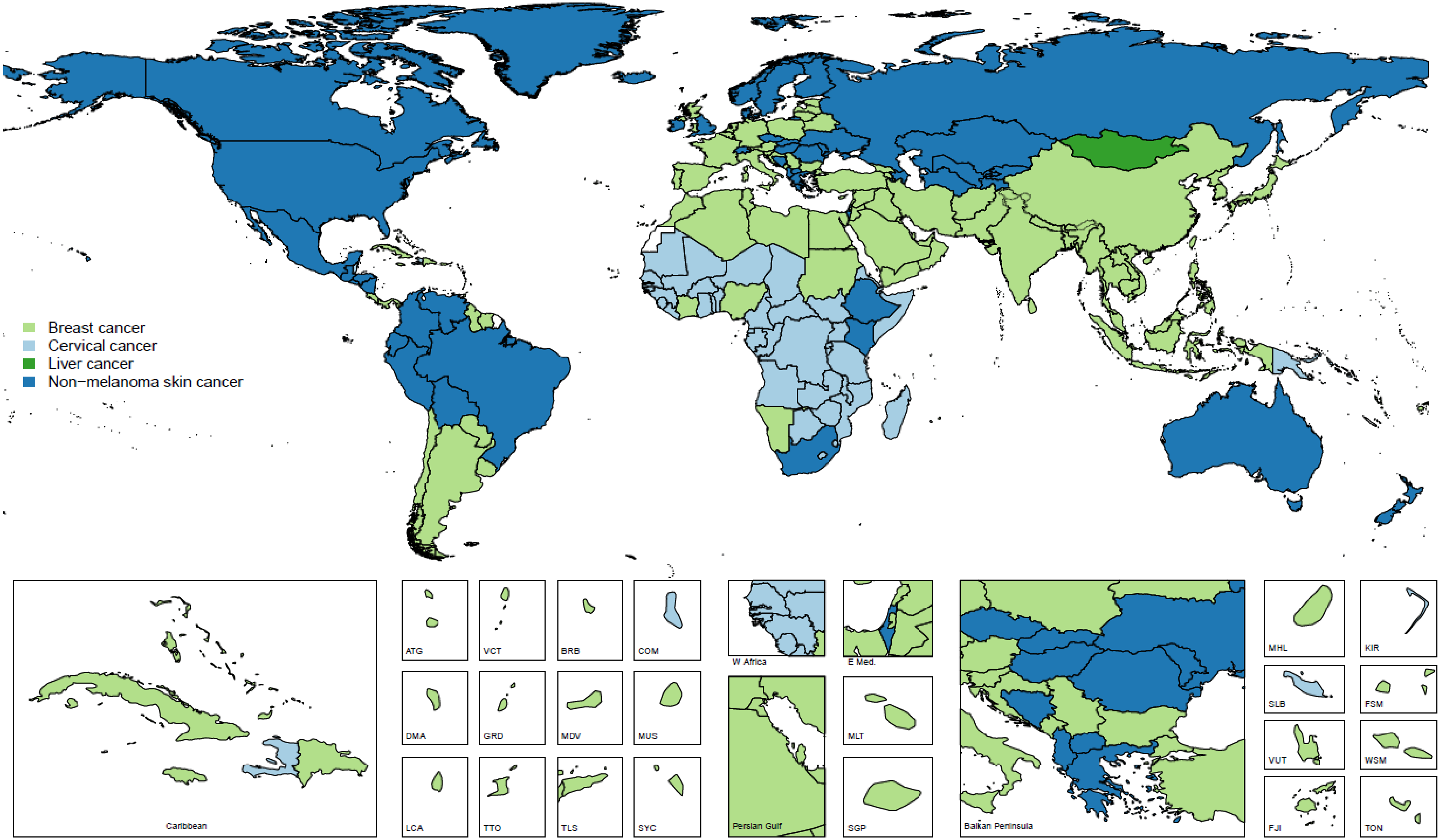

eFigure 8: Top-ranked cancers by absolute incident cases for all ages in females, 2017

© 2019 Global Burden of Disease Cancer Collaboration. JAMA Oncol.

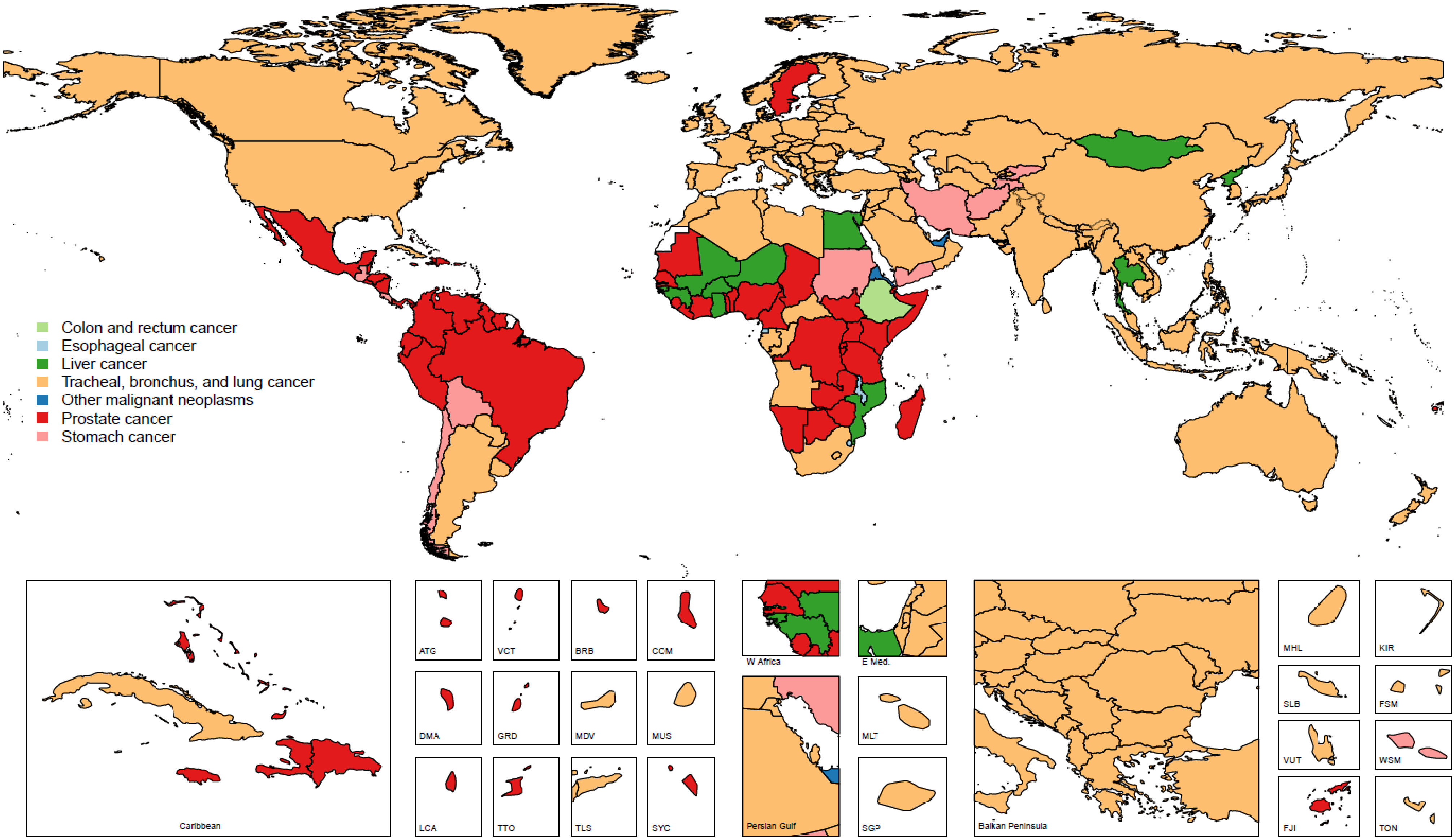

eFigure 9: Top-ranked cancers by absolute deaths for all ages in males, 2017

© 2019 Global Burden of Disease Cancer Collaboration. JAMA Oncol.

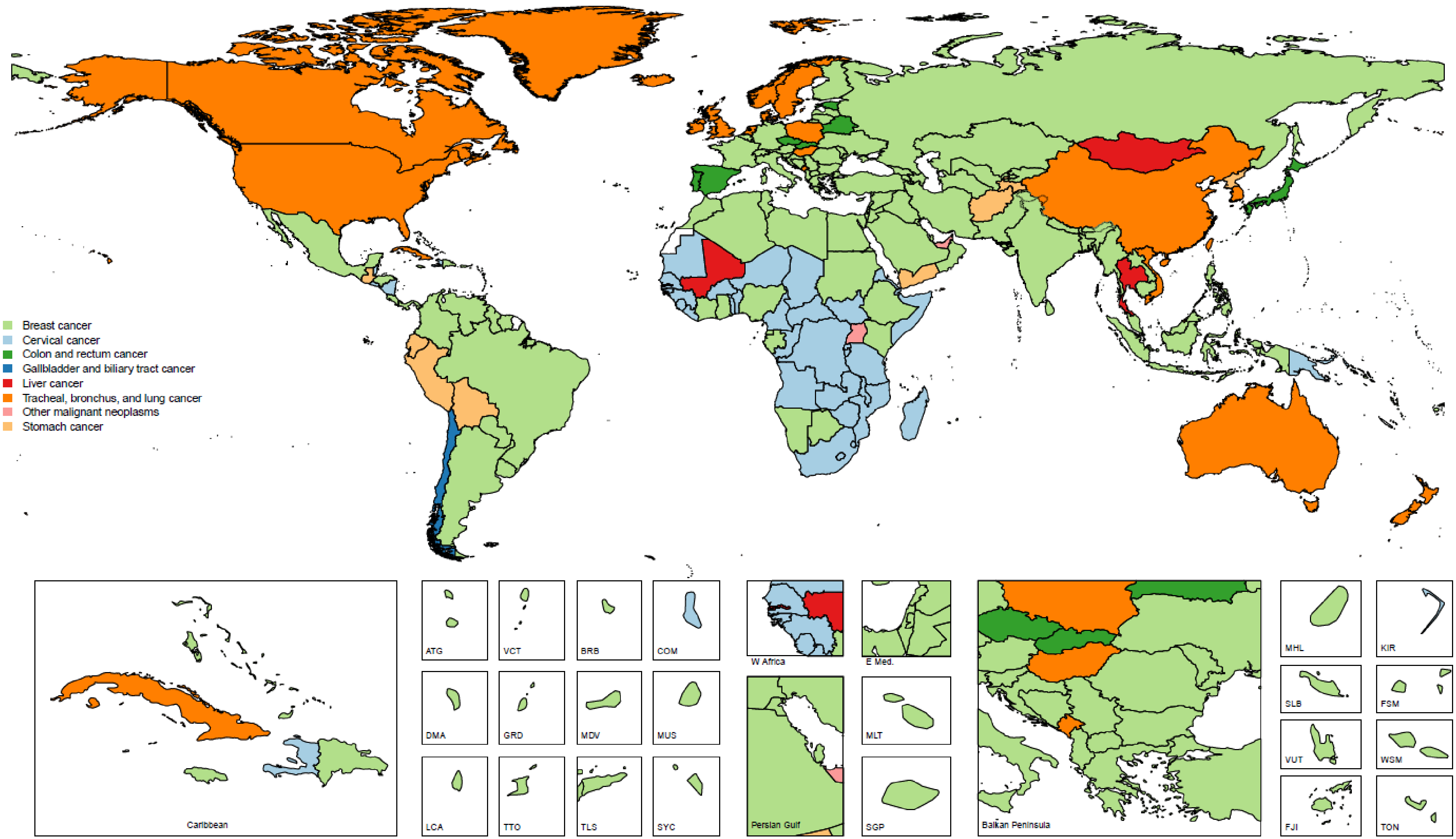

eFigure 10: Top-ranked cancers by absolute deaths for all ages in females, 2017

© 2019 Global Burden of Disease Cancer Collaboration. JAMA Oncol.

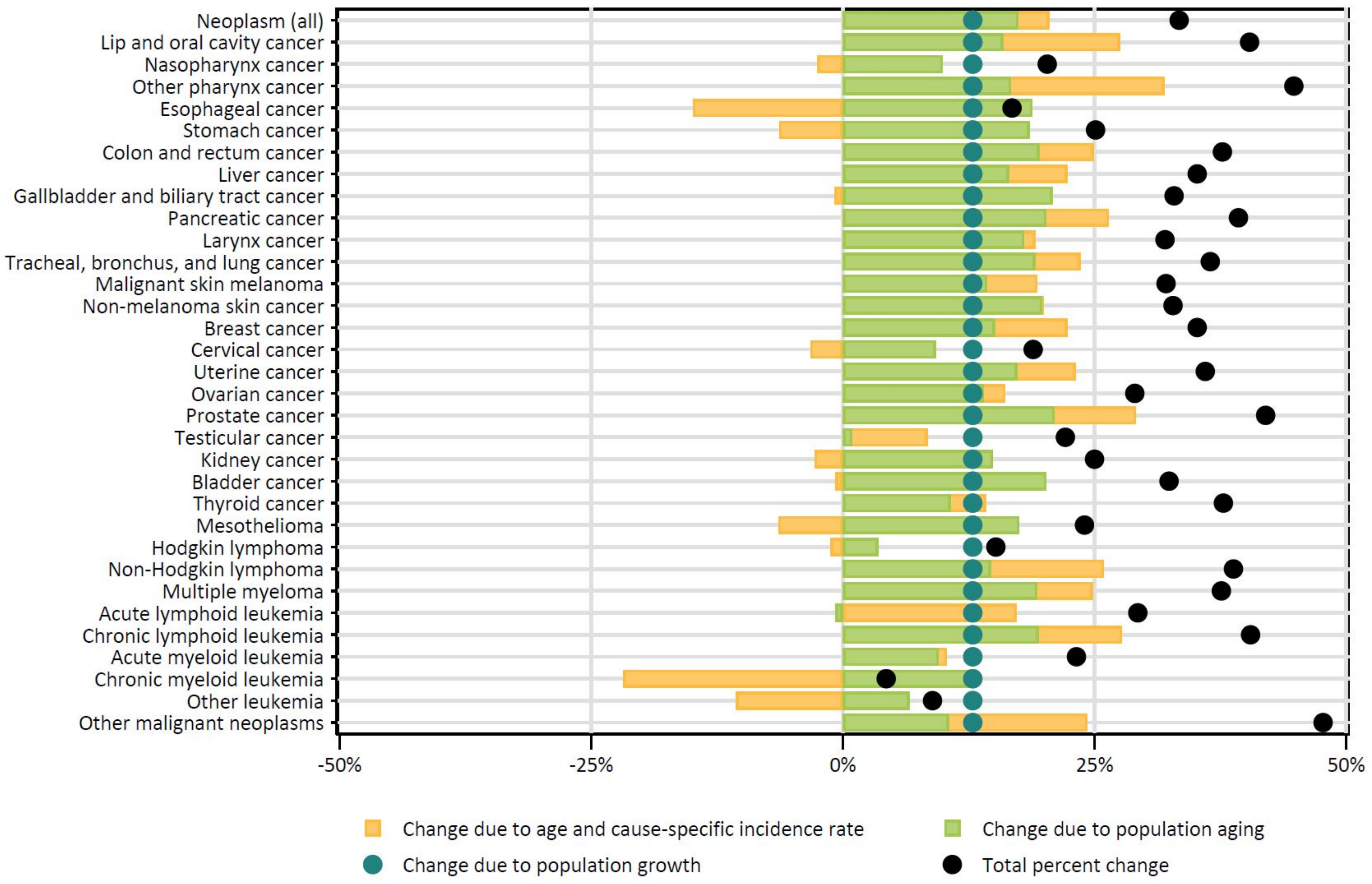

eFigure 11: Global Decomposition of Changes in Cancer Incident Cases due to Population Growth, Population Aging, and Changes in Age-specific Incidence Rates, Both Sexes, 2007 to 2017.

Results are also presented in eTable 14. To estimate the effect of population growth we applied the population size of 2017 onto the rate, sex, and age structure of 2007. Since the global population grew by 12.9% between 2007 and 2017, and rates and age structure remained the same as in 2007, incidence due to all cancers increased by 12.9% in this counterfactual scenario. To estimate the effect of aging on incident cases we applied the age structure of 2017 onto the rate, sex distribution, and population size of 2007. The change in incident cases reported herein shows the proportion of the change in incident cases between 2007 and 2017 that can be attributed to the changing age structure of the population. To estimate the effect of changing incidence rates on the incident cases we applied the incidence rates for 2007 onto the population size and age structure of 2007. The change in incident cases reported herein shows the proportion of the change in incident cases between 2007 and 2017 that can be attributed to a change in incidence rates.

© 2019 Global Burden of Disease Cancer Collaboration. JAMA Oncol.

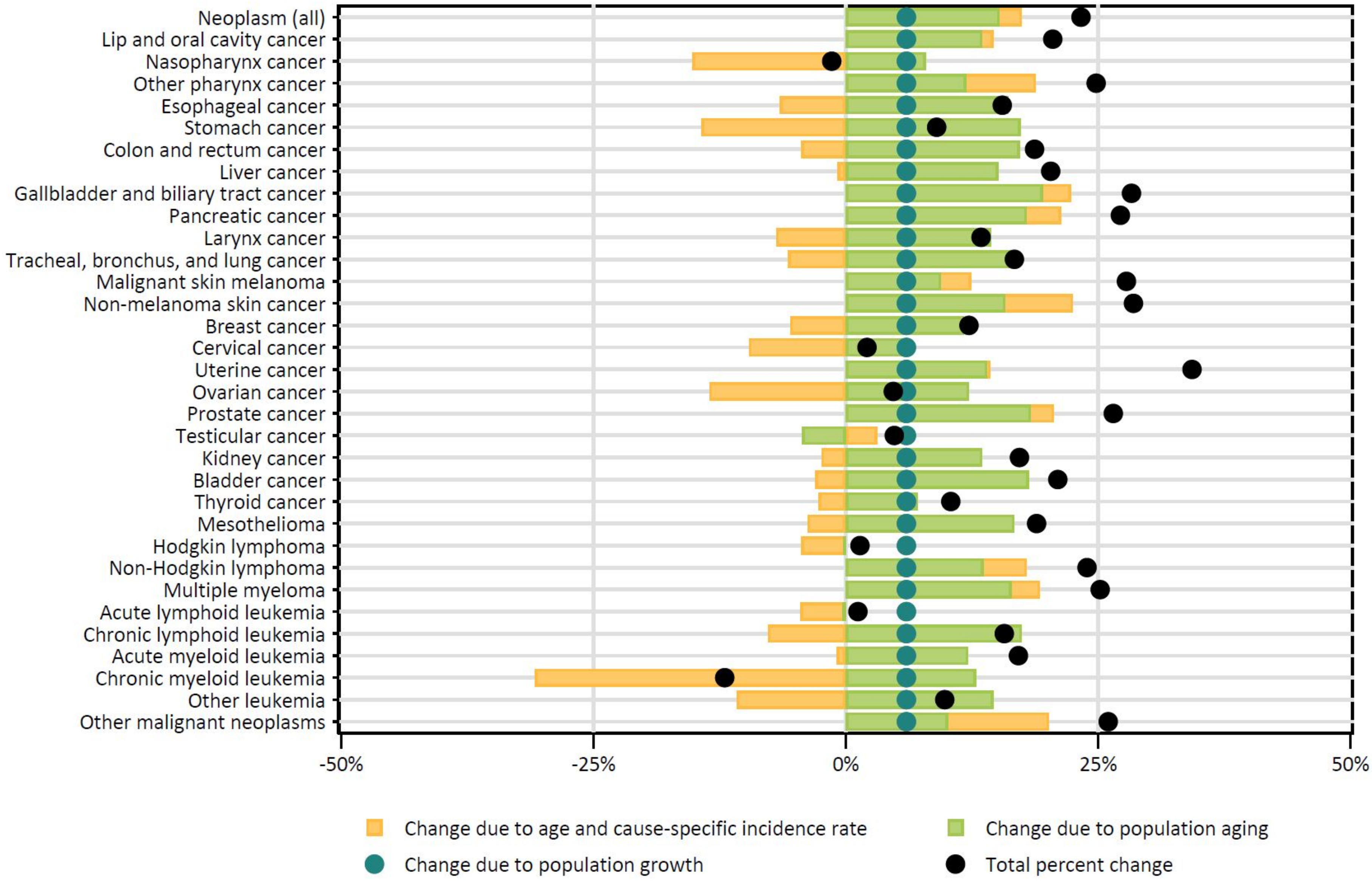

eFigure 12: High SDI Quintile Decomposition of Changes in Cancer Incident Cases due to Population Growth, Population Ageing, and Changes in Age-specific Incidence Rates, Both Sexes, 2007 to 2017.

© 2019 Global Burden of Disease Cancer Collaboration. JAMA Oncol.

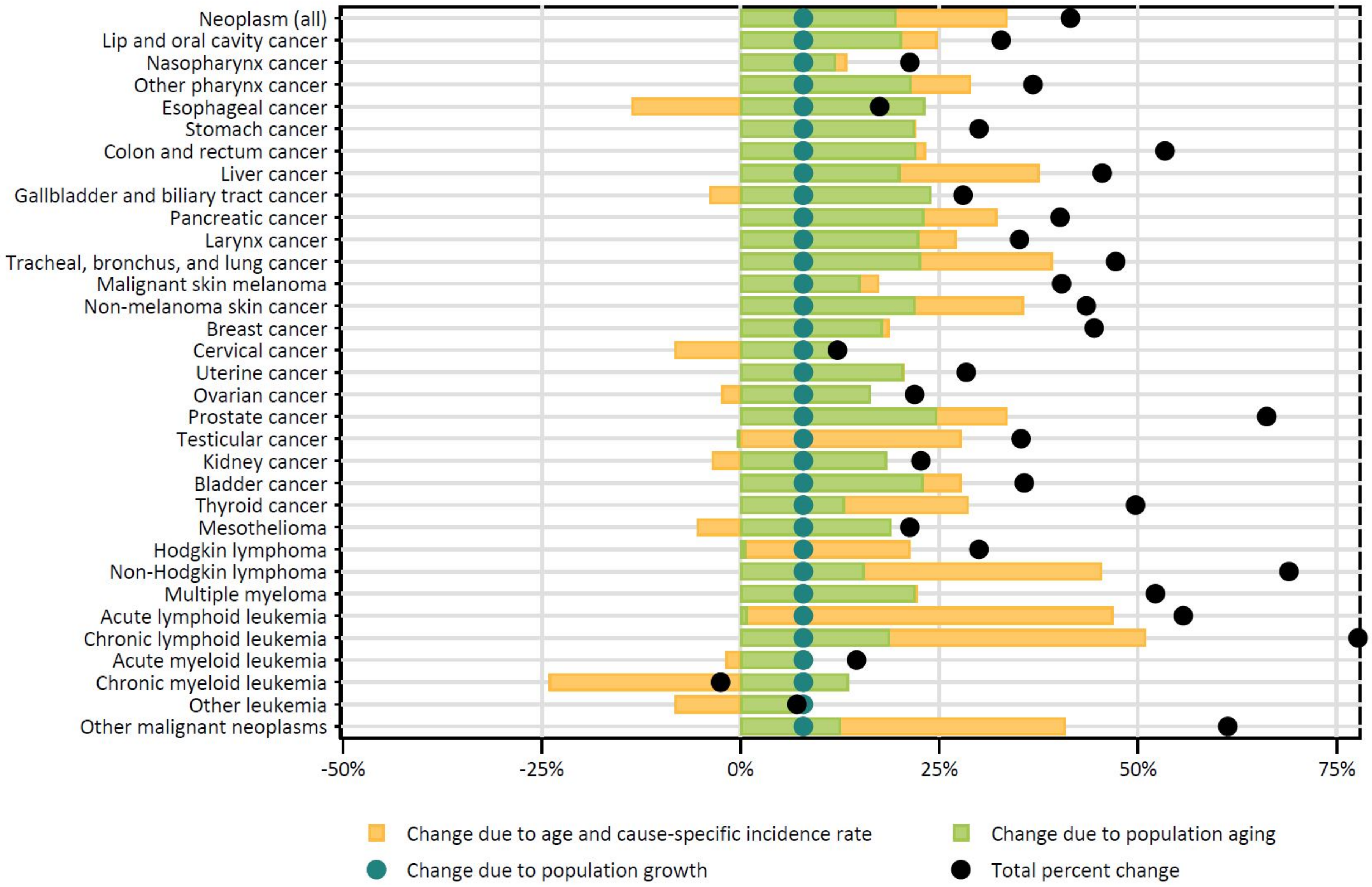

eFigure 13: High-middle SDI Quintile Decomposition of Changes in Cancer Incident Cases due to Population Growth, Population Ageing, and Changes in Age-specific Incidence Rates, Both Sexes, 2007 to 2017.

© 2019 Global Burden of Disease Cancer Collaboration. JAMA Oncol.

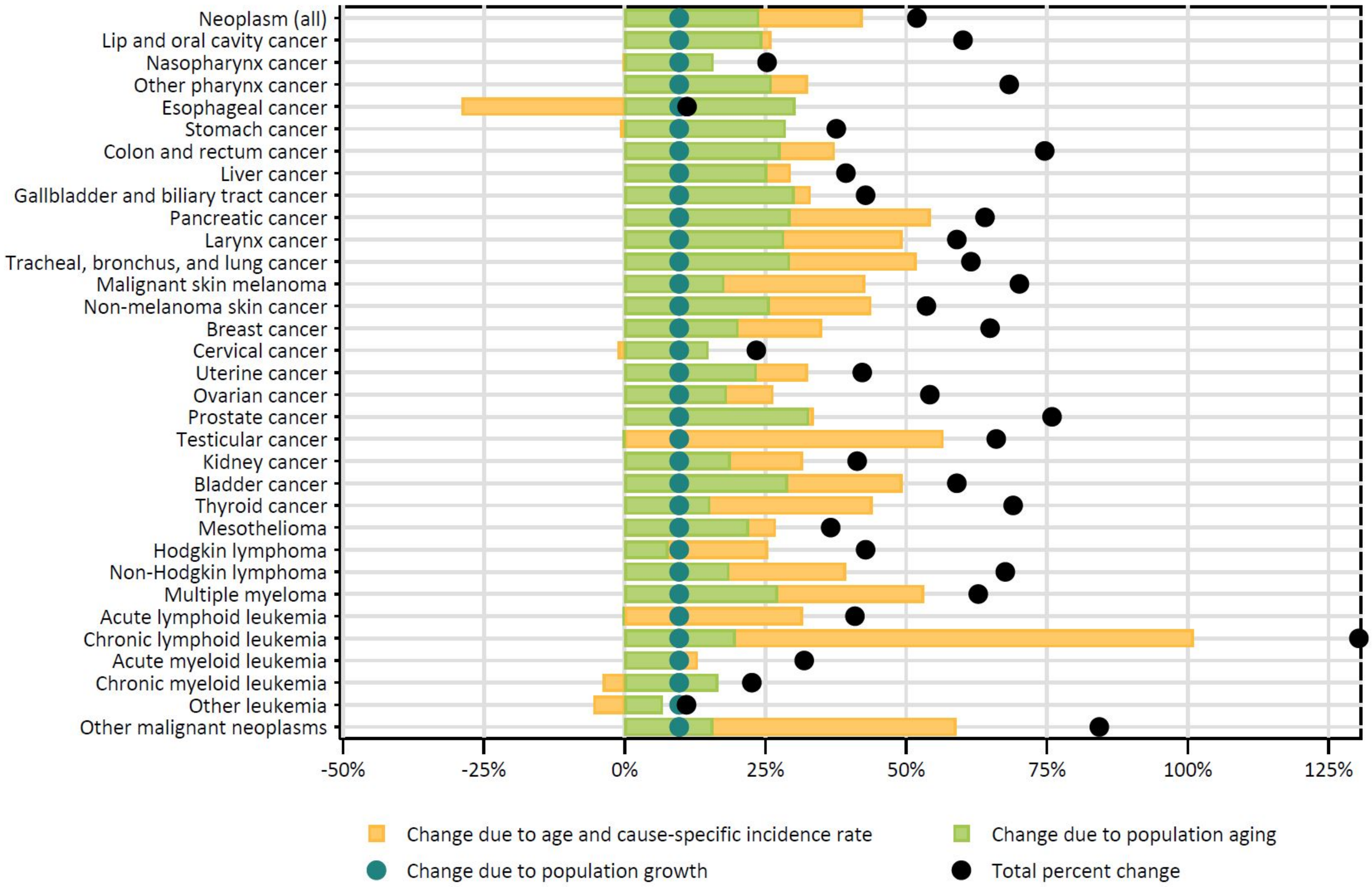

eFigure 14: Middle SDI Quintile Decomposition of Changes in Cancer Incident Cases due to Population Growth, Population Ageing, and Changes in Age-specific Incidence Rates, Both Sexes, 2007 to 2017.

© 2019 Global Burden of Disease Cancer Collaboration. JAMA Oncol.

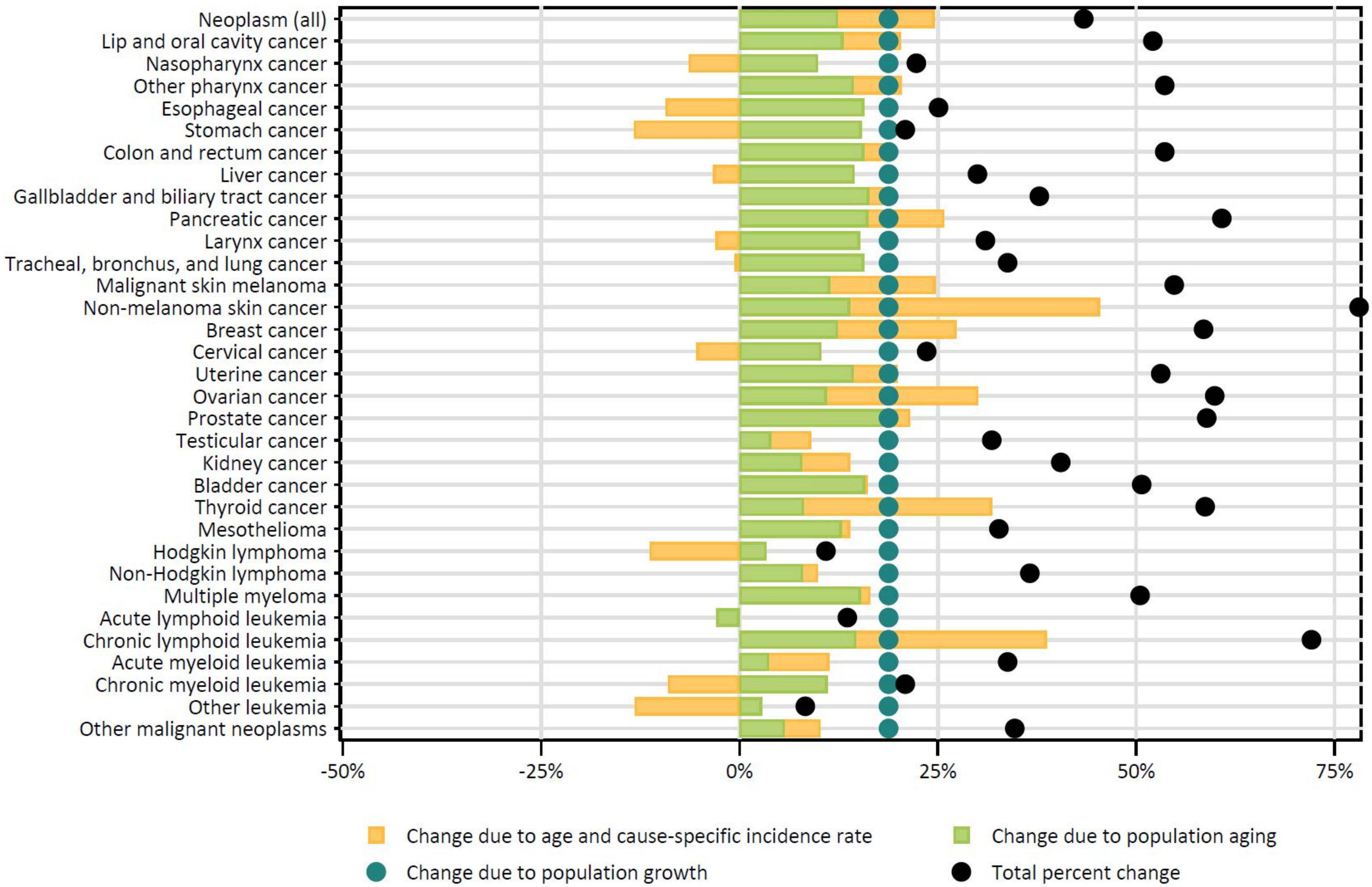

eFigure 15: Low-middle SDI Quintile Decomposition of Changes in Cancer Incident Cases due to Population Growth, Population Ageing, and Changes in Age-specific Incidence Rates, Both Sexes, 2007 to 2017.

© 2019 Global Burden of Disease Cancer Collaboration. JAMA Oncol.

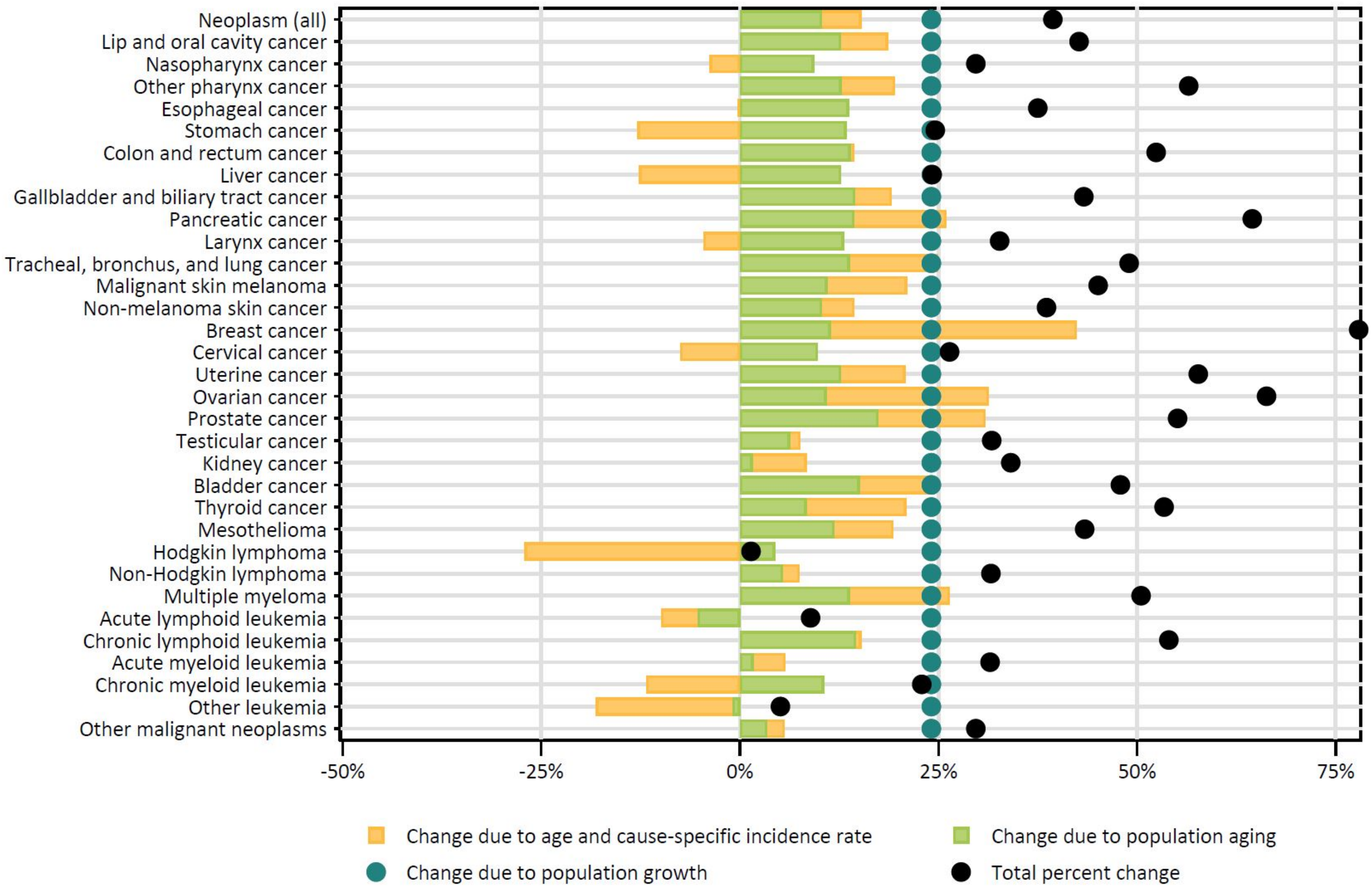

eFigure 16: Low SDI Quintile Decomposition of Changes in Cancer Incident Cases due to Population Growth, Population Ageing, and Changes in Age-specific Incidence Rates, Both Sexes, 2007 to 2017.
